# Reproducible protocol for the extraction and semi-automated quantification of macroscopic charcoal from soil

**DOI:** 10.1371/journal.pone.0304198

**Published:** 2024-07-12

**Authors:** Javier Ruiz-Pérez, Julie C. Aleman, Joseph W. Veldman

**Affiliations:** 1 Department of Ecology and Conservation Biology, Texas A&M University, College Station, Texas, United States of America; 2 Centre Européen de Recherche et d’Enseignement des Géosciences de l’Environnement, Centre National de la Recherche Scientifique, Aix-en-Provence, France; Israel Antiquities Authority, ISRAEL

## Abstract

Charcoal fragments preserved in soils or sediments are used by scientists to reconstruct fire histories and thereby improve our understanding of past vegetation dynamics and human-plant relationships. Unfortunately, most published methods for charcoal extraction and analysis are incompletely described and are therefore difficult to reproduce. To improve the standardization and replicability of soil charcoal analysis, as well as to facilitate accessibility for non-experts, we developed a detailed, step-by-step protocol to isolate charcoal from soil and to efficiently count and measure charcoal fragments. The extraction phase involves the chemical soaking and wet sieving of soils followed by the collection of macrocharcoal (≥500 μm). The analysis phase is performed semi-automatically using the open-source software ImageJ to count and measure the area, length, and width of fragments from light stereo microscope images by means of threshold segmentation. The protocol yields clean charcoal fragments, a set of charcoal images, and datasets containing total charcoal mass, number of fragments, and morphological measurements (area, length, and width) for each sample. We tested and validated the protocol on 339 soil samples from tropical savannas and forests in eastern lowland Bolivia. We hope that this protocol will be a valuable resource for scientists in a variety of fields who currently study, or wish to study, macroscopic charcoal in soils as a proxy for past fires.

## Introduction

In a process called charcoalification, the thermal degradation of plants produces the black, carbon-rich material known as charcoal [1]. After a fire, charcoal fragments are incorporated into soils or sediments (sometimes after being transported by water, wind, or humans), where they can persist for thousands to millions of years; indeed, the oldest charcoal fragments recovered to date are from the late Silurian period (~420 Myr) [2–5]. The study of charcoal in soils and sediments provides valuable information on past fire regimes, climate change, plant community composition, vegetation dynamics, and human-vegetation relationships, including plant use and landscape modification. Charcoal analysis is an established practice in paleoecology that includes the identification of distinct charcoal types, the quantification of charcoal abundance, and the measurement of light absorption/reflectance of charcoal fragments as proxies for different components of past fire regimes, including fire frequency, intensity, severity, and extent [6–15]. These approaches allow researchers to infer spatio-temporal interactions between plant communities and fires, sometimes as a response to climate changes or human activities such as deforestation or agriculture [7, 8, 16, 17]. In archaeological contexts, the examination of charcoal assemblages contributes to our understanding of ancient human use of plants as, for example, food, fuel, and building material [3, 17]. Furthermore, charcoal is extensively used to establish chronological frameworks of past events through radiocarbon dating (i.e., the age determination of charcoal by measuring its carbon-14 content) [18].

Several methods have been developed to reconstruct fire histories using charcoal preserved in soils and sediments. Although the study of soil and sedimentary charcoal is based on slightly different premises, both generally involve a two-phase process of extraction of charcoal fragments followed by analysis of the charcoal assemblages [8, 11, 19]. Conventional extraction techniques include: 1) preparation of petrographic thin sections of sediment [20, 21]; 2) recovery of charcoal together with pollen grains from sedimentary records using standard pollen extraction procedures [20, 22]; 3) dispersion (and occasionally bleaching or digestion) of soil or sediment followed by filtration through one or various nested sieves [23–26]; 4) density separation of sediments using heavy liquid with a specific gravity of 2.2 g/cm^3^ with optional bleaching [27]; 5) water flotation of soils [28]; 6) dry or wet sieving of soils [28, 29]; and 7) hand-picking or hand-sorting soil charcoal under binocular magnifying lens [28, 29]. The analysis of extracted charcoal may then proceed in a variety of ways to gather information on fire regimes, fuel types, and chronologies of fire occurrence. In addition to the taxonomic analysis of charcoal assemblages and radiocarbon dating of charcoal fragments, the most common approach to analyze charcoal in paleofire reconstructions is through quantification of the number of fragments and 2-dimensional morphological measurements, such as fragment area, length, and width [6, 7, 18, 19, 30, 31].

Charcoal quantification (i.e., counting and measurement of charcoal fragments) is usually performed with the naked eye, a stereo microscope, or a compound microscope, depending on the size of the charcoal fragments. Manual analysis of charcoal is a time-consuming and subjective task that can be replaced with semi-automated or fully automated analysis of microscope images or live videos using a microscope camera and image software with integrated digital measurement tools. Image thresholding (i.e., classification of pixels into two classes using a cut-off value to differentiate dark charcoal fragments from the background) and automated counting and measurement of isolated charcoal is a conventional method, often carried out using software such as the open-source ImageJ or the proprietary WinSEEDLE (Regent Instruments Inc.) [32–38]. In CharTool, a suite in ImageJ, users first select each charcoal fragment manually, then the software automatically delimits the fragment by edge detection (i.e., recognition of boundaries between objects based on pixel intensity values and variation across neighboring pixels), and finally collects 21 morphometric measurements (e.g., area, perimeter, circularity) [39]. In addition to thresholding and edge detection, convolutional neural networks, a machine learning method used in computer vision, have provided promising results in charcoal detection and morphological classification [40].

While a variety of methods for extracting and quantifying charcoal exist, including several detailed protocols [24, 26, 32, 41, 42], we are unaware of any published protocols that comprehensively and precisely detail both the extraction and quantification steps combined. Moreover, methods are seldom described in publications containing charcoal data, which limits the reproducibility of results and the accessibility by non-experts or junior researchers to charcoal research [43]. We came to these conclusions after conducting a systematic review of the methods employed in charcoal studies. We assessed the reporting practices of procedures to extract and analyze charcoal fragments from either soil or sediment samples in 100 publications randomly selected from the existing literature (see S1 Appendix for details on the assessment methodology and S1 File to access the results [44]). Our review, which included publications from 1986 through 2023, showed that sieving is the most common technique to isolate macroscopic charcoal (74%, *n* = 100). Furthermore, we found that most quantification analyses (i.e., counting and measurement) are done manually (82%, *n* = 94), despite the availability of (semi-)automatic image processing methods since the early 1990s [33, 35, 39, 40]. We also confirmed methodological gaps in reporting: none of the studies reported complete protocols for extraction and analysis, whether cited or described in detail. Among details omitted, 94% of studies lacked detail on how samples were treated before being processed and 72% did not sufficiently describe how charcoal was isolated (*n* = 100). Of studies that used chemicals during the extraction (*n* = 49), 94% did not specify how chemicals were applied. Of studies that quantified charcoal after isolation (*n* = 99), 41% did not provide details about how the analysis was conducted. Of the publications in which image software was used for quantification (*n* = 12), none provided the code, details of the workflow, or software configuration.

**Fig 1:**
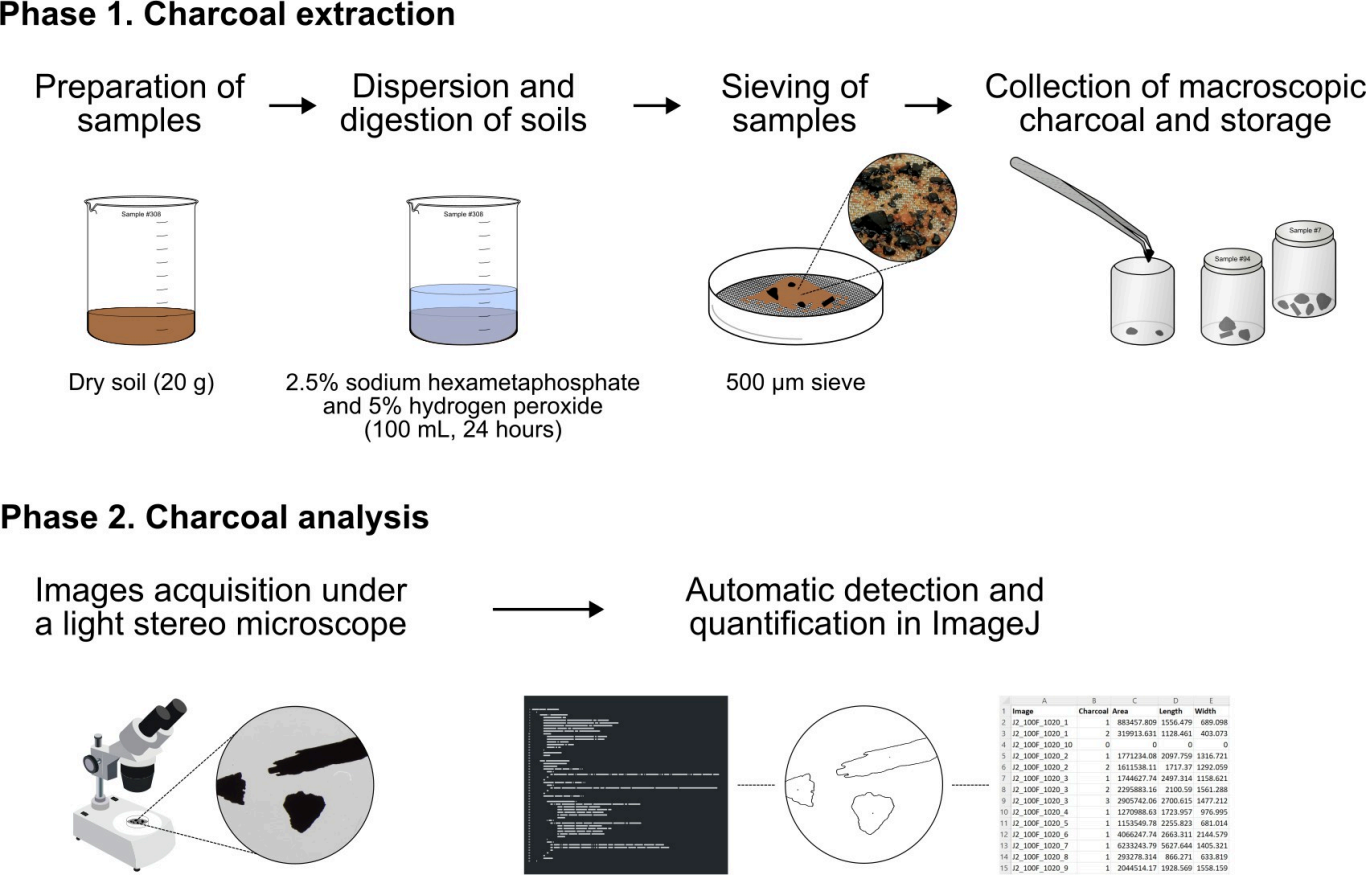
Diagram of the protocol showing the main steps of the charcoal extraction and analysis phases.

In this paper, we present in detail a simple, reproducible, step-by-step protocol for the extraction, counting, and measurement of charcoal fragments from soil samples as a proxy for past fires. It is intended for both scientists with experience in the study of charcoal as well as for novices who wish to learn and implement these techniques. The protocol consists of two major phases: extraction of charcoal and analysis of fragments (Fig 1). First, the extraction entails the dispersion and digestion of the soil, washing it through a sieve, and collecting the isolated charcoal fragments. Second, the quantification involves taking stereo microscope images of the extracted charcoal and the automated counting and measurement of the fragments using an ImageJ macro provided with the protocol.

The extraction phase of the protocol disperses and digests 20 g of dry soil for 24 hours in a 100 mL solution of 2.5% sodium hexametaphosphate (Na_6_P_6_O_18_) and 5% hydrogen peroxide (H_2_O_2_)—prepared as a 1:1 mixture of 50 mL of 5% sodium hexametaphosphate and 50 mL of 10% hydrogen peroxide. Then, to separate and concentrate macroscopic charcoal fragments from finer soil particles, the soil is rinsed through a 500-micron mesh sieve. Previous charcoal studies have used a variety of different chemicals to disperse, digest, and bleach soils and sediments prior to isolation; among them, mild concentrations of sodium hexametaphosphate and hydrogen peroxide have proven to be safe for charcoal produced at moderate to high temperatures (i.e., > 400°C) and are effective in organic- and clay-rich soils and sediments [24, 45–48]. But note that, for studies interested in recovering charcoal produced at low temperatures (i.e., 250–400°C), oxidants like hydrogen peroxide should be avoided or milder concentrations and exposure times should be tested beforehand [49, 50]. Additionally, to obtain consistent extractions in case some aspect of the protocol causes any bias, soil samples should always receive the same treatment (e.g., consistent soil dry weight and identical chemical volume, concentration, and exposure times) [24, 46, 47]. When compared to other extraction methods, the protocol’s sieving technique is fast, reliable, and minimizes mechanical pressure that can damage charcoal fragments [24, 27, 51]. In our protocol, we preferred a 500-micron mesh over smaller sizes to capture only large macrocharcoal for two main reasons. First, macrocharcoal (≥500 μm) tends to be incorporated into the soil within a few meters of where it was produced, as opposed to smaller charcoal that is more readily transported long distances [2, 13, 14, 52, 53]. Second, smaller charcoal may originate from larger fragments affected by taphonomic processes, which would bias the interpretation of fragment counts and sizes.

For the analysis of charcoal fragments in ImageJ [38], we wrote a macro code to batch process charcoal images, detect fragments via threshold segmentation, and then calculate the number, area, length, and width of the fragments. Even though such an automated approach is gaining importance, researchers tend not to make macros publicly accessible [36], design macros for specific analyses that are difficult to customize for other contexts [35], or otherwise do not describe methods with reproducible detail [34, 37, 46, 54, 55]. For this reason, we have made available the ImageJ macro code in this protocol. It is a simple and short code that can be easily modified if desired (e.g., to incorporate additional measurements). The code generates a user-friendly interface that allows analysts to set the scale of the images in different units (i.e., microns, mm, and cm), choose between manual and automatic threshold methods (i.e., custom adjustment of the minimum and maximum threshold values or calculation of threshold values with algorithms included in ImageJ), to activate watershed segmentation (i.e., automatic separation of fragments in contact), and to set automatic filling of holes within charcoal [56].

## Materials and methods

### Ethics statement

This study was approved by the Bolivian Ministry of the Environment and Water under permit number CAR/MMAYA/VMABCCGDF/DGBAP/MEG N° 0286/2022. Soil samples were shipped to the Laboratory of Fire Ecology and Savanna Conservation at Texas A&M University (College Station, Texas, USA) in accordance with permit number P330-22-00089 issued by the Animal and Plant Health Inspection Service of the United States Department of Agriculture.

### Protocol

The protocol described in this peer-reviewed article is published on protocols.io (https://dx.doi.org/10.17504/protocols.io.kxygx9xr4g8j/v1) and is included for printing as S1 Protocol with this article.

## Expected results and validation

**Fig 2:**
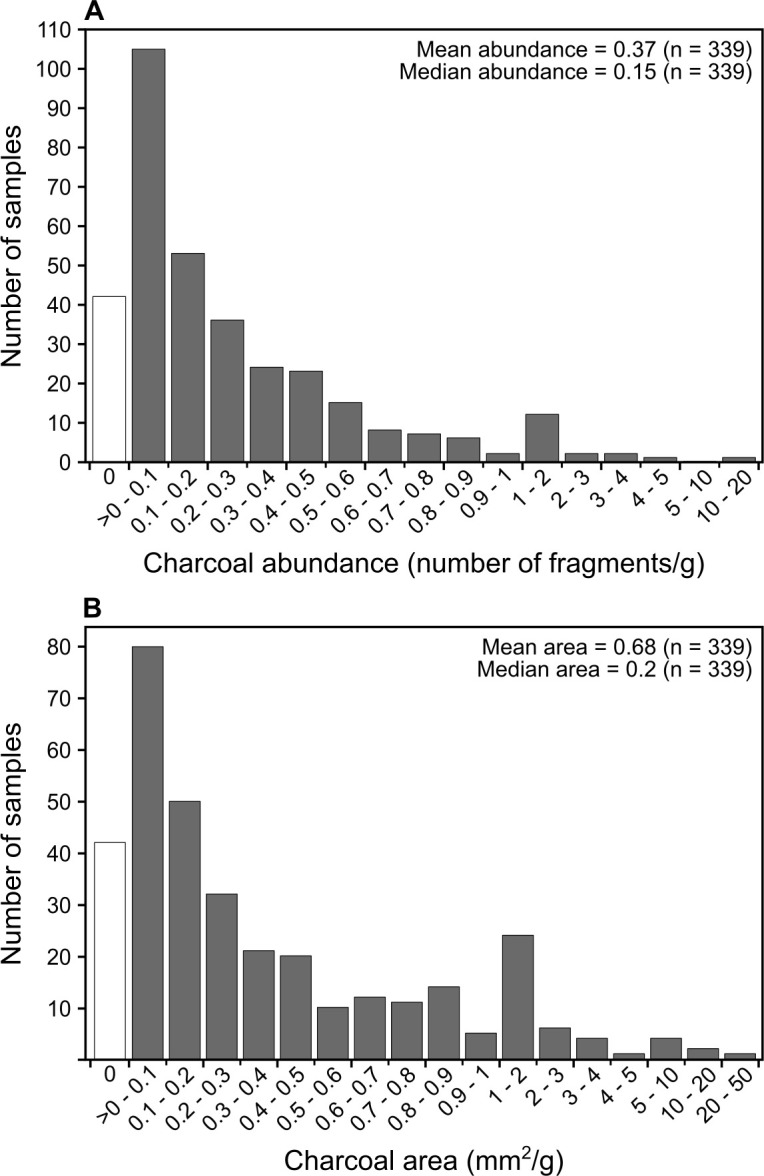
Histograms of charcoal abundance and size obtained from 339 test soil samples. (A) Abundance expressed in number of fragments counted per gram of dry soil. (B) Total area expressed in mm^2^ per gram of dry soil. White bars correspond to samples without charcoal and gray bars represent samples containing charcoal. Note that the interval values in the x-axes are non-linear.

We used the protocol to process 339 soil samples corresponding to 18 cores of ~2 m depth collected in tropical dry forests and savannas from the Chiquitania region of eastern lowland Bolivia (see S2 Appendix for more details on the samples [44]). Of the 339 samples, 297 contained charcoal, from which we obtained a total of 2,480 fragments ≥500 μm. Overall, the samples exhibit considerable variation in anthracomass (i.e., total mass of extracted charcoal), number of fragments, and size. Charcoal mass ranged from 0 to 23.3 mg per gram of dry soil, with a mean of 0.4 and median of 0.1. Charcoal abundance ranged from 0 to 18.9 fragments per gram of dry soil, with a mean of 0.4 and a median of 0.2 (Fig 2A). Total charcoal area ranged from 0 to 33.5 mm^2^ per gram of dry soil, with a mean of 0.7 and a median of 0.2 (Fig 2B).

**Fig 3:**
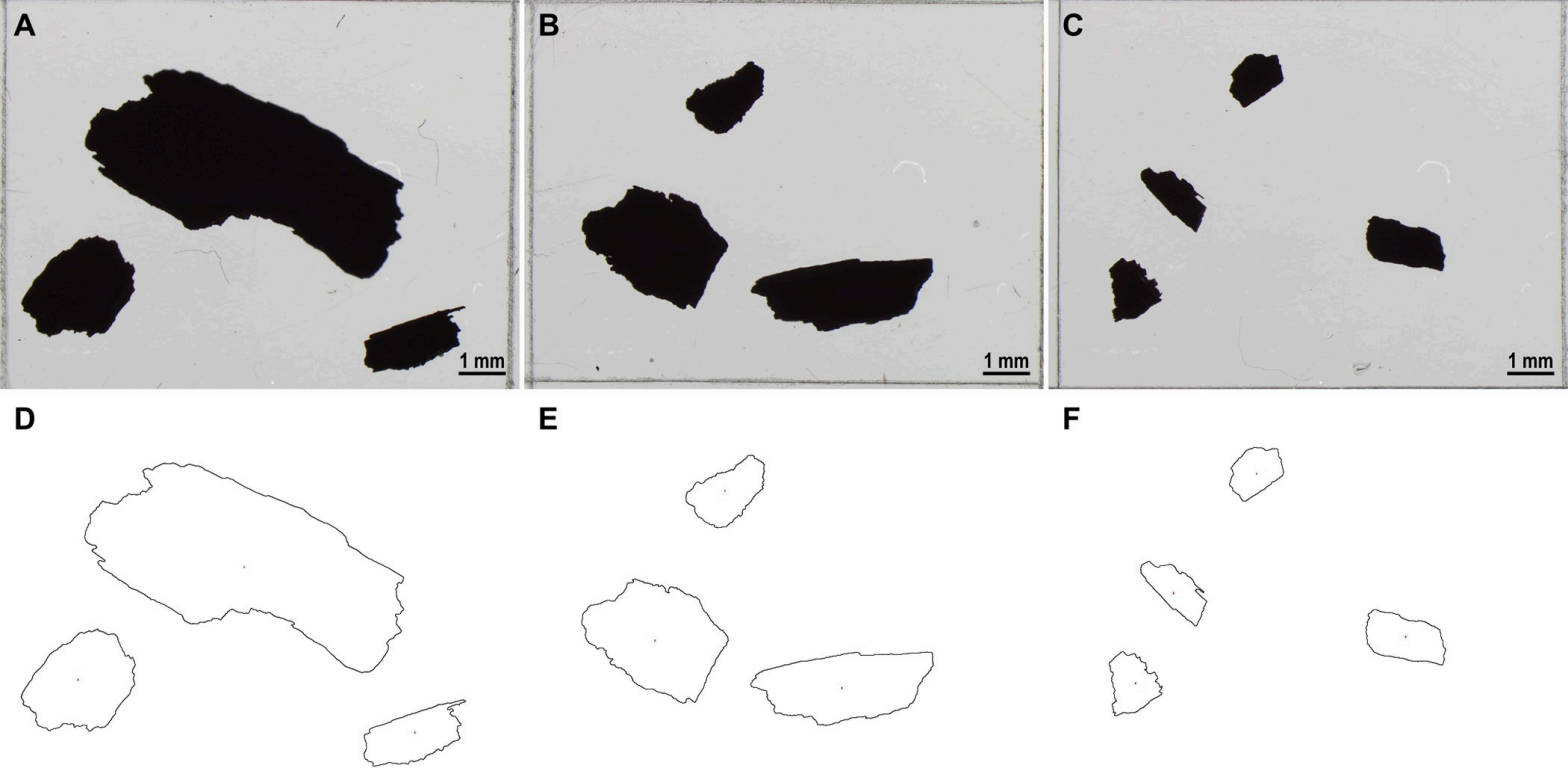
Microscope images of charcoal fragments and outline images generated by the ImageJ macro. Example of charcoal fragments under the light stereo microscope in transmitted light at 12x magnification (A, B, C), and the resulting outline images after the analysis (D, E, F, respectively).

To assess the effectiveness of the methodology and to give examples of the data that can be produced with the protocol, we randomly selected a subset of 100 of the 339 samples processed. First, we provide results of the extraction of charcoal fragments as the dry weight of the initial soil sample and the dry weight of the isolated fragments in S2 File [44]. These weights are typically used to calculate charcoal concentration per unit of soil mass or volume [28]. Second, we provide 463 JPG images taken under a light stereo microscope of the extracted charcoal along with an empty image with a scale bar in S1 Dataset [44]. We collected images at 12x magnification using an Olympus SZX7 stereo microscope (eyepiece WHSZ10X-H, objective DFPL1.5X-4) and an Olympus LC30 camera (C-mount adapter U-TV0.5XC-3). These images are used by the ImageJ macro code of the protocol to analyze charcoal (see example in Fig 3) and can also be used for additional measurements or validation of results (as reported below). Third, in S2 Dataset [44], we provide the raw output files that generates the macro: 1) a CSV file that reports the analysis parameters, date of analysis, and software versions; 2) a CSV file with the counting and measurement results; and 3) outline images in JPG format of the charcoal fragments detected. Count data can be processed to generate a file with the total number of fragments per sample as in S3 File, while the measurements of each fragment (i.e., area, length, and width) can be saved as in S4 File [44]. Number and area are standard metrics utilized to reconstruct fire regimes [6–8, 19], whereas length to width ratio offers information about the type of fuel (i.e., grasses tend to produce longer and thinner charcoal fragments than woody plants) [30, 54, 57, 58]. Note that this protocol provides data as concentration per unit mass of dry soil and that the age determination of the soil samples would be required to establish a chronological framework for the reconstruction and interpretation of fire histories [59, 60]. Outline images help to determine the accuracy of the charcoal fragments detected in ImageJ and to control for any extraneous particles that might be erroneously identified as charcoal.

**Fig 4:**
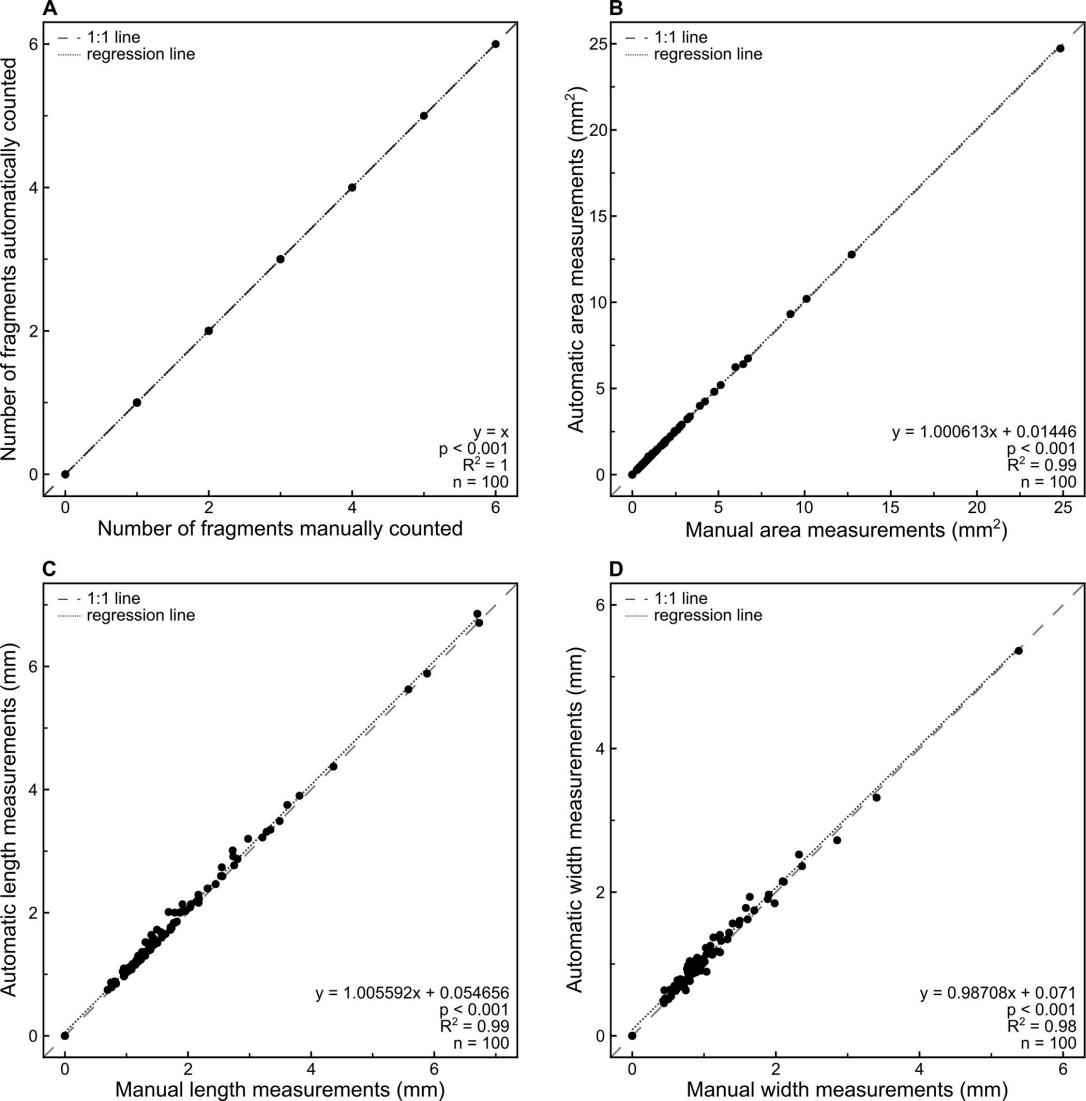
Validation of the results obtained with the ImageJ macro against manual quantification. (A) Abundance of charcoal fragments in 100 images. (B) Area of 100 fragments expressed in mm^2^. (C) Length of 100 fragments expressed in mm. (D) Width of 100 fragments expressed in mm.

We validated the count and measurement data produced automatically by the ImageJ macro against manual measurements of 100 fragments (S5 File [44]). We randomly selected 100 fragments from S4 File and collected the corresponding light stereo microscope images from S1 Dataset [44]. We first counted all charcoal fragments ≥500 μm on each image and then manually measured each selected fragment in ImageJ (version 1.54d for Microsoft Windows with Java version 1.8.0_345). In *Analyze -> Set Scale*, we set the scale values using the same parameters as the defaults in the macro of the protocol: distance in pixels of 185.75, known distance of 1000, and unit of length in microns. We opened each image separately and measured the length and width of each fragment using the tool *Straight Line* (i.e., fifth tool from the left on the ImageJ toolbar) and the area by drawing the perimeter with the tool *Polygon Selections* (i.e., third tool from the left on the ImageJ toolbar). We recorded every measurement in a results table by pressing *Ctrl + M* after drawing each line and perimeter. In R (version 4.1.1 for Microsoft Windows) [61], we analyzed the relationship between the automatic and manual quantifications using simple linear regressions (Fig 4). We found a perfect fit for number of fragments and high correlations for area (*F*(1, 98) = 678,400, *p* = <0.001, *R*^*2*^ = 0.99), length (*F*(1, 98) = 30,390, *p* = <0.001, *R*^*2*^ = 0.99) and width (*F*(1, 98) = 7,443, *p* = <0.001, *R*^*2*^ = 0.98). These results confirm that the macro generates robust values, which have a 1:1 relationship or close to it, depending on the metric, to those obtained manually. The obvious difference between the macro and measuring each fragment manually was that the later took 1–5 minutes on average per fragment, varying with the fragment size and shape, while in ImageJ it only required ~0.2 seconds on average per fragment on a computer with an AMD Ryzen 7 PRO 5750GE 3.20 GHz, 32 GB of RAM, and Microsoft Windows 10 version 22H2.

## Conclusion

The protocol we present in this paper offers a simple step-by-step guide to isolate charcoal fragments from soil samples and obtain data necessary to interpret charcoal assemblages as a proxy for past fires. It is fast and inexpensive, preserves macrocharcoal fragments, and provides unbiased, accurate charcoal counts, and measurement data. After completion of the protocol, charcoal mass, number of fragments, and morphological measurements (i.e., area, length, and width) are generated from each sample (e.g., supporting files mentioned above). In addition, the set of charcoal images collected for analysis in ImageJ, together with the outline images produced after analysis, constitute a valuable record for prospective (re)analyses, validation controls, and reproducibility assessments, particularly if these images are made available as raw data upon publication of the results. Furthermore, the protocol produces clean extractions of charcoal, which can be archived for future analyses, such as radiocarbon dating, morphological or taxonomic identifications, and fire temperature estimations [3, 9, 10, 17, 18, 62, 63]. Another added advantage to the protocol is that the extraction procedure and the counting and measurement method are independent, and therefore the extraction protocol can be used for purposes other than quantifying charcoal fragments (e.g., radiocarbon dating), while the counting and measurement method can be performed on charcoal samples obtained following other extraction techniques (e.g., flotation, density separation).

Despite the strengths of the protocol, it is also subject to some limitations. First, as with any oxidant, the hydrogen peroxide used in the protocol should be avoided or tested before use to avoid damaging charcoal formed at ≤400°C [49, 50]. Second, given that the thresholding technique relies on fixed cut-off values, the results of the image analysis should always be supervised to control for possible over- or underestimation of the charcoal size and false positives (i.e., dark particles other than charcoal automatically classified as charcoal) that need to be removed [19, 37]. Third, while the four metrics collected by the ImageJ macro in this protocol are among the most utilized in charcoal studies [6, 7, 19], anyone wishing to collect additional morphological metrics would need to incorporate these into the code. Finally, the data files generated by the ImageJ macro may require additional preparation for analysis (e.g., sum the fragments identified to obtain the total number of fragments per sample) or for archiving in a data repository (e.g., generate metadata files to make results reusable and interoperable).

## Supporting information

S1 Protocol Step-by-step protocol, also available on protocols.io. Guide to extract and semi-automatically count and measure charcoal fragments from soil samples.
(PDF)

S1 Appendix (DOCX)

S2 Appendix (DOCX)

S1 Dataset (ZIP)

S2 Dataset (ZIP)

S1 File (CSV)

S2 File (CSV)

S3 File (CSV)

S4 File (CSV)

S5 File (CSV)

S6 File (CSV)

S7 File (CSV)

S8 File (CSV)

S9 File (CSV)

S10 File (CSV)
