# Supplementary material for: Reproducible protocol for the extraction and semi-automated quantification of macroscopic charcoal from soil
Source: PLoS One. 2024 Jul 12;19(7):e0304198. doi: 10.1371/journal.pone.0304198 (PMC11244820; doi:10.1371/journal.pone.0304198)
Supplement: S1 Protocol — Guide to extract and semi-automatically count and measure charcoal fragments from soil samples. (PDF) [file pone.0304198.s001.pdf]

May 06, 2024

Reproducible protocol for the extraction and semi-automated quantification of macroscopic charcoal from soil

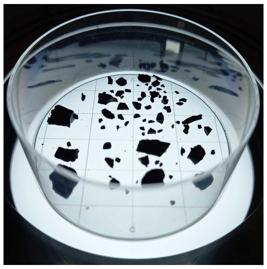

DOI
dx.doi.org/10.17504/protocols.io.kxygx9xr4g8j/v1

Javier Ruiz-Pérez[1], Julie C. Aleman[2], Joseph W. Veldman[1]

[1]Department of Ecology and Conservation Biology, Texas A&M University, College Station, Texas 77843-2258, USA;
[2]Centre Européen de Recherche et d'Enseignement des Géosciences de l'Environnement, Centre National de la Recherche Scientifique, 13545 Aix-en-Provence, France

Javier Ruiz-Pérez: jruizperez@tamu.edu, javier.ruizperez.academic@gmail.com;

Javier Ruiz-Pérez
Texas A&M University - College Station

OPEN ACCESS

DOI: dx.doi.org/10.17504/protocols.io.kxygx9xr4g8j/v1

Protocol Citation: Javier Ruiz-Pérez, Julie C. Aleman, Joseph W. Veldman 2024. Reproducible protocol for the extraction and semi-automated quantification of macroscopic charcoal from soil. **protocols.io** https://dx.doi.org/10.17504/protocols.io.kxygx9xr4g8j/v1

License: This is an open access protocol distributed under the terms of the **Creative Commons Attribution License**, which permits unrestricted use, distribution, and reproduction in any medium, provided the original author and source are credited

Protocol status: Working

Created: April 05, 2023

Last Modified: May 06, 2024

Protocol Integer ID: 80056

Keywords: Charcoal analysis, Pedoanthracology, Paleofire, Fire ecology, Automatic detection, ImageJ

Funders Acknowledgement:
National Science Foundation
Grant ID: DEB-1931232
USDA-NIFA McIntire-Stennis
Grant ID: Project 1016880

Disclaimer

USE AT YOUR OWN RISK

Any action you take or refrain from taking using or relying upon the information presented in this protocol is strictly at your own risk. You agree that neither the Company nor any of the authors, contributors, administrators, or anyone else associated with protocols.io 1) can be held responsible for your use of the information contained in or linked to this protocol or any of our Sites/Apps and Services; 2) can be liable for any special, incidental, indirect, or consequential damages whatsoever arising out of or in connection with your access or use the protocol; and 3) can be liable to you or anyone else for any decision made or action taken in reliance on the information given by the protocol or for any consequential, special or similar damages, even if advised of the possibility of such damages.

Abstract

Charcoal fragments preserved in soils or sediments are used by scientists to reconstruct fire histories and thereby improve our understanding of past vegetation dynamics and human-plant relationships. Unfortunately, most published methods for charcoal extraction and analysis are incompletely described and are therefore difficult to reproduce. To improve the standardization and replicability of soil charcoal analysis, as well as to facilitate accessibility for non-experts, we developed a detailed, step-by-step protocol to isolate charcoal from soil and to efficiently count and measure charcoal fragments. The extraction phase involves the chemical soaking and wet sieving of soils followed by the collection of macrocharcoal (≥500 μm). The analysis phase is performed semi-automatically using the open-source software ImageJ to count and measure the area, length, and width of fragments from light stereo microscope images by means of threshold segmentation. The protocol yields clean charcoal fragments, a set of charcoal images, and datasets containing total charcoal mass, number of fragments, and morphological measurements (area, length, and width) for each sample. We tested and validated the protocol on 339 soil samples from tropical savannas and forests in eastern lowland Bolivia. We hope that this protocol will be a valuable resource for scientists in a variety of fields who currently study, or wish to study, macroscopic charcoal in soils as a proxy for past fires.

Image Attribution

Javier Ruiz-Pérez

Materials

Equipment

- Oven (Thermo Scientific Heratherm OGS 180)
- Orbital shaker (Bellco Glass 7744-01000) with 250/300 mL clamps (Bellco Glass 7744-25016)
- Electronic analytical balance with readability of 0.1 mg (Mettler Toledo ME54E)
- Light stereo microscope (Olympus SZX7 with eyepiece WHSZ10X-H, objective DFPL1.5X-4, light source illuminator SCHOTT KL 1600 LED, and ringlight SCHOTT A08600) and camera (Olympus LC30 with C-mount adapter U-TV0.5XC-3)
- Sieve, 20 cm in diameter with 500-micron mesh (Gilson V200SF 500U)
- Glass beakers (200 mL, Pyrex 1000-250)
- Plastic beaker (5 L, United Scientific 11117)
- Plastic Petri dishes, 50 mm in diameter (Sterilin 502014-07P)
- Plastic funnel, 20 cm in diameter (United Scientific 57231)
- Wash bottle (500 mL, VWR 414004-227)
- Tweezers (Cole-Parmer 07398-12)
- Chattaway micro spatula (Eisco S80826)

Chemicals

- 5% sodium hexametaphosphate ($Na_6P_6O_{18}$, Sigma-Aldrich 305553)
- 10% hydrogen peroxide (H_2O_2, Lab Alley HPL30, 30% diluted to 10%)
- 99% isopropyl alcohol (C_3H_8O, USA Lab 810047120186)
- Purified water (deionized H_2O)

Consumables

- Sample containers (30 mL, Dynalon 226254-1500)

Software

- Microscope imaging software (Olympus cellSens Entry 2.3 [build 18987])
- ImageJ version 1.54d with Java version 1.8.0_345

Safety warnings

Always wear laboratory personal protective equipment (PPE), work under a chemical fume hood, and take extra precaution when handling hazardous materials. Review the safety data sheets from the manufacturer of all chemicals when following this protocol. Consult with your institution's health and safety department for specific recommendations and policies.

Before start

The extraction procedure and the counting and measurement method are independent, and therefore the extraction phase can be used for purposes other than counting and measuring charcoal fragments (e.g., radiocarbon dating), while the quantification phase can be performed on charcoal samples obtained following other extraction procedures.

For reference, a list of equipment, chemicals, consumables, and software that we used to develop and test this protocol is provided in the Materials section.

Charcoal extraction

1 **Preparation of samples**

1.A. Oven-dry soil samples to constant weight at 40 °C.

1.B. Label ≥200 mL beakers.

1.C. Place 20 g of each soil sample in a beaker and record weight (after taring the weight of the beaker).

Note

1.A. To prevent clays from hardening and avoid potential charcoal breakage, we suggest not exceeding 40 °C for drying. Samples are ready when they stop losing weight by evaporation, which can be checked by repeated weighing on an analytical balance. Although drying samples is not strictly necessary, it is recommended to obtain precise calculations of charcoal concentrations and improve consistency in the ratio of soil material to chemical solution in step 2.A.

1.B, 1.C. We developed the extraction procedure in this protocol on tropical soil samples with a mean of 7.3 and a median of 3 charcoal fragments ≥500 µm in 20 g of dry topsoil and subsoil samples (*n* = 339). We recommend adjusting the sample mass or volume proportionately to the expected charcoal content and desired number of fragments to recover. If sample mass or volume is modified, the size of the beakers in step 1.B should be adjusted to the volume of the samples to avoid spills and sample loss during step 2.

1.C. If soil samples are transferred to the beakers using any tool (e.g., spoon), be sure to prevent cross-contamination by cleaning the tool each time a new sample is processed (e.g., by rinsing it with 99% isopropyl alcohol).

1.C. Weighing the samples and containers in steps 1.C, 4.A, and 4.C is intended to calculate the concentration of charcoal in each sample by mass (i.e., mass of extracted charcoal divided by total mass of sample), but it can be skipped if that information is not needed.

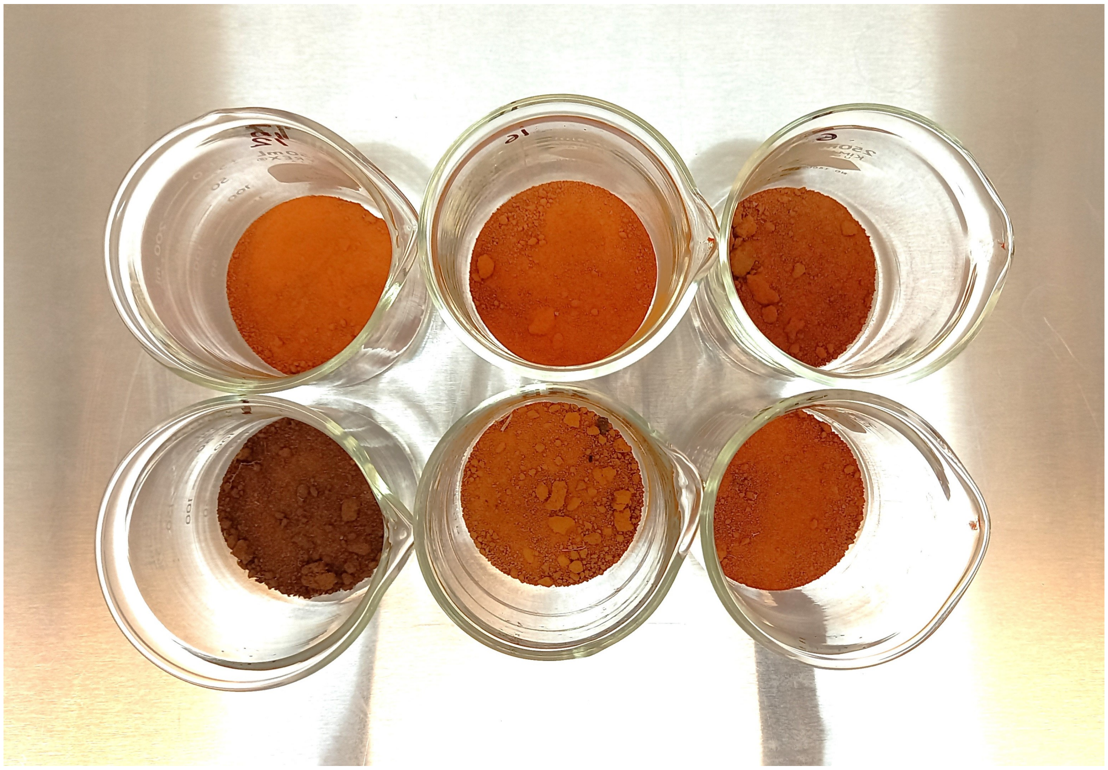

Dry soil samples (20 g each) in 200 mL glass beakers.

2 **Dispersion and digestion of soils**

2.A. To each beaker containing 20 g of soil, add 100 mL of a solution of 2.5% sodium hexametaphosphate ($Na_6P_6O_{18}$) and 5% hydrogen peroxide (H_2O_2)—prepared as a 1:1 mixture of 50 mL of 5% sodium hexametaphosphate and 50 mL of 10% hydrogen peroxide.

2.B. Place the beakers in an orbital shaker at 250-300 RPM and leave them reacting for 24 hours.

Note

2.A. If sample mass or volume has been modified in step 1.C, the solution of 2.5% sodium hexametaphosphate and 5% hydrogen peroxide to be added to each sample should follow a relationship of 5 mL per g or cm^3 of dry sample. Because organic-rich samples often react vigorously by oxidation, we suggest adding the solution in small quantities and monitor the reaction.

2.A. For studies interested in recovering charcoal produced at low temperatures (i.e., 250-400 ºC), oxidants like hydrogen peroxide should be avoided or milder concentrations and exposure times should be tested beforehand.

2.B. If charcoal fragments are particularly fragile, reduce the speed. An orbital shaker helps the dispersion of soils and the solution to act on charcoal surfaces by constant stirring, but it can be substituted by gentle manual shaking.

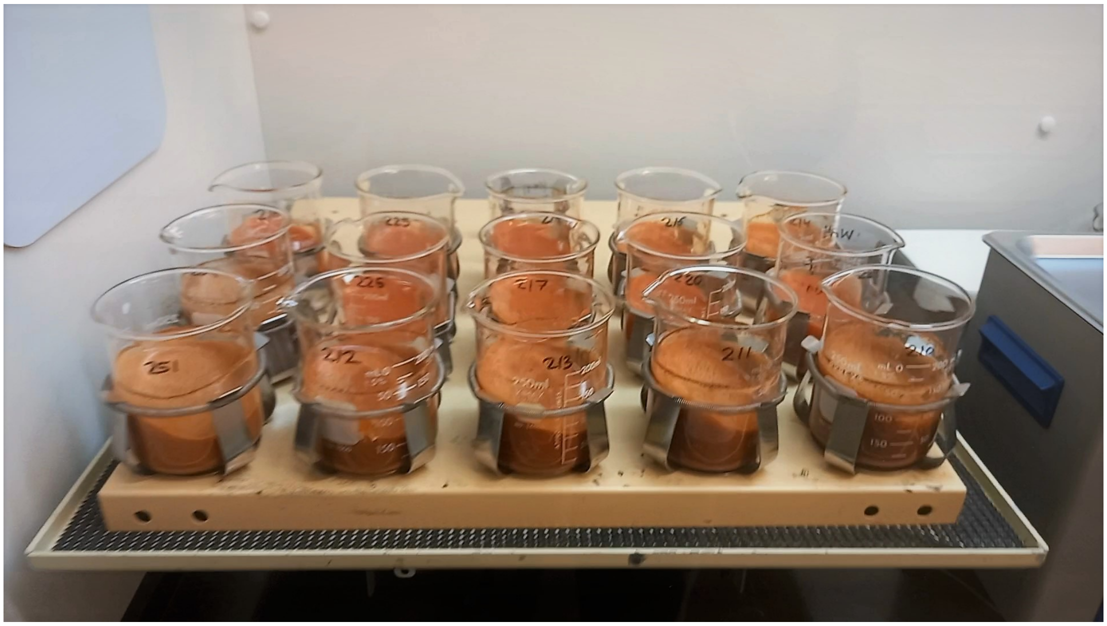

20 g soil samples in an orbital shaker reacting with 100 mL of a solution of 2.5% sodium hexametaphosphate ($Na_6P_6O_{18}$) and 5% hydrogen peroxide (H_2O_2).

3 **Sieving of samples**

3.A. Place a sieve with a 500-micron mesh over a container or sink and gently pour each sample (i.e., the soil sample with the solution) from the beaker into the sieve using a wash bottle with purified water.

3.B. Carefully rinse each sample with purified water, utilizing either a wash bottle or running water from a faucet, until only particles ≥500 microns are retained, separated from each other, and charcoal fragments are clean.

Note

3.A. To recover charcoal with sizes other than ≥500 microns, change the mesh size or add nested sieves of different mesh sizes (where top sieve is the largest size and bottom sieve the smallest).

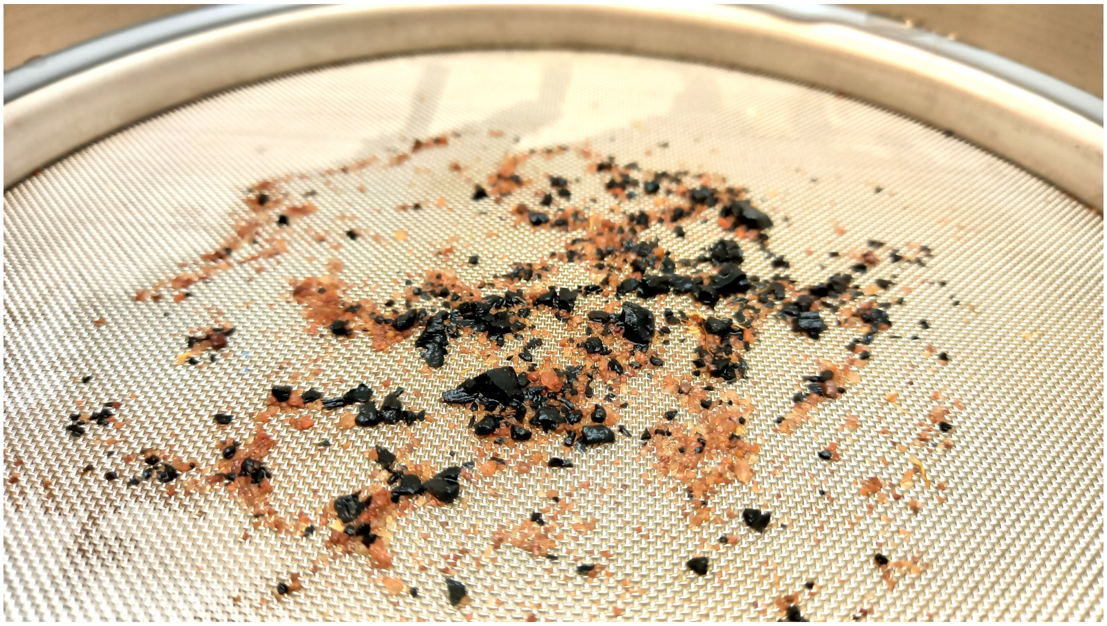

Charcoal fragments and sand grains captured in a 500-micron mesh sieve after being rinsed with purified water.

4 **Collection of macroscopic charcoal and storage**

4.A. Label sample containers (e.g., vials), weigh them, and record weight. Carefully collect the charcoal fragments from the sieve using tweezers and transfer them to the containers. Discard all non-charcoal material.

4.B. To remove moisture from the charcoal fragments, oven-dry the containers while open at 40 ºC.

4.C. Visually inspect the extracted material in the containers, with the naked eye or under magnification, to remove non-charcoal bodies (i.e., extraneous material, like plant parts or sand grains, may attach to the charcoal fragments or be mistaken for charcoal while the samples are wet in step 4.A). Weigh the containers with the extracted charcoal and record weight. Store the containers with their lids closed in a dry environment; the samples are ready for analysis.

Note

4.A. If the fragments are very fragile or if a sieve with openings smaller than 500 microns was used in step 3.A, we suggest using a wash bottle with purified water and a Chattaway spatula to carefully transfer the extract (i.e., charcoal fragments and soil particles) to the containers.

4.A, 4.C. If data on the concentration of charcoal by mass is not needed, weighing the containers and samples in steps 4.A and 4.C may be omitted.

4.B. Temperatures above 40 ºC should be avoided to prevent potential charcoal breakage.

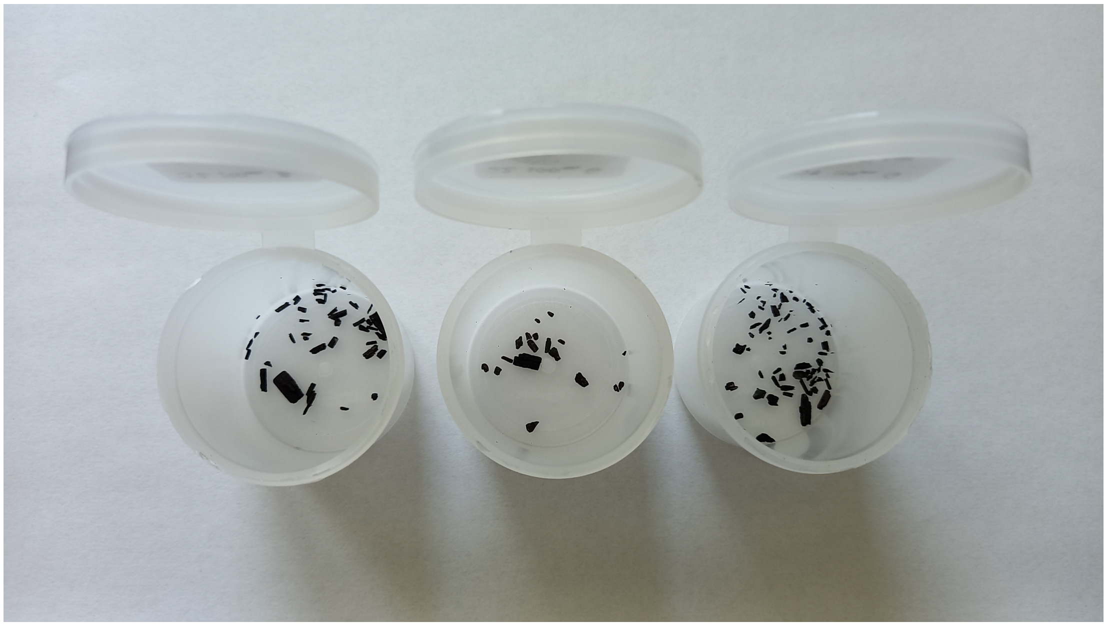

Charcoal samples in labelled plastic containers.

Charcoal counting and measurement

5 **Images acquisition**

5.A. For each sample, carefully place the extracted charcoal on a Petri dish (with the help of tweezers, a Chattaway spatula, or purified water, for example). To avoid dense clusters and overlapping fragments, add small amounts to the Petri dish, proceed to step 5.B, and repeat until all charcoal has been photographed. We recommend using a gridded Petri dish to facilitate image acquisition when the microscope field of view does not cover the entire Petri dish and multiple pictures per sample need to be taken.

5.B. Take pictures of the charcoal fragments under a light stereo microscope mounted with a digital camera. A magnification of 10-15x should be appropriate for charcoal fragments ≥500

µm. Images must have comparable settings (e.g., resolution, exposure, white balance) and same magnification. At least one image with a reference scale on it (e.g., scale bar) must be acquired to calculate the scale parameters in ImageJ in step 6.C.

5.C. Save all images in one folder and create another folder where to save the results to be obtained with ImageJ in step 6.C.

Note

5.A. Clean the Petri dish each time a new sample is processed (e.g., with 99% isopropyl alcohol) to prevent cross-contamination. To avoid possible bias in subsequent analyses, such as radiocarbon dating, allow the cleaning product to evaporate before adding charcoal to the Petri dish.

5.B. If the extraction contains only charcoal fragments, we recommend transmitted light to increase the contrast between the charcoal and the background. If the extraction contains other particles that were not removed during the extraction (e.g., sand grains), images should be captured in reflected light or in both reflected and transmitted light to make charcoal and other particles distinguishable from each other.

5.B, 5.C. Stereo microscope and camera software settings (e.g., brightness, contrast, white balance parameters) should be the same for all the images to minimize variation in color and light.

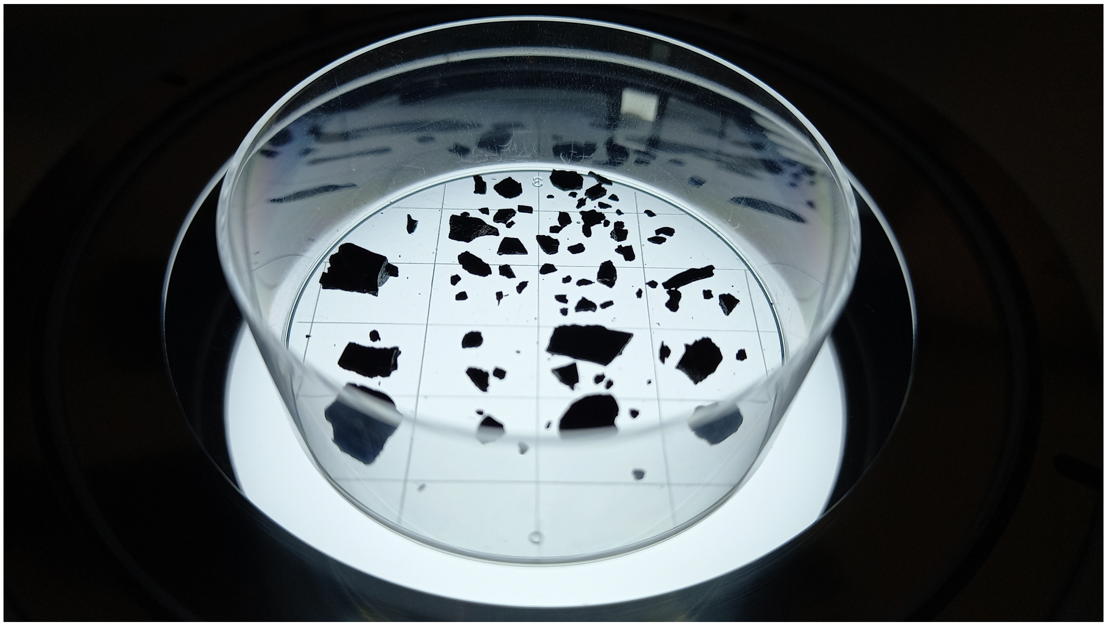

Charcoal fragments in a gridded Petri dish.

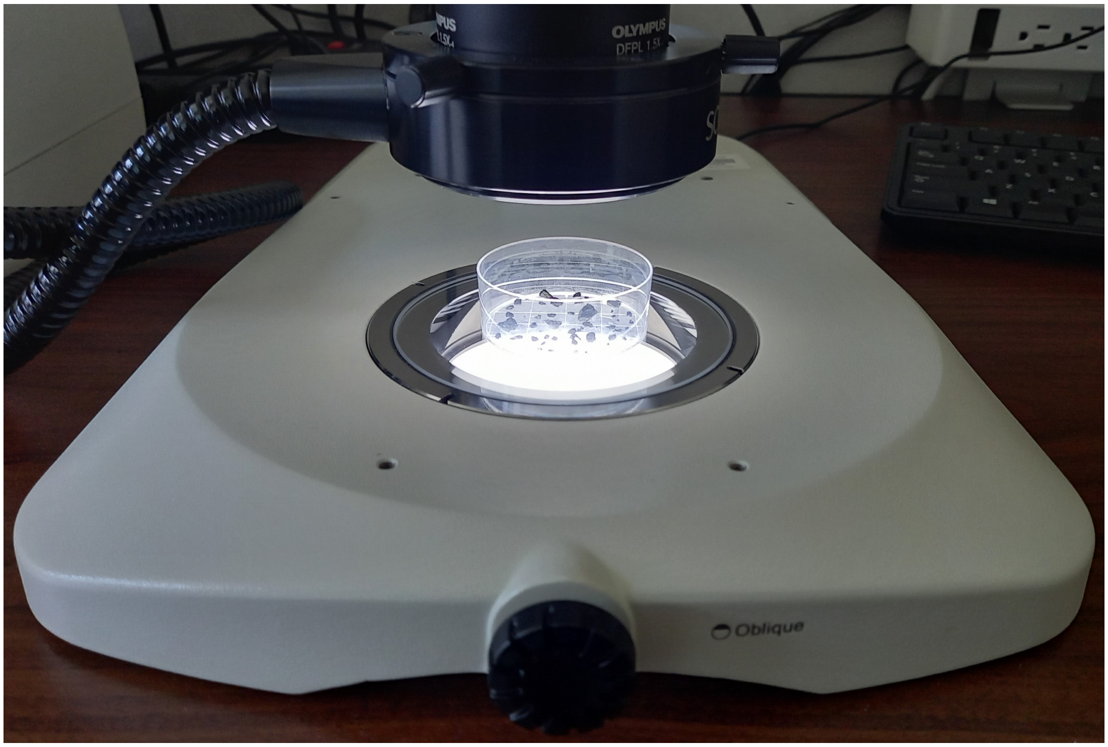

Petri dish containing charcoal fragments in transmitted light under a light stereo microscope mounted with a digital camera for image acquisition (camera not shown).

6 **Counting and measurement**

6.A. Download ImageJ from https://imagej.net/ij/download.html. ImageJ is an open source and multi-platform software that runs on Microsoft Windows, macOS, and Linux.

6.B. Copy the macro code from below and save it as a text file (i.e., with extension .txt). The macro allows the user to automatically isolate charcoal fragments from a group of images by means of global threshold segmentation (i.e., classification of pixels into two classes using a certain cut-off value), count and measure them (area, length, and width), and export the results. The output files consist of 1) a CSV file with the parameters set for analysis (see 6.C), date of analysis, and software versions; 2) a CSV file containing the counting and measurement data; and 3) outline image(s) in JPEG or TIFF format. The macro has been written and tested using ImageJ version 1.54d for Microsoft Windows with Java version 1.8.0_345.

6.C. Start ImageJ, go to *Plugins -> Macros -> Run...*, and open the macro saved in step 6.B. A parameters window will open and the following settings can be entered:

1. Folder containing the images to be analyzed and the destination folder to save the output files.
2. Scale unit of the images, known distance in original unit, and its equivalent in pixels. To obtain the scale values in ImageJ, 1) open an image with a reference scale such a scale bar (*File -> Open...*); 2) select the tool *Straight Line* (i.e., fifth tool from the left on the ImageJ

toolbar); 3) draw a line along the length of the scale bar while holding the Shift key in the keyboard; and 4) go to *Analyze -> Set Scale...* and record the distance in pixels and the known distance values.

3. Thresholding method and, if Manual method is selected, minimum and maximum pixel values (from 0 to 255) to discriminate the charcoal fragments against the background and non-charcoal particles. Automatic thresholding methods are described at https://imagej.net/plugins/auto-threshold#available-methods. Manual threshold values can be explored in ImageJ by 1) opening an image (*File -> Open...*); 2) transforming the image to grayscale (*Image -> Type -> 8-bit*); and 3) directly adjusting threshold values with the *Threshold* tool (*Image -> Adjust -> Threshold...*). Note that, when manual thresholding is selected, the macro applies the same minimum and maximum threshold values entered in the dialogue box to all images; in contrast, when an automatic threshold method is selected, it will calculate new threshold values for each image. To learn more about the threshold tool, visit https://imagej.net/ij/docs/guide/146-28.html#sub:Threshold...[T].
4. Size range (in the set unit, from 0 to Infinity) of the fragments to be counted and measured. Note that fragments touching the edges of the images are excluded from analysis.
5. Watershed segmentation to automatically separate fragments in contact (https://imagej.net/ij/docs/menus/process.html#watershed).
6. Holes filling to fill holes within charcoal.
7. Outline images format as JPEG or TIFF.

```
// 1. TITLE
// Macro to automatically detect, count, and measure charcoal
fragments from a batch of images in ImageJ

// 2. AUTHORS, DATE, AND LICENSE
// Javier Ruiz-Pérez (jruizperez@tamu.edu,
javier.ruizperez.academic@gmail.com) - Department of Ecology and
Conservation Biology, Texas A&M University; Julie C. Aleman -
Centre Européen de Recherche et d'Enseignement des Géosciences de
l'Environnement, Centre National de la Recherche Scientifique;
Joseph W. Veldman - Department of Ecology and Conservation
Biology, Texas A&M University
// 2024
// This macro is distributed under the terms of a CC BY 4.0
license

// 3. DESCRIPTION
// Macro to automatically detect, count, and measure (i.e., area,
length, and width) charcoal fragments in a set of images via
global thresholding (i.e., classification of pixels into two
classes using a cut-off value) and export the results. The output
files consist of 1) a CSV file with the parameters set for
analysis, date of analysis, and software versions; 2) a CSV file
containing the counting and measurement data; and 3) outline
image(s) in JPEG or TIFF format. This macro has been written and
tested using ImageJ version 1.54d for Microsoft Windows with Java
version 1.8.0_345. To use it, download ImageJ from
https://imagej.net/ij/download.html, open it, go to Plugins ->
Macros -> Run..., and open this macro as a .txt file

// 4. SCRIPT
// The script has two sections: definition of parameters (section
4.1) and batch analysis (section 4.2). The following parameters
are entered in a dialog box:
//      1) Folder containing the images to be analyzed (input) and
destination folder to save the output files (output)
//      2) Scale unit of the images (unit), known distance in
original unit (distanceUnit), and its equivalent in pixels
(distancePixels). To obtain the scale values in ImageJ, 1) open an
image with a reference scale such a scale bar (File -> Open...);
2) select the tool Straight Line (i.e., fifth tool from the left
on the ImageJ toolbar); 3) draw a line along the length of the
scale bar while holding the Shift key in the keyboard; and 4) go
```

```
to Analyze -> Set Scale... and record the distance in pixels and
the known distance values
//      3) Thresholding method (thresholdMethod) and, if Manual
method is selected, minimum and maximum pixel values
(thresholdMin, thresholdMax, respectively, from 0 to 255) to
discriminate the charcoal fragments against the background and
non-charcoal particles. Automatic thresholding methods are
described at https://imagej.net/plugins/auto-threshold#available-
methods. Manual threshold values can be explored in ImageJ by 1)
opening an image (File -> Open...); 2) transforming the image to
grayscale (Image -> Type -> 8-bit); and 3) directly adjusting
threshold values with the Threshold tool (Image -> Adjust ->
Threshold...). Note that, when manual thresholding is selected,
the macro applies the same minimum and maximum threshold values
entered in the dialogue box to all images; in contrast, when an
automatic threshold method is selected, it will calculate new
threshold values for each image. To learn more about the threshold
tool, visit https://imagej.net/ij/docs/guide/146-
28.html#sub:Threshold...[T]
//      4) Size range (in the set unit, from 0 to Infinity) of the
fragments to be counted and measured (sizeMin, sizeMax). Note that
fragments touching the edges of the images are excluded from
analysis
//      5) Watershed segmentation (separation) to automatically
separate fragments in contact
(https://imagej.net/ij/docs/menus/process.html#watershed)
//      6) Holes filling (holes) to fill holes within charcoal
//      7) Outline images format as JPEG or TIFF (format)

// 4.1. Parameters dialog
// List of parameters and default values
input = "Input folder";
output = "Output folder";
unit = newArray("micron", "mm", "cm");
distanceUnit = 1000;
distancePixels = 185.75;
thresholdAutoMethods = getList("threshold.methods");
thresholdMethod = Array.concat("Manual", thresholdAutoMethods);
thresholdMin = 0;
thresholdMax = 116;
sizeMin = 500;
sizeMax = "Infinity";
separation = newArray("No", "Yes");
holes = newArray("No", "Yes");
format = newArray("Jpeg", "Tiff");
```

```
// Parameters dialog box
Dialog.create("Select parameters");
Dialog.addDirectory("Folder containing the images to be
analyzed:", input);
Dialog.addDirectory("Folder where to save the results:", output);
Dialog.addChoice("Scale unit:", unit);
Dialog.addNumber("Scale distance in original unit:",
distanceUnit);
Dialog.addNumber("Scale distance in pixels:", distancePixels);
Dialog.addChoice("Thresholding method:", thresholdMethod);
Dialog.addNumber("Minimum manual threshold (0 to 255):",
thresholdMin);
Dialog.addNumber("Maximum manual threshold (0 to 255):",
thresholdMax);
Dialog.addNumber("Minimum charcoal size in set unit:", sizeMin);
Dialog.addNumber("Maximum charcoal size in set unit:", sizeMax);
Dialog.addChoice("Watershed separation:", separation);
Dialog.addChoice("Fill holes:", holes);
Dialog.addChoice("Outline image(s) format:", format);
Dialog.show();

// List of parameters, entered values, date of analysis, and
software versions
input = Dialog.getString();
output = Dialog.getString();
unit = Dialog.getChoice();
distanceUnit = Dialog.getNumber();
distancePixels = Dialog.getNumber();
thresholdMethod = Dialog.getChoice();
thresholdMin = Dialog.getNumber();
thresholdMax = Dialog.getNumber();
sizeMin = Dialog.getNumber();
sizeMin2 = Math.pow(sizeMin, 2);
sizeMax = Dialog.getNumber();
sizeMax2 = Math.pow(sizeMax, 2);
separation = Dialog.getChoice();
holes = Dialog.getChoice();
format = Dialog.getChoice();
monthNames = newArray("Jan", "Feb", "Mar", "Apr", "May", "Jun",
"Jul", "Aug", "Sep", "Oct", "Nov", "Dec");
getDateAndTime(year, month, week, day, hour, min, sec, msec);
dateTime = ""+day+"/"+monthNames[month]+"/"+year+"
"+hour+":"+min+":"+sec;
versionIJ = getVersion();
```

```
versionJava = getInfo("java.version");

// Export parameters, values, date, and versions list
Table.create("Parameters");
selectWindow("Parameters");
listParameters = Array.concat("Folder containing the charcoal
images", "Folder containing the results", "Scale unit", "Scale
distance in original unit", "Scale distance in pixels",
"Thresholding method", "Minimum manual threshold", "Maximum manual
threshold", "Minimum charcoal size", "Maximum charcoal size",
"Watershed separation", "Fill holes", "Outline image(s) format",
"Date and time", "ImageJ version", "Java version");
listParametersSelected = Array.concat(input, output, unit,
distanceUnit, distancePixels, thresholdMethod, thresholdMin,
thresholdMax, sizeMin, sizeMax, separation, holes, format,
dateTime, versionIJ, versionJava);
for (i = 0; i < 16; i++) {
        Table.set("Parameter", i, listParameters[i]);
        Table.set("Value", i, listParametersSelected[i])
}
Table.save(output + "Parameters" + ".csv");
run("Close");

// 4.2. Batch to process every image from the input folder and
export the results
// Names list from the images in the input folder
setBatchMode(true);
listNames = getFileList(input);
listNamesOutput = getFileList(input);
for (i = 0; i < listNamesOutput.length; i++) {
        dotIndex = indexOf(listNamesOutput[i], ".");
        listNamesOutput[i] = substring(listNamesOutput[i], 0,
dotIndex);
}

// Batch analysis
Table.create("Table of results");
for (i = 0; i < listNames.length; i++) {
        open(input + listNames[i]);
        run("8-bit");
        run("Set Scale...", "distance=distancePixels
known=distanceUnit unit=unit");
        if (thresholdMethod == "Manual") {
        setThreshold(thresholdMin, thresholdMax);
        setOption("BlackBackground", false);
```

```
		run("Convert to Mask");
		} else {
		setAutoThreshold(thresholdMethod);
		setOption("BlackBackground", false);
		run("Convert to Mask");
		}
		if (separation == "Yes"){
		run("Watershed");
		}
		if (holes == "Yes"){
		run("Fill Holes");
		}
		run("Set Measurements...", "area feret's redirect=None");
		run("Analyze Particles...", "size=sizeMin2-sizeMax2
show=Outlines display exclude clear");
		if (nResults > 0) {
			saveAs(format, output + listNamesOutput[i]);
			selectWindow("Results");
			count = nResults;
			area = Table.getColumn("Area");
			length = Table.getColumn("Feret");
			width = Table.getColumn("MinFeret");
			run("Close");
			selectWindow("Table of results");
			totalCount = Table.size;
			for (j = 0; j < count; j++) {
				Table.set("Image", totalCount+j,
listNamesOutput[i]);
				Table.set("Charcoal", totalCount+j, j+1);
				Table.set("Area", totalCount+j, area[j]);
				Table.set("Length", totalCount+j,
length[j]);
				Table.set("Width", totalCount+j,
width[j]);
			}
			Table.setLocationAndSize(100, 100, 750, 750);
			Table.update;
			close();
		} else {
			selectWindow("Table of results");
			totalCount = Table.size;
			Table.set("Image", totalCount,
listNamesOutput[i]);
			Table.set("Charcoal", totalCount, 0);
			Table.set("Area", totalCount, 0);
```

```
                Table.set("Length", totalCount, 0);
                Table.set("Width", totalCount, 0);
                Table.setLocationAndSize(100, 100, 750, 750);
                Table.update;
                close();
        }
}
Table.save(output + "Results" + ".csv");

// Message of completion/error
if (File.exists(output + "Results" + ".csv") == true) {
        showMessage("Analysis completed.");
} else {
        showMessage("An error has occurred.");
}
```

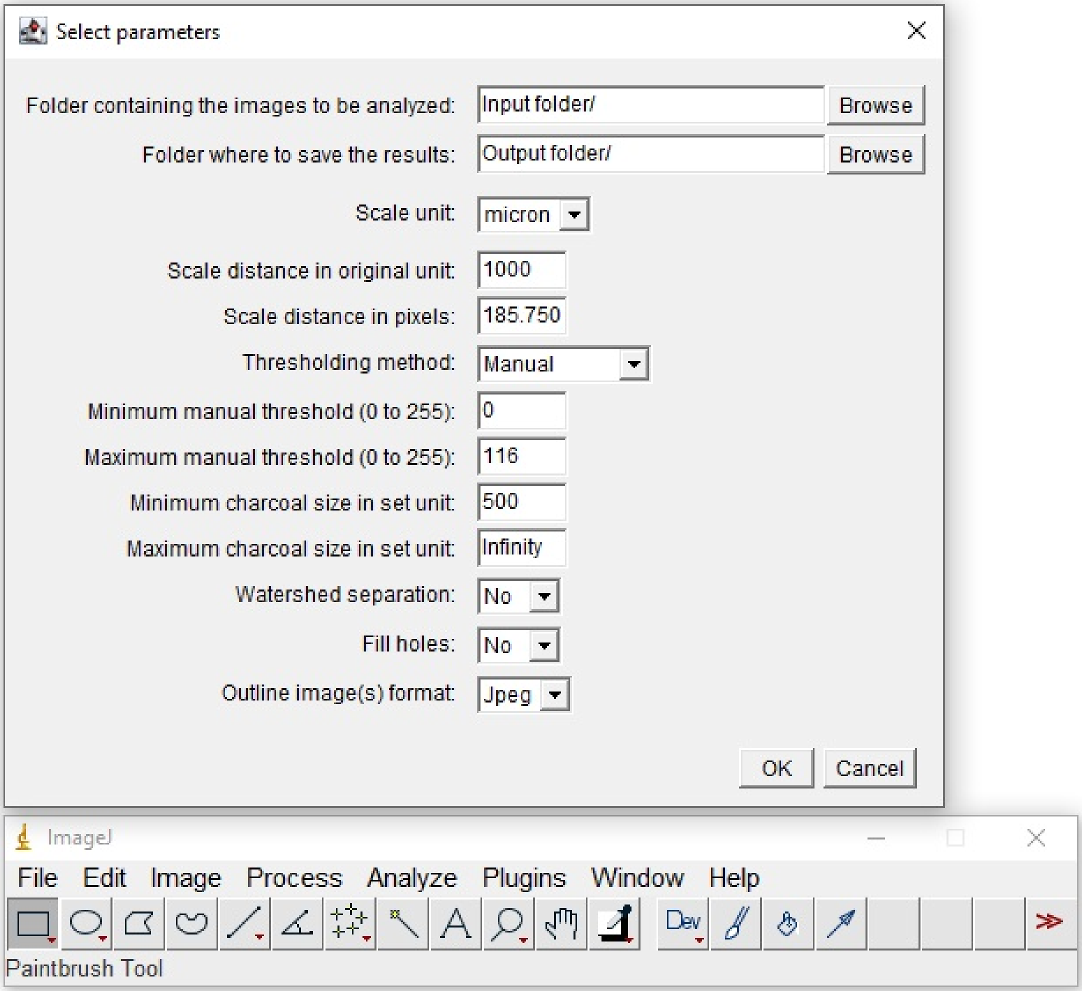

Parameters window in ImageJ after running the macro.

	A	B
1	**Parameter**	**Value**
2	Folder containing the charcoal images	C:\Users\javier.ruizperez\Documents\Images\
3	Folder containing the results	C:\Users\javier.ruizperez\Documents\Results\
4	Scale unit	micron
5	Scale distance in original unit	1000
6	Scale distance in pixels	185.75
7	Thresholding method	Manual
8	Minimum manual threshold	0
9	Maximum manual threshold	116
10	Minimum charcoal size	500
11	Maximum charcoal size	Infinity
12	Watershed separation	No
13	Fill holes	No
14	Outline image(s) format	Jpeg
15	Date and time	6/5/2023 10:02
16	ImageJ version	1.54d
17	Java version	1.8.0_345

Example of a table of parameters generated after running the analysis.

	A	B	C	D	E
1	**Image**	**Charcoal**	**Area**	**Length**	**Width**
2	J2_100F_1020_1	1	883457.809	1556.479	689.098
3	J2_100F_1020_1	2	319913.631	1128.461	403.073
4	J2_100F_1020_10	0	0	0	0
5	J2_100F_1020_2	1	1771234.08	2097.759	1316.721
6	J2_100F_1020_2	2	1611538.11	1717.37	1292.059
7	J2_100F_1020_3	1	1744627.74	2497.314	1158.621
8	J2_100F_1020_3	2	2295883.16	2100.59	1561.288
9	J2_100F_1020_3	3	2905742.06	2700.615	1477.212
10	J2_100F_1020_4	1	1270988.63	1723.957	976.995
11	J2_100F_1020_5	1	1153549.78	2255.823	681.014
12	J2_100F_1020_6	1	4066247.74	2663.311	2144.579
13	J2_100F_1020_7	1	6233243.79	5627.644	1405.321
14	J2_100F_1020_8	1	293278.314	866.271	633.819
15	J2_100F_1020_9	1	2044514.17	1928.569	1558.159

Example of a table of results generated after running the analysis.

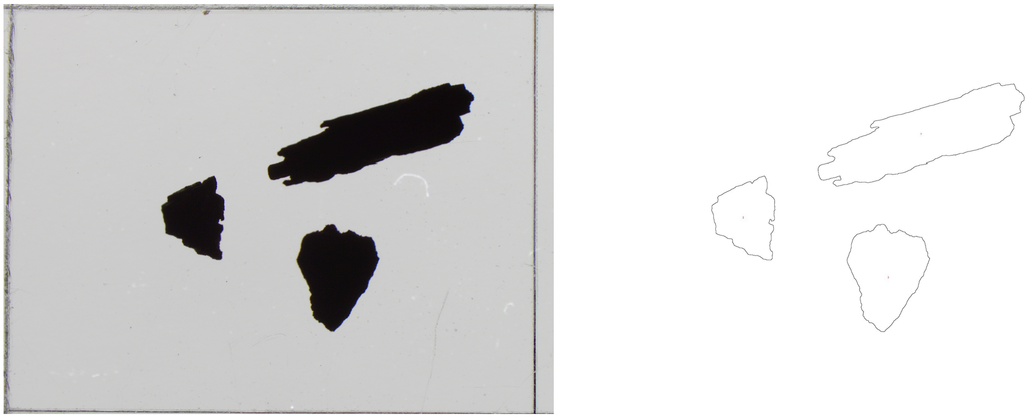

Example of charcoal fragments under the light stereo microscope in transmitted light at 12x magnification (left) and the resulting outline image after the analysis (right).
