# Supplementary material for: Reproducible protocol for the extraction and semi-automated quantification of macroscopic charcoal from soil
Source: PLoS One. 2024 Jul 12;19(7):e0304198. doi: 10.1371/journal.pone.0304198 (PMC11244820; doi:10.1371/journal.pone.0304198)
Supplement: S1 Appendix — (DOCX) [file pone.0304198.s002.docx]

**SUPPLEMENTARY APPENDIX 1**

This file describes the methodology that we used to assess the reporting practices of extraction and analysis methods of charcoal fragments from soil or sediment samples in 100 publications.

**Selection of publications**

We conducted a Google Scholar search on 8 March 2023 using Publish or Perish (version 8.8.4275 for Microsoft Windows) to obtain a list of 500 publications [1]. The following search parameters were used:

- Years: 0 - 0.
- Keywords: charcoal AND (fragment OR particle) AND fire AND (soil OR sediment).
- Maximum number of results: 500.
- Include: uncheck *CITATION records* and *Patents*.

We saved the results of the search and cleaned the dataset by removing publications without year of publication and keeping only the columns Year and DOI. We then randomly selected a subsample of 100 publications. In the resulting selection, we scanned and deleted unsuitable publications (i.e., not containing the extraction of charcoal fragments from soils/sediments), review and compendium texts, general books, reference collection articles, and studies analyzing charcoal only in pollen slides or petrographic thin sections. We randomly selected new publications and scanned them for suitability, and repeated this process until a list of 100 valid publications was completed.

**Assessment**

For each article we extracted the following variables and possible answers:

- Sample type: origin of soil/sediment samples.
  - Dry: dry environments including soil profile, terrestrial soil core, surface soil sample, surface soil trap, lithological profile, outcrop, archaeological feature.
  - Wet: wet conditions including (paleo)lake core, peat core, peat profile, pond core.
  - Both: study using samples from both dry and wet contexts.
- Extraction procedure: method for charcoal isolation from samples.
  - Sieving: mesh to separate fragments of a specific size from soil/sediment.
  - Heavy liquid: specific gravity solution to separate by flotation light from heavy particles.
  - Flotation: separation of charcoal from sample using water.
  - Hand: hand-picking/hand-sorting fragments directly from sample.
  - Several: combined use of different methods (e.g., flotation, sieving).
  - Not reported: extraction procedure not described or referenced.
- Extraction description: higher level of description of extraction protocol.
  - Specific protocol: complete step-by-step description of extraction protocol included in article or as a reference.
  - Summary: broad explanation without a step-by-step description and with low level of detail.
  - Generic protocol article: reference to a publication (protocol or research study) that does not contain a step-by-step description of extraction procedure.
  - Not reported: extraction procedure not described or referenced.
- Sample: description of soil/sediment samples.
  - Yes: description of soil/sediment mass/volume and treatment before charcoal extraction (i.e., drying including temperature, or wet).
  - No: incomplete description of soil/sediment sample.
  - NA: not applicable when study does not include soil/sediment samples (e.g., hand-picking charcoal fragments from archaeological features), extraction protocol is not reported or only a reference to another publication is provided.
- Chemicals: description of chemicals used during extraction.
  - Yes: description of chemicals including name, concentration, mass/volume added and reaction time.
  - No: incomplete description of chemicals.
  - NA: not applicable when extraction procedure does not involve using chemicals, extraction protocol is not reported or only a reference to another publication is provided.
- Extraction: description of isolation method of charcoal from samples.
  - Yes: description of sieving (i.e., mesh size, dry or wet sieving, liquid used for wet sieving), heavy liquid (i.e., chemical name, specific gravity, volume added), flotation (i.e., use of mesh or not and its size) or hand-picking/hand-sorting (i.e., details on tools and magnification if employed).
  - No: incomplete description of isolation method.
  - NA: not applicable when extraction protocol is not reported or only a reference to another publication is provided.
- Analysis procedure: method for charcoal analysis.
  - Manual: counting, measurement and/or identification analyses performed without using digital image analysis tools.
  - Software-assisted: use of image analysis software to count, measure and/or identify charcoal fragments.
  - Not reported: analysis procedure not described or referenced.
  - NA: not applicable when analysis is not intended to count, measure or identify charcoal fragments (e.g., radiocarbon dating).
- Analysis description: higher level of description of analysis protocol.
  - Specific protocol: complete step-by-step description of analysis protocol included in article or as a reference.
  - Summary: broad explanation without a step-by-step description and with low level of detail.
  - Generic protocol article: reference to a publication (protocol or research study) that does not contain a step-by-step description of analysis procedure.
  - Not reported: analysis procedure not described or referenced.
  - NA: not applicable when analysis is not intended to calculate mass, count, measure or identify charcoal fragments.
- Analysis: description of analysis method.
  - Yes: description of procedure to calculate mass/volume (i.e., equipment used), count (i.e., manual or software-assisted including magnification, use of gridded Petri dish/slide, software name and version, and script), measure (i.e., manual or software-assisted including magnification, use of gridded Petri dish/slide, software name and version, and script) and/or identify charcoal fragments (i.e., manual or software-assisted including magnification, software name and version, and script).
  - No: incomplete description of analysis procedure.
  - NA: not applicable when analysis is not intended to calculate mass, count, measure or identify charcoal fragments, or when extraction protocol is not reported or only a reference to another publication is provided.

The results of the assessment are in File S1. Metadata S1 contains metadata (i.e., description of variables and answers) associated with File S1.

**References**

1. Harzing AW. Publish or Perish. Version 8.8.4275 [software]. 2007 [cited 2023 Jul 6]. Available from: https://harzing.com/resources/publish-or-perish
