# Supplementary material for: Reproducible protocol for the extraction and semi-automated quantification of macroscopic charcoal from soil
Source: PLoS One. 2024 Jul 12;19(7):e0304198. doi: 10.1371/journal.pone.0304198 (PMC11244820; doi:10.1371/journal.pone.0304198)
Supplement: S2 Appendix — (DOCX) [file pone.0304198.s003.docx]

**SUPPLEMENTARY APPENDIX 2**

This file provides information on the soil samples that we used to test and validate the protocol.

**Soil samples**

In May-June 2022, we collected 339 soil samples from 18 ~2-m deep cores along each of three 500-m long transects (six cores per transect) spanning forest-savanna boundaries in the region of Chiquitania in eastern lowland Bolivia (Table 1 in S2 Appendix). The region supports mosaics of forests and savannas with seasonally dry tropical forest being the most widespread ecosystem. Our transects extended 100 m into the forest and 400 m into fire-frequented bamboo savannas dominated by *Guadua paniculata* Munro (Bambusoideae, Poaceae) locally known as *guapasales* [1]. At the endpoints of each transect and intermediate points spaced 100 m apart, we removed litter and duff (O horizon) to expose mineral soil. We then used a 9-cm diameter bucket auger (Forestry Suppliers 67190) to extract soils in 10-cm increments to a depth of 2 m (or until we reached bedrock). We collected each 10-cm increment sample in plastic bags in the field and shipped 60-g sub-samples to the Laboratory of Fire Ecology and Savanna Conservation at Texas A&M University for charcoal extraction and quantification. Transects were all on well-drained, acidic, upland soils, with mostly silty and loamy textures (Table 1 in S2 Appendix). Organic matter content is generally high in the surface soil samples (0-20 cm depth), ranging from 2.0% to 4.3% (median = 2.9%), and low in the subsoil samples (90-110 cm depth), ranging from 1.0% to 2.4% (median = 1.4%) (soil organic matter analysis conducted by Woods End Laboratories LLC, Augusta, Maine, USA).

Table 1. Description of the soil samples used in this study.

| **Transect** | **Section** | **Transect distance (m)** | **Latitude** | **Longitude** | **Core depth (m)** | **10-cm samples collected** | **Example of sample ID code in supporting information files*** | **Surface soil texture**  **(0-20 cm)**** | **Subsoil texture (90-110 cm)**** |
| --- | --- | --- | --- | --- | --- | --- | --- | --- | --- |
| J2 | Forest | 100 | -16.3951 | -61.18863 | 2 | 20 | J2_100F_1020 | Silt loam | Silt clay loam |
|  | Edge | 0 | -16.39422 | -61.18881 | 2 | 20 | J2_0E_1020 | Silt loam | Loam |
|  | Savanna | 100 | -16.39336 | -61.18898 | 2 | 20 | J2_100S_1020 | Silt | Silt loam |
|  |  | 200 | -16.39249 | -61.18929 | 2 | 20 | J2_200S_1020 | Silt loam | Loam |
|  |  | 300 | -16.39162 | -61.18953 | 2 | 20 | J2_300S_1020 | Silt | Loam |
|  |  | 400 | -16.39074 | -61.18971 | 2 | 20 | J2_400S_1020 | Silt loam | Clay loam |
| J7 | Forest | 100 | -16.40558 | -61.15384 | 1.3 | 13 | J7_100F_1020 | Silt loam | Loam |
|  | Edge | 0 | -16.40579 | -61.15476 | 1.4 | 14 | J7_0E_1020 | Silt loam | Silt loam |
|  | Savanna | 100 | -16.40605 | -61.15564 | 2 | 20 | J7_100S_1020 | Silt loam | Loam |
|  |  | 200 | -16.40624 | -61.15656 | 2 | 20 | J7_200S_1020 | Silt loam | Loam |
|  |  | 300 | -16.4065 | -61.15746 | 2 | 20 | J7_300S_1020 | Silt loam | Clay loam |
|  |  | 400 | -16.40664 | -61.1584 | 2 | 20 | J7_400S_1020 | Sandy loam | Silt loam |
| J8 | Forest | 100 | -16.37917 | -61.22145 | 2 | 20 | J8_100F_1020 | Sandy loam | Sandy clay loam |
|  | Edge | 0 | -16.3791 | -61.22237 | 2 | 20 | J8_0E_1020 | Silt | Clay loam |
|  | Savanna | 100 | -16.379 | -61.22331 | 1.8 | 18 | J8_100S_1020 | Loam | Clay loam |
|  |  | 200 | -16.37895 | -61.22425 | 1.4 | 14 | J8_200S_1020 | Sandy loam | Loam |
|  |  | 300 | -16.37883 | -61.2252 | 2 | 20 | J8_300S_1020 | Silt loam | Loam |
|  |  | 400 | -16.37877 | -61.22613 | 2 | 20 | J8_400S_1020 | Silt loam | Loam |

* Each part, separated by underscores, refers to the transect, the distance (m) and section in transect, and the depth interval (cm), respectively.

** Texture analysis, which we provide for descriptive purposes only, was performed by Woods End Laboratories LLC, Augusta, Maine, USA.

**References**

1. Killeen TJ, Louman BT, Grimwood T. La ecología paisajística de la región de Concepción y Lomerío en la Província de Ñuflo de Chávez, Santa Cruz, Bolivia. Ecología en Bolivia. 1990;16: 1–45.
