# Supplementary figures and images for: Reproducible protocol for the extraction and semi-automated quantification of macroscopic charcoal from soil (part 2 of 2)
Source: PLoS One. 2024 Jul 12;19(7):e0304198. doi: 10.1371/journal.pone.0304198 (PMC11244820; doi:10.1371/journal.pone.0304198)

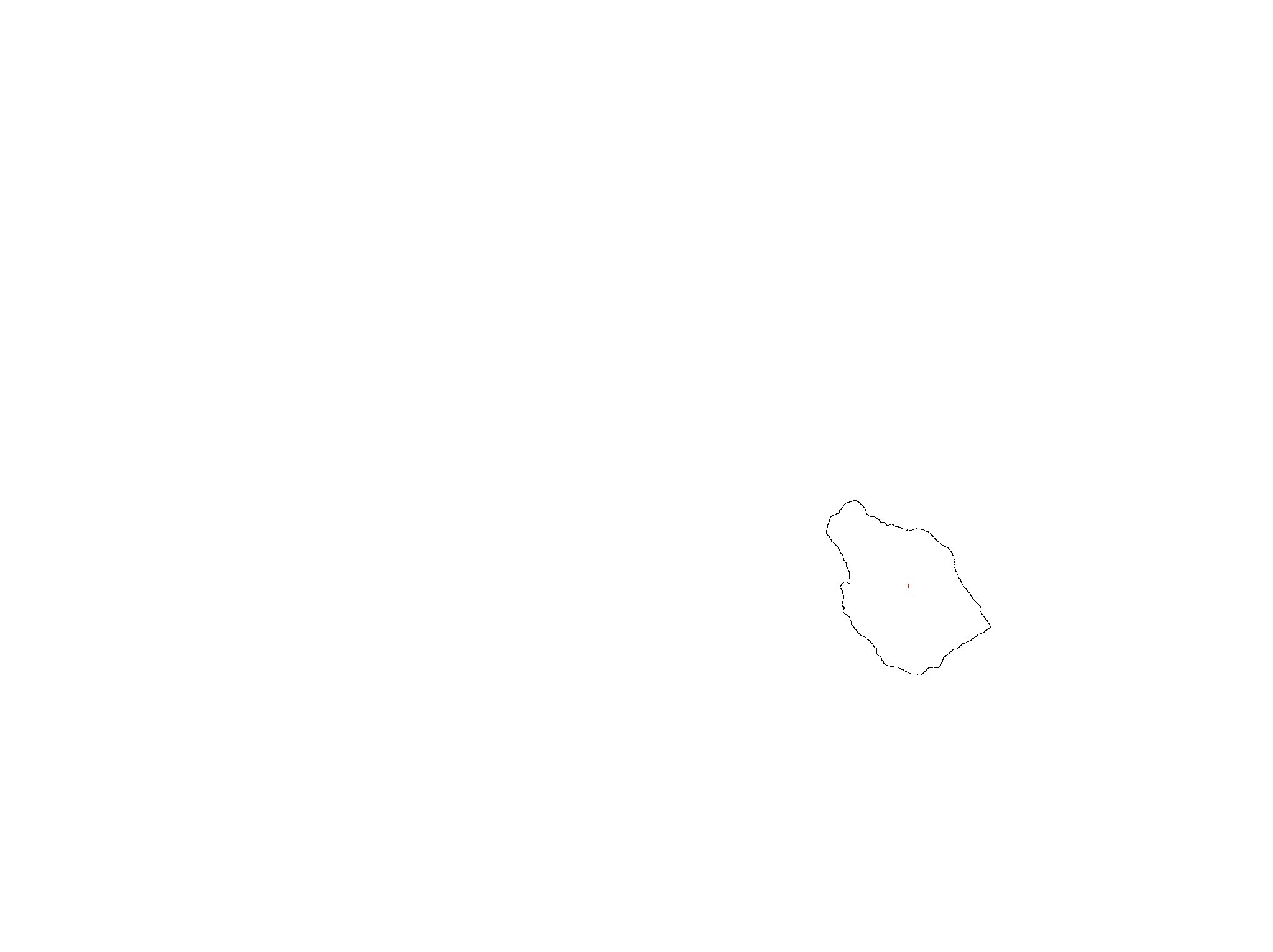

Supplement: S2 Dataset — (ZIP) [file pone.0304198.s005.zip › S2_Dataset_Raw_results_ImageJ/J2_200S_3040_8.jpg]

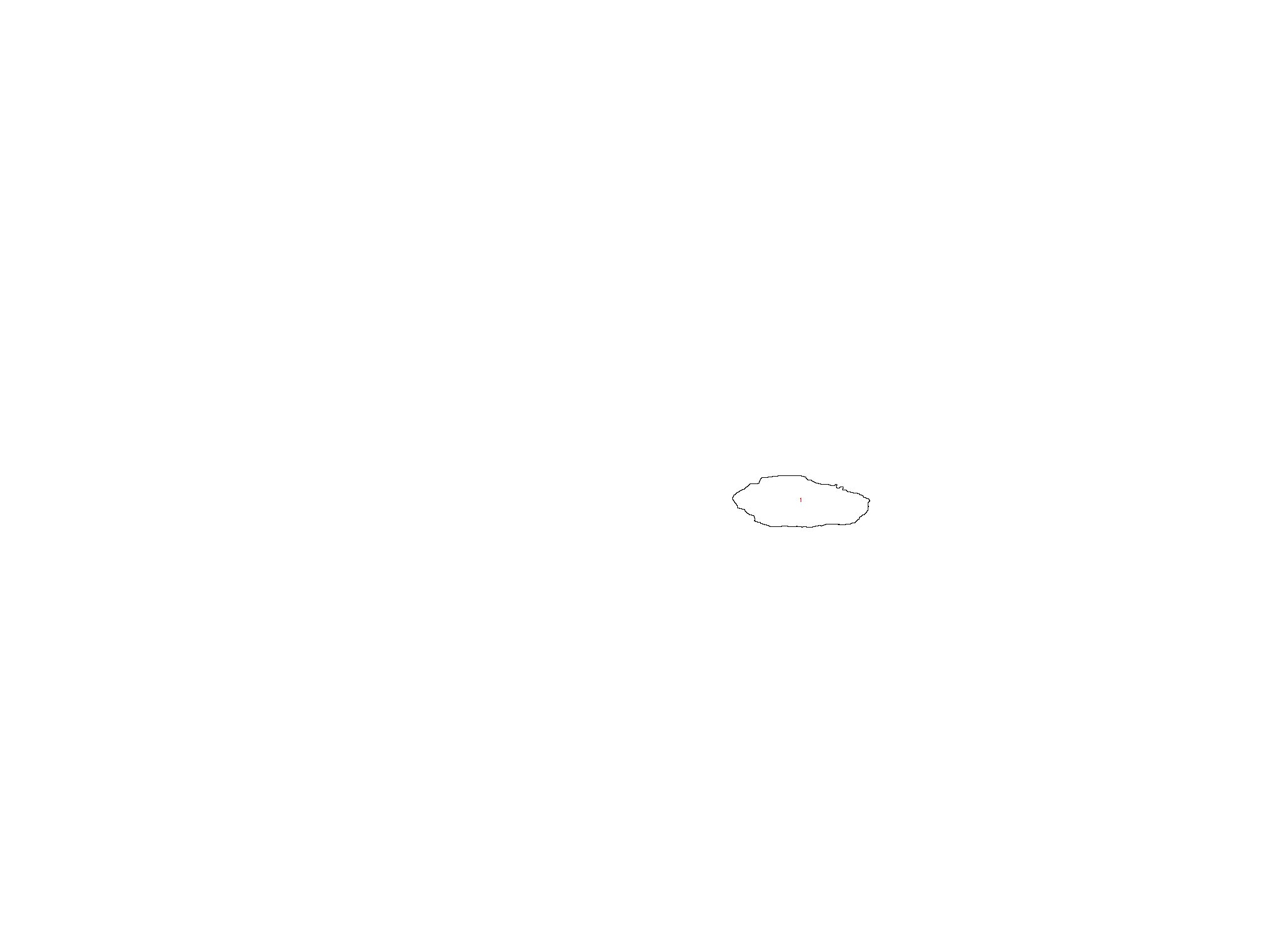

Supplement: S2 Dataset — (ZIP) [file pone.0304198.s005.zip › S2_Dataset_Raw_results_ImageJ/J2_200S_3040_9.jpg]

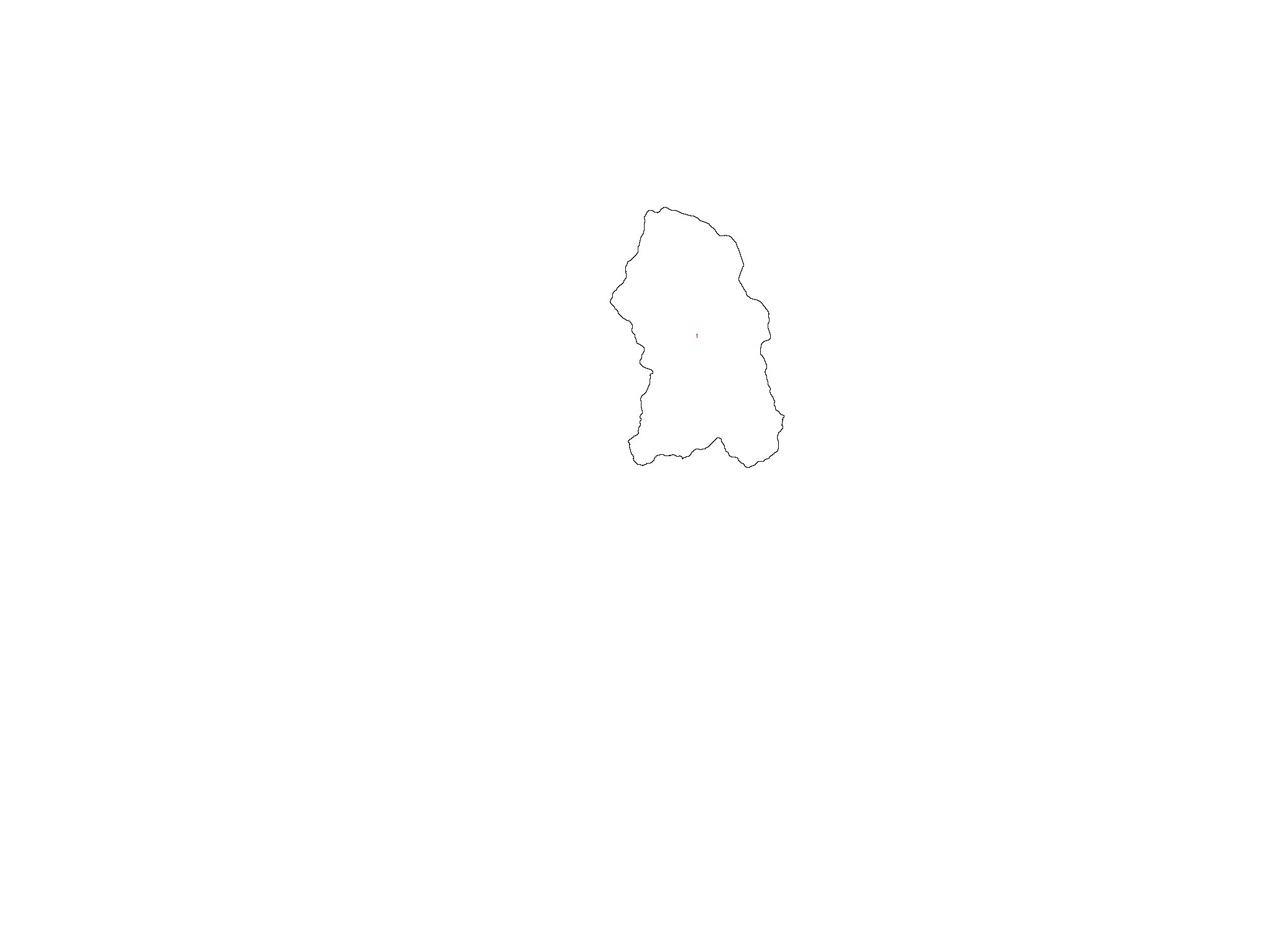

Supplement: S2 Dataset — (ZIP) [file pone.0304198.s005.zip › S2_Dataset_Raw_results_ImageJ/J2_200S_7080_1.jpg]

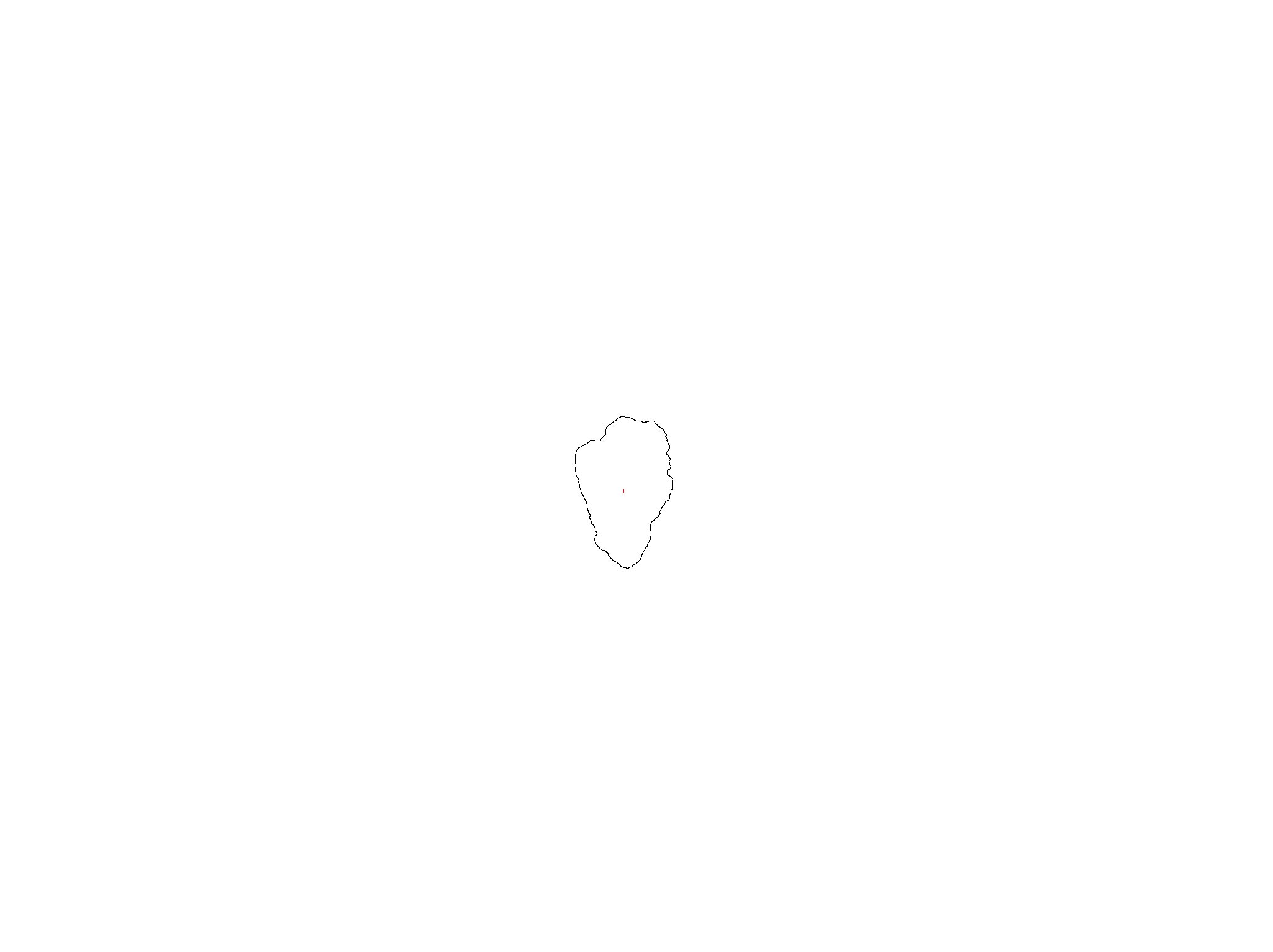

Supplement: S2 Dataset — (ZIP) [file pone.0304198.s005.zip › S2_Dataset_Raw_results_ImageJ/J2_200S_7080_2.jpg]

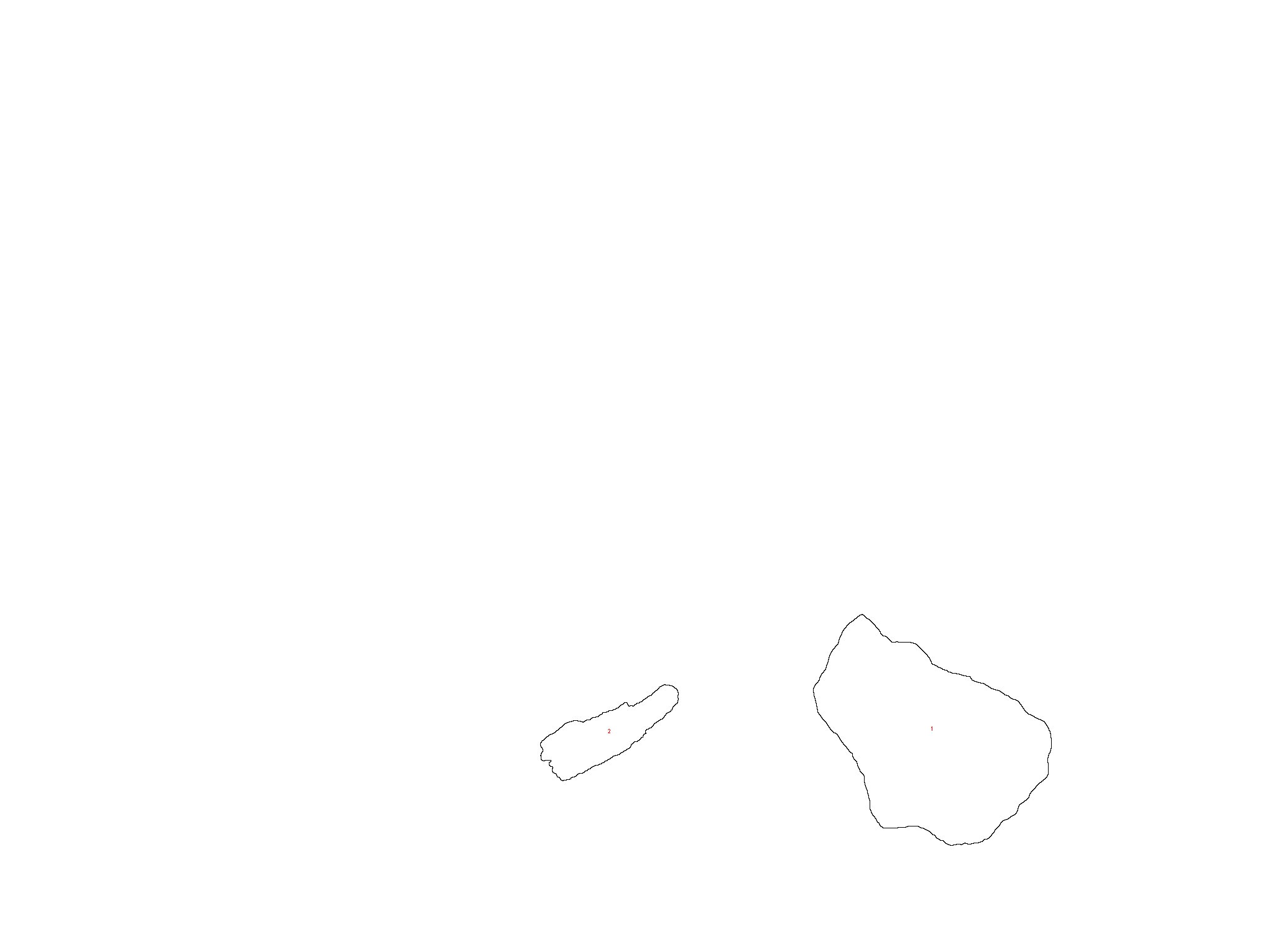

Supplement: S2 Dataset — (ZIP) [file pone.0304198.s005.zip › S2_Dataset_Raw_results_ImageJ/J2_200S_8090_1.jpg]

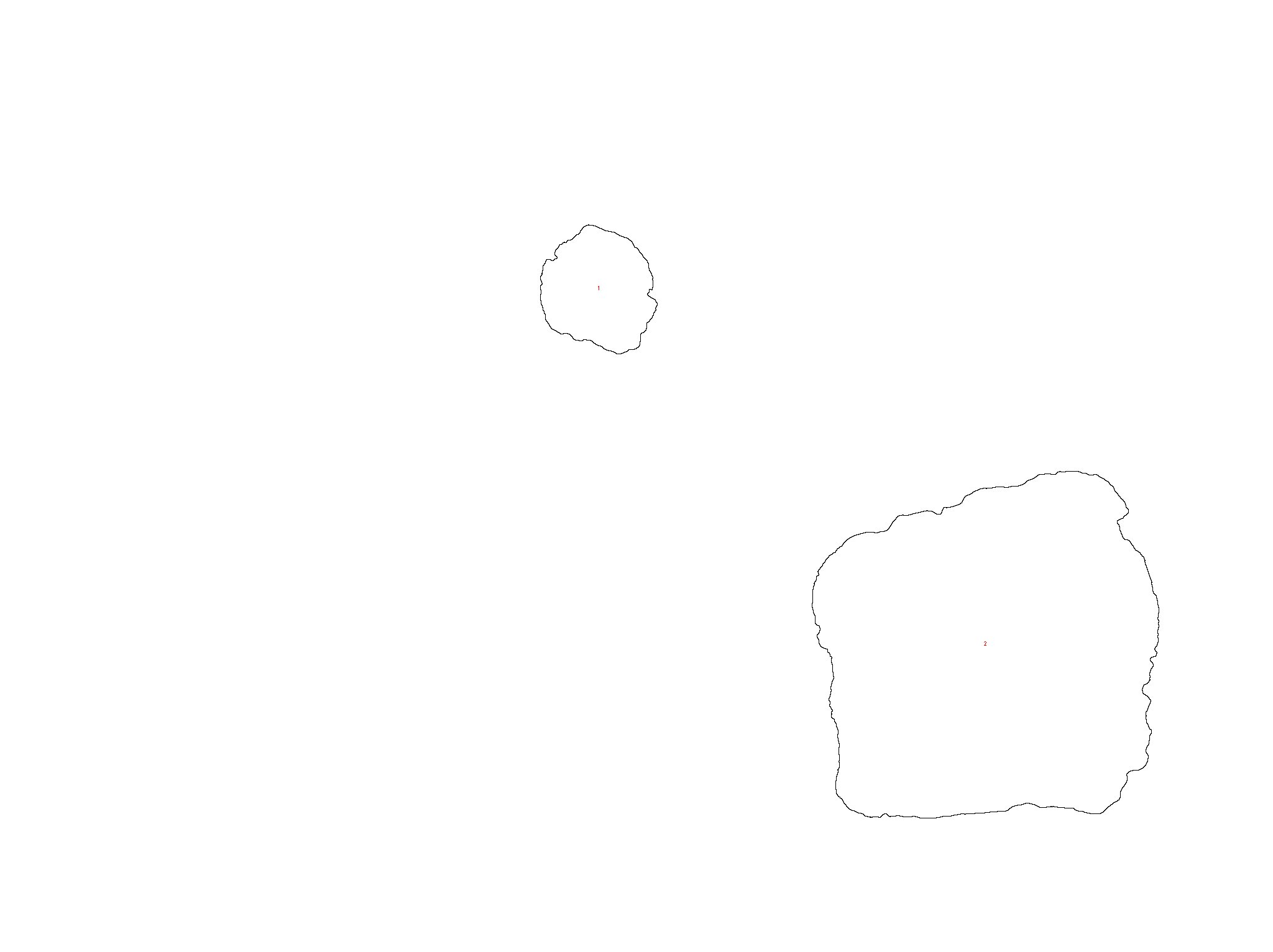

Supplement: S2 Dataset — (ZIP) [file pone.0304198.s005.zip › S2_Dataset_Raw_results_ImageJ/J2_200S_8090_2.jpg]

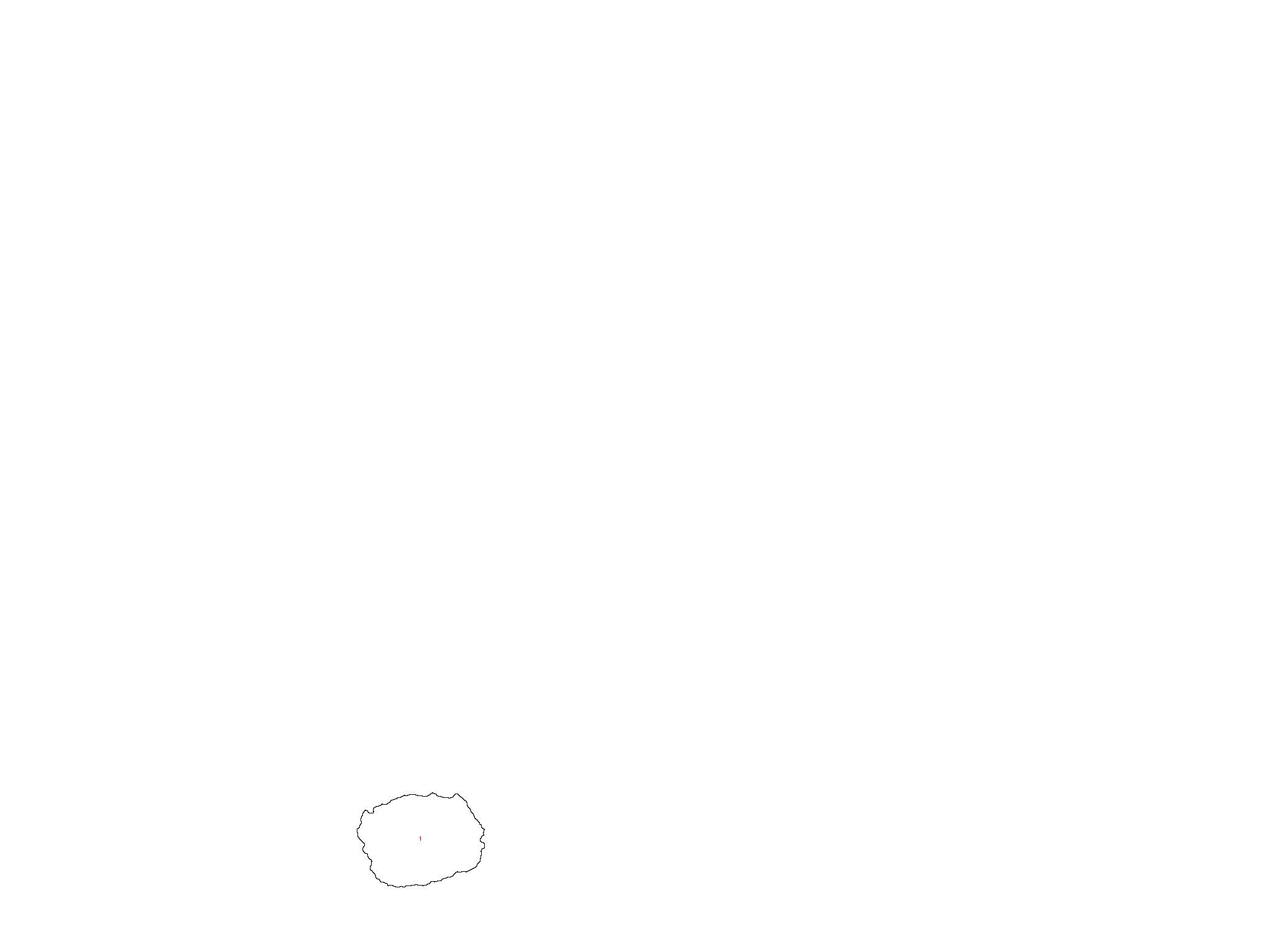

Supplement: S2 Dataset — (ZIP) [file pone.0304198.s005.zip › S2_Dataset_Raw_results_ImageJ/J2_200S_8090_3.jpg]

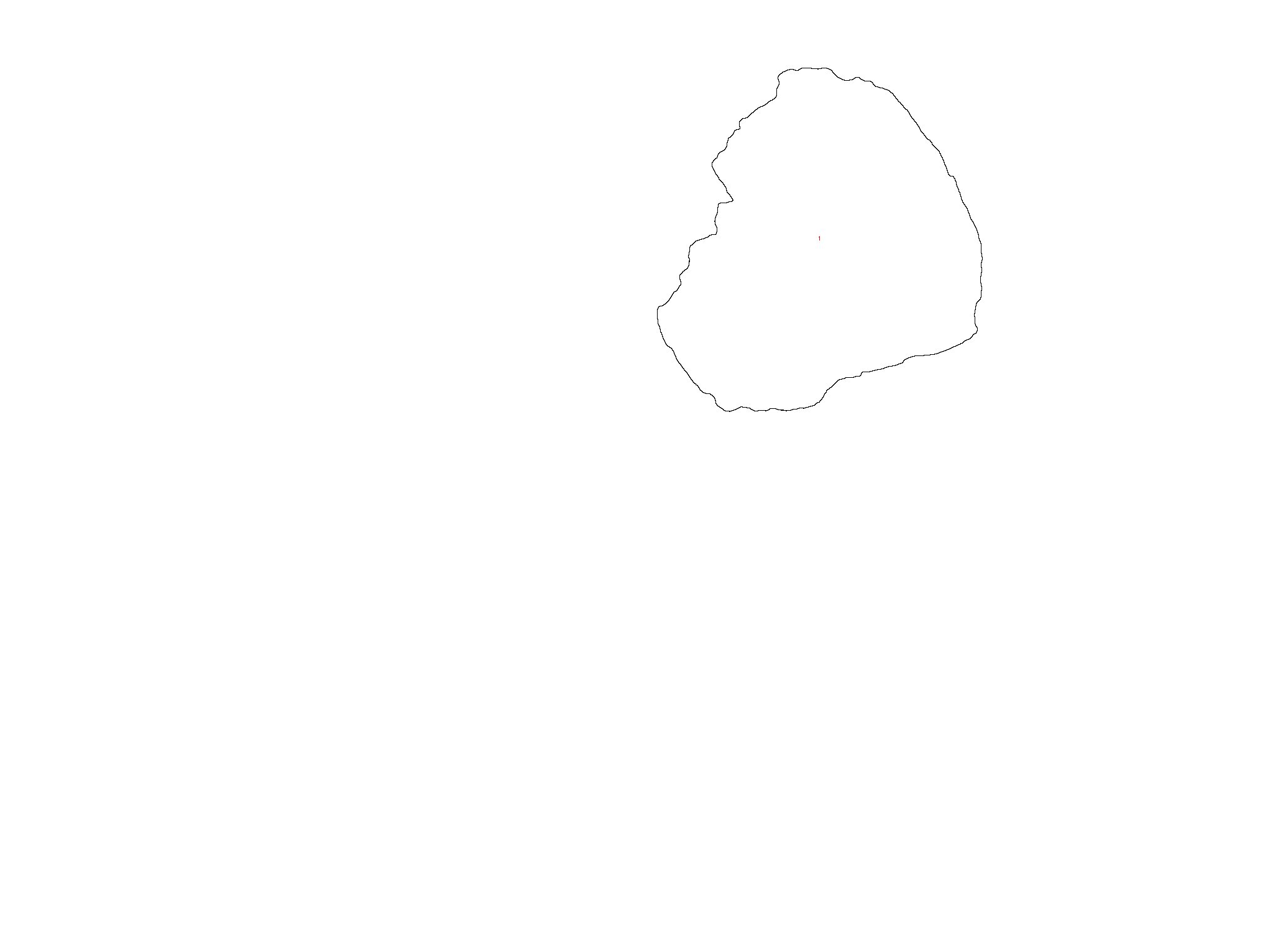

Supplement: S2 Dataset — (ZIP) [file pone.0304198.s005.zip › S2_Dataset_Raw_results_ImageJ/J2_200S_8090_5.jpg]

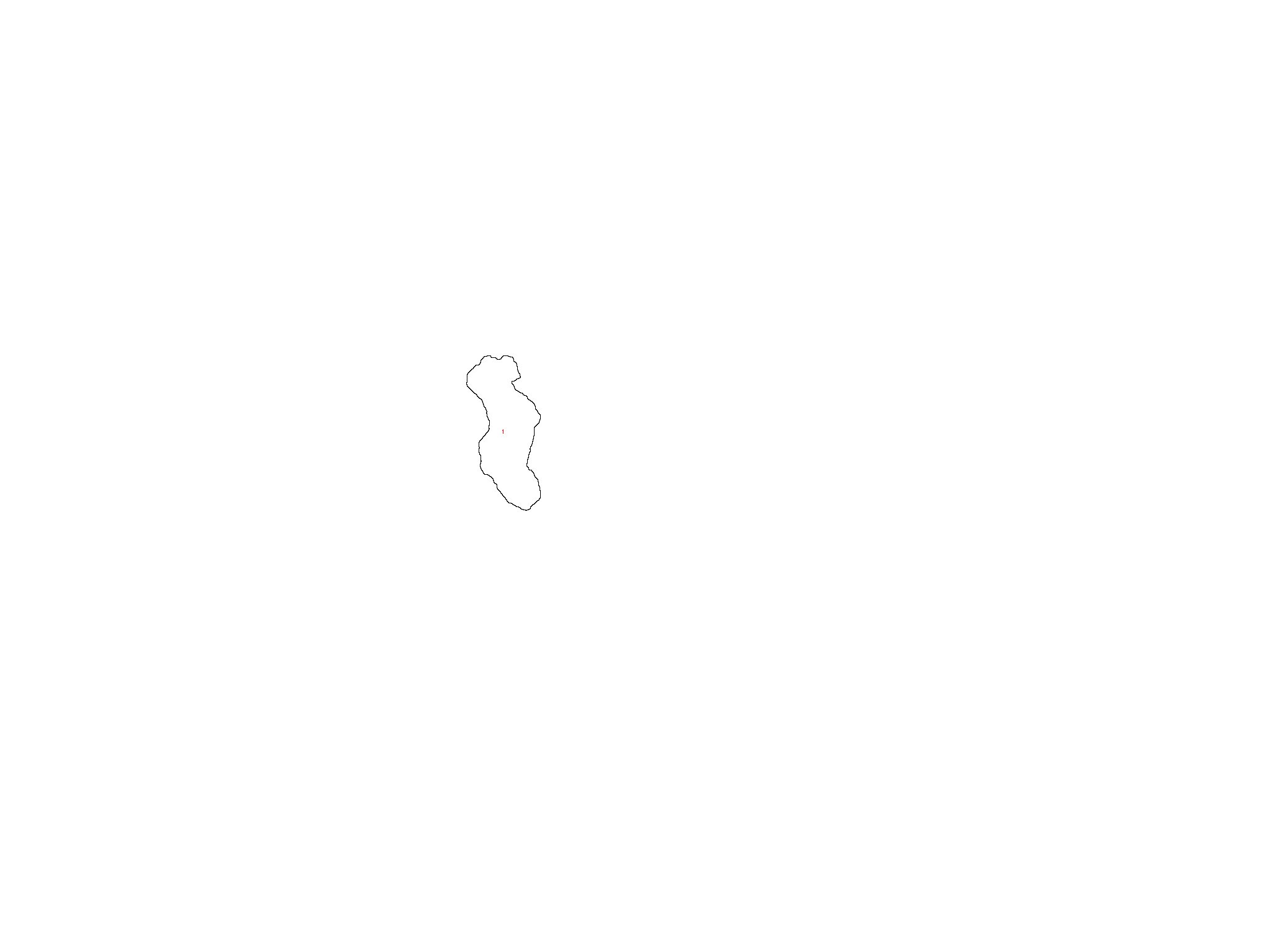

Supplement: S2 Dataset — (ZIP) [file pone.0304198.s005.zip › S2_Dataset_Raw_results_ImageJ/J2_200S_8090_6.jpg]

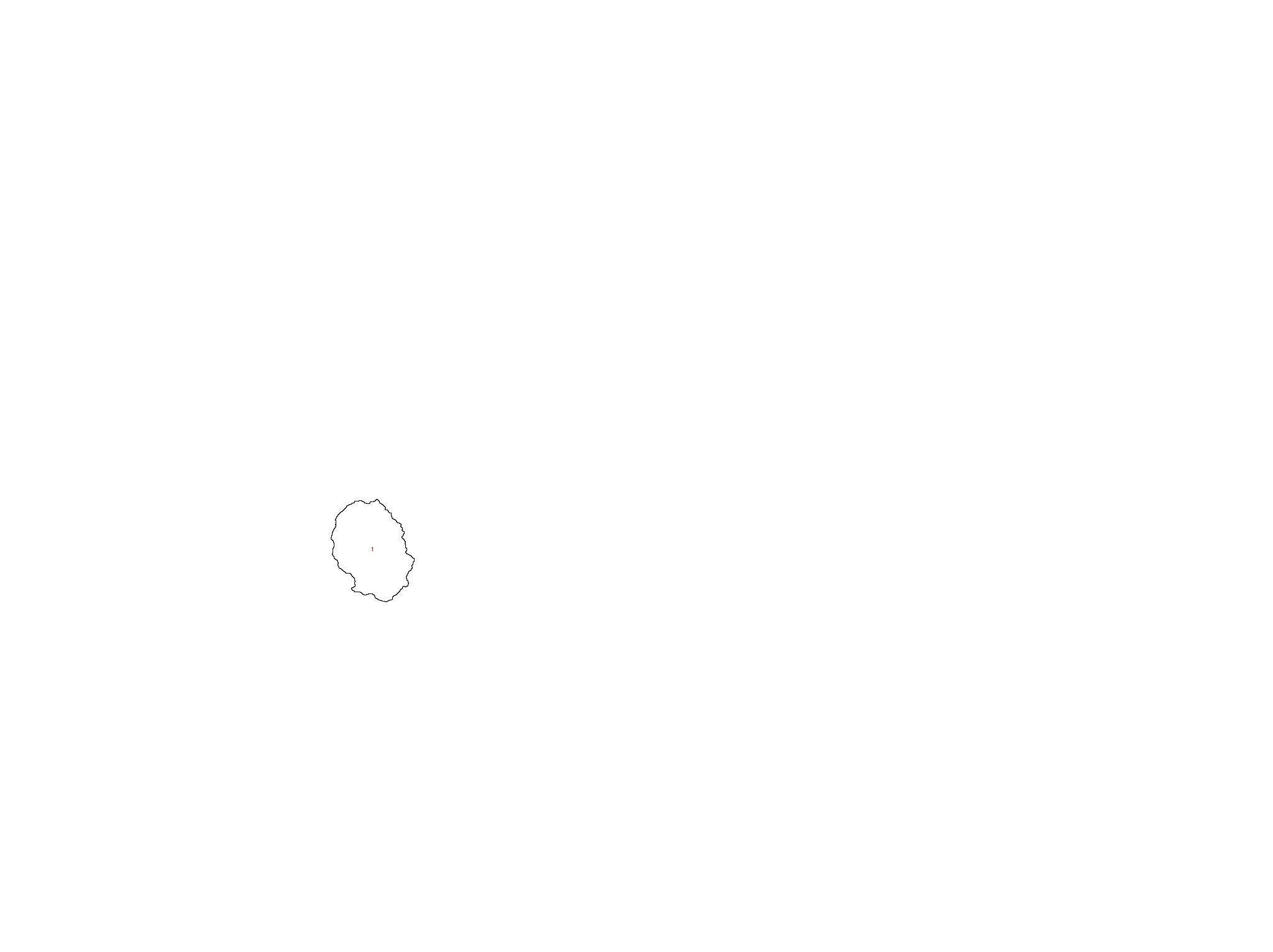

Supplement: S2 Dataset — (ZIP) [file pone.0304198.s005.zip › S2_Dataset_Raw_results_ImageJ/J2_200S_90100_1.jpg]

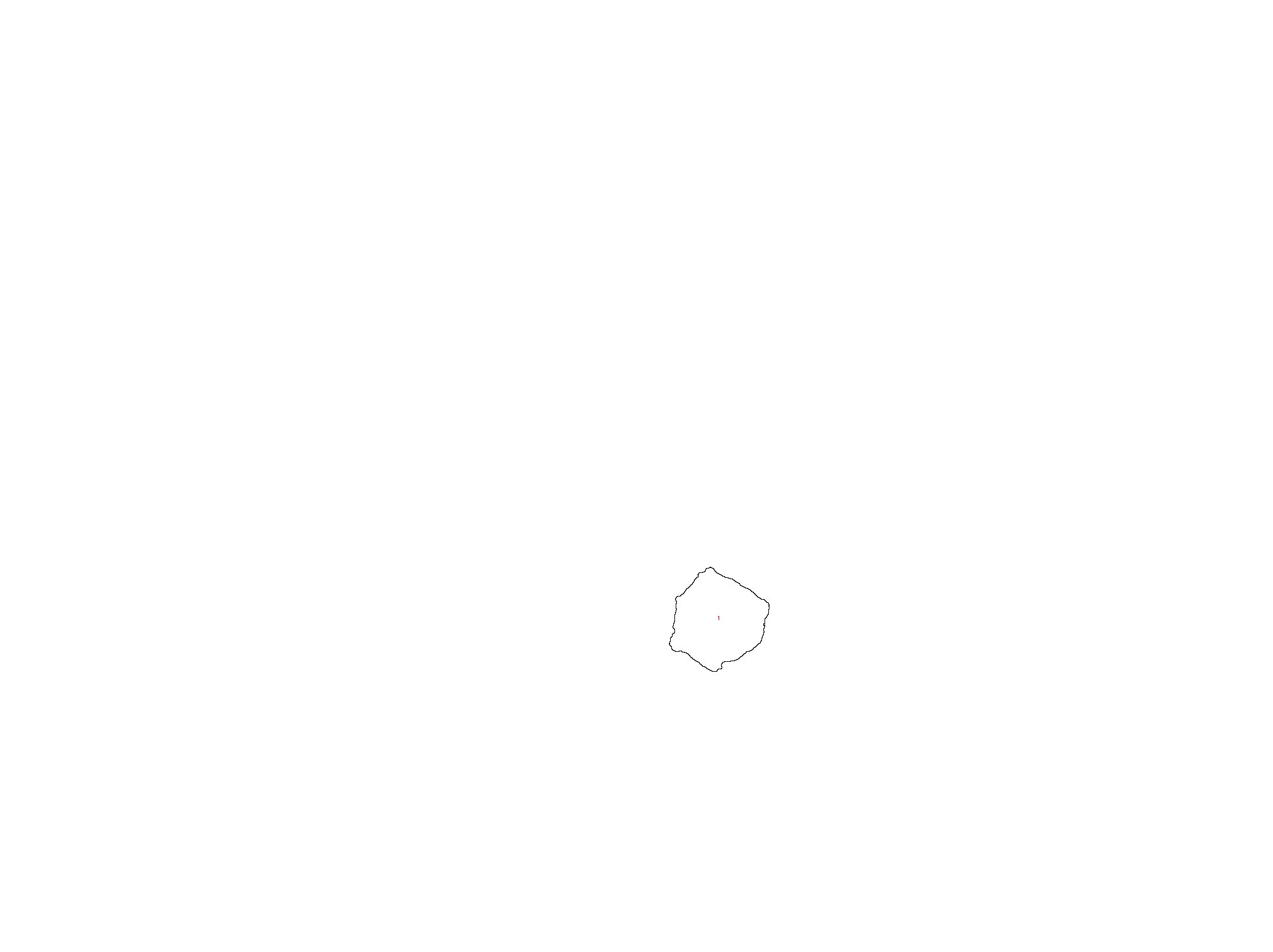

Supplement: S2 Dataset — (ZIP) [file pone.0304198.s005.zip › S2_Dataset_Raw_results_ImageJ/J2_200S_90100_2.jpg]

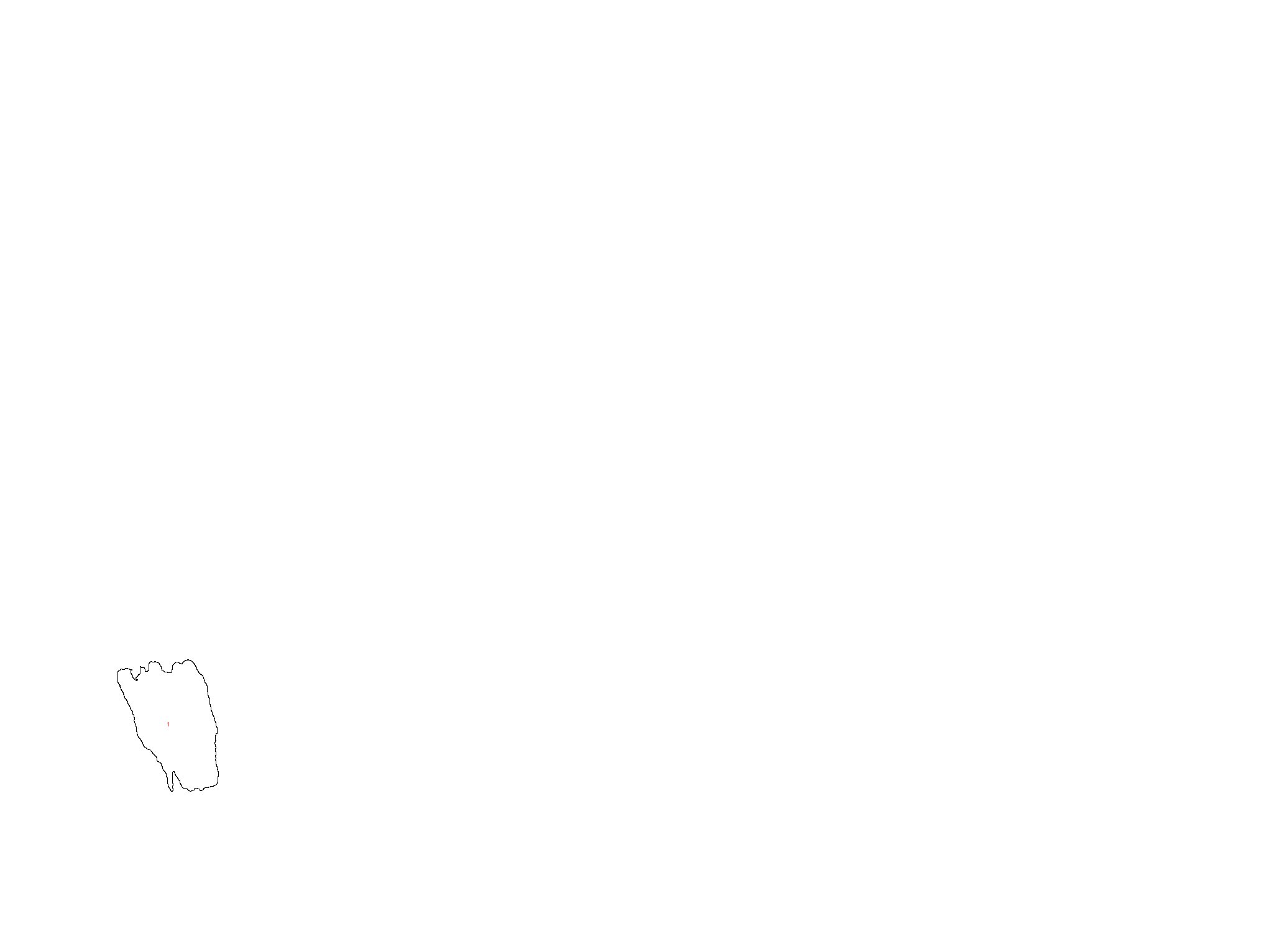

Supplement: S2 Dataset — (ZIP) [file pone.0304198.s005.zip › S2_Dataset_Raw_results_ImageJ/J2_200S_90100_3.jpg]

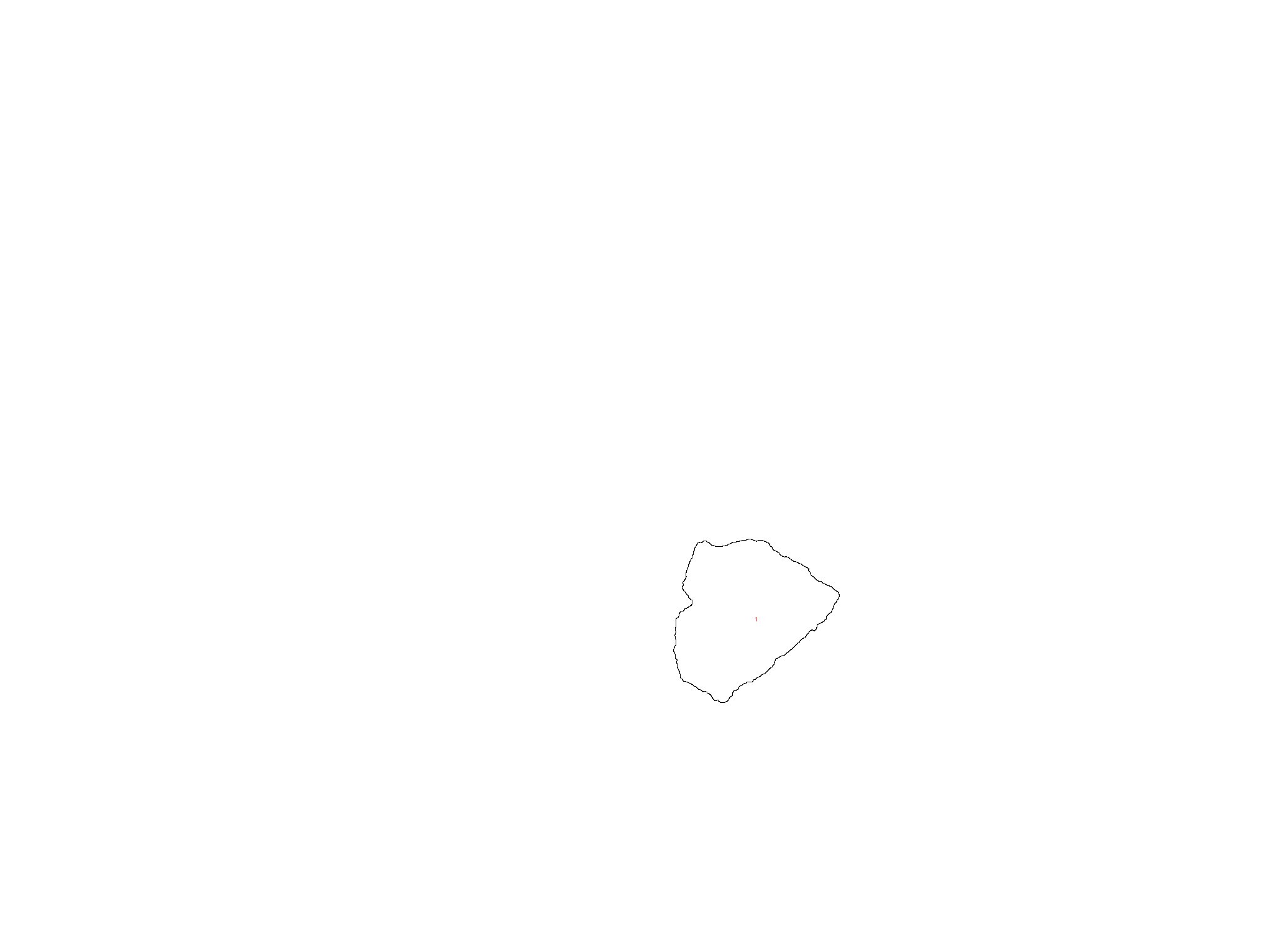

Supplement: S2 Dataset — (ZIP) [file pone.0304198.s005.zip › S2_Dataset_Raw_results_ImageJ/J2_300S_130140_1.jpg]

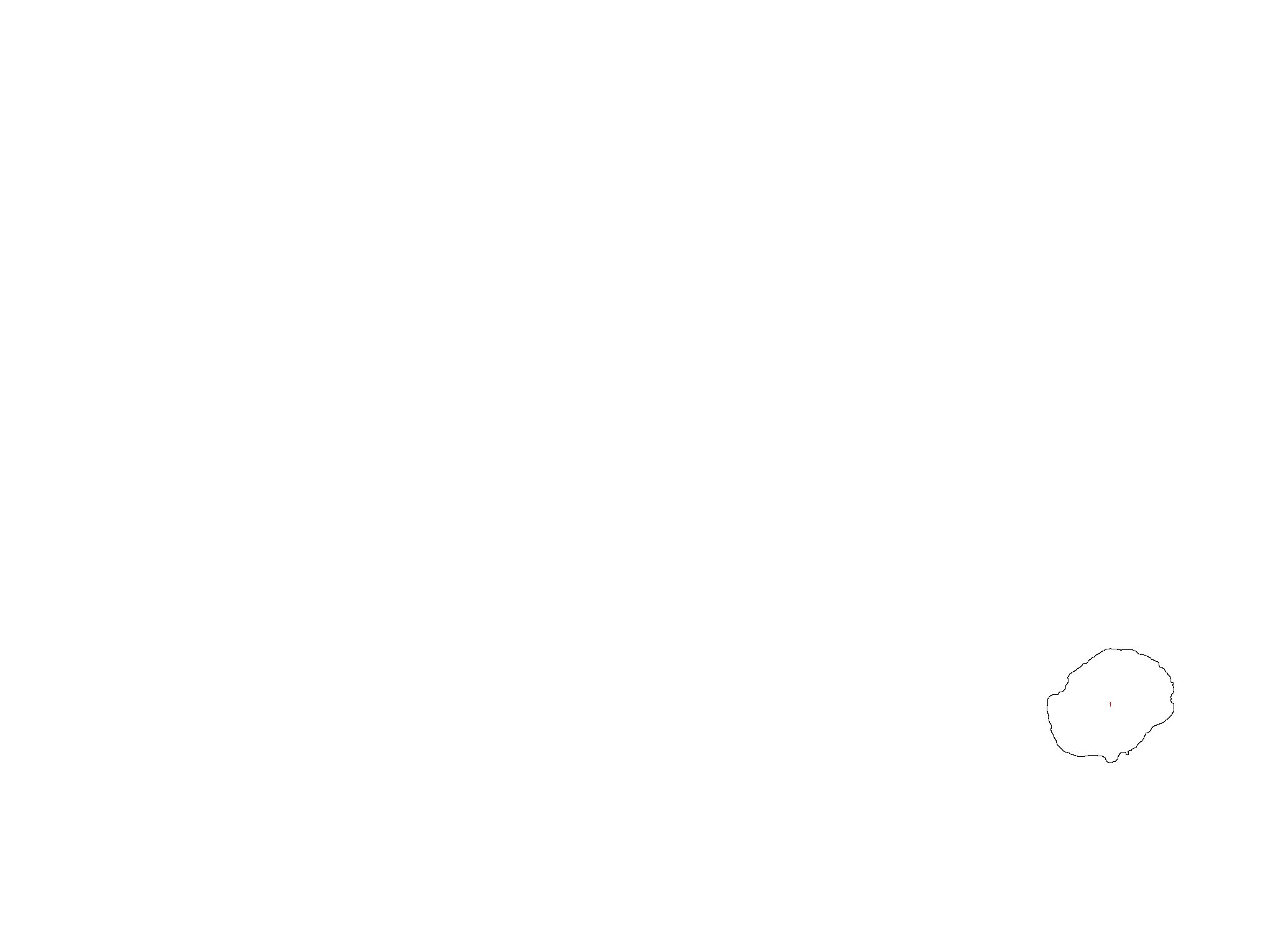

Supplement: S2 Dataset — (ZIP) [file pone.0304198.s005.zip › S2_Dataset_Raw_results_ImageJ/J2_300S_130140_2.jpg]

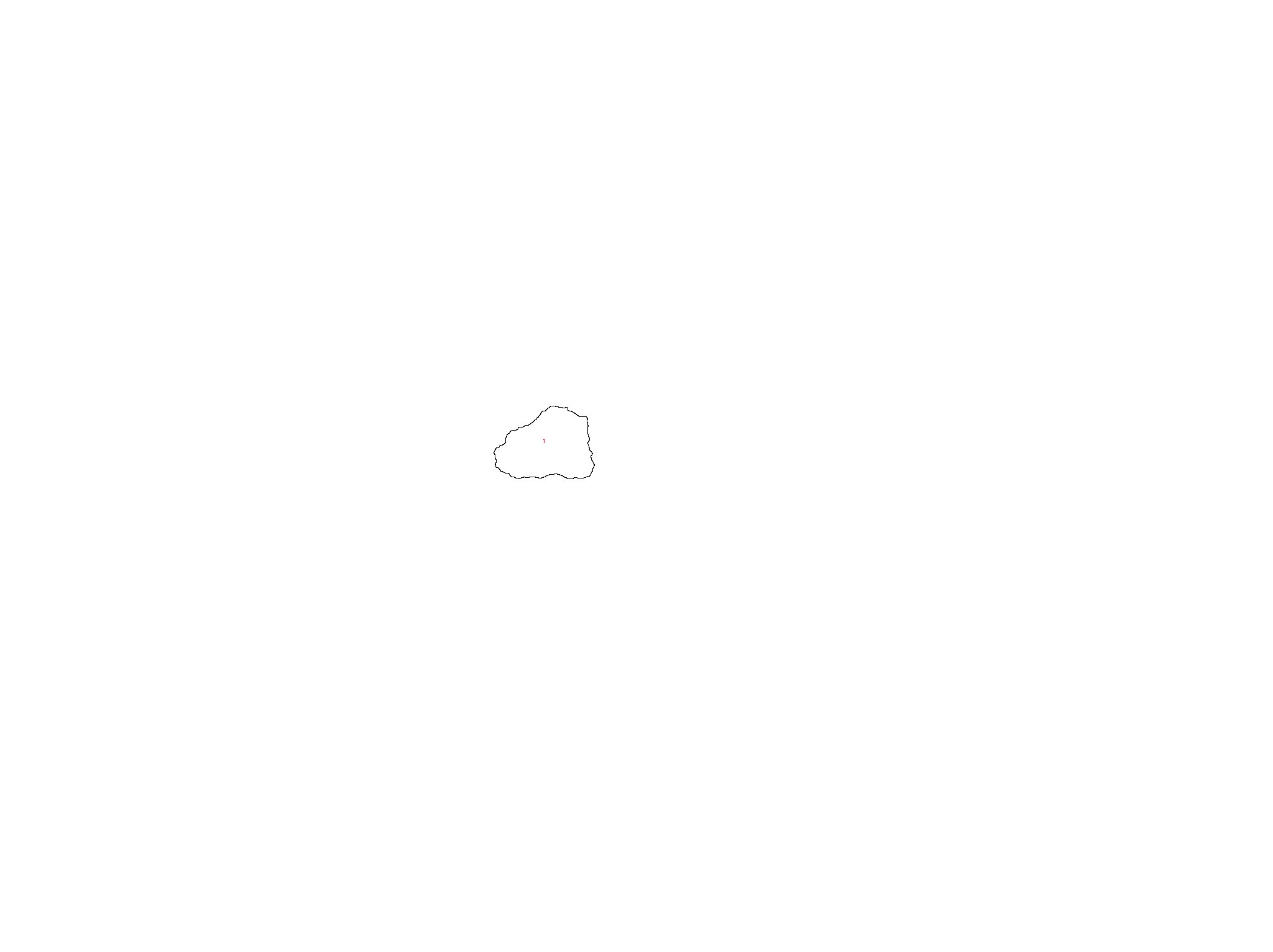

Supplement: S2 Dataset — (ZIP) [file pone.0304198.s005.zip › S2_Dataset_Raw_results_ImageJ/J2_300S_160170_1.jpg]

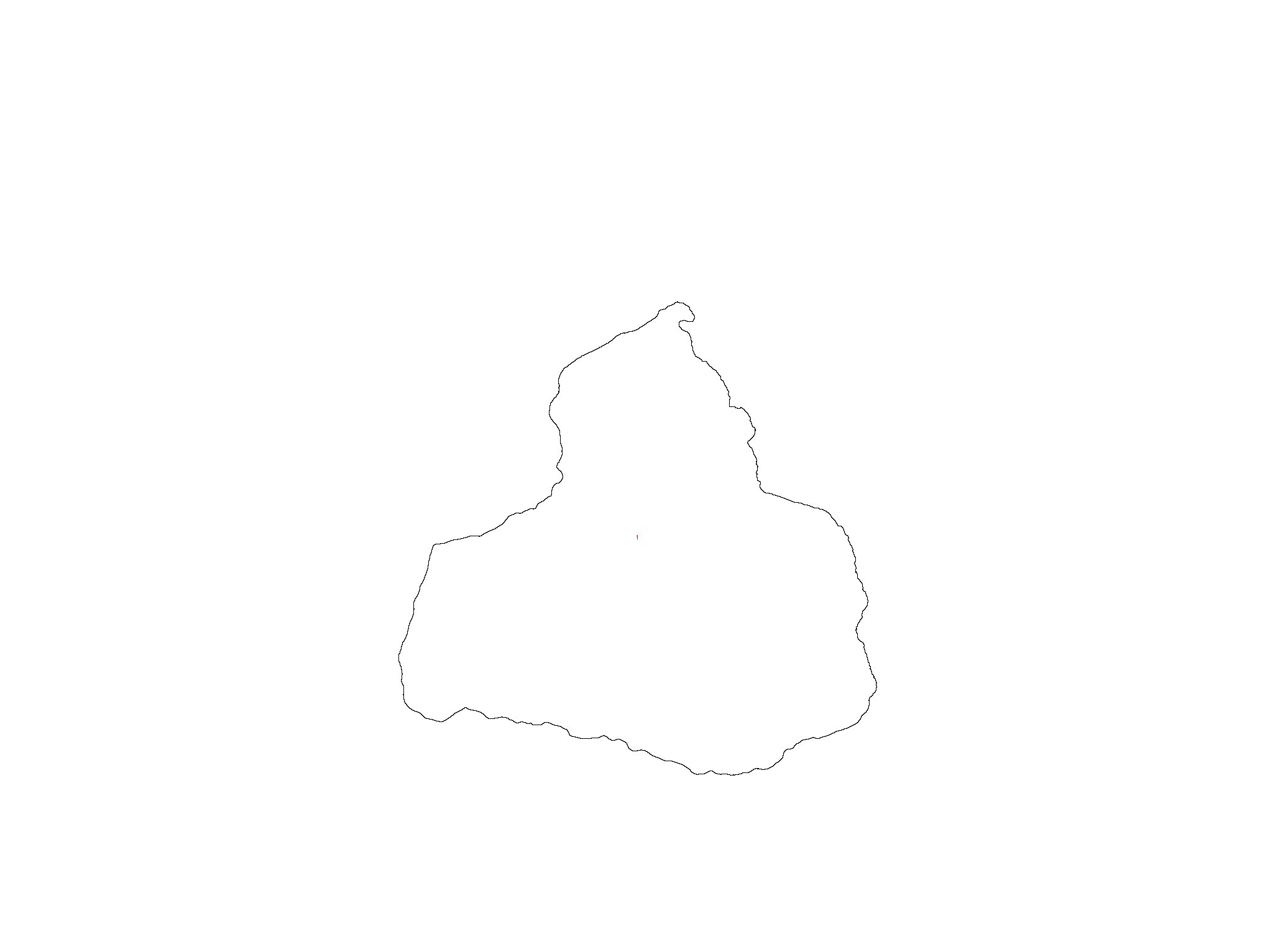

Supplement: S2 Dataset — (ZIP) [file pone.0304198.s005.zip › S2_Dataset_Raw_results_ImageJ/J2_300S_4050_1.jpg]

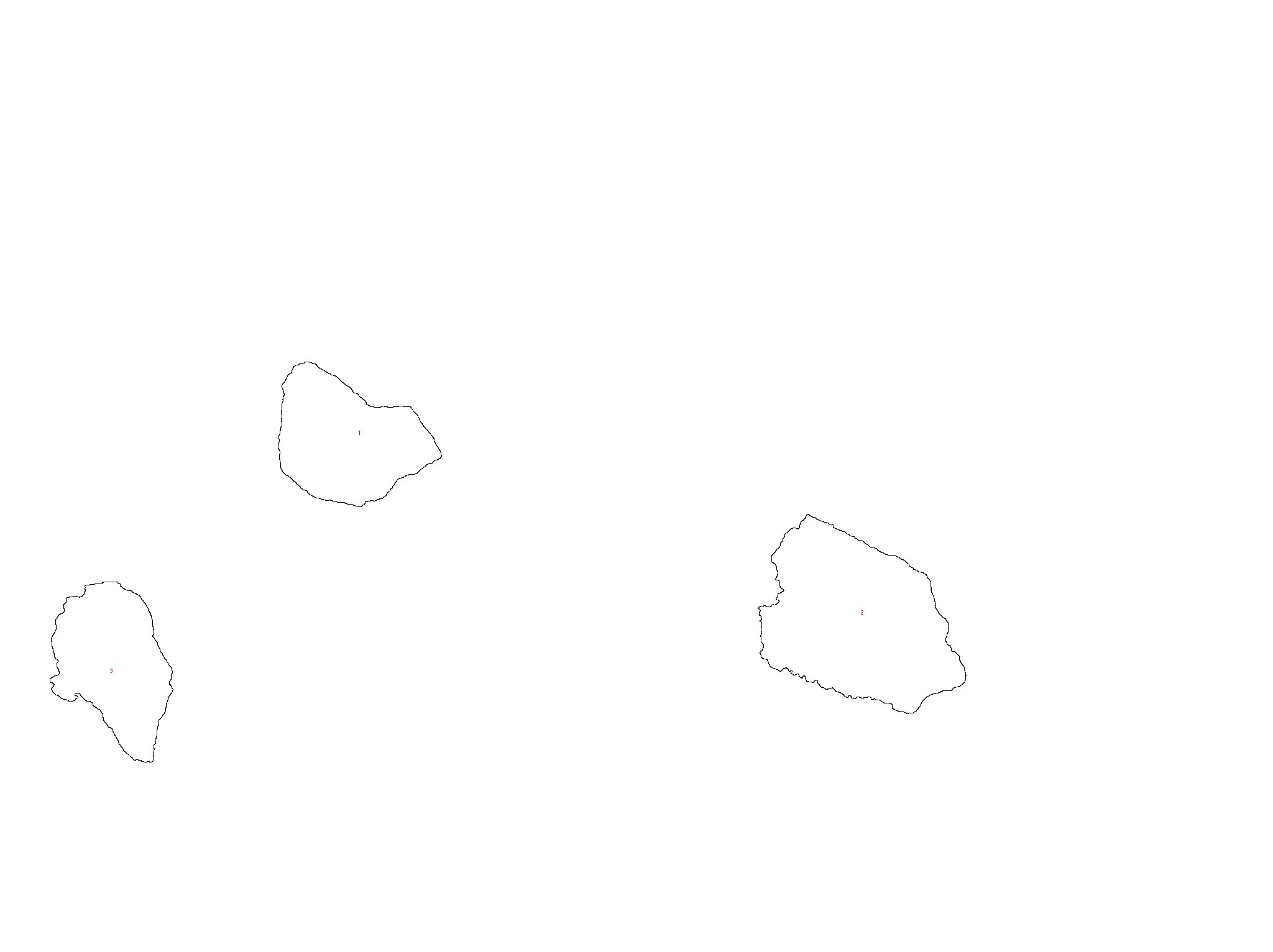

Supplement: S2 Dataset — (ZIP) [file pone.0304198.s005.zip › S2_Dataset_Raw_results_ImageJ/J2_300S_4050_10.jpg]

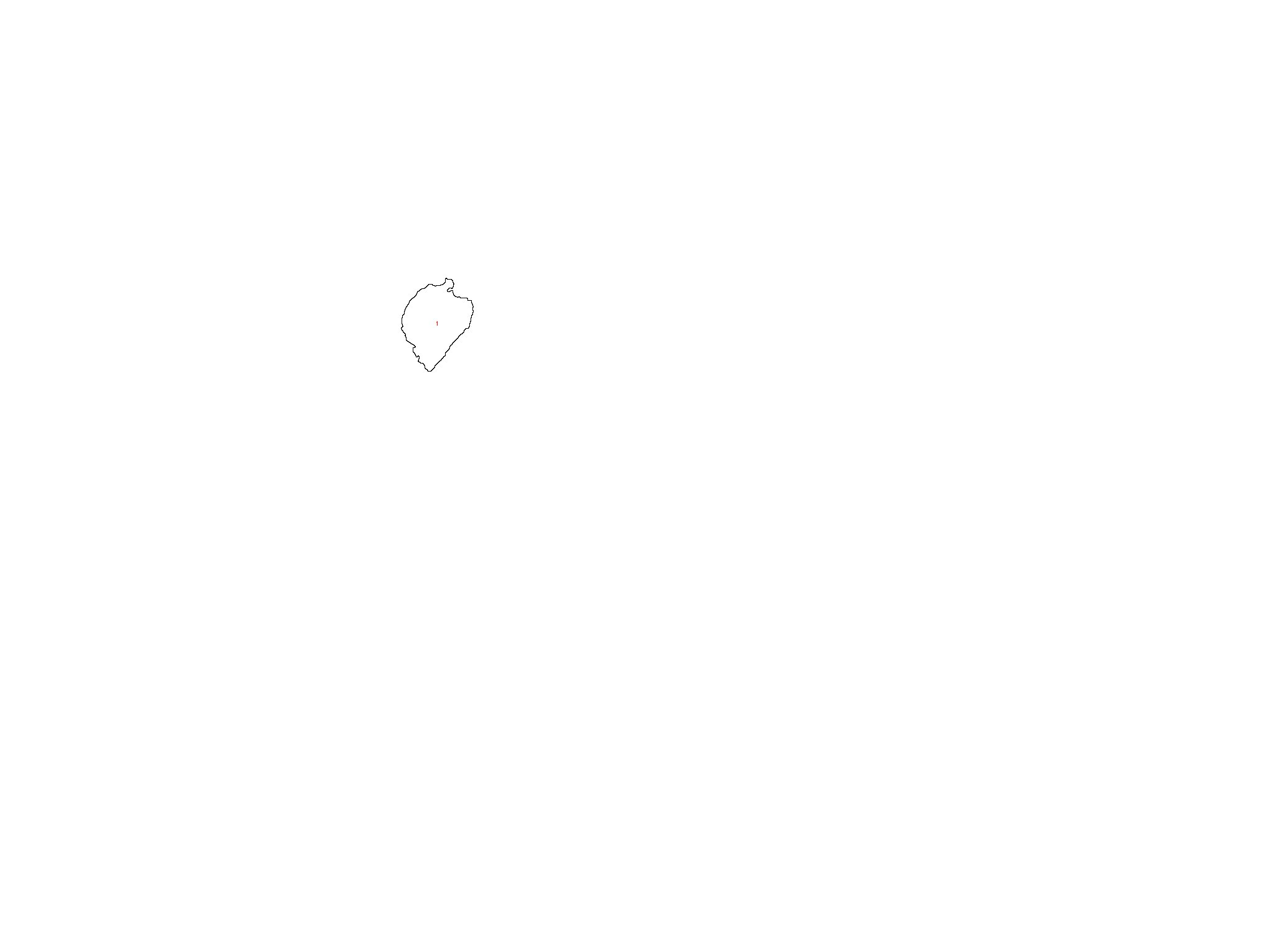

Supplement: S2 Dataset — (ZIP) [file pone.0304198.s005.zip › S2_Dataset_Raw_results_ImageJ/J2_300S_4050_11.jpg]

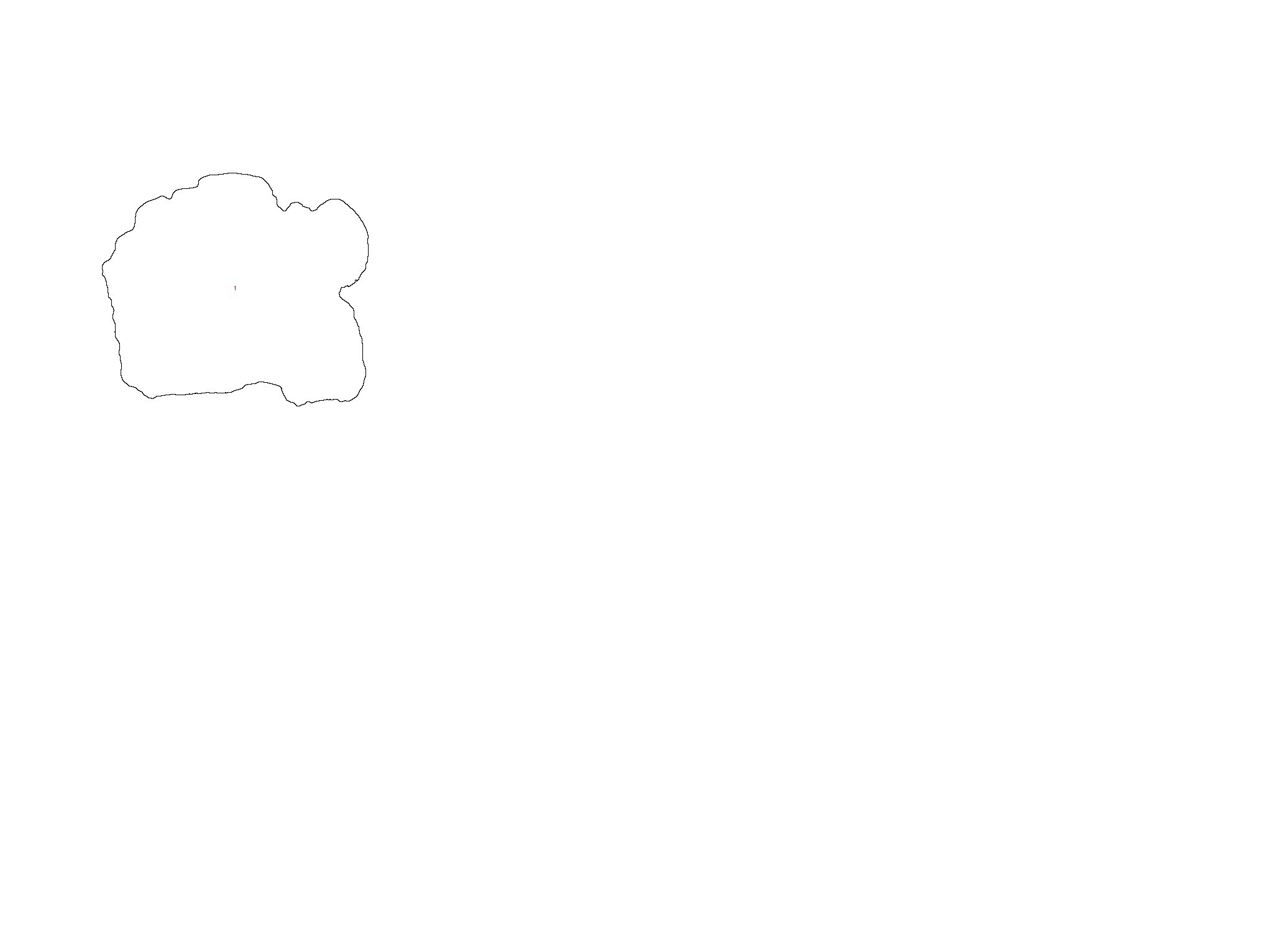

Supplement: S2 Dataset — (ZIP) [file pone.0304198.s005.zip › S2_Dataset_Raw_results_ImageJ/J2_300S_4050_2.jpg]

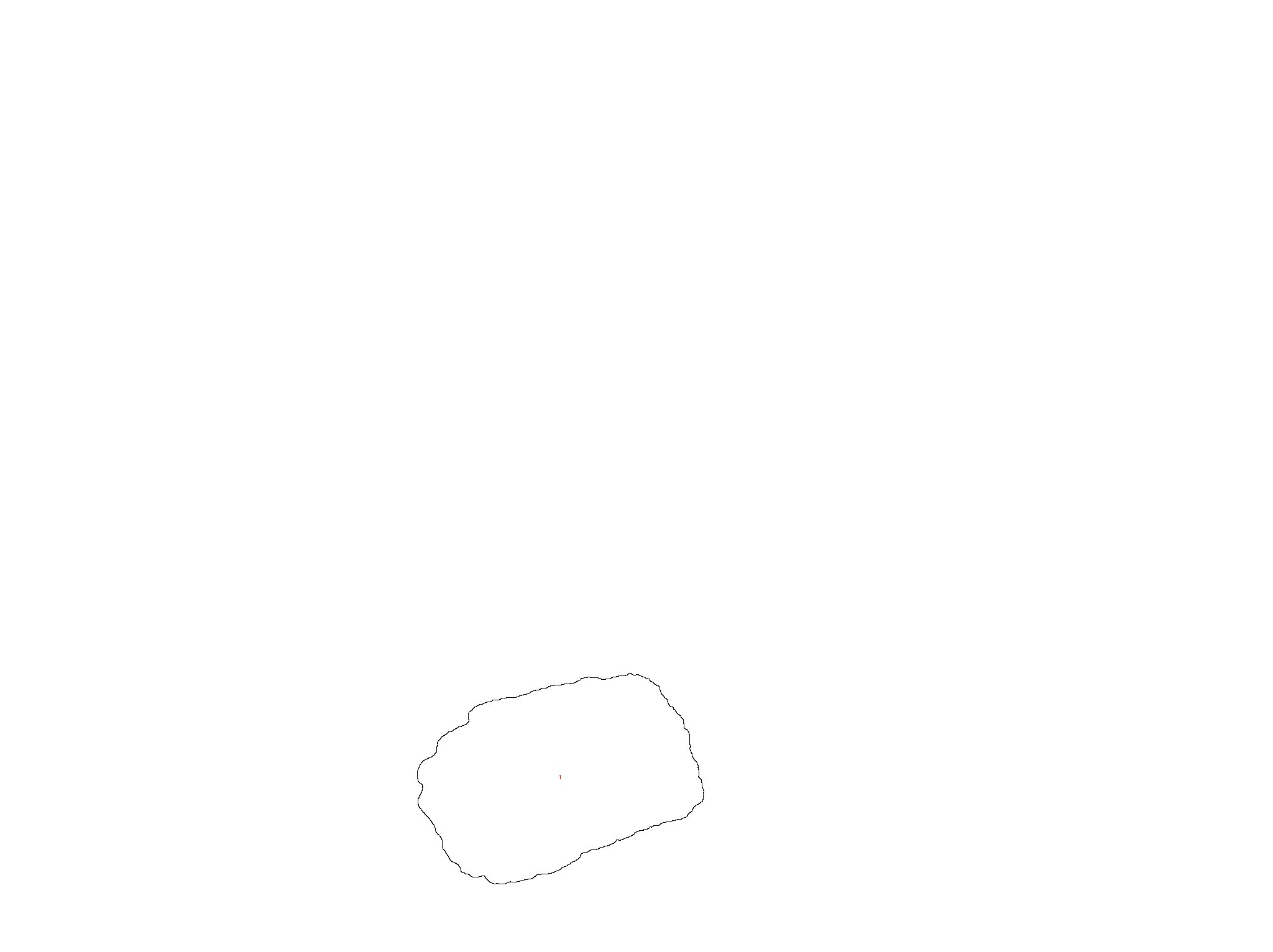

Supplement: S2 Dataset — (ZIP) [file pone.0304198.s005.zip › S2_Dataset_Raw_results_ImageJ/J2_300S_4050_4.jpg]

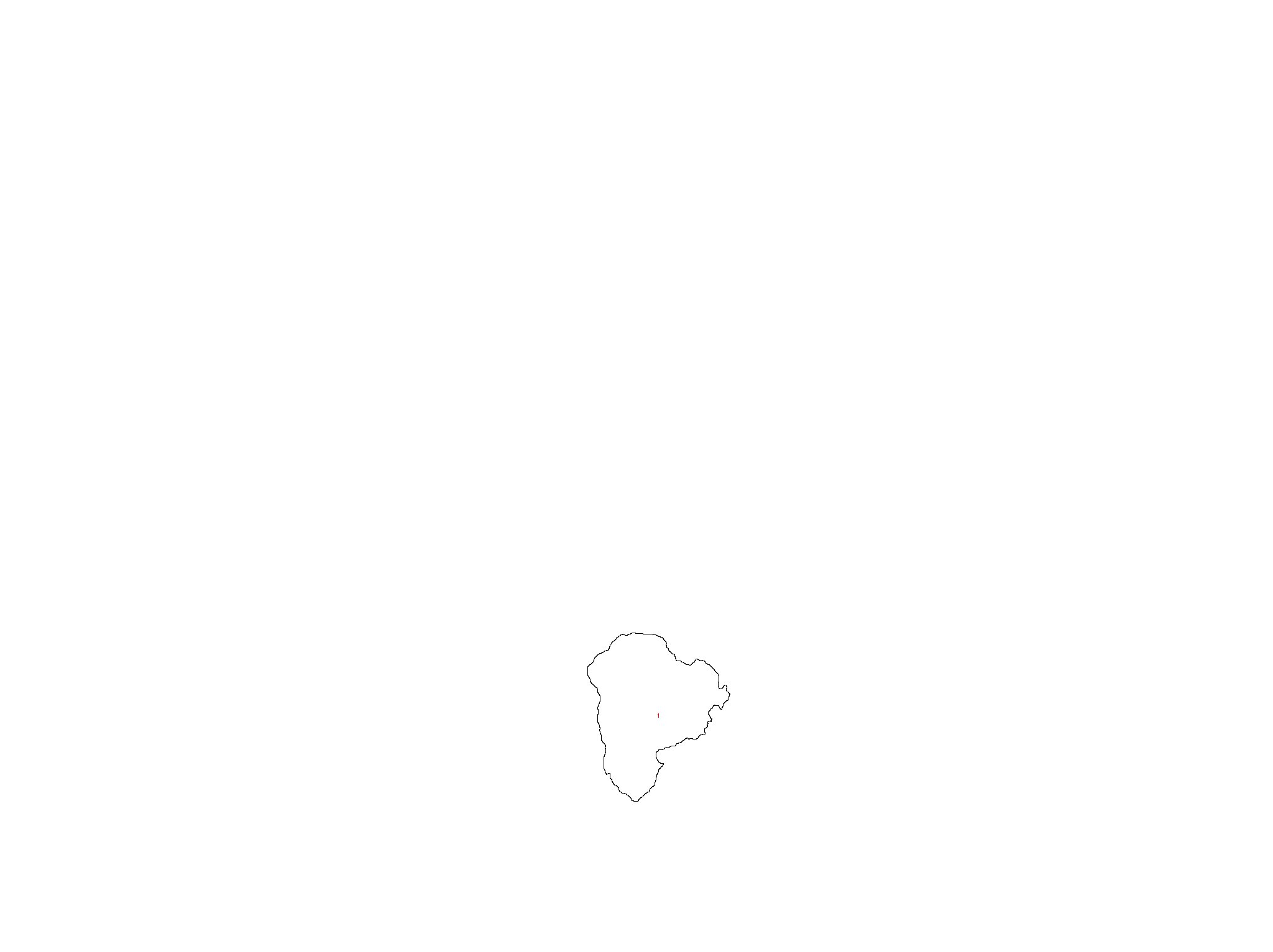

Supplement: S2 Dataset — (ZIP) [file pone.0304198.s005.zip › S2_Dataset_Raw_results_ImageJ/J2_300S_4050_5.jpg]

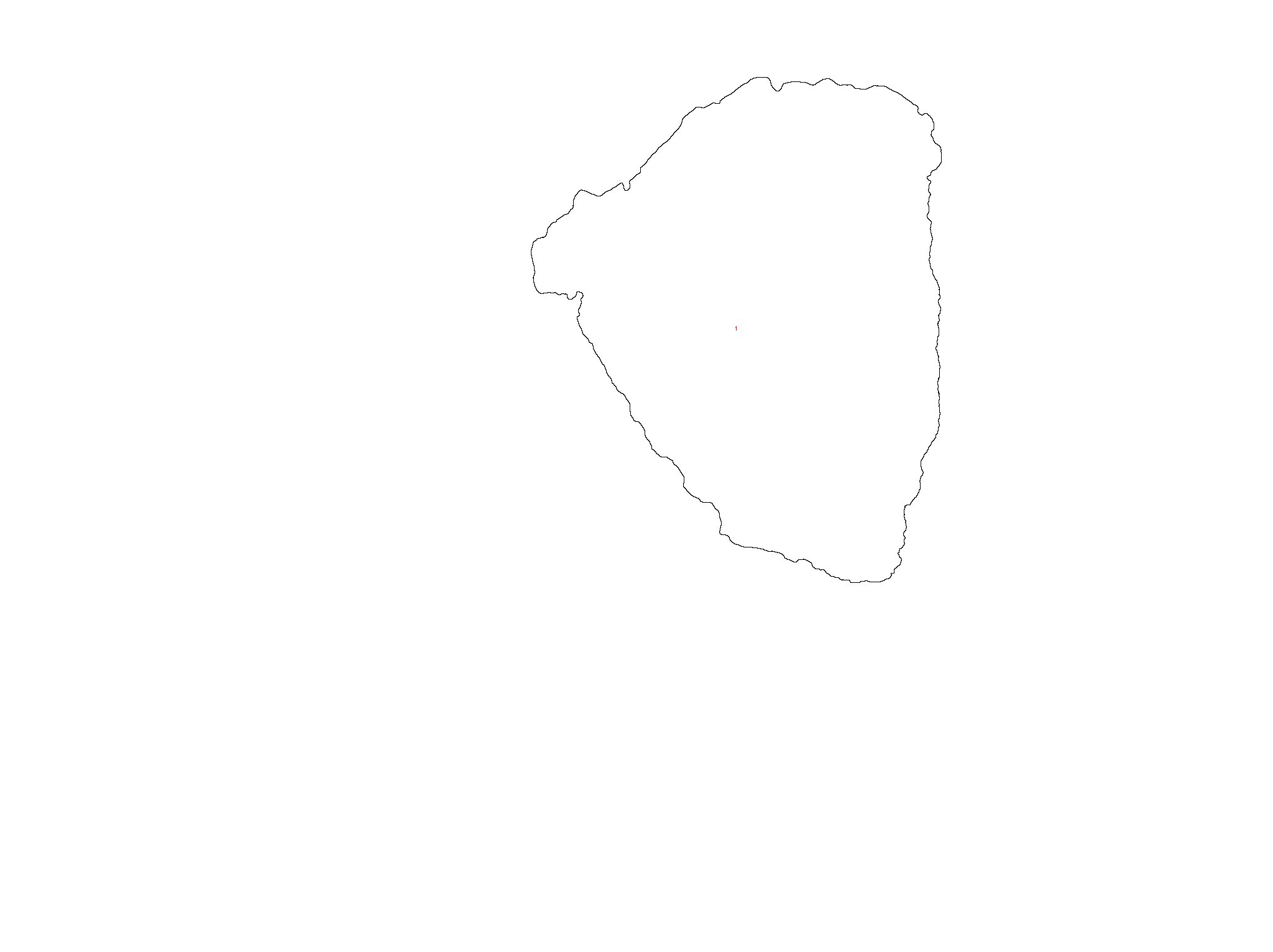

Supplement: S2 Dataset — (ZIP) [file pone.0304198.s005.zip › S2_Dataset_Raw_results_ImageJ/J2_300S_4050_6.jpg]

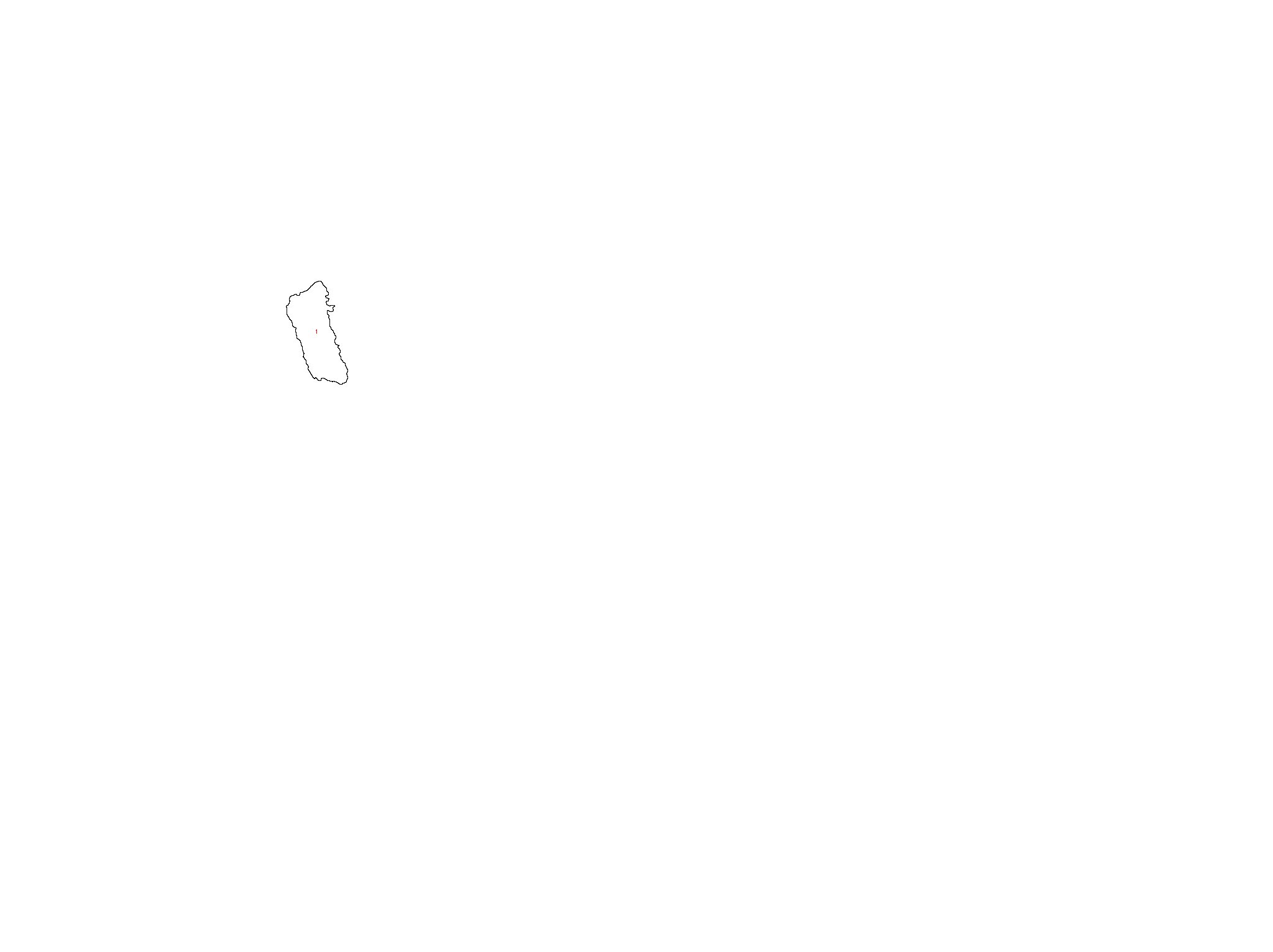

Supplement: S2 Dataset — (ZIP) [file pone.0304198.s005.zip › S2_Dataset_Raw_results_ImageJ/J2_300S_4050_7.jpg]

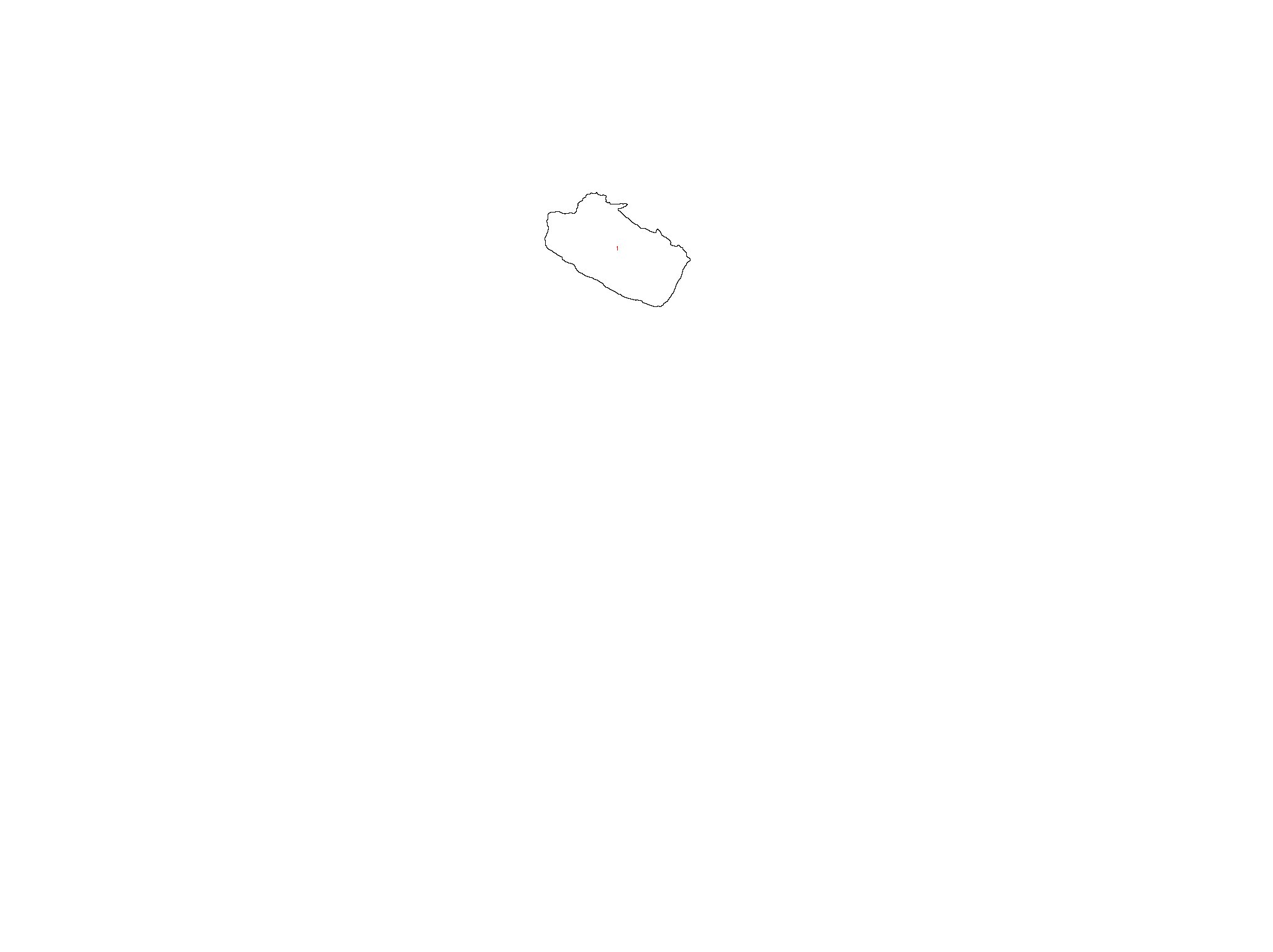

Supplement: S2 Dataset — (ZIP) [file pone.0304198.s005.zip › S2_Dataset_Raw_results_ImageJ/J2_300S_4050_8.jpg]

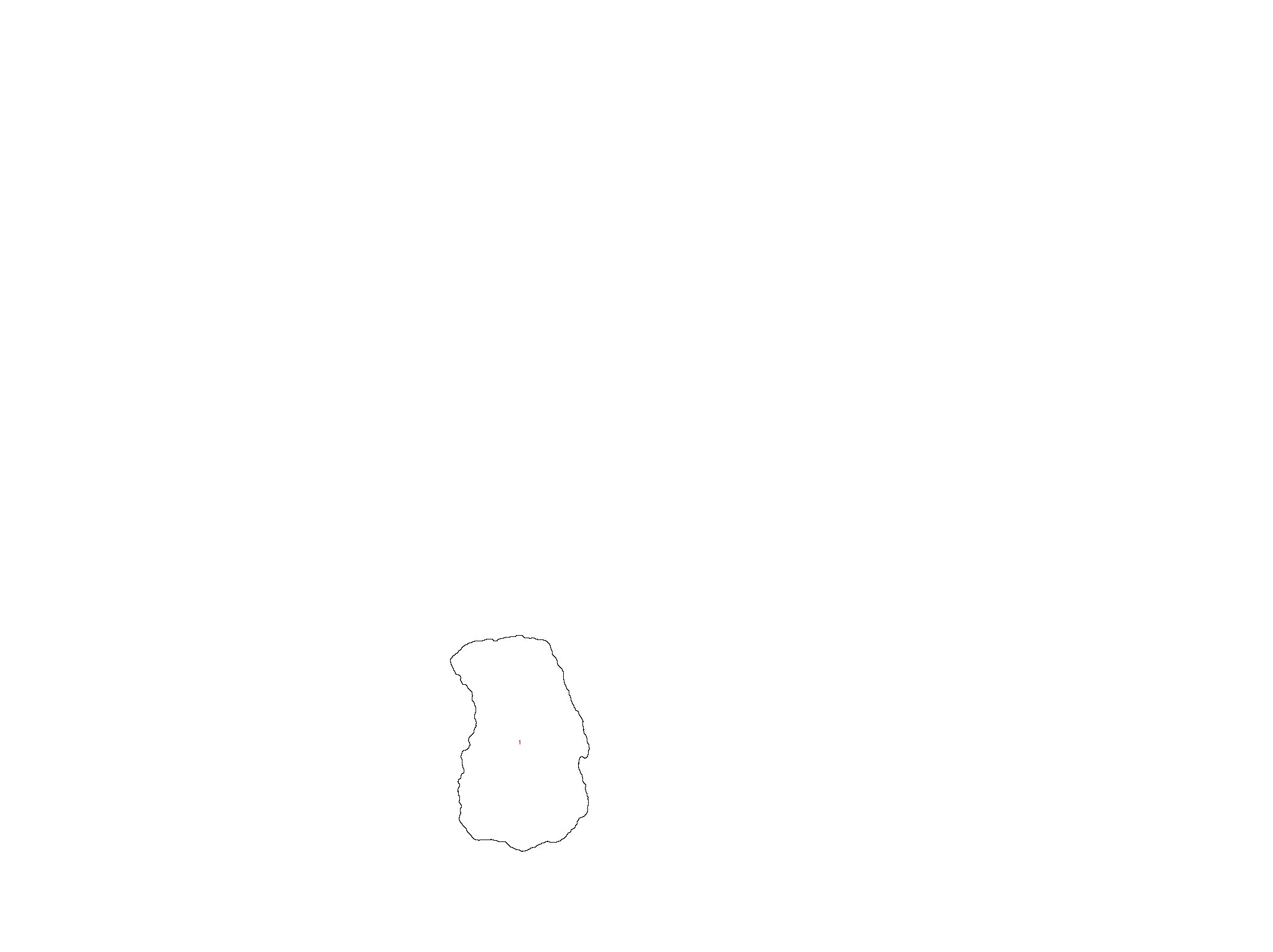

Supplement: S2 Dataset — (ZIP) [file pone.0304198.s005.zip › S2_Dataset_Raw_results_ImageJ/J2_300S_6070_1.jpg]

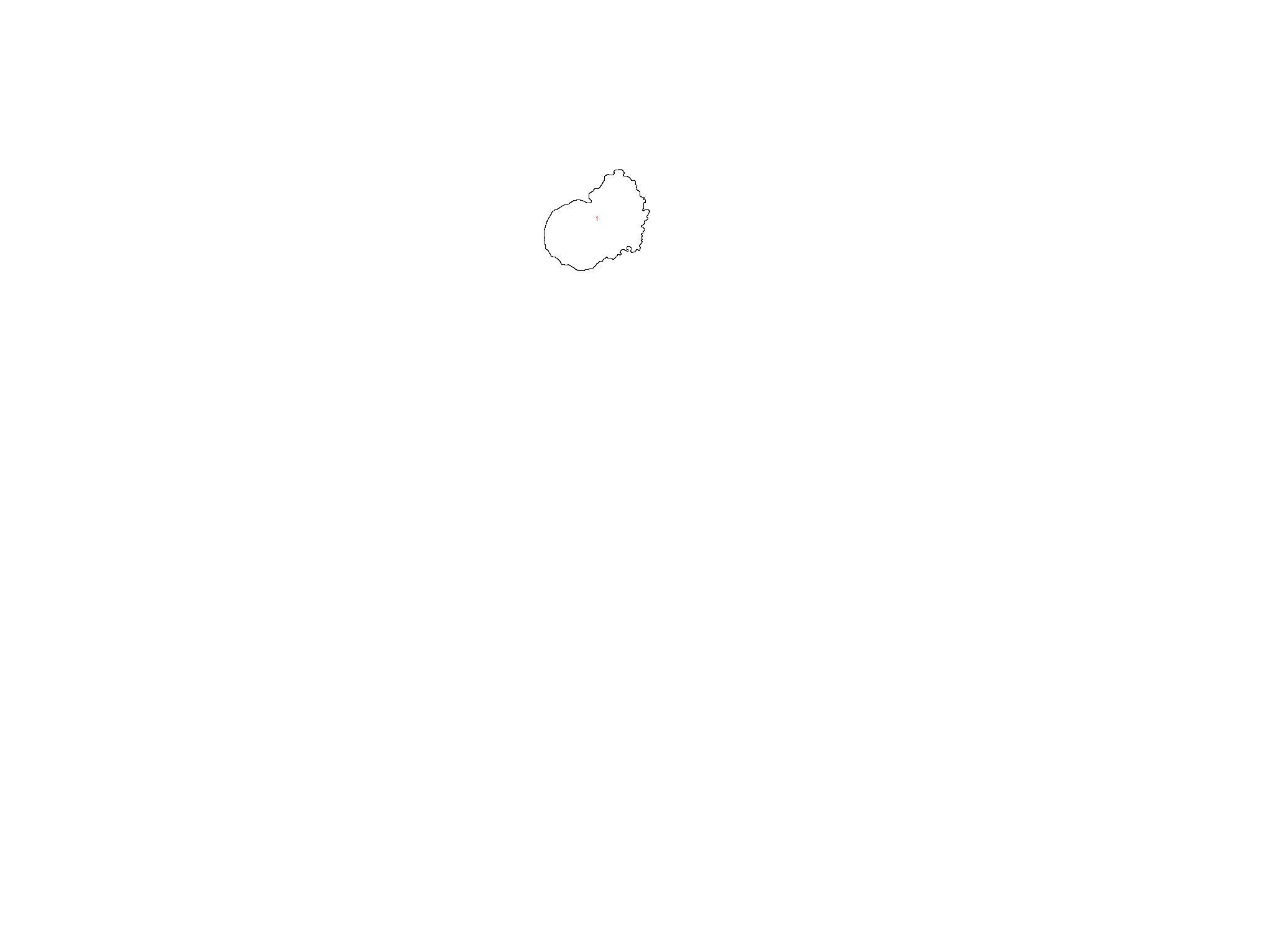

Supplement: S2 Dataset — (ZIP) [file pone.0304198.s005.zip › S2_Dataset_Raw_results_ImageJ/J2_300S_6070_2.jpg]

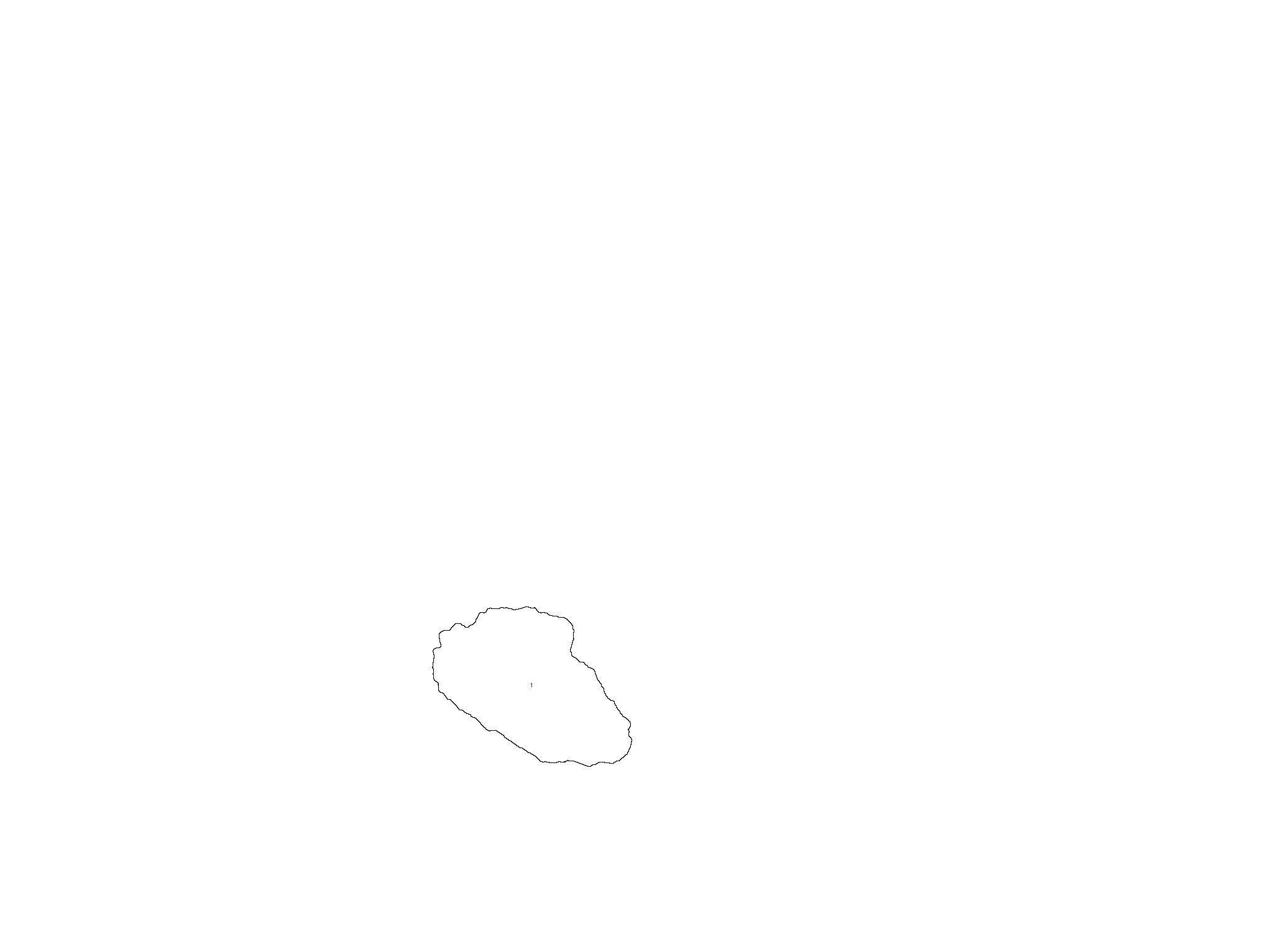

Supplement: S2 Dataset — (ZIP) [file pone.0304198.s005.zip › S2_Dataset_Raw_results_ImageJ/J2_300S_6070_3.jpg]

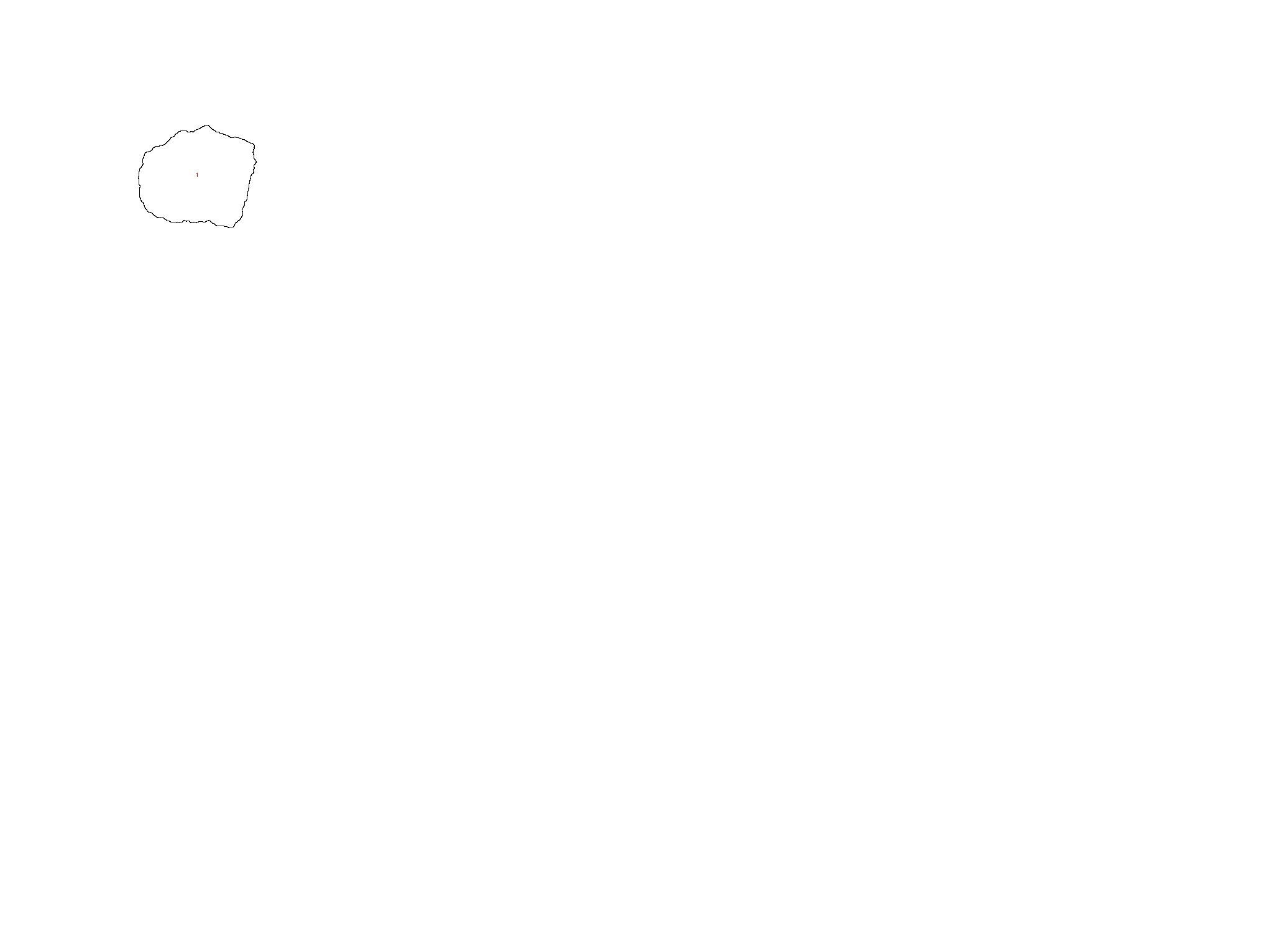

Supplement: S2 Dataset — (ZIP) [file pone.0304198.s005.zip › S2_Dataset_Raw_results_ImageJ/J2_300S_6070_4.jpg]

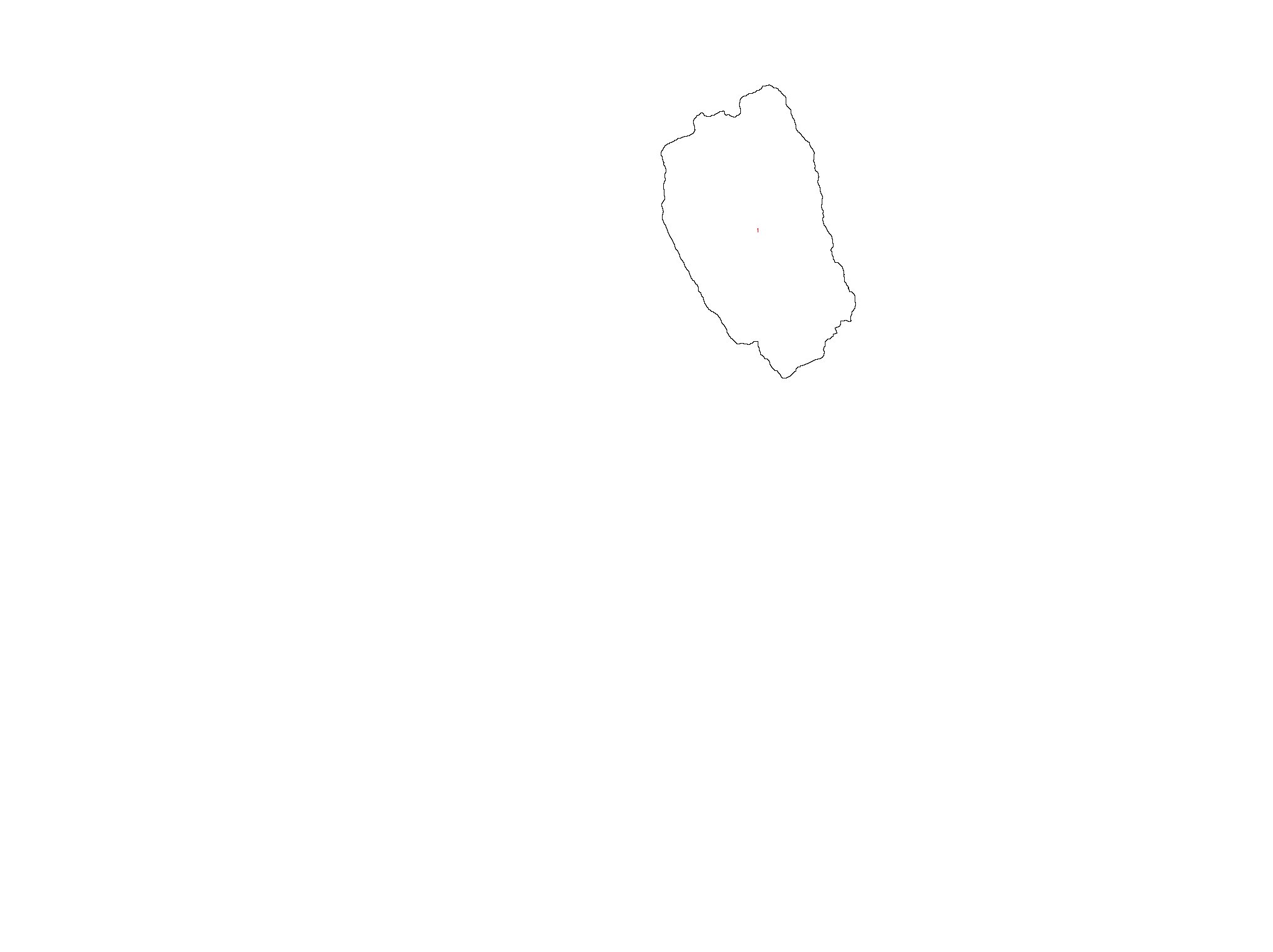

Supplement: S2 Dataset — (ZIP) [file pone.0304198.s005.zip › S2_Dataset_Raw_results_ImageJ/J2_300S_6070_5.jpg]

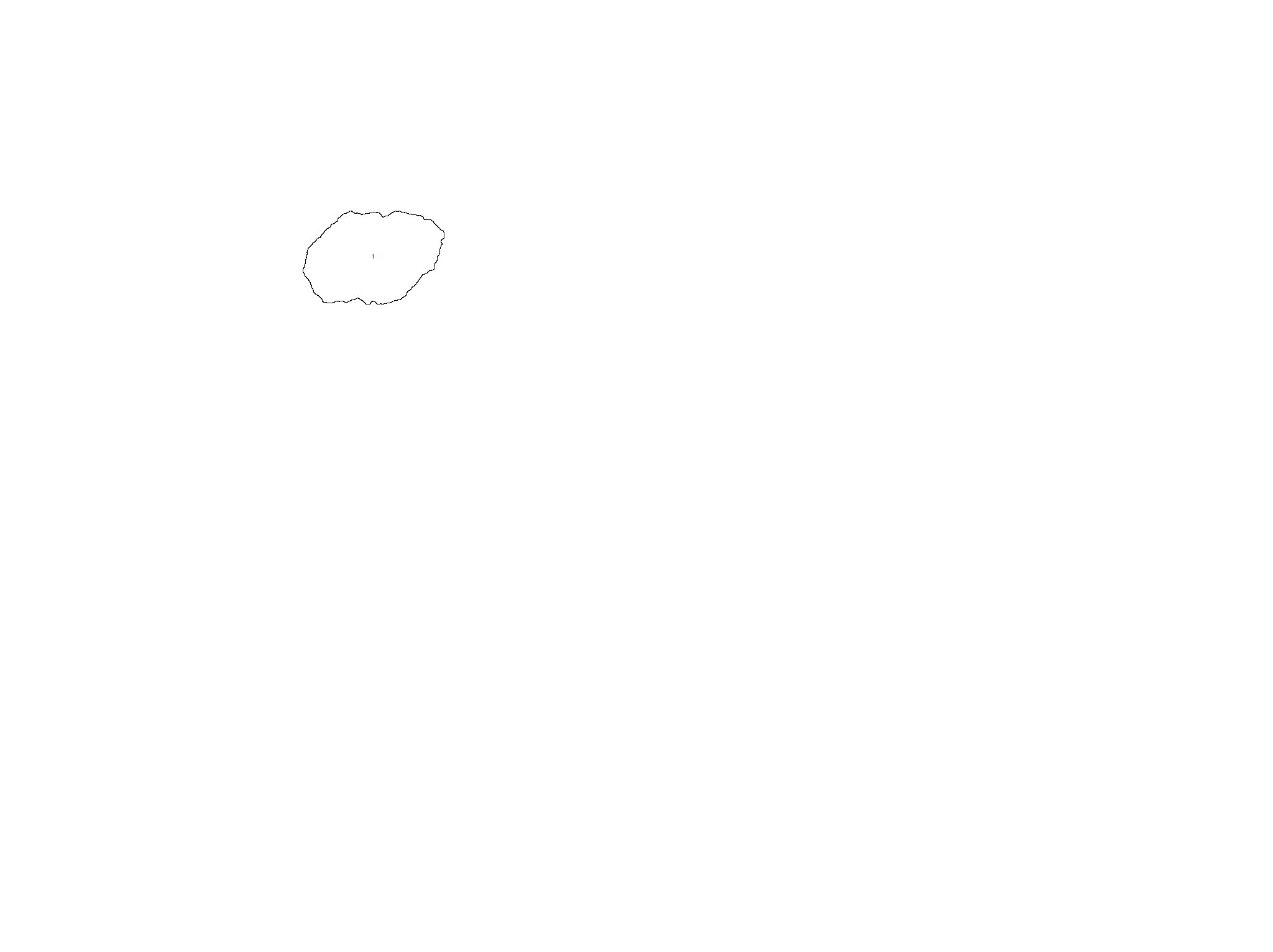

Supplement: S2 Dataset — (ZIP) [file pone.0304198.s005.zip › S2_Dataset_Raw_results_ImageJ/J2_300S_6070_6.jpg]

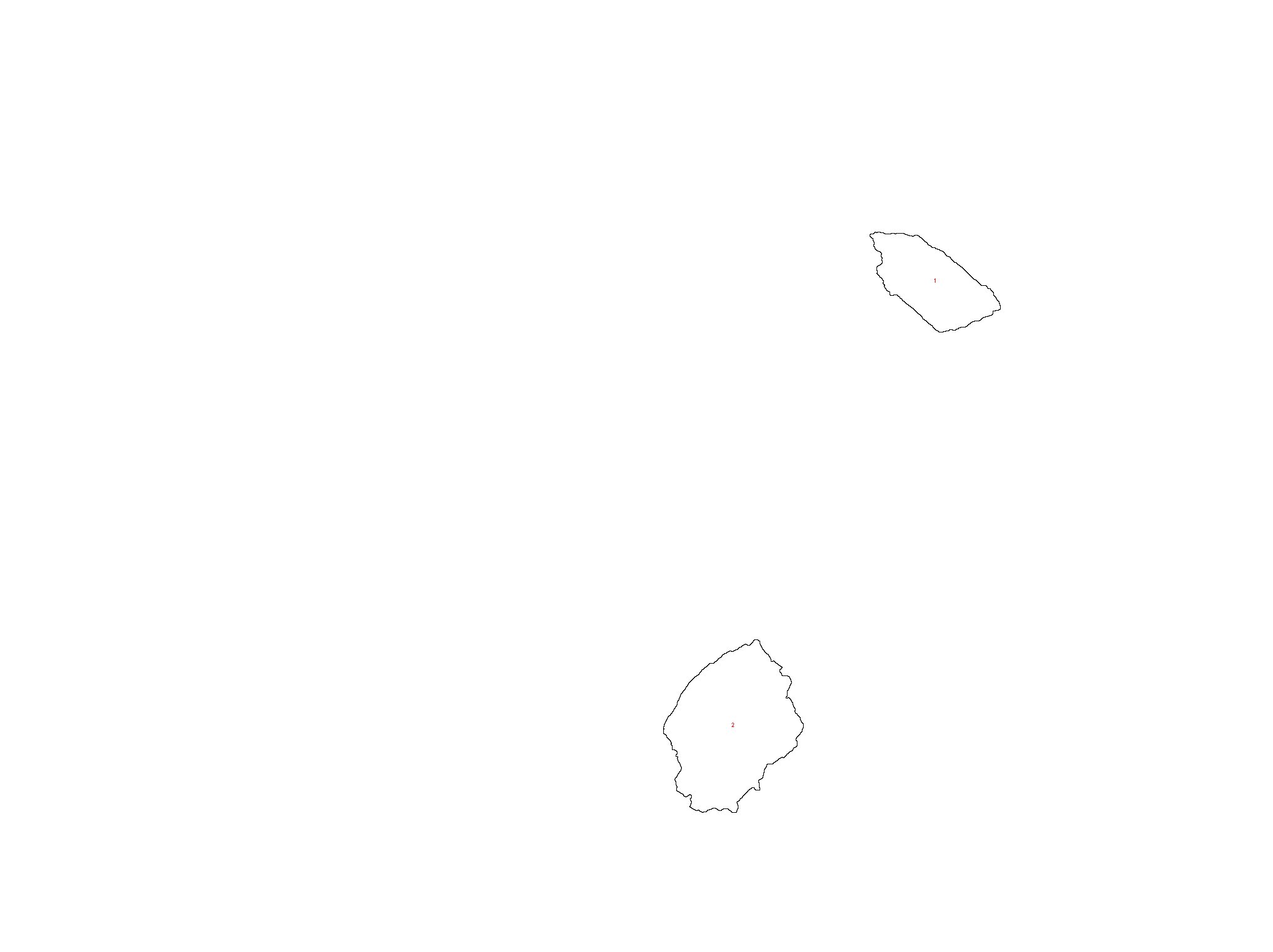

Supplement: S2 Dataset — (ZIP) [file pone.0304198.s005.zip › S2_Dataset_Raw_results_ImageJ/J2_300S_7080_1.jpg]

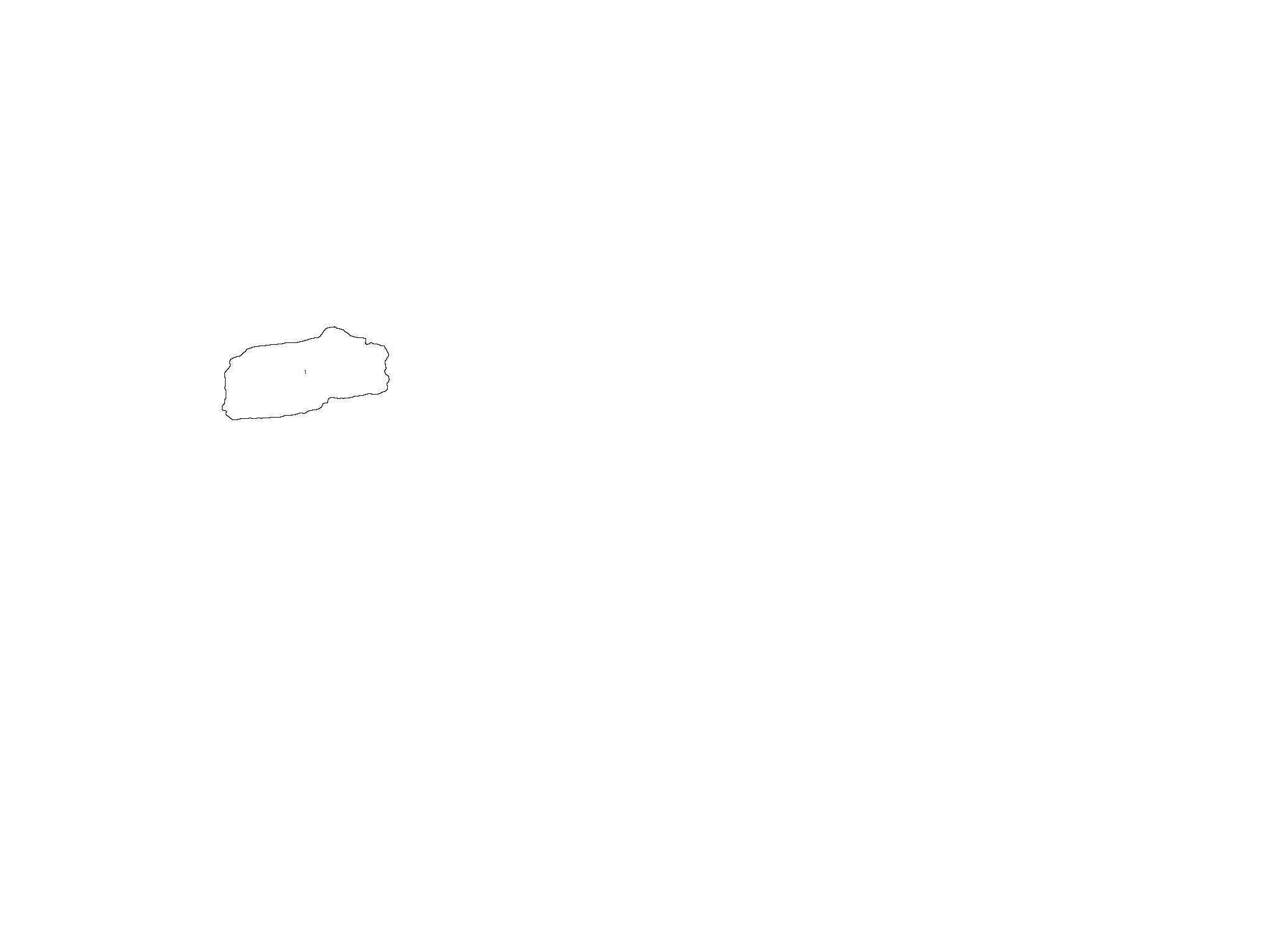

Supplement: S2 Dataset — (ZIP) [file pone.0304198.s005.zip › S2_Dataset_Raw_results_ImageJ/J2_300S_7080_2.jpg]

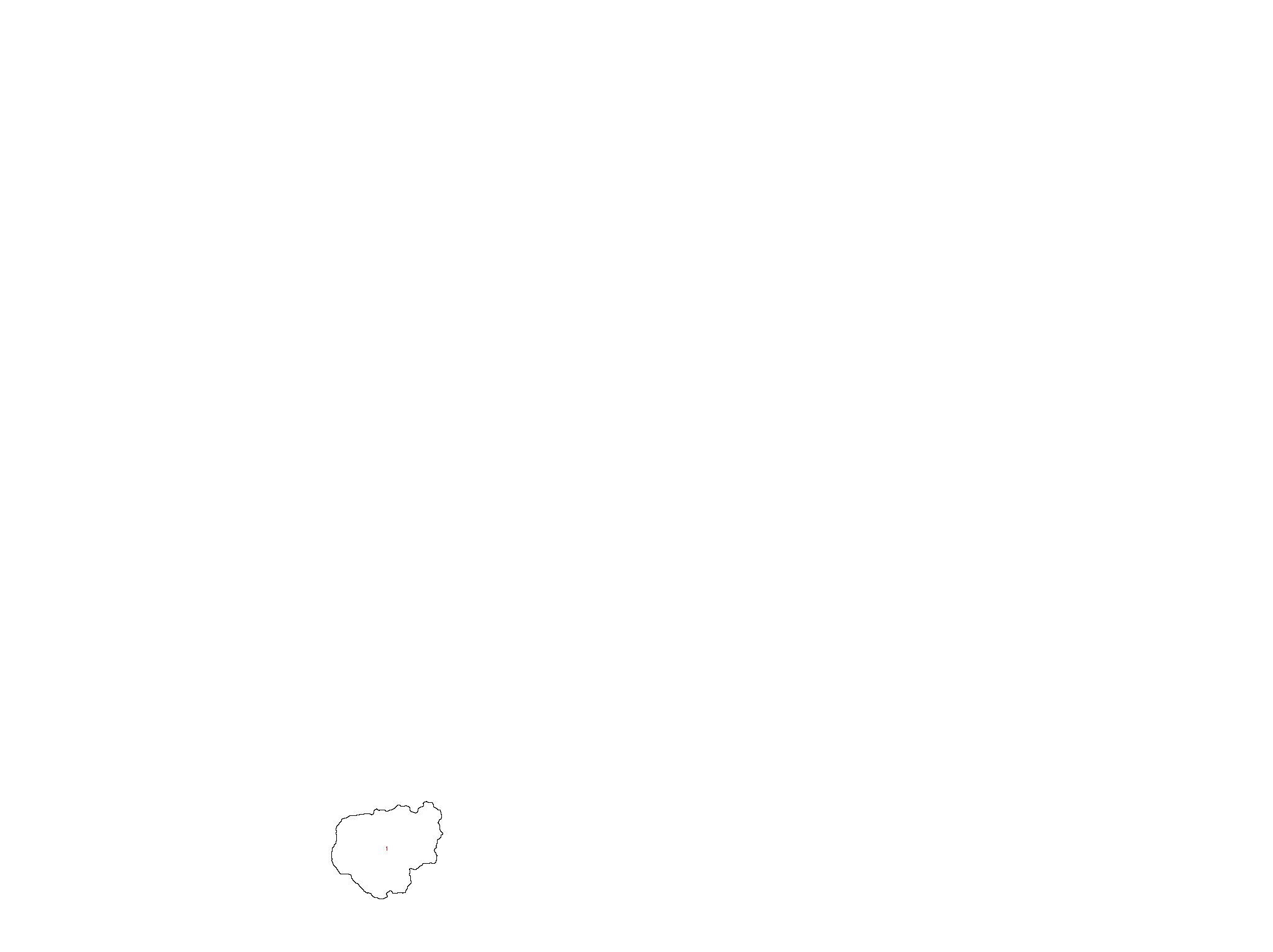

Supplement: S2 Dataset — (ZIP) [file pone.0304198.s005.zip › S2_Dataset_Raw_results_ImageJ/J2_300S_7080_3.jpg]

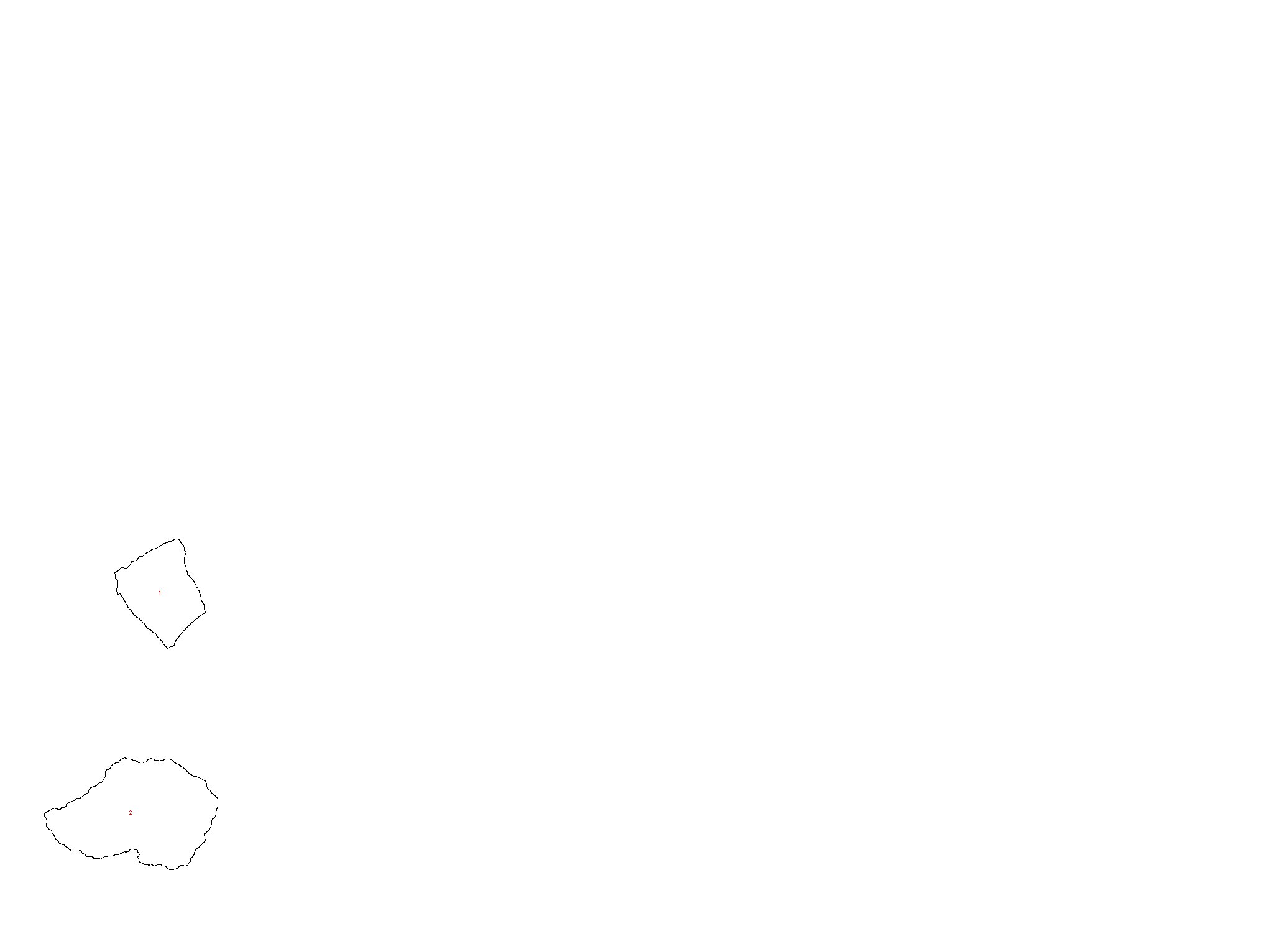

Supplement: S2 Dataset — (ZIP) [file pone.0304198.s005.zip › S2_Dataset_Raw_results_ImageJ/J2_300S_7080_4.jpg]

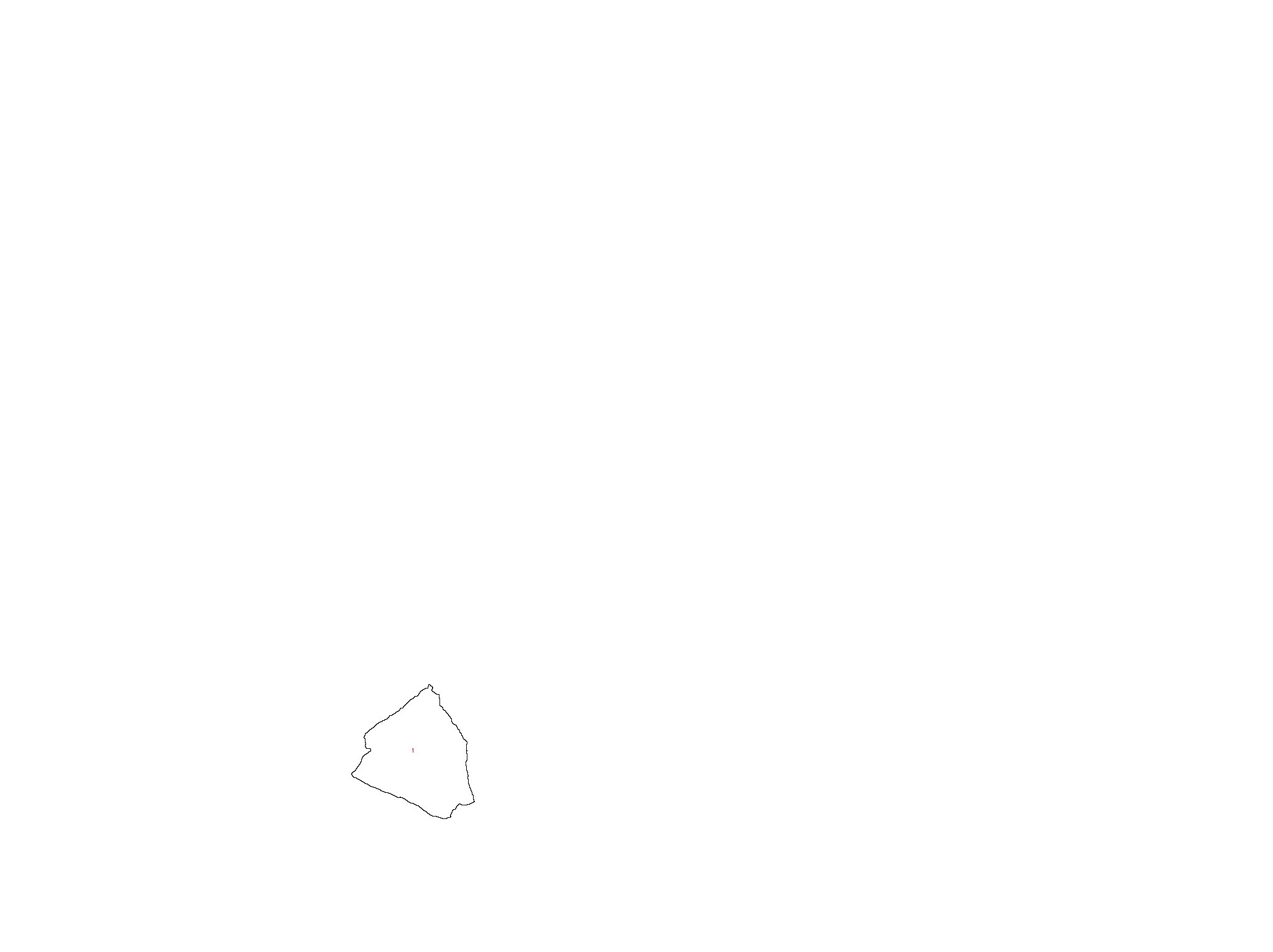

Supplement: S2 Dataset — (ZIP) [file pone.0304198.s005.zip › S2_Dataset_Raw_results_ImageJ/J2_400S_100110_1.jpg]

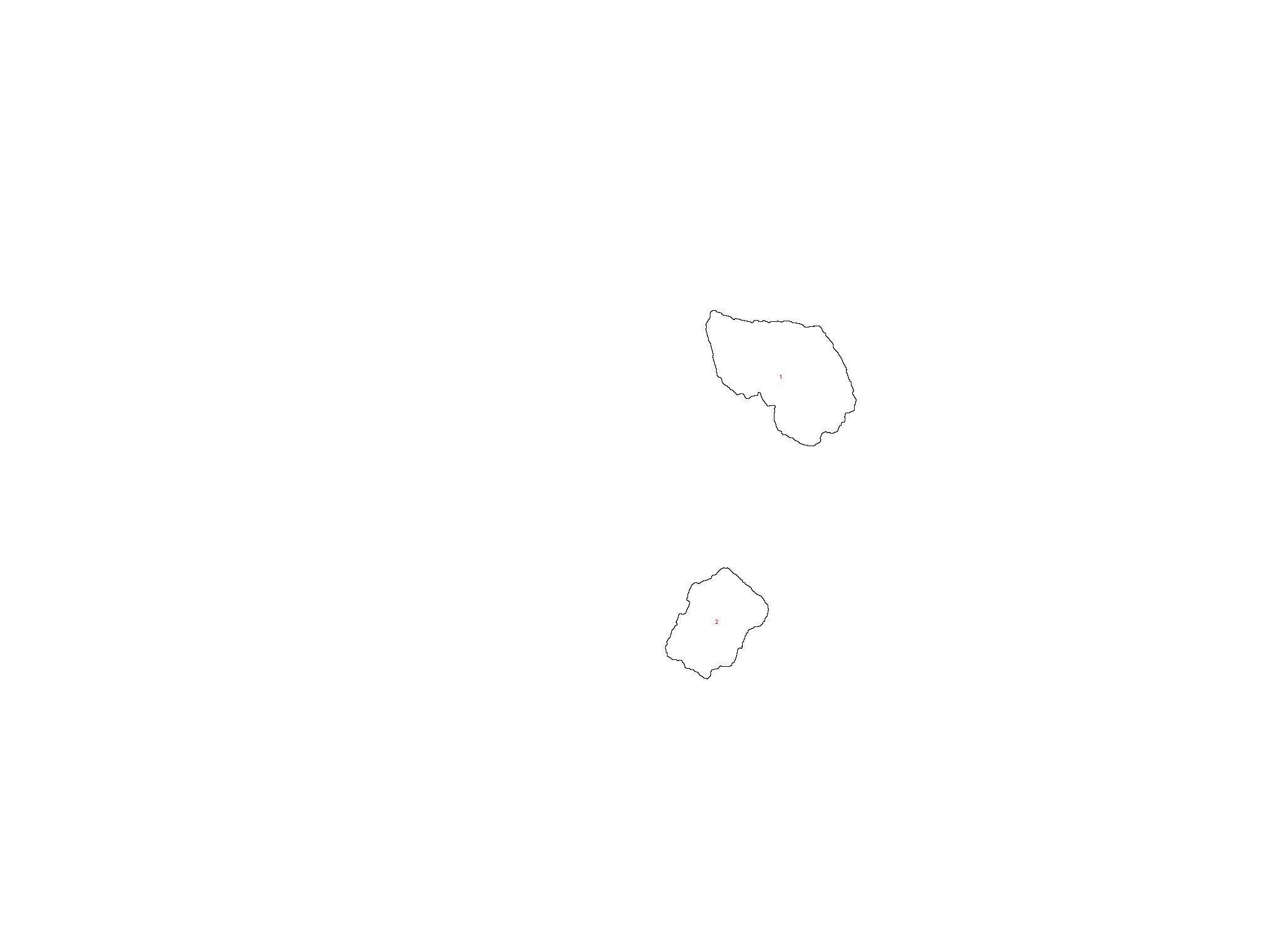

Supplement: S2 Dataset — (ZIP) [file pone.0304198.s005.zip › S2_Dataset_Raw_results_ImageJ/J2_400S_100110_2.jpg]

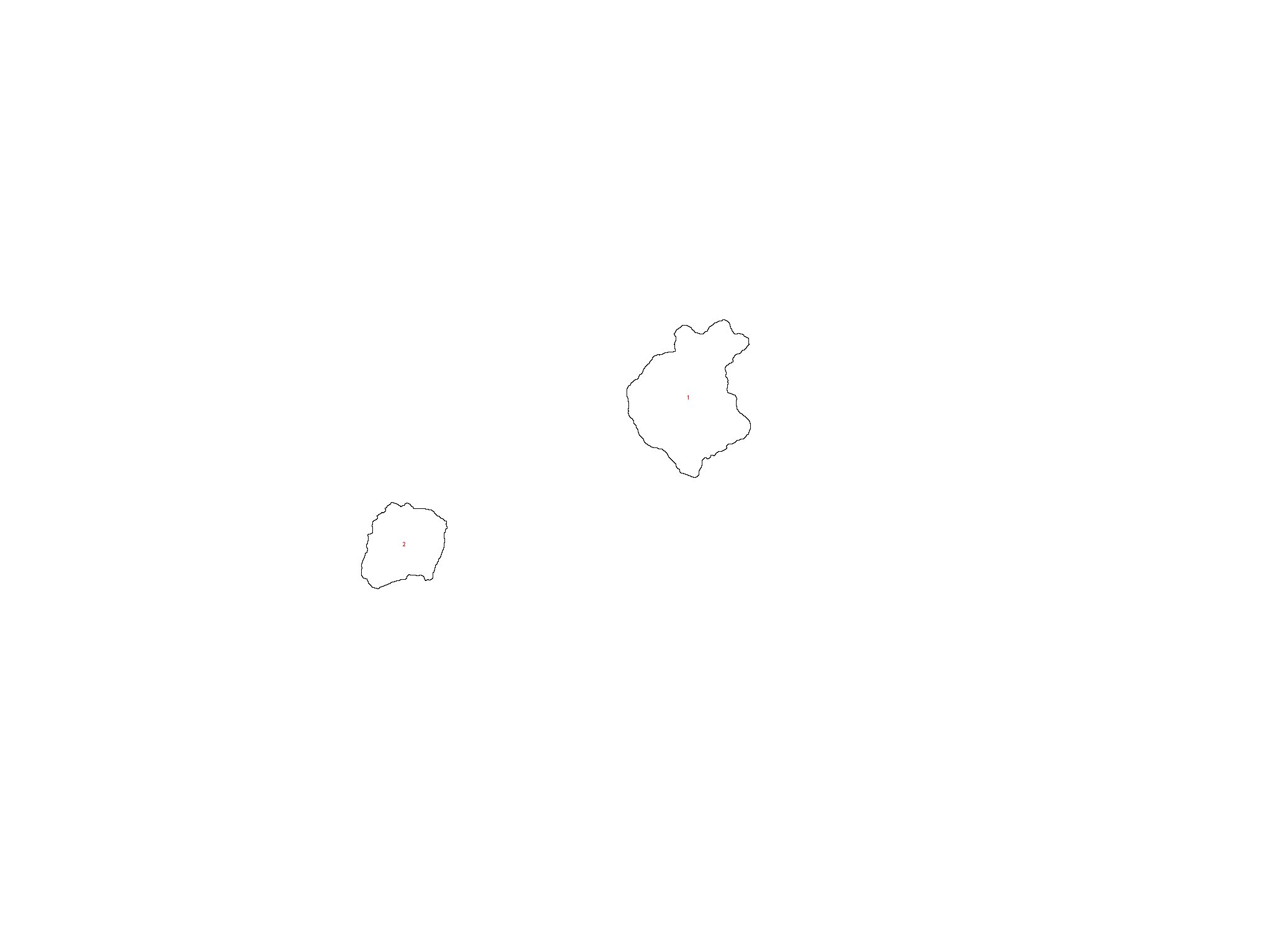

Supplement: S2 Dataset — (ZIP) [file pone.0304198.s005.zip › S2_Dataset_Raw_results_ImageJ/J2_400S_100110_3.jpg]

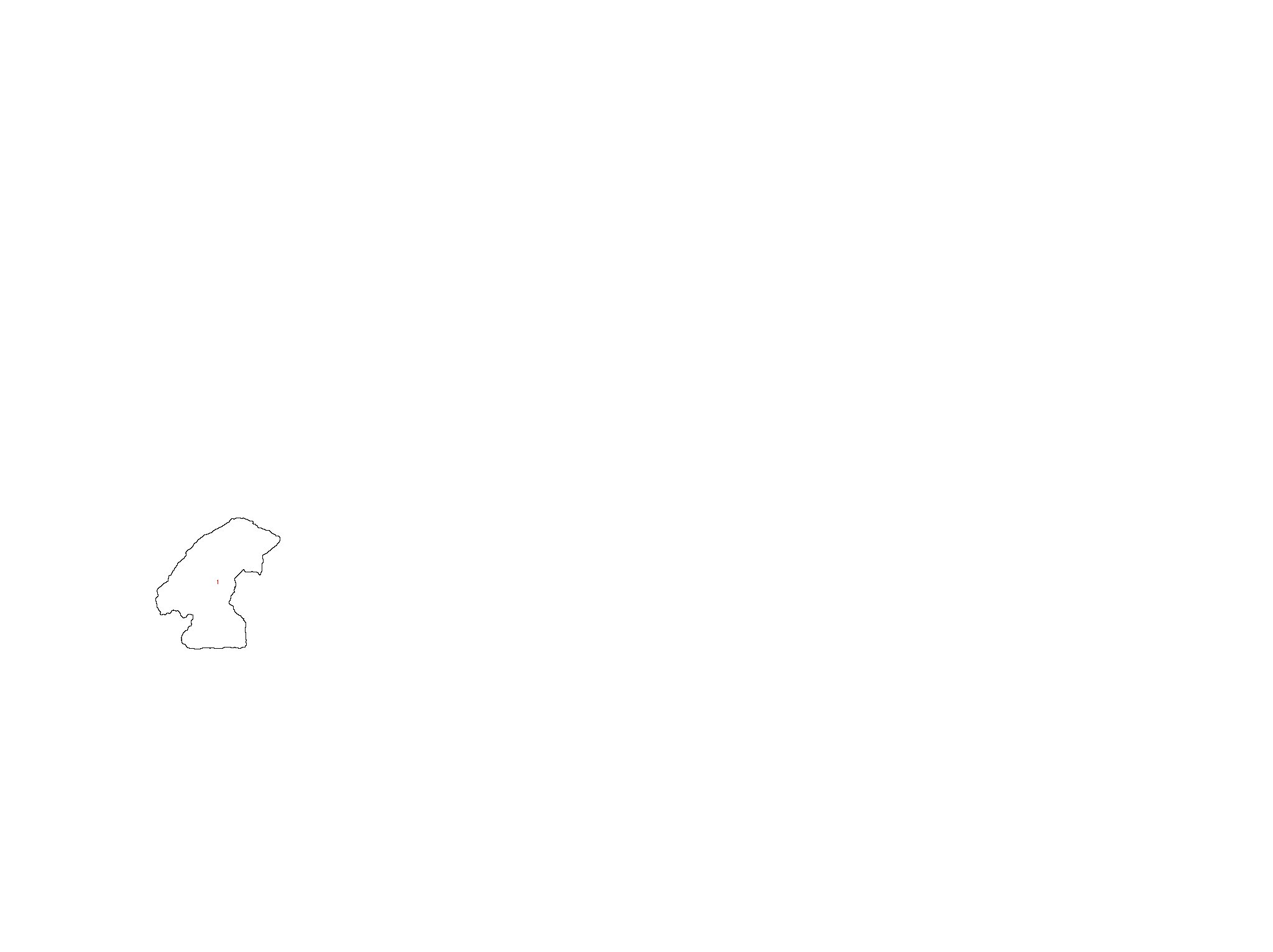

Supplement: S2 Dataset — (ZIP) [file pone.0304198.s005.zip › S2_Dataset_Raw_results_ImageJ/J2_400S_100110_4.jpg]

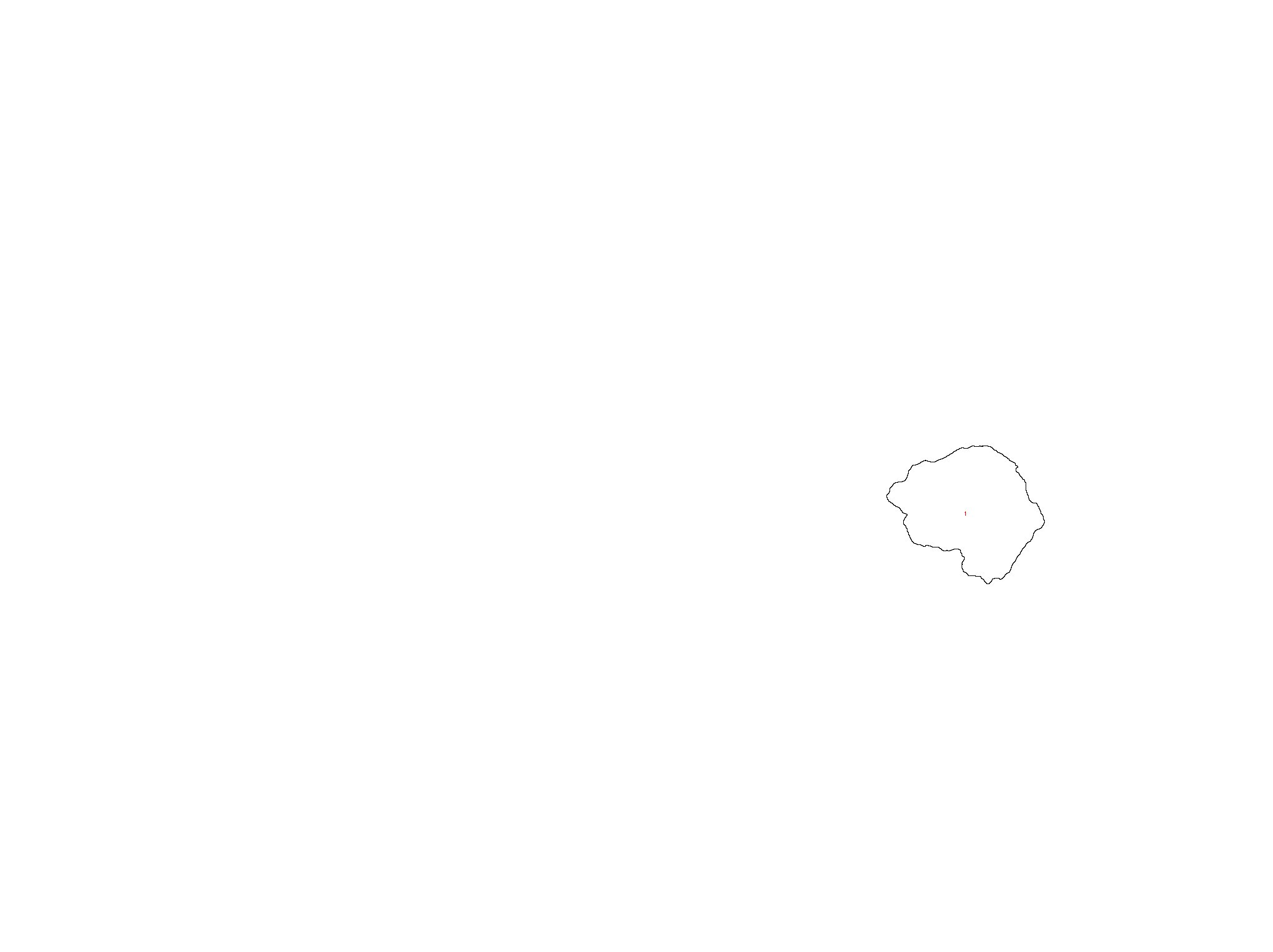

Supplement: S2 Dataset — (ZIP) [file pone.0304198.s005.zip › S2_Dataset_Raw_results_ImageJ/J2_400S_100110_5.jpg]

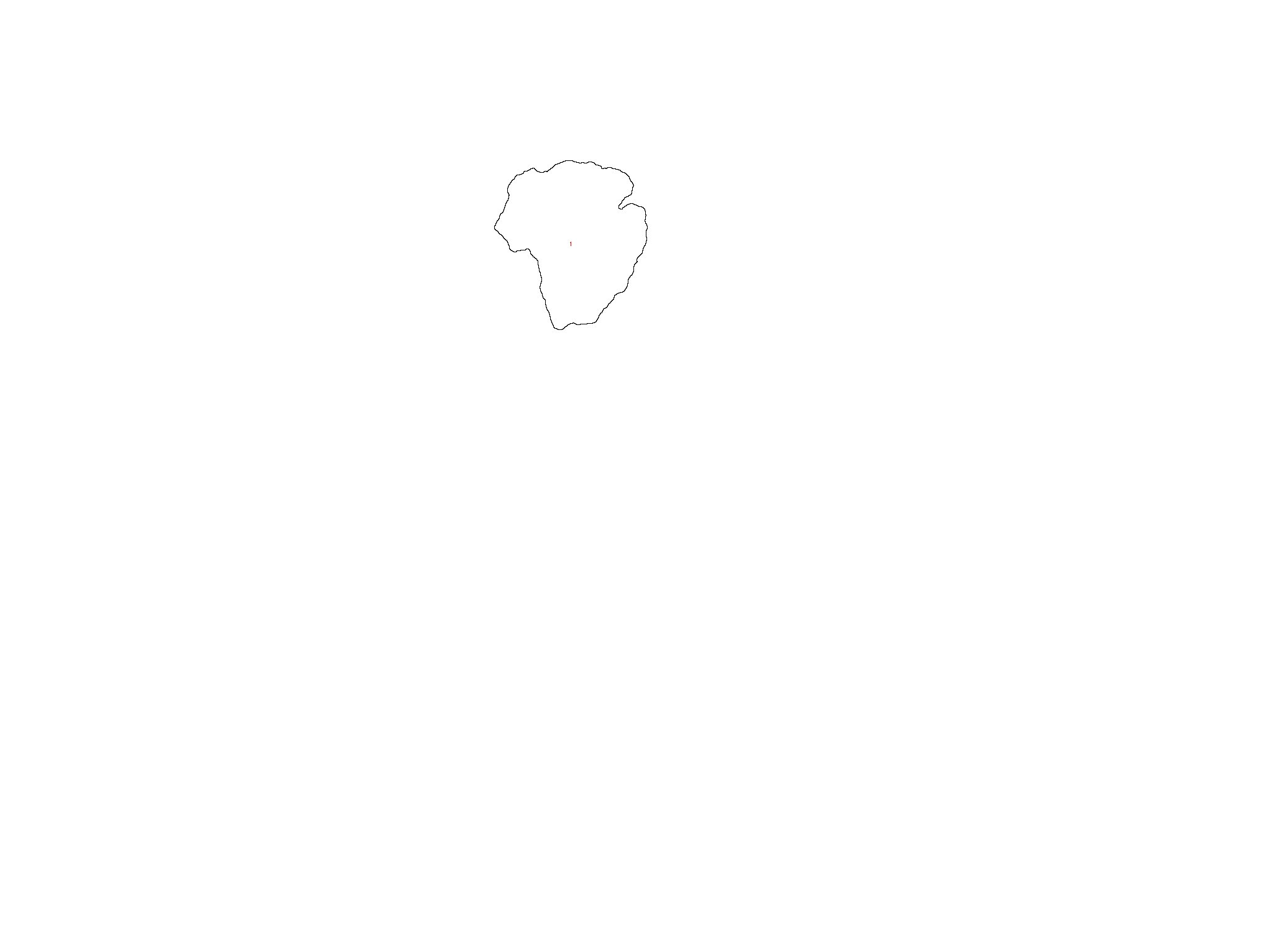

Supplement: S2 Dataset — (ZIP) [file pone.0304198.s005.zip › S2_Dataset_Raw_results_ImageJ/J2_400S_100110_6.jpg]

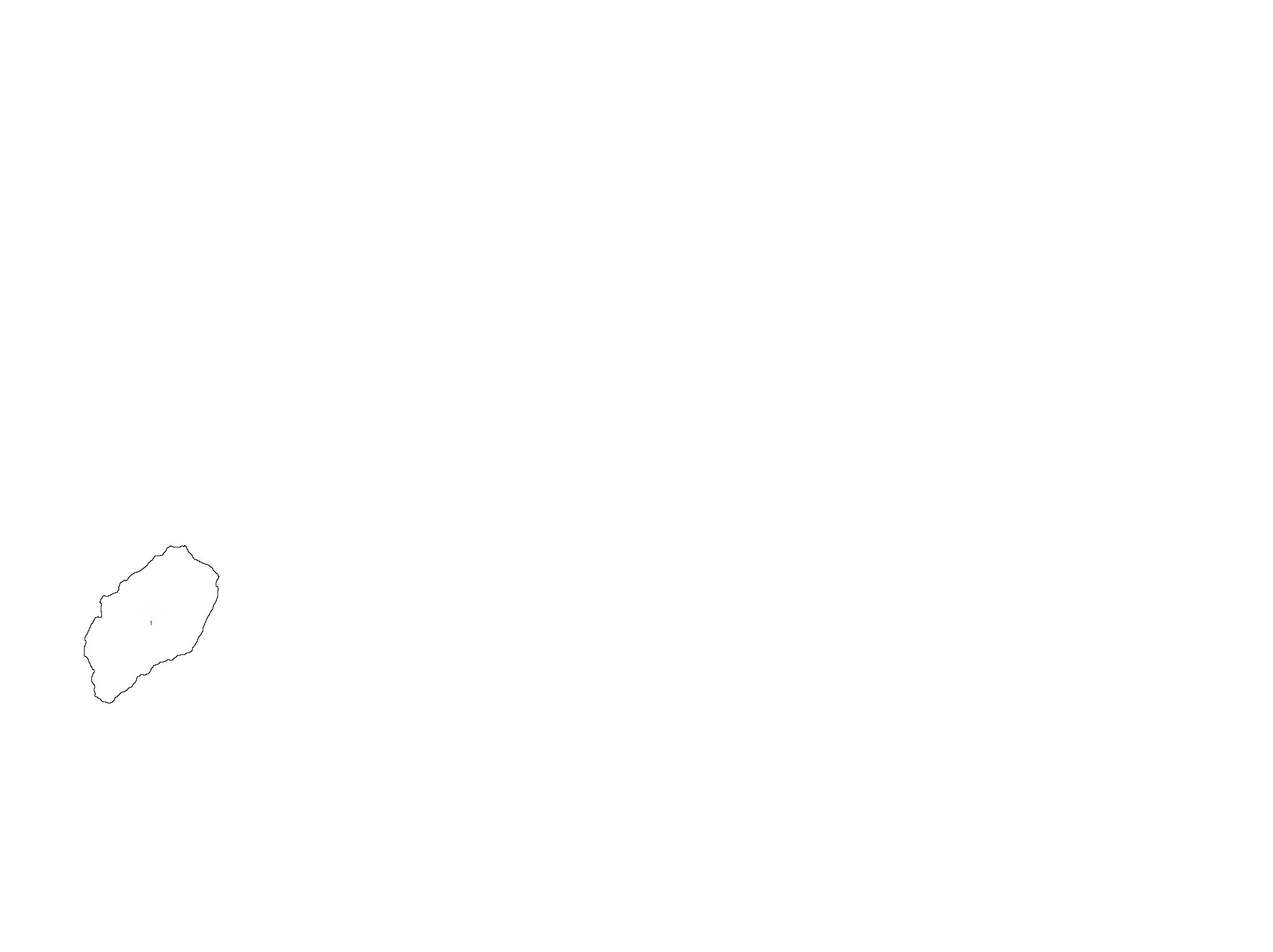

Supplement: S2 Dataset — (ZIP) [file pone.0304198.s005.zip › S2_Dataset_Raw_results_ImageJ/J2_400S_140150_1.jpg]

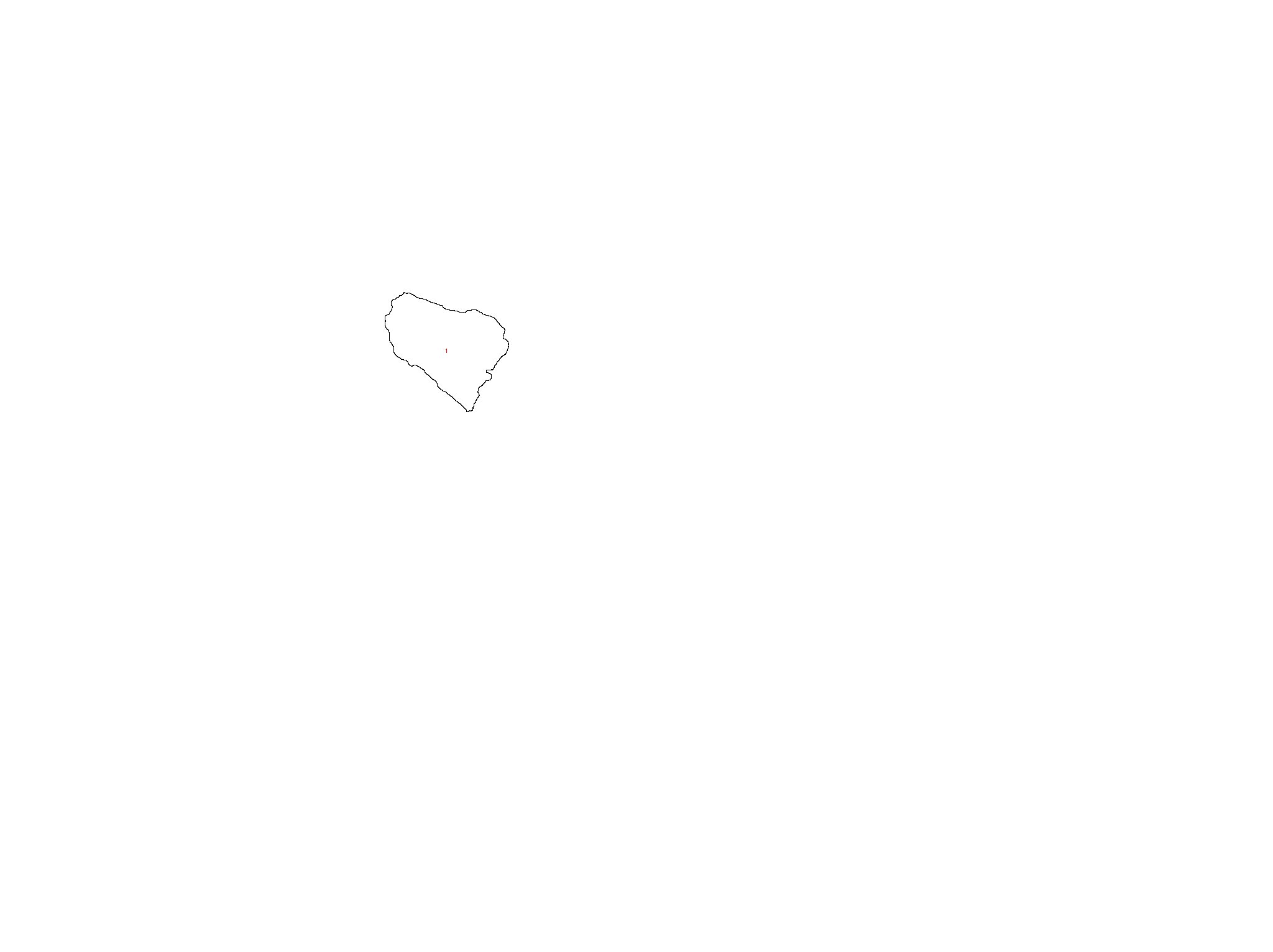

Supplement: S2 Dataset — (ZIP) [file pone.0304198.s005.zip › S2_Dataset_Raw_results_ImageJ/J2_400S_140150_3.jpg]

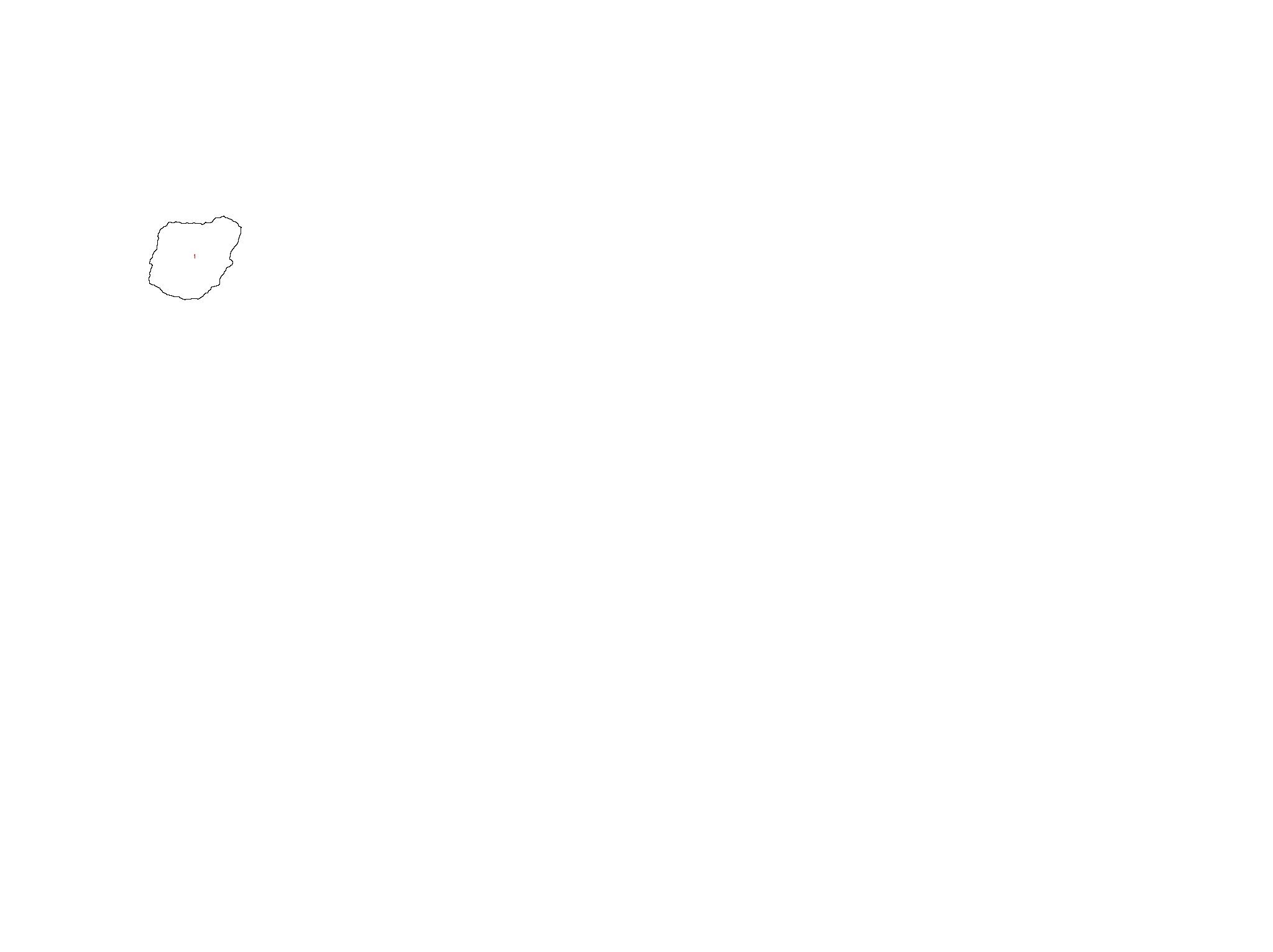

Supplement: S2 Dataset — (ZIP) [file pone.0304198.s005.zip › S2_Dataset_Raw_results_ImageJ/J2_400S_140150_4.jpg]

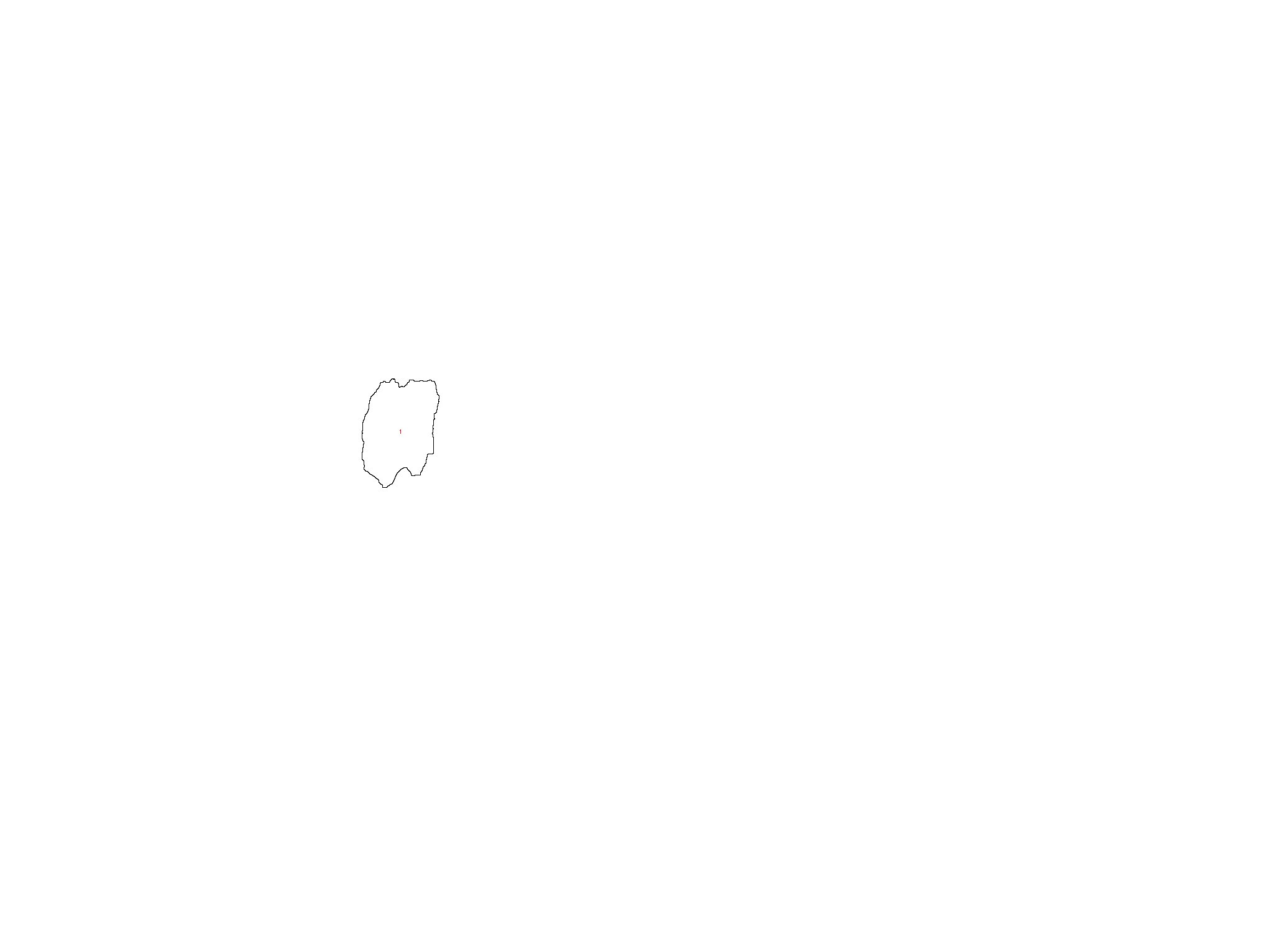

Supplement: S2 Dataset — (ZIP) [file pone.0304198.s005.zip › S2_Dataset_Raw_results_ImageJ/J2_400S_190200_1.jpg]

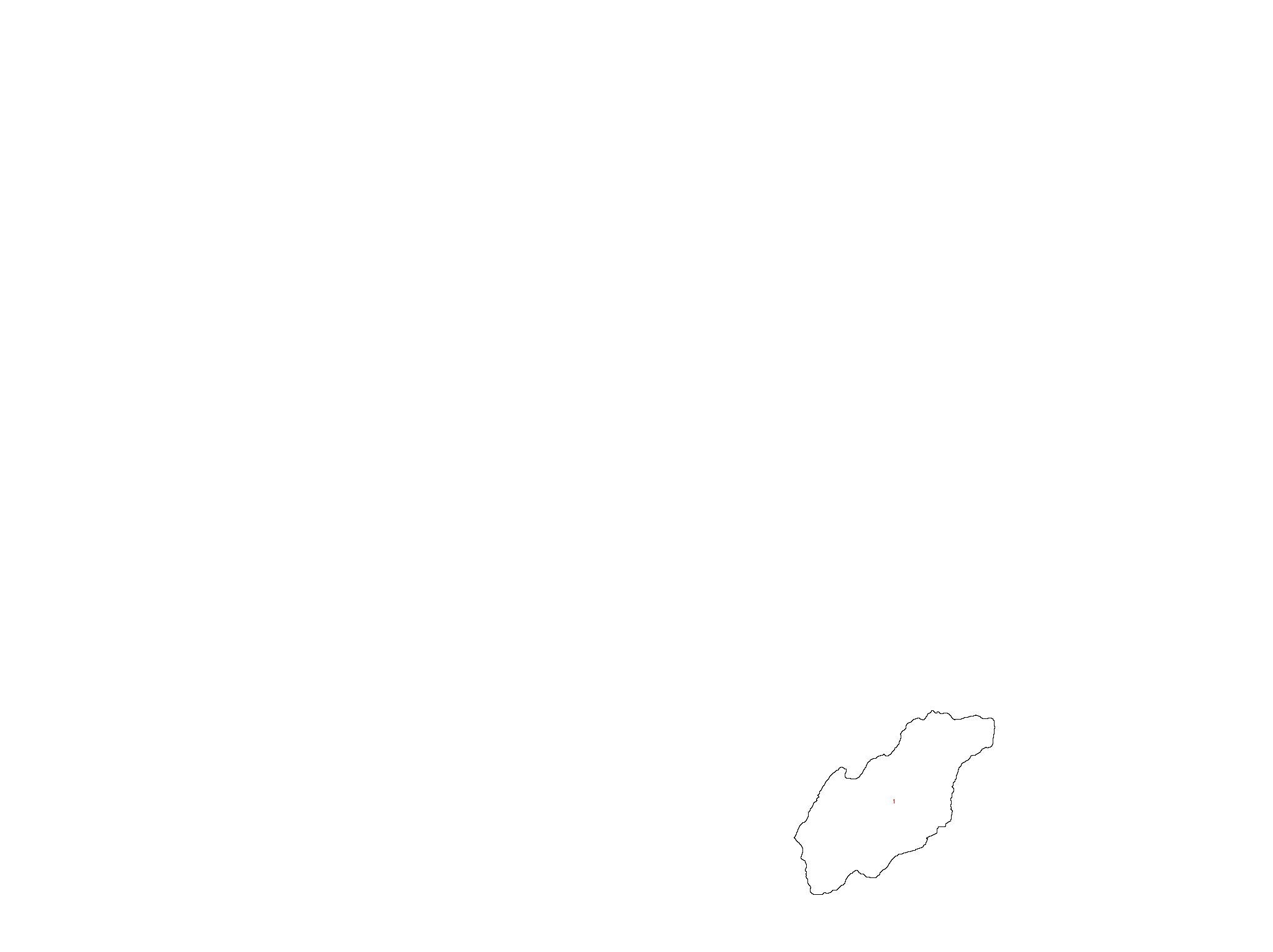

Supplement: S2 Dataset — (ZIP) [file pone.0304198.s005.zip › S2_Dataset_Raw_results_ImageJ/J2_400S_190200_2.jpg]

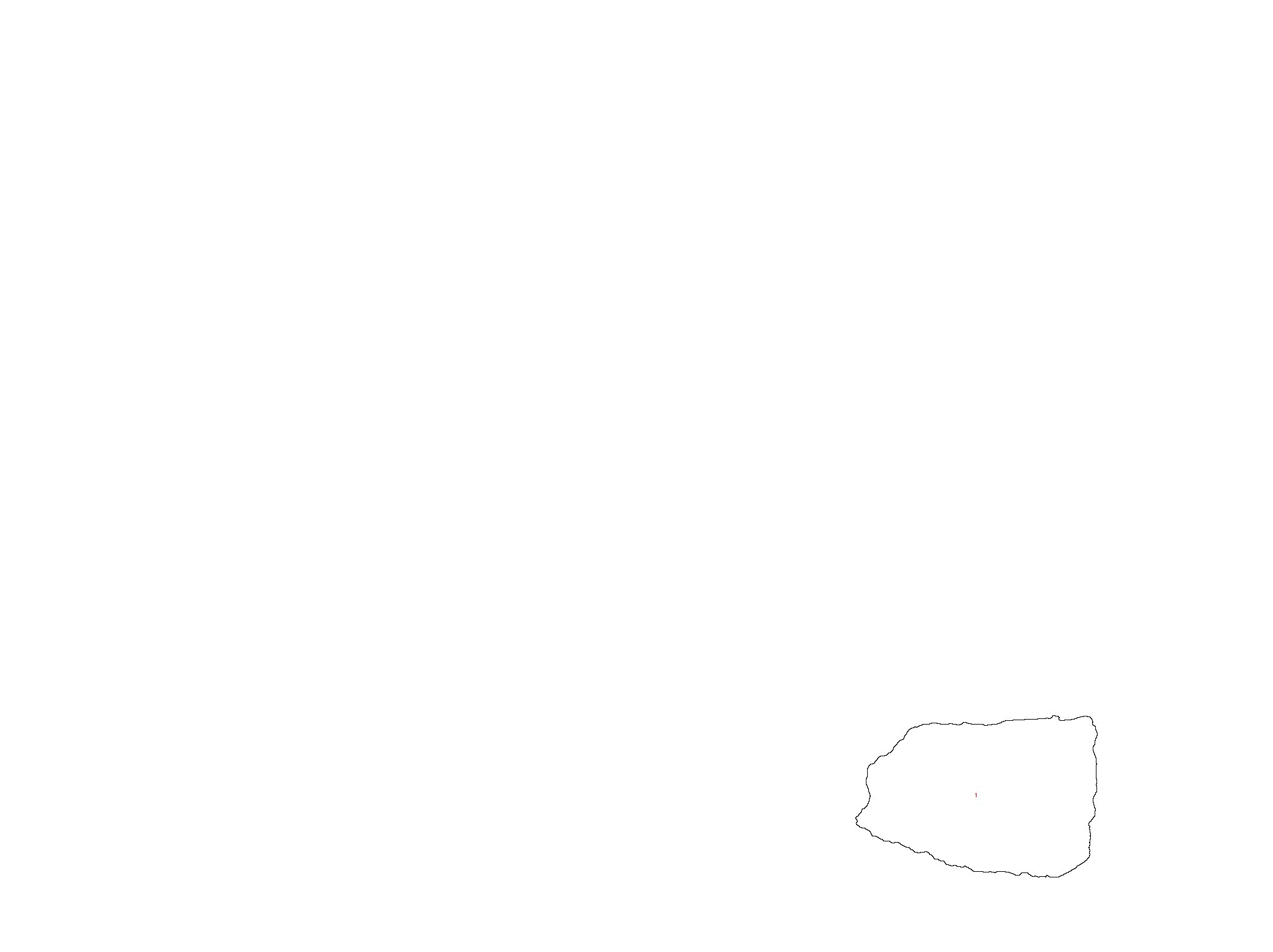

Supplement: S2 Dataset — (ZIP) [file pone.0304198.s005.zip › S2_Dataset_Raw_results_ImageJ/J2_400S_6070_1.jpg]

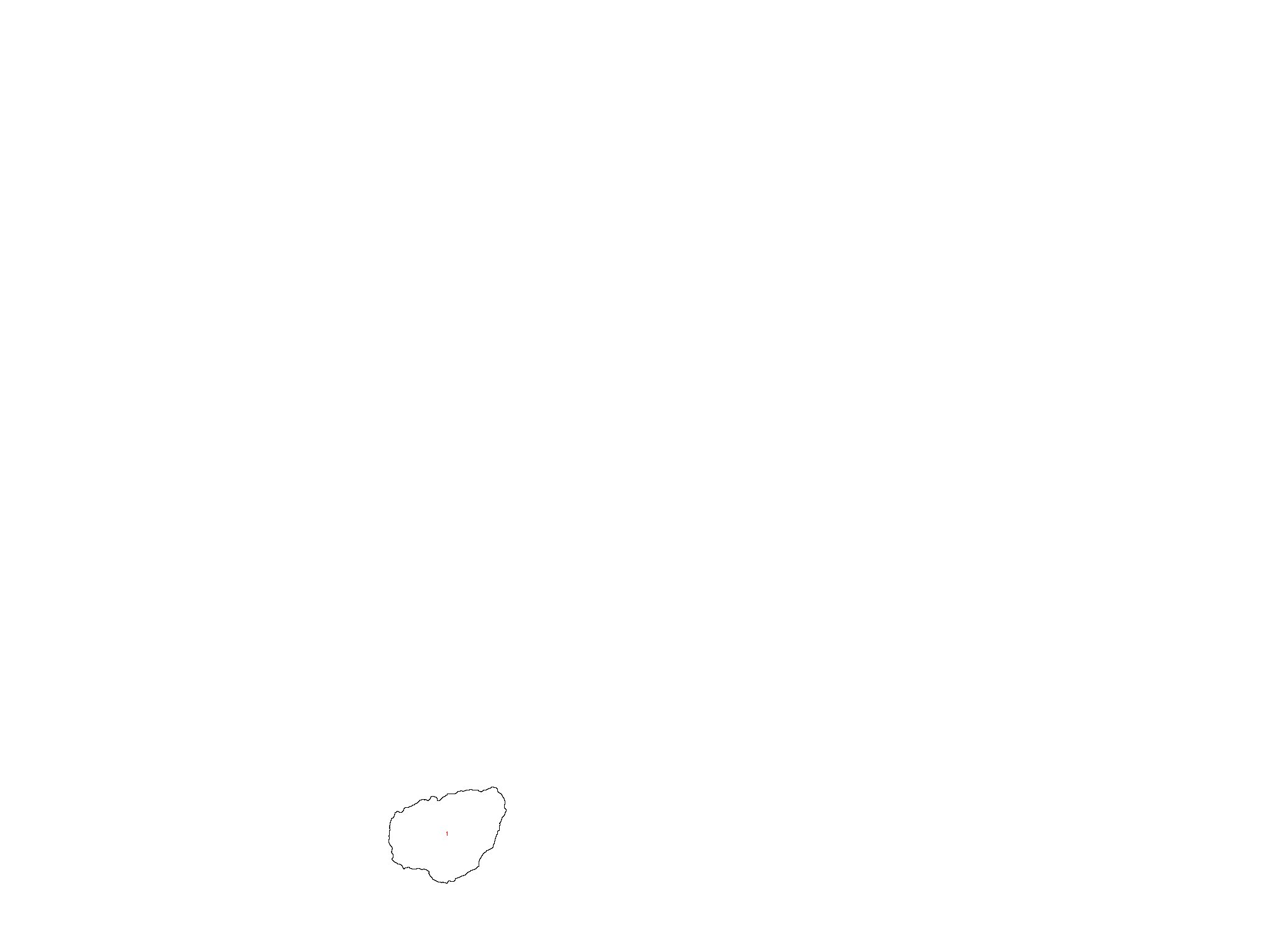

Supplement: S2 Dataset — (ZIP) [file pone.0304198.s005.zip › S2_Dataset_Raw_results_ImageJ/J2_400S_6070_2.jpg]

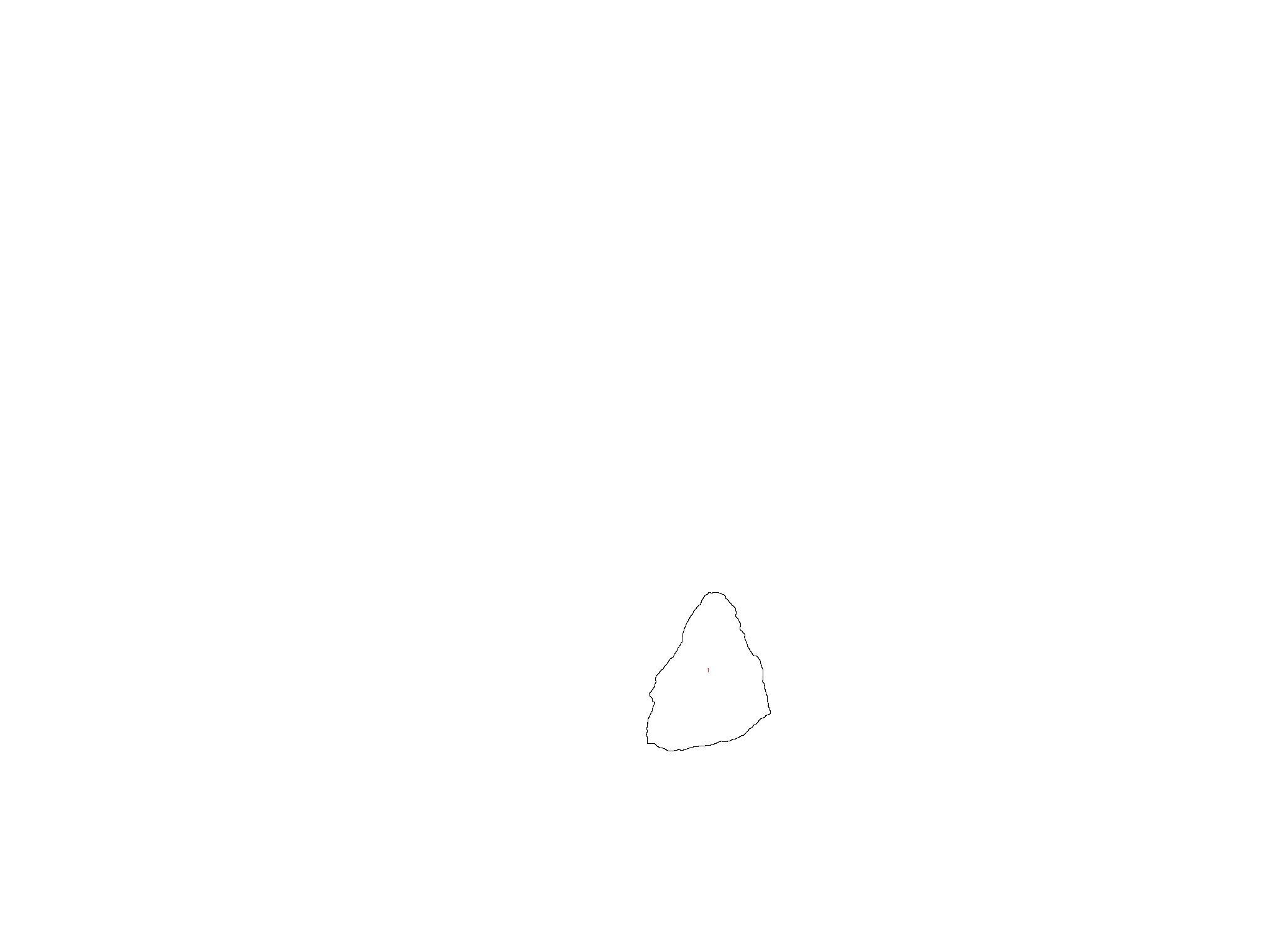

Supplement: S2 Dataset — (ZIP) [file pone.0304198.s005.zip › S2_Dataset_Raw_results_ImageJ/J2_400S_90100_1.jpg]

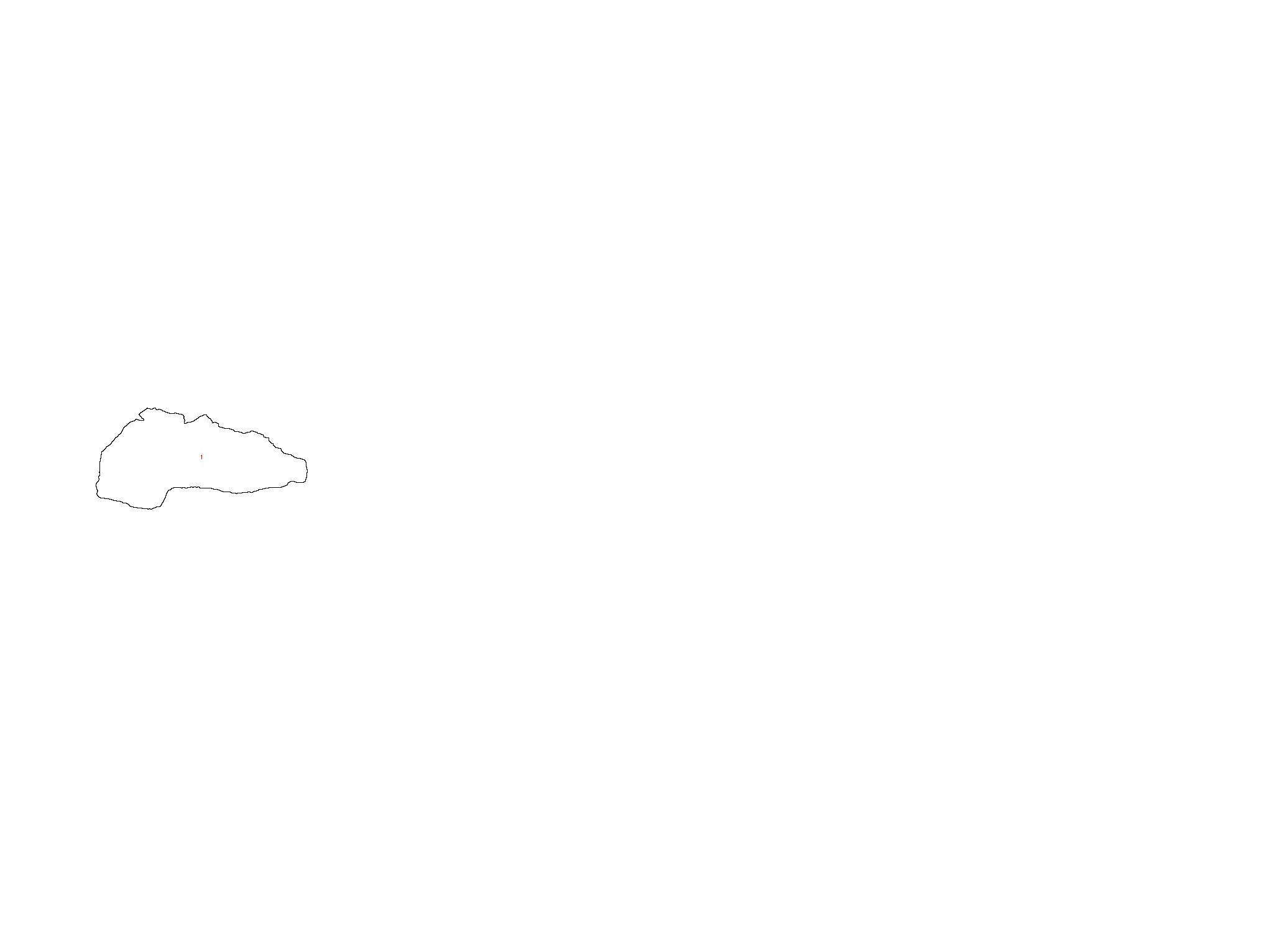

Supplement: S2 Dataset — (ZIP) [file pone.0304198.s005.zip › S2_Dataset_Raw_results_ImageJ/J2_400S_90100_2.jpg]

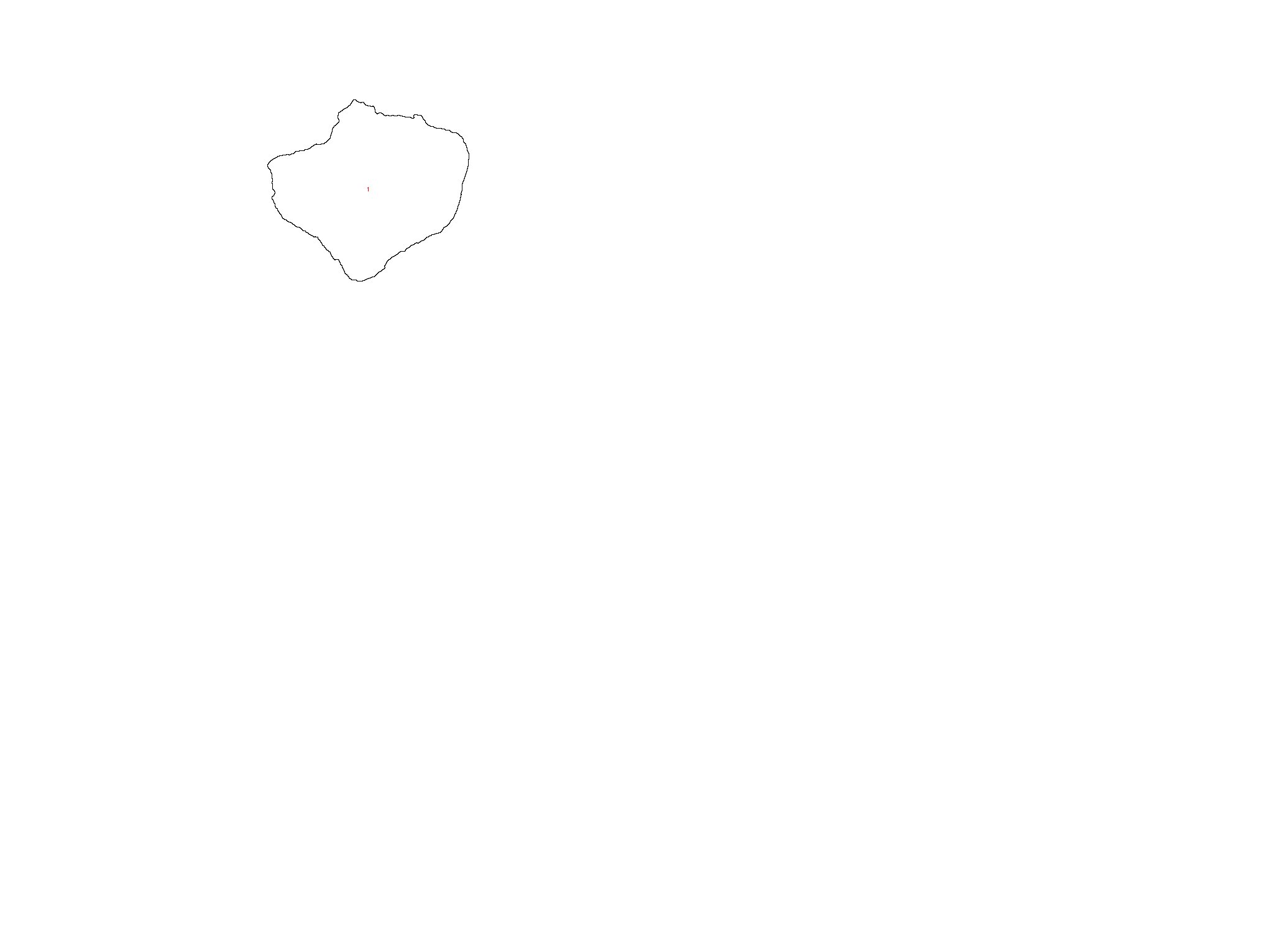

Supplement: S2 Dataset — (ZIP) [file pone.0304198.s005.zip › S2_Dataset_Raw_results_ImageJ/J2_400S_90100_3.jpg]

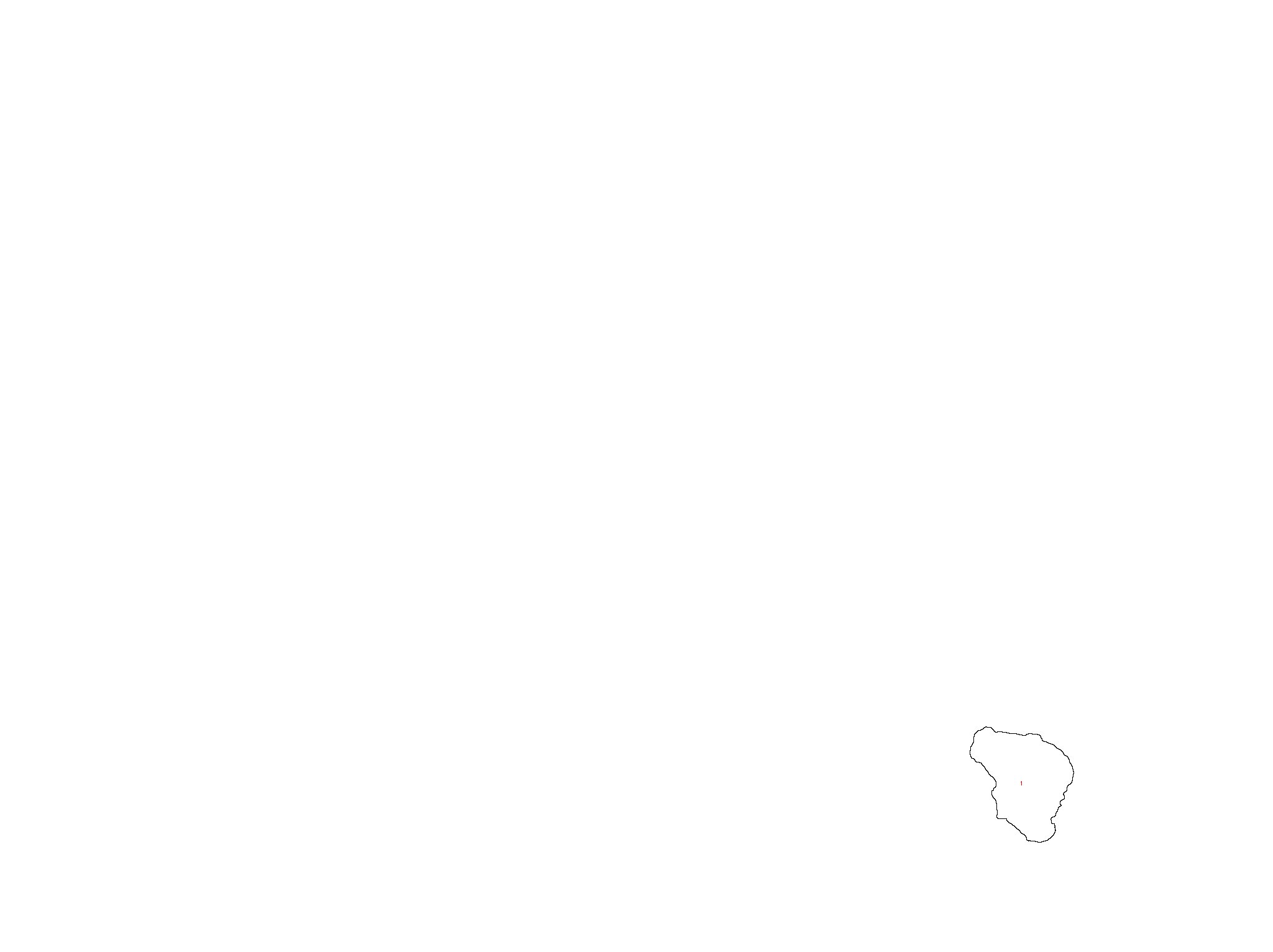

Supplement: S2 Dataset — (ZIP) [file pone.0304198.s005.zip › S2_Dataset_Raw_results_ImageJ/J7_0E_110120_1.jpg]

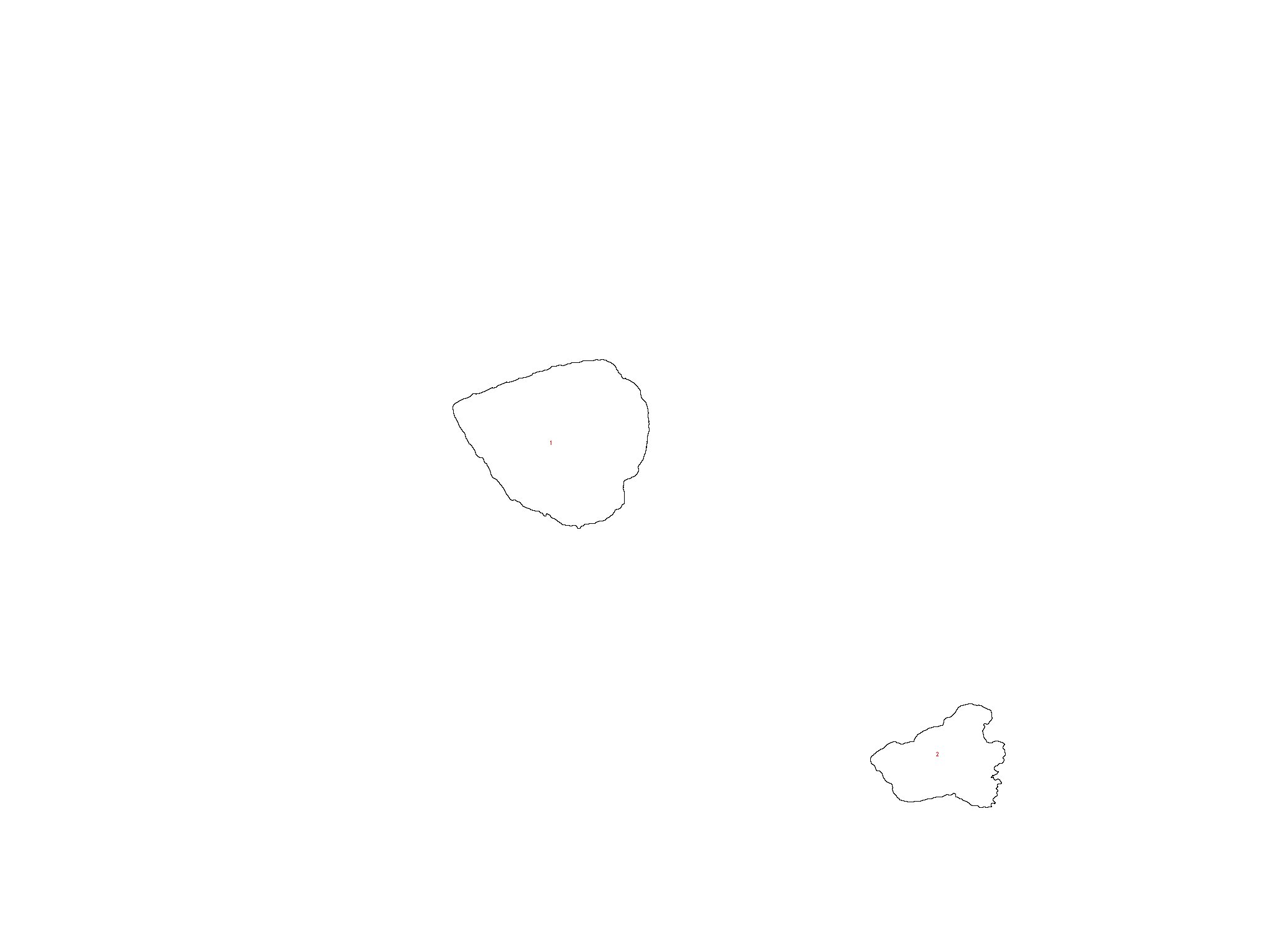

Supplement: S2 Dataset — (ZIP) [file pone.0304198.s005.zip › S2_Dataset_Raw_results_ImageJ/J7_0E_110120_2.jpg]

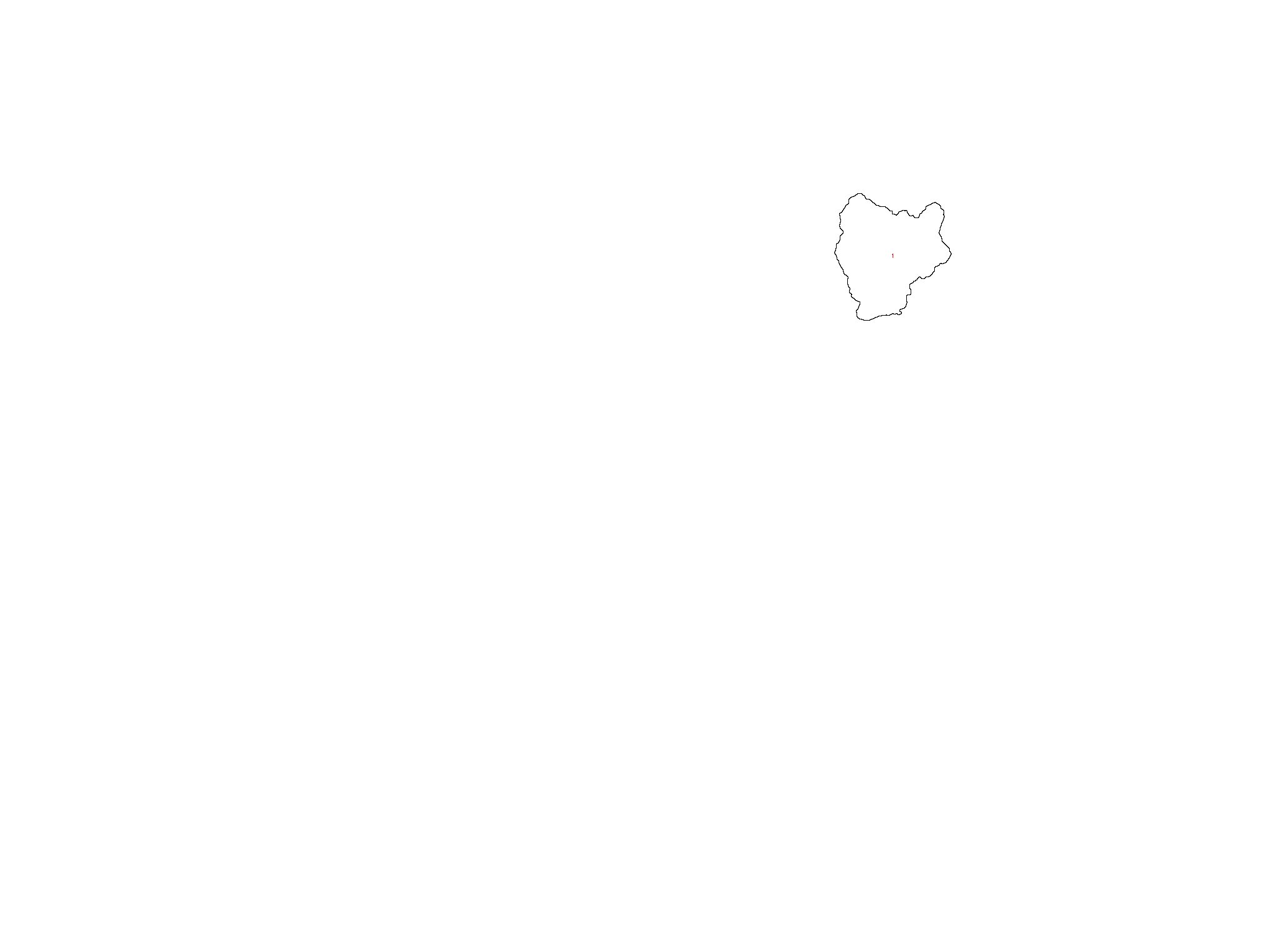

Supplement: S2 Dataset — (ZIP) [file pone.0304198.s005.zip › S2_Dataset_Raw_results_ImageJ/J7_0E_110120_3.jpg]

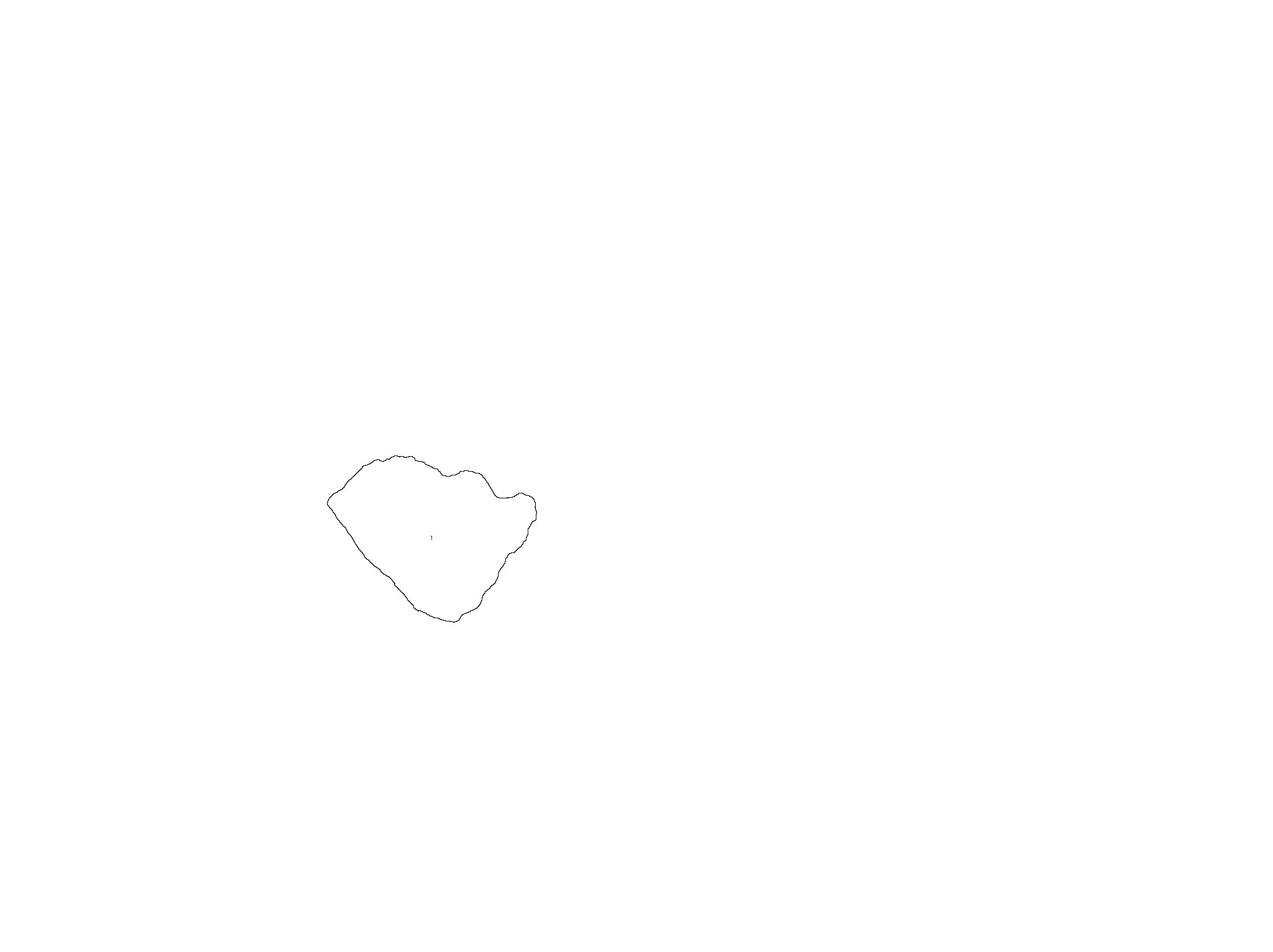

Supplement: S2 Dataset — (ZIP) [file pone.0304198.s005.zip › S2_Dataset_Raw_results_ImageJ/J7_0E_120130_1.jpg]

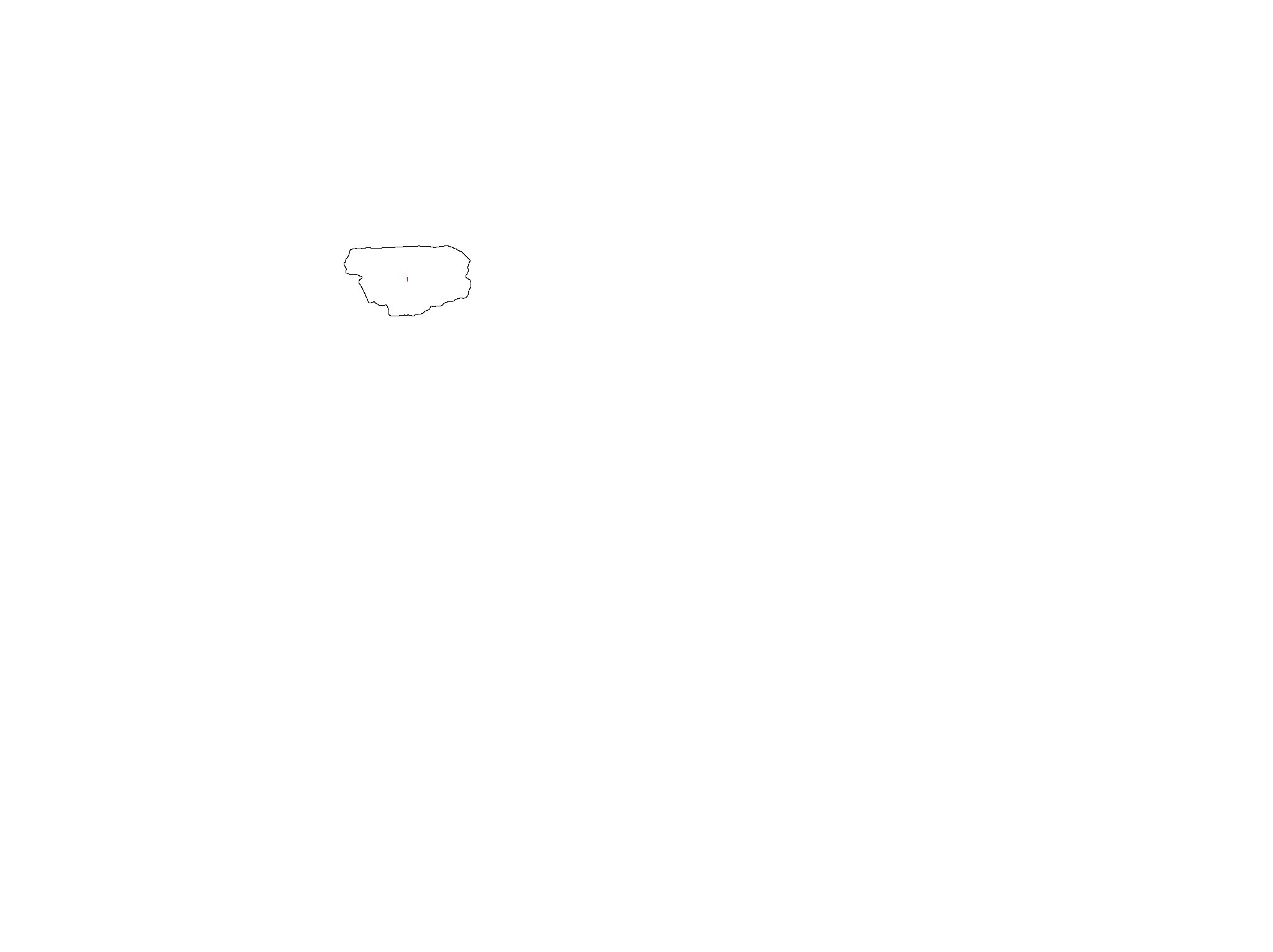

Supplement: S2 Dataset — (ZIP) [file pone.0304198.s005.zip › S2_Dataset_Raw_results_ImageJ/J7_0E_120130_2.jpg]

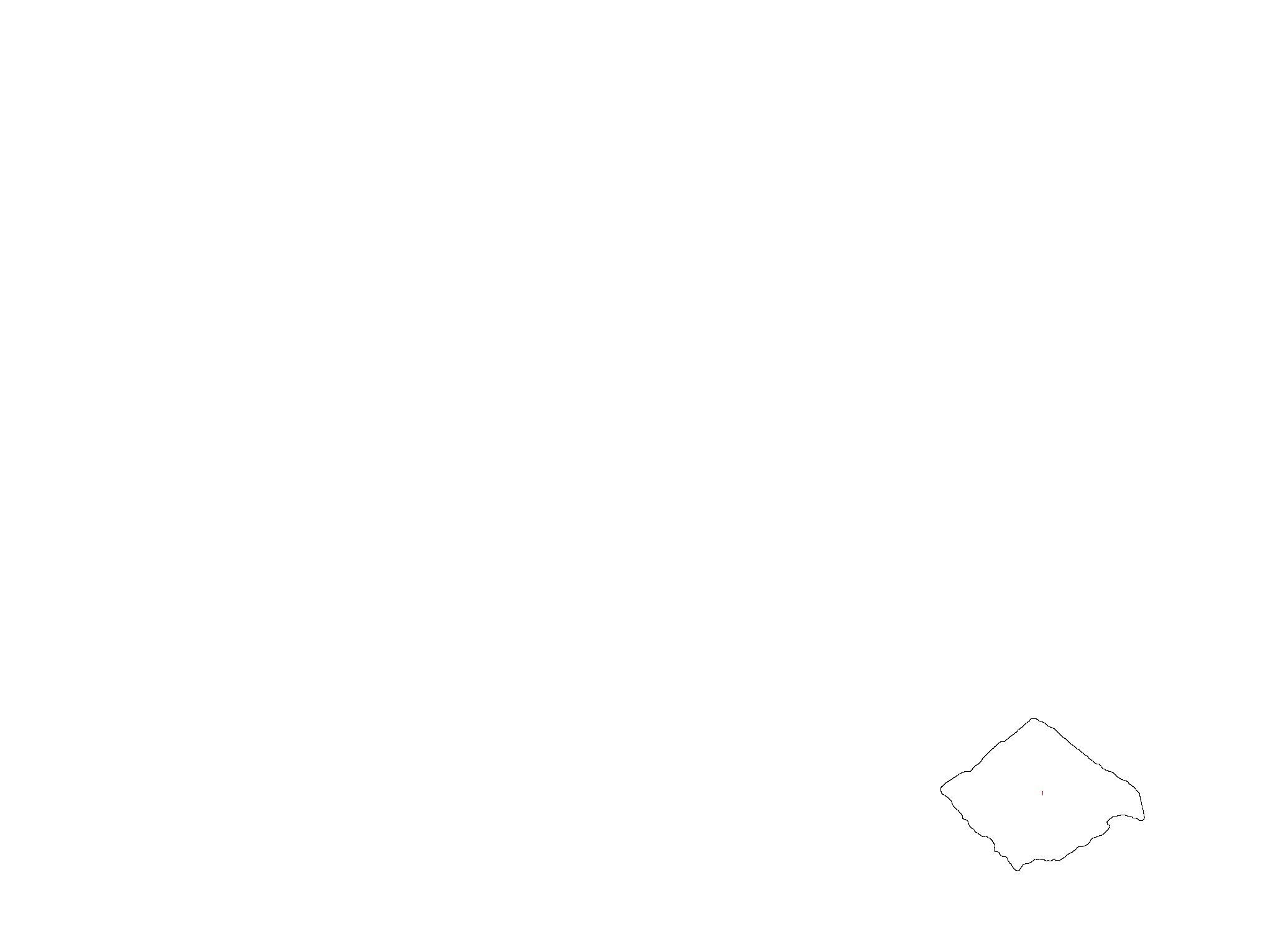

Supplement: S2 Dataset — (ZIP) [file pone.0304198.s005.zip › S2_Dataset_Raw_results_ImageJ/J7_0E_4050_1.jpg]

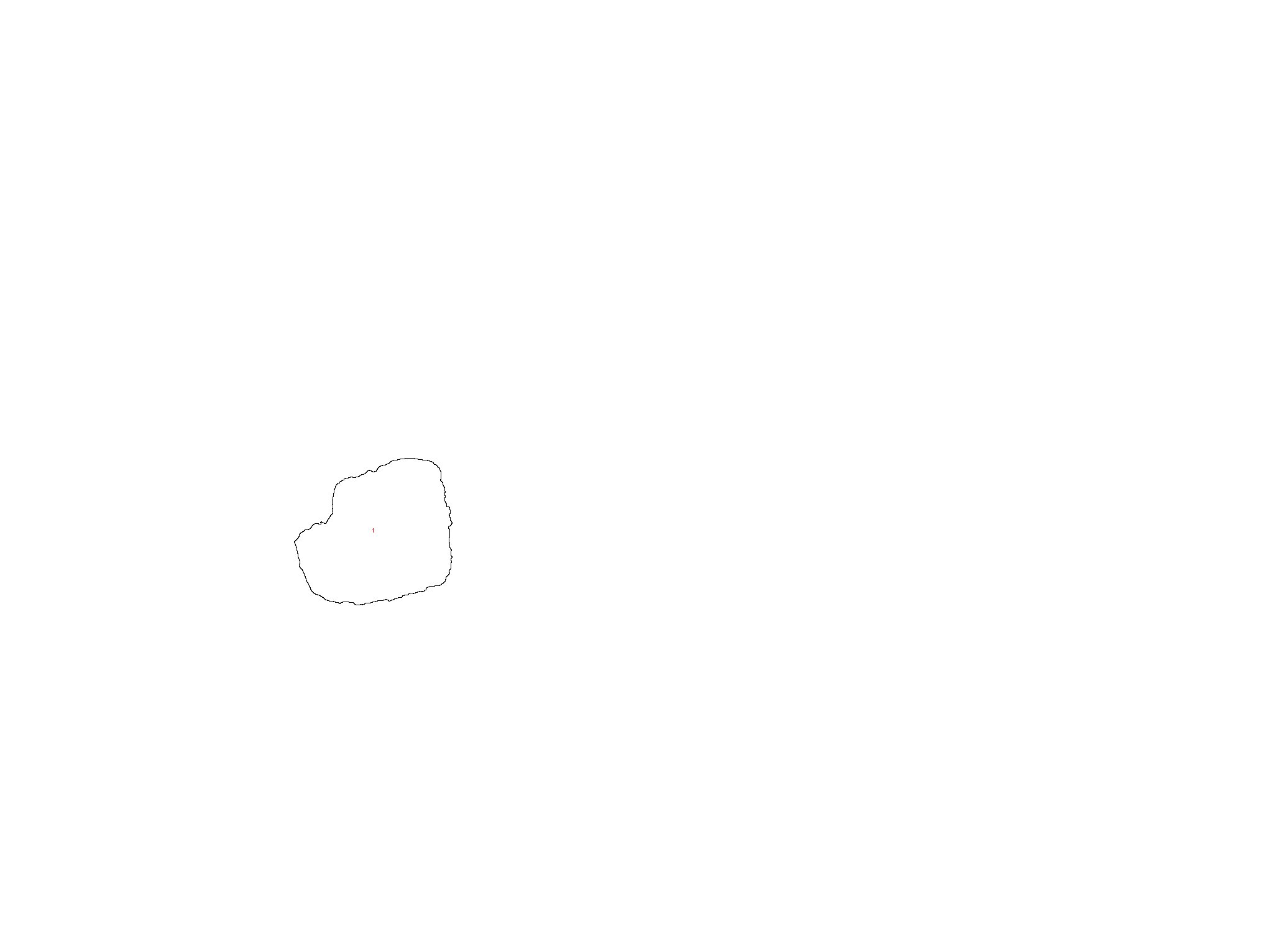

Supplement: S2 Dataset — (ZIP) [file pone.0304198.s005.zip › S2_Dataset_Raw_results_ImageJ/J7_0E_4050_2.jpg]

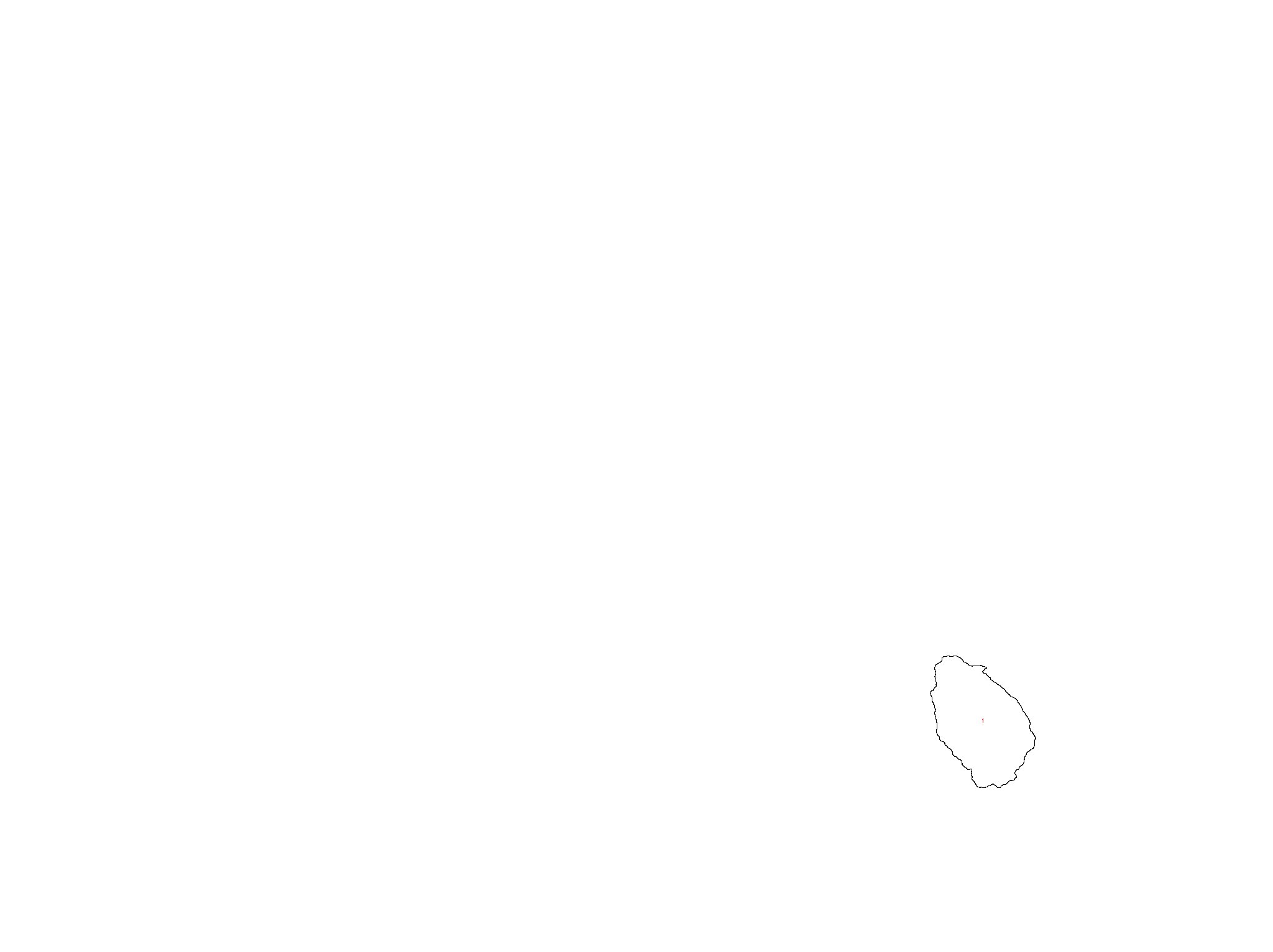

Supplement: S2 Dataset — (ZIP) [file pone.0304198.s005.zip › S2_Dataset_Raw_results_ImageJ/J7_0E_4050_3.jpg]

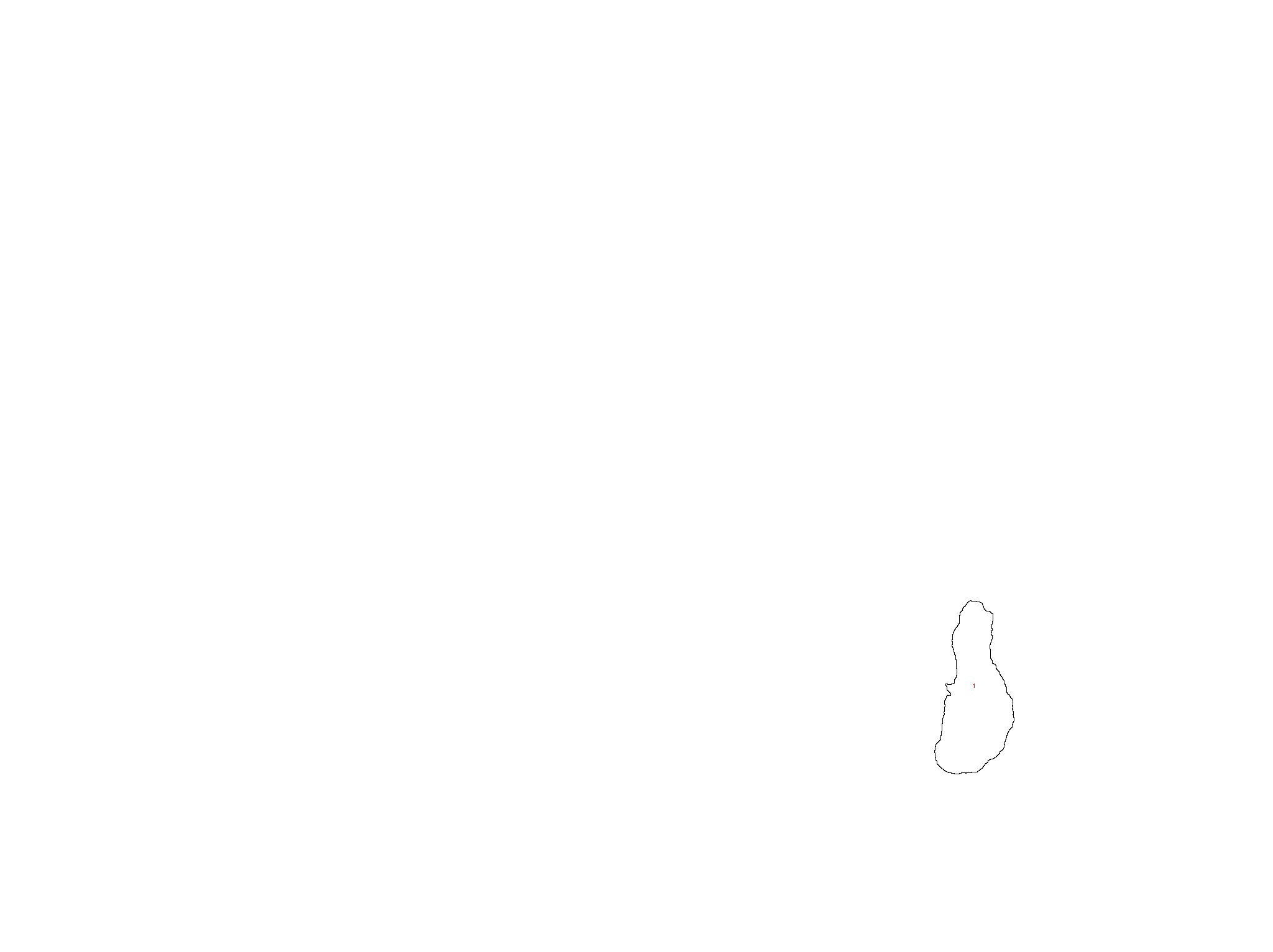

Supplement: S2 Dataset — (ZIP) [file pone.0304198.s005.zip › S2_Dataset_Raw_results_ImageJ/J7_0E_4050_4.jpg]

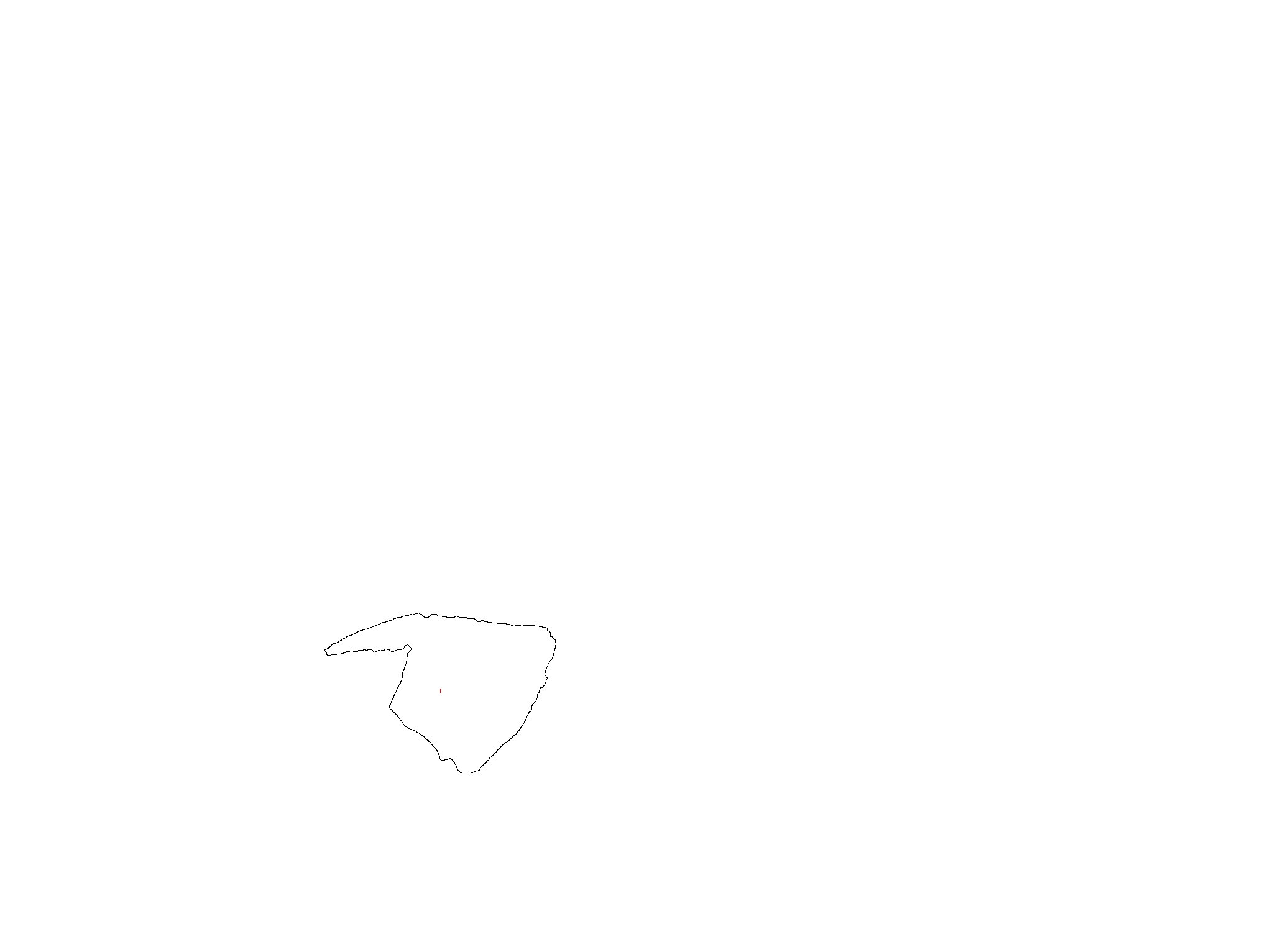

Supplement: S2 Dataset — (ZIP) [file pone.0304198.s005.zip › S2_Dataset_Raw_results_ImageJ/J7_0E_4050_5.jpg]

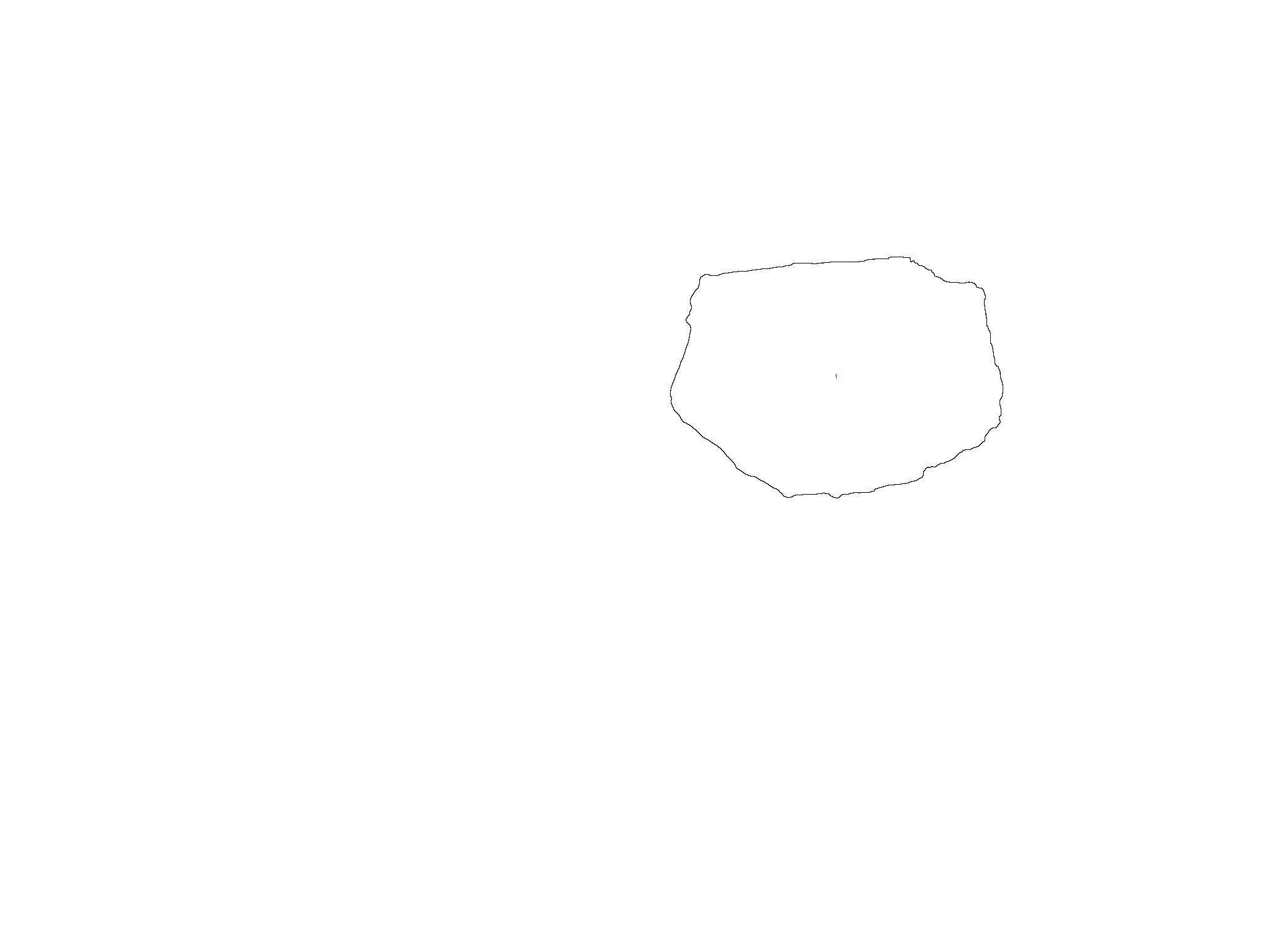

Supplement: S2 Dataset — (ZIP) [file pone.0304198.s005.zip › S2_Dataset_Raw_results_ImageJ/J7_0E_4050_6.jpg]

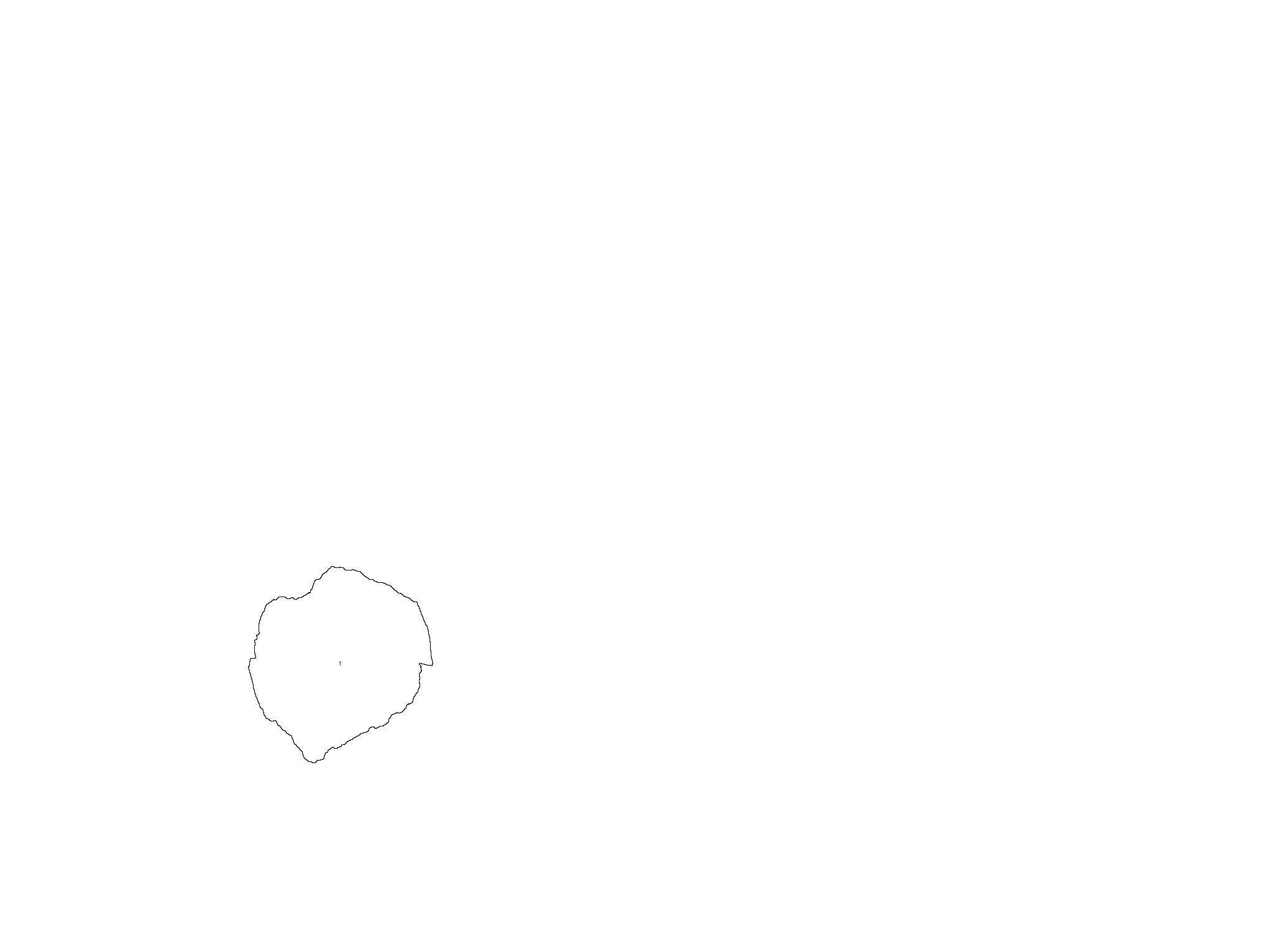

Supplement: S2 Dataset — (ZIP) [file pone.0304198.s005.zip › S2_Dataset_Raw_results_ImageJ/J7_0E_90100_1.jpg]

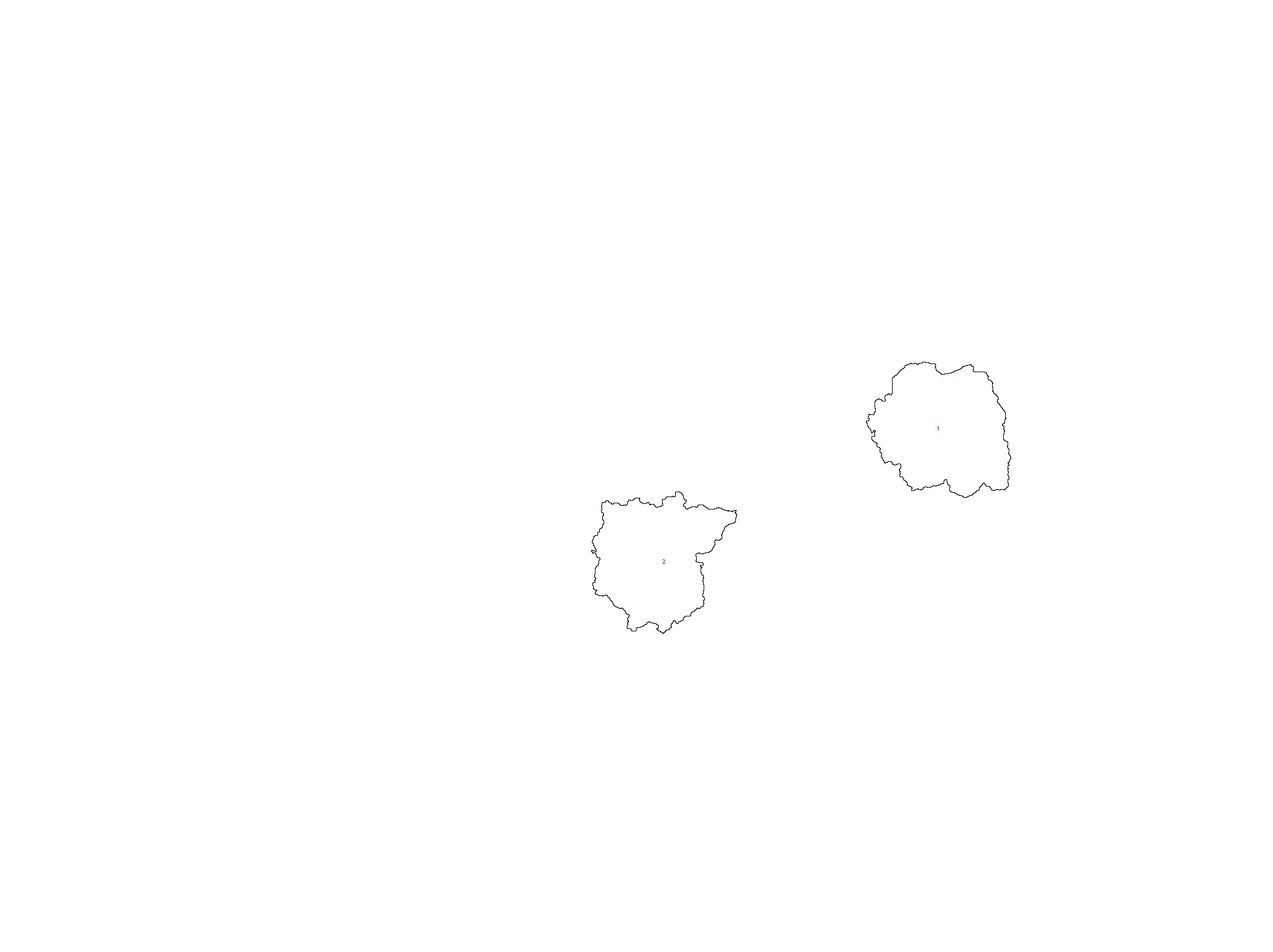

Supplement: S2 Dataset — (ZIP) [file pone.0304198.s005.zip › S2_Dataset_Raw_results_ImageJ/J7_100F_010_1.jpg]

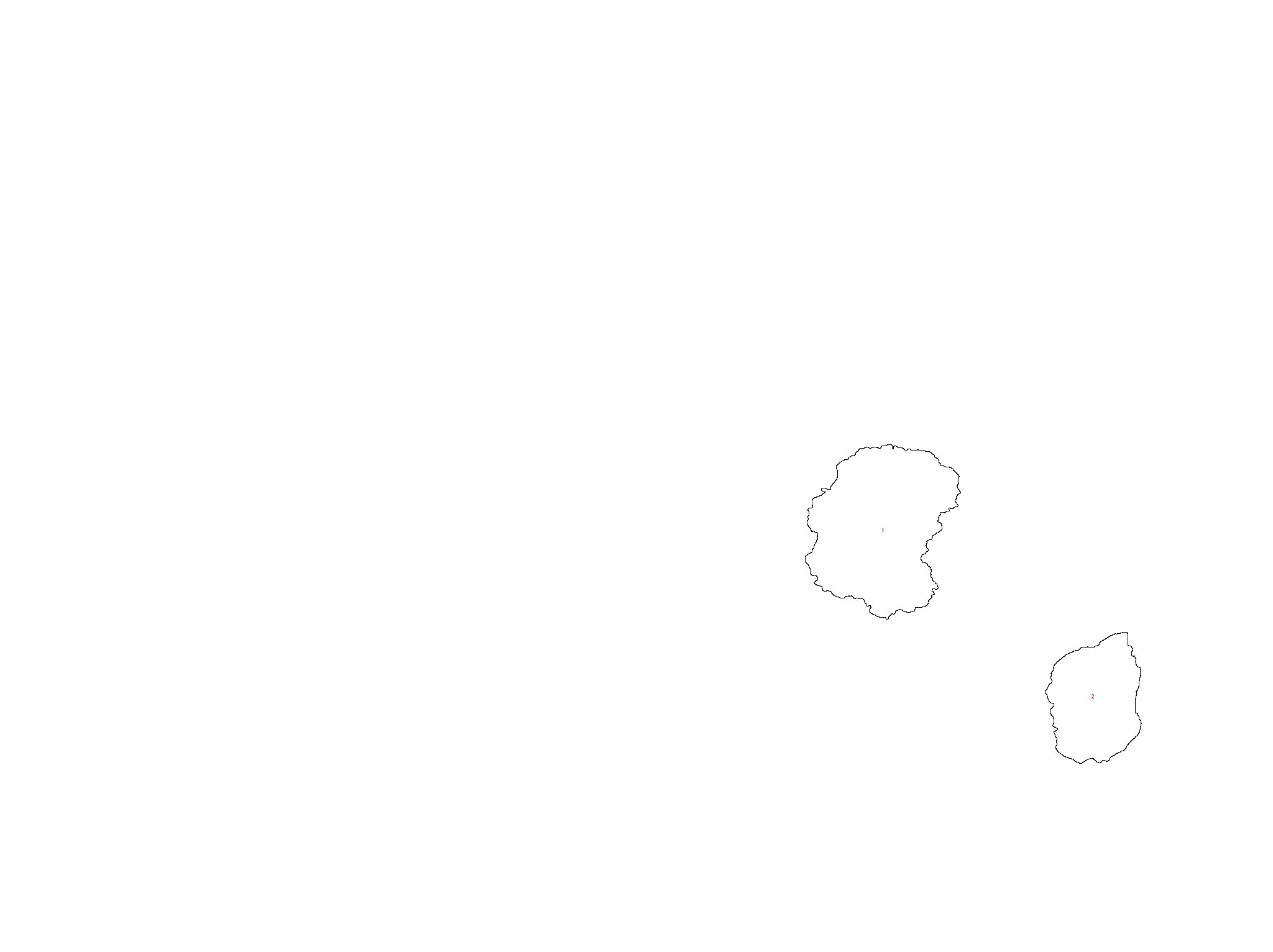

Supplement: S2 Dataset — (ZIP) [file pone.0304198.s005.zip › S2_Dataset_Raw_results_ImageJ/J7_100F_010_10.jpg]

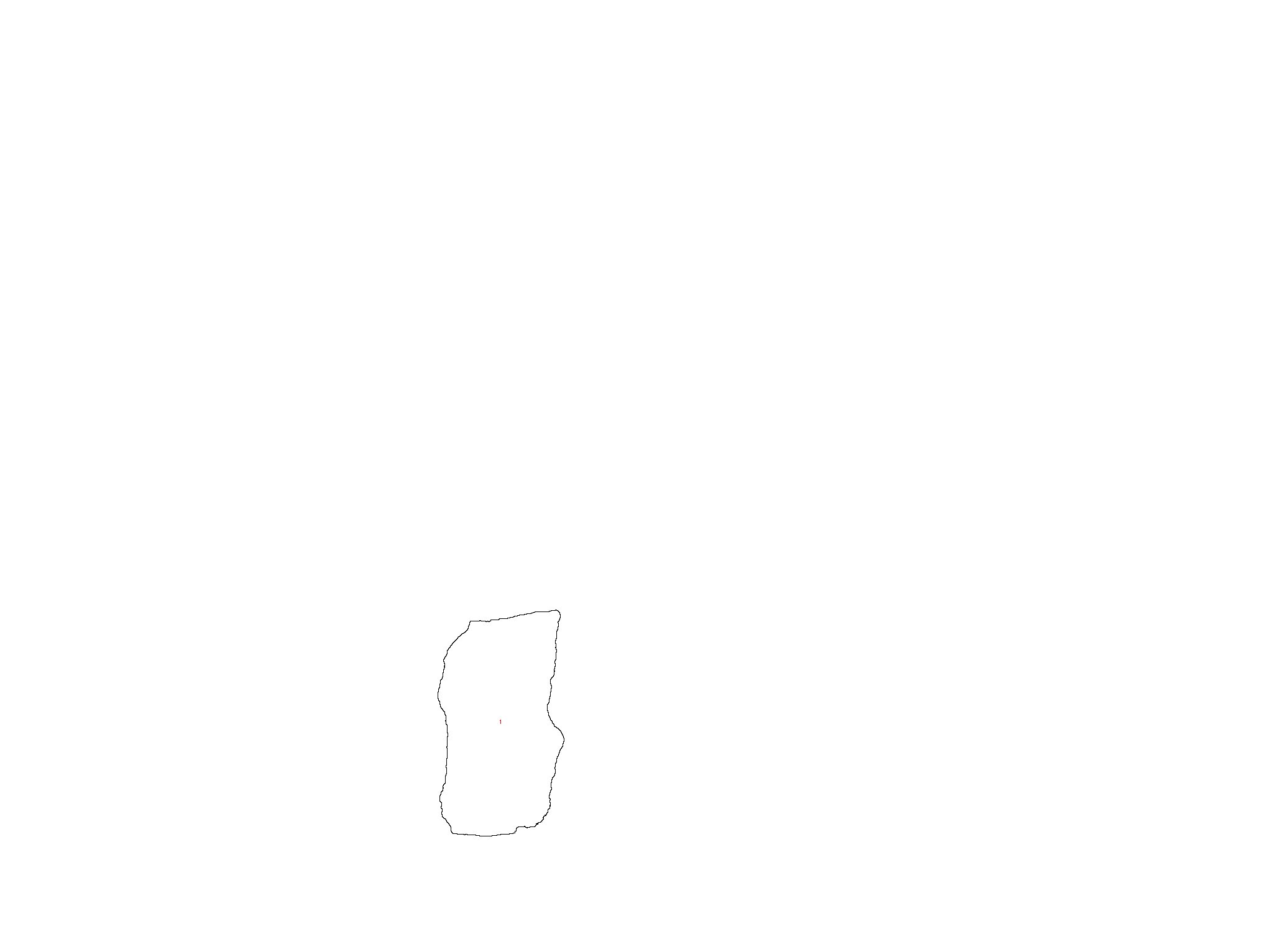

Supplement: S2 Dataset — (ZIP) [file pone.0304198.s005.zip › S2_Dataset_Raw_results_ImageJ/J7_100F_010_11.jpg]

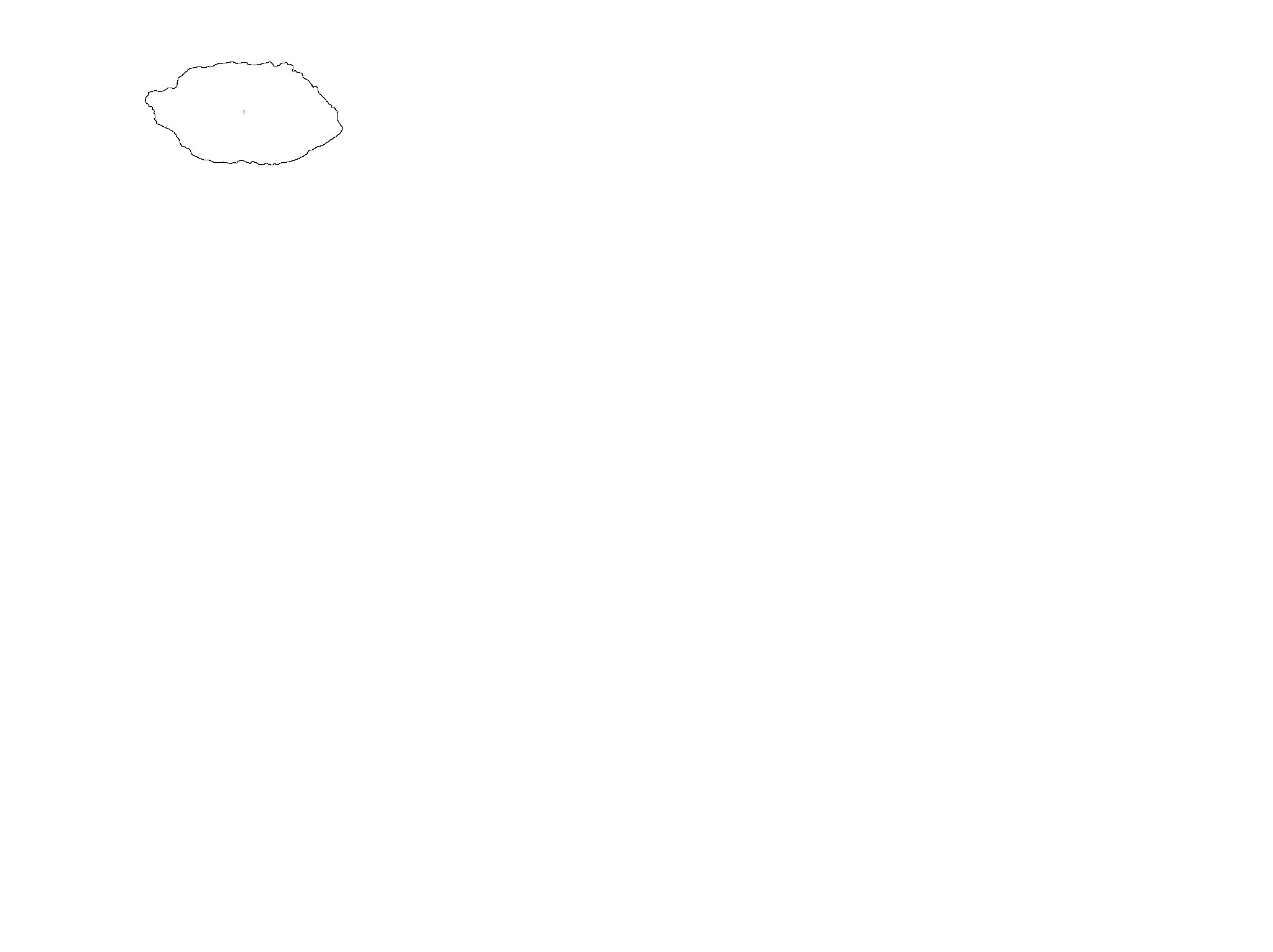

Supplement: S2 Dataset — (ZIP) [file pone.0304198.s005.zip › S2_Dataset_Raw_results_ImageJ/J7_100F_010_12.jpg]

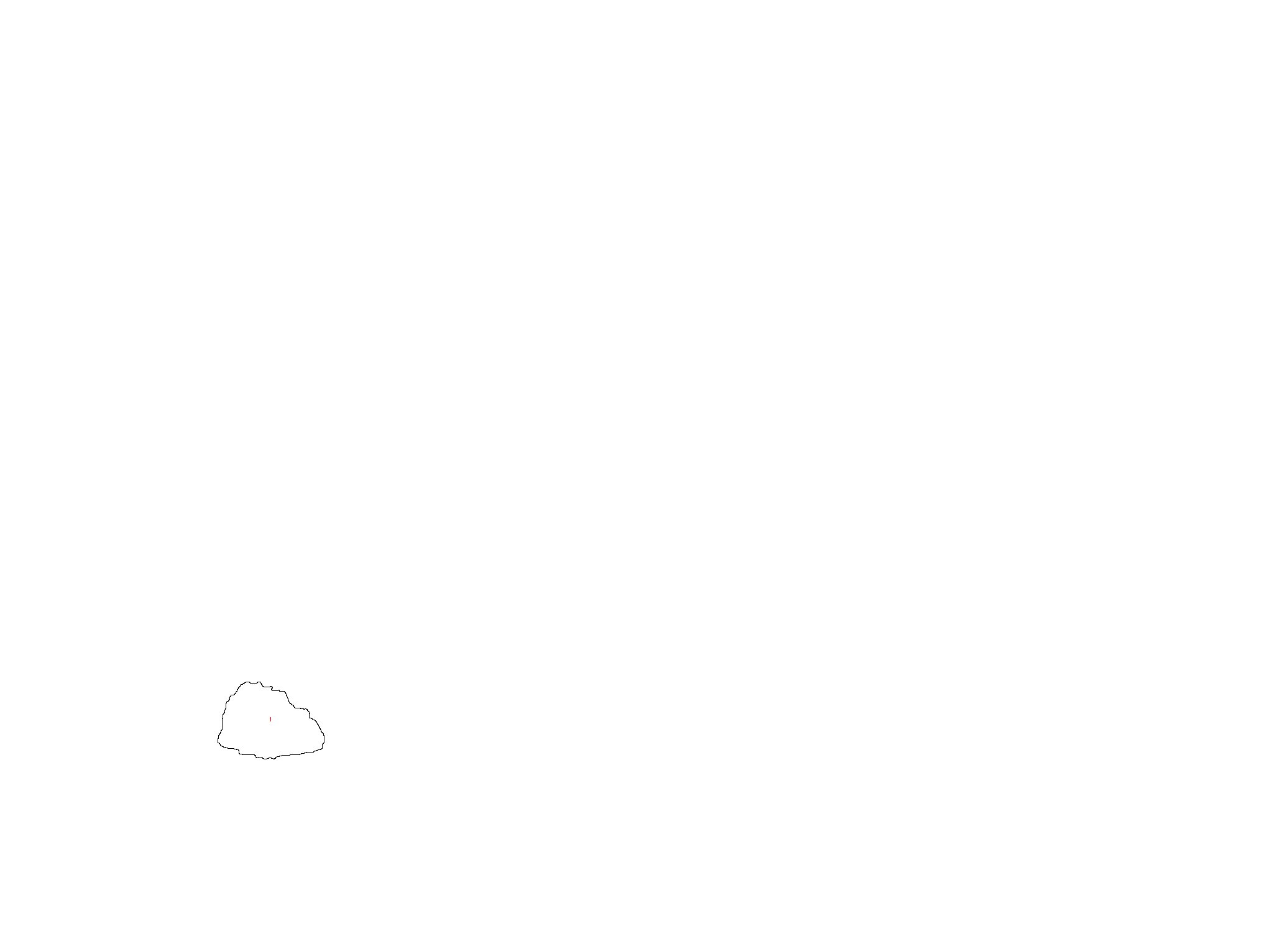

Supplement: S2 Dataset — (ZIP) [file pone.0304198.s005.zip › S2_Dataset_Raw_results_ImageJ/J7_100F_010_2.jpg]

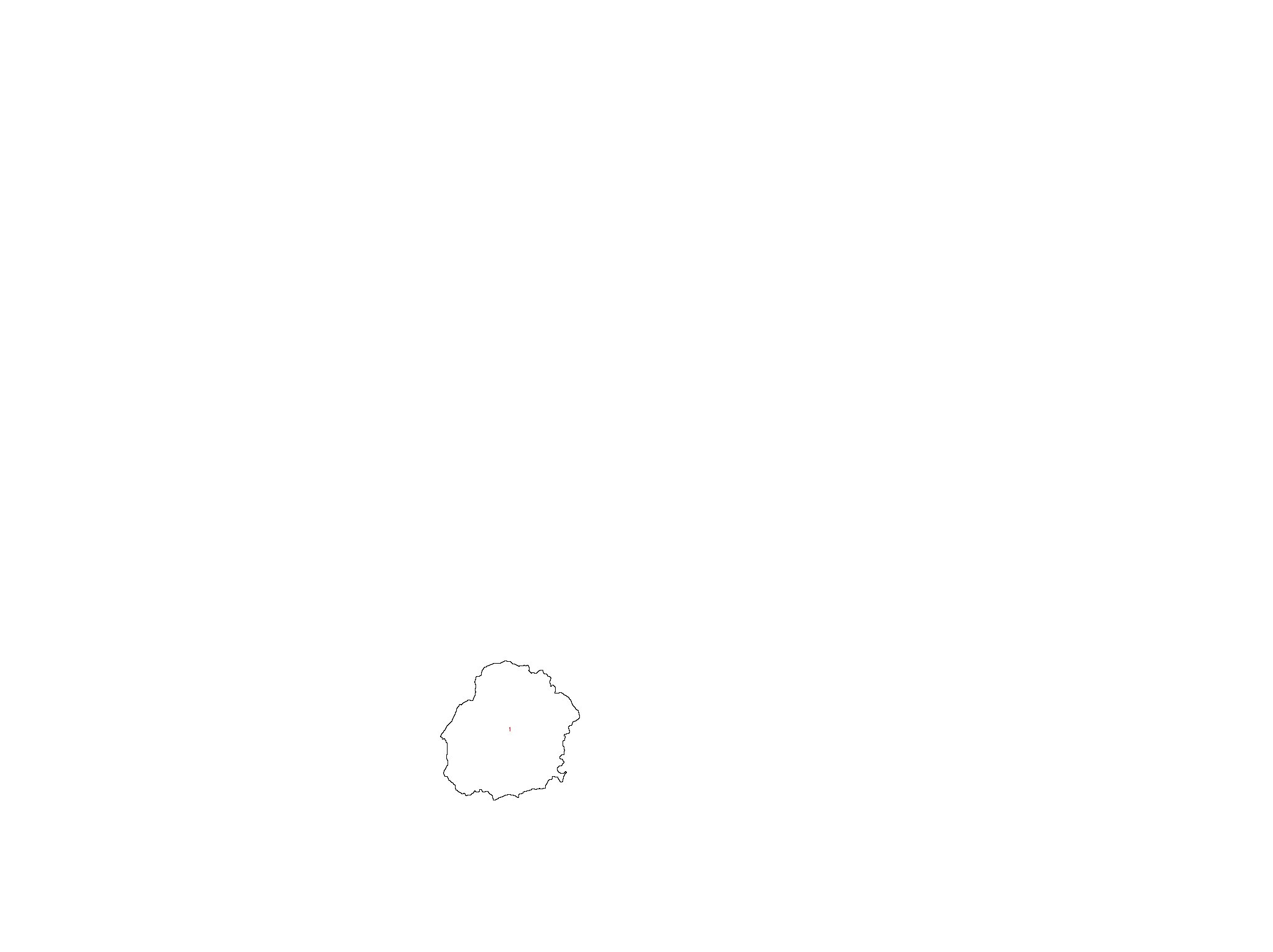

Supplement: S2 Dataset — (ZIP) [file pone.0304198.s005.zip › S2_Dataset_Raw_results_ImageJ/J7_100F_010_4.jpg]

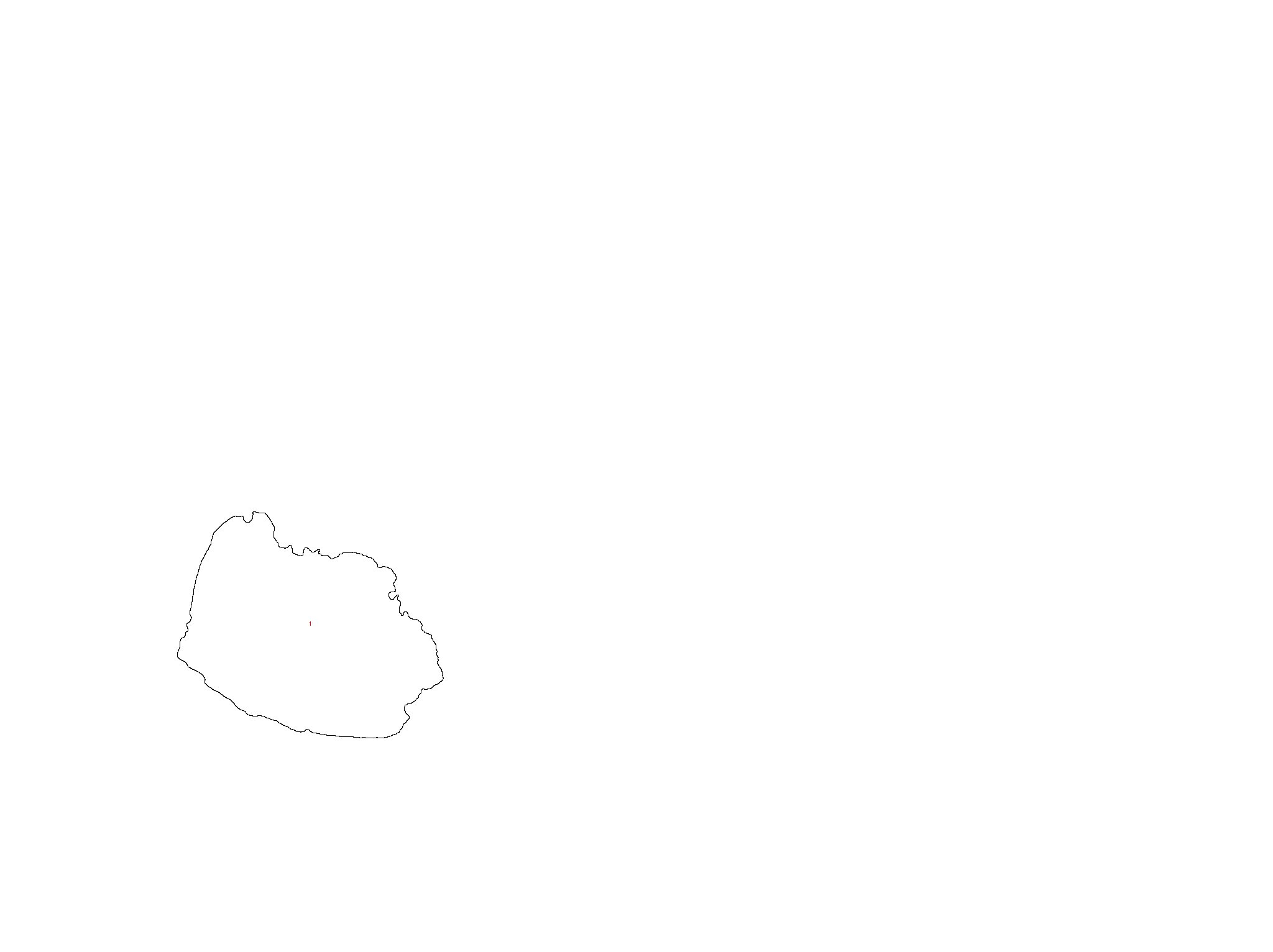

Supplement: S2 Dataset — (ZIP) [file pone.0304198.s005.zip › S2_Dataset_Raw_results_ImageJ/J7_100F_010_5.jpg]

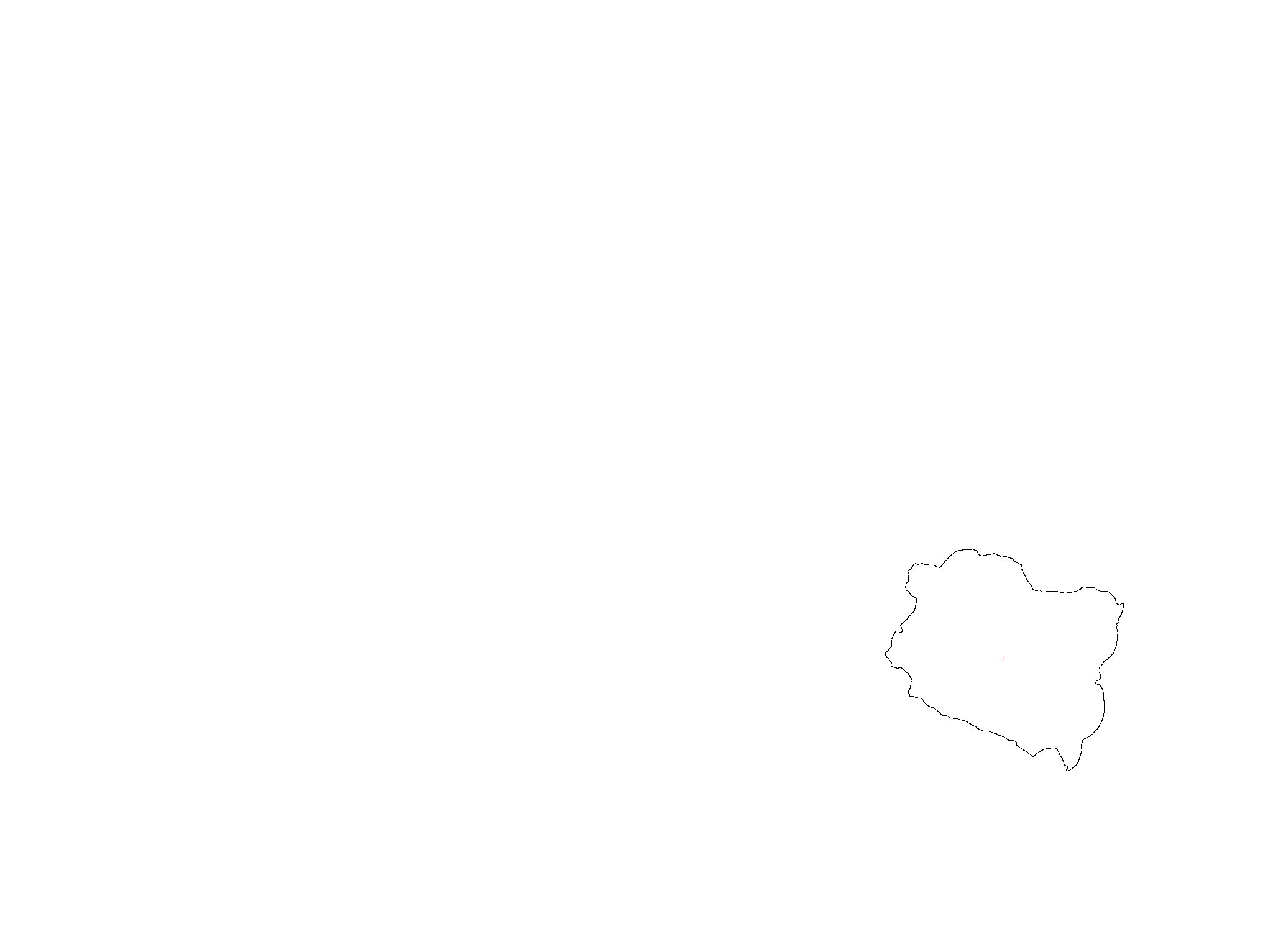

Supplement: S2 Dataset — (ZIP) [file pone.0304198.s005.zip › S2_Dataset_Raw_results_ImageJ/J7_100F_010_9.jpg]

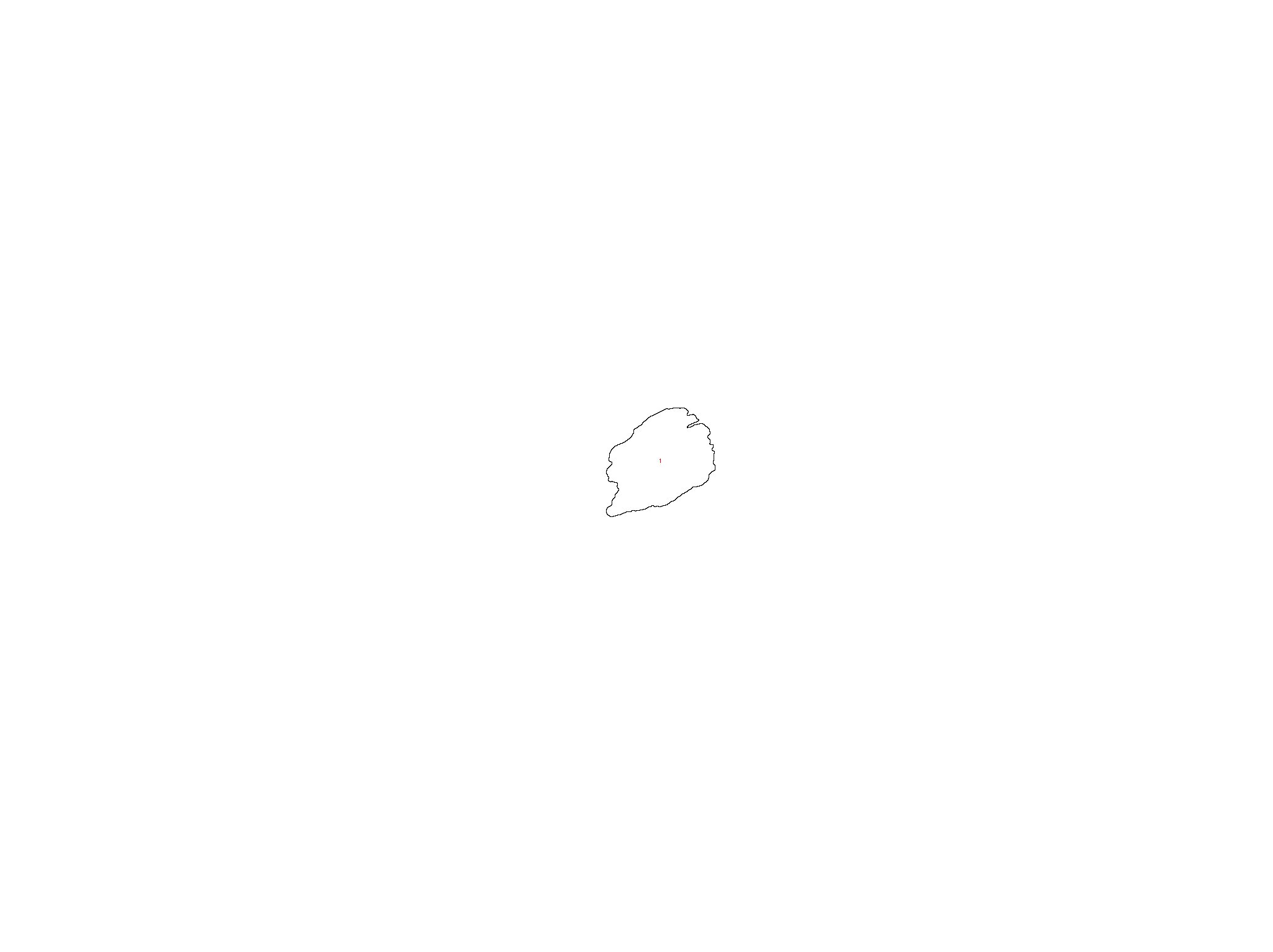

Supplement: S2 Dataset — (ZIP) [file pone.0304198.s005.zip › S2_Dataset_Raw_results_ImageJ/J7_100F_100110_1.jpg]

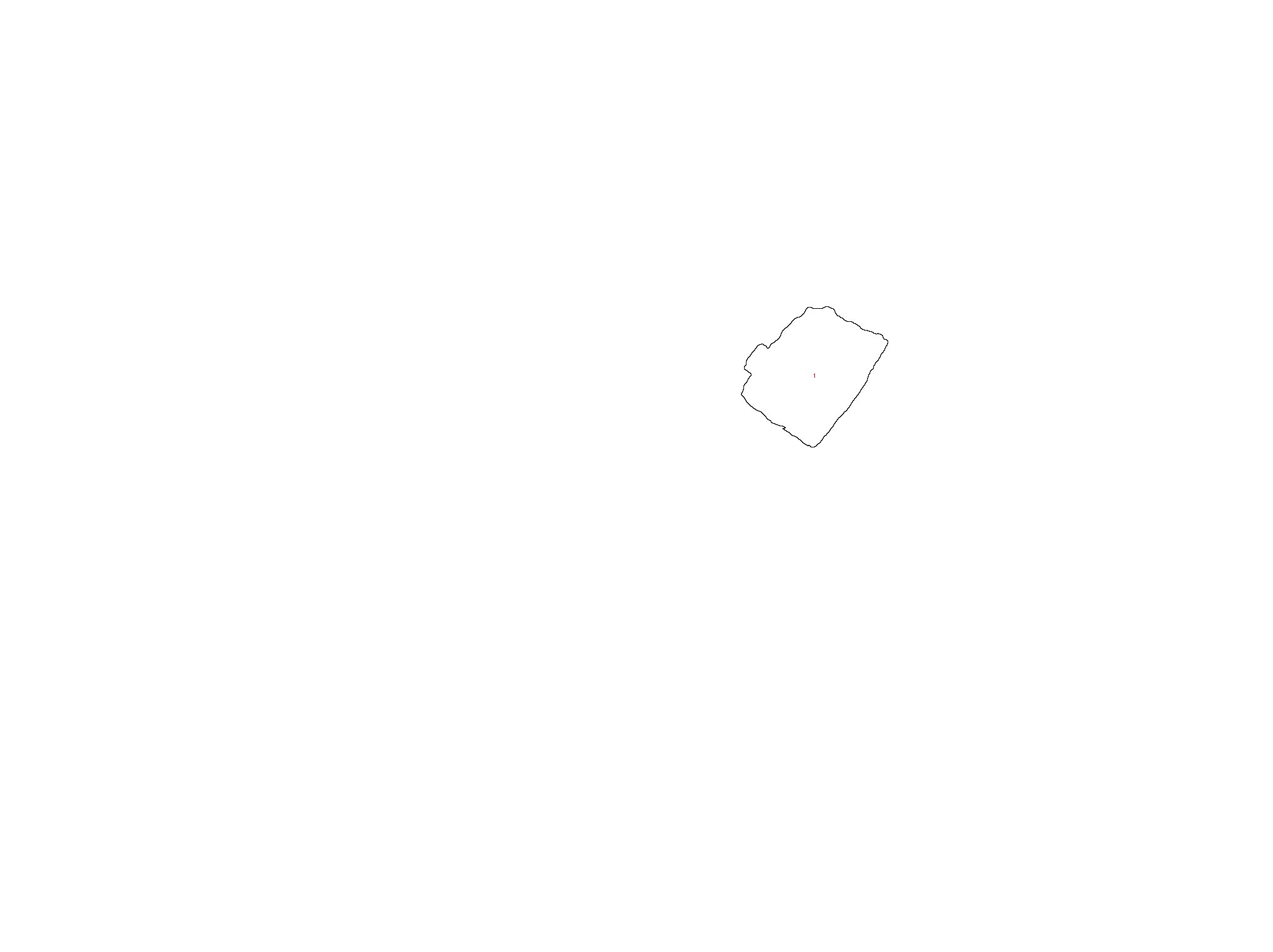

Supplement: S2 Dataset — (ZIP) [file pone.0304198.s005.zip › S2_Dataset_Raw_results_ImageJ/J7_100F_110120_1.jpg]

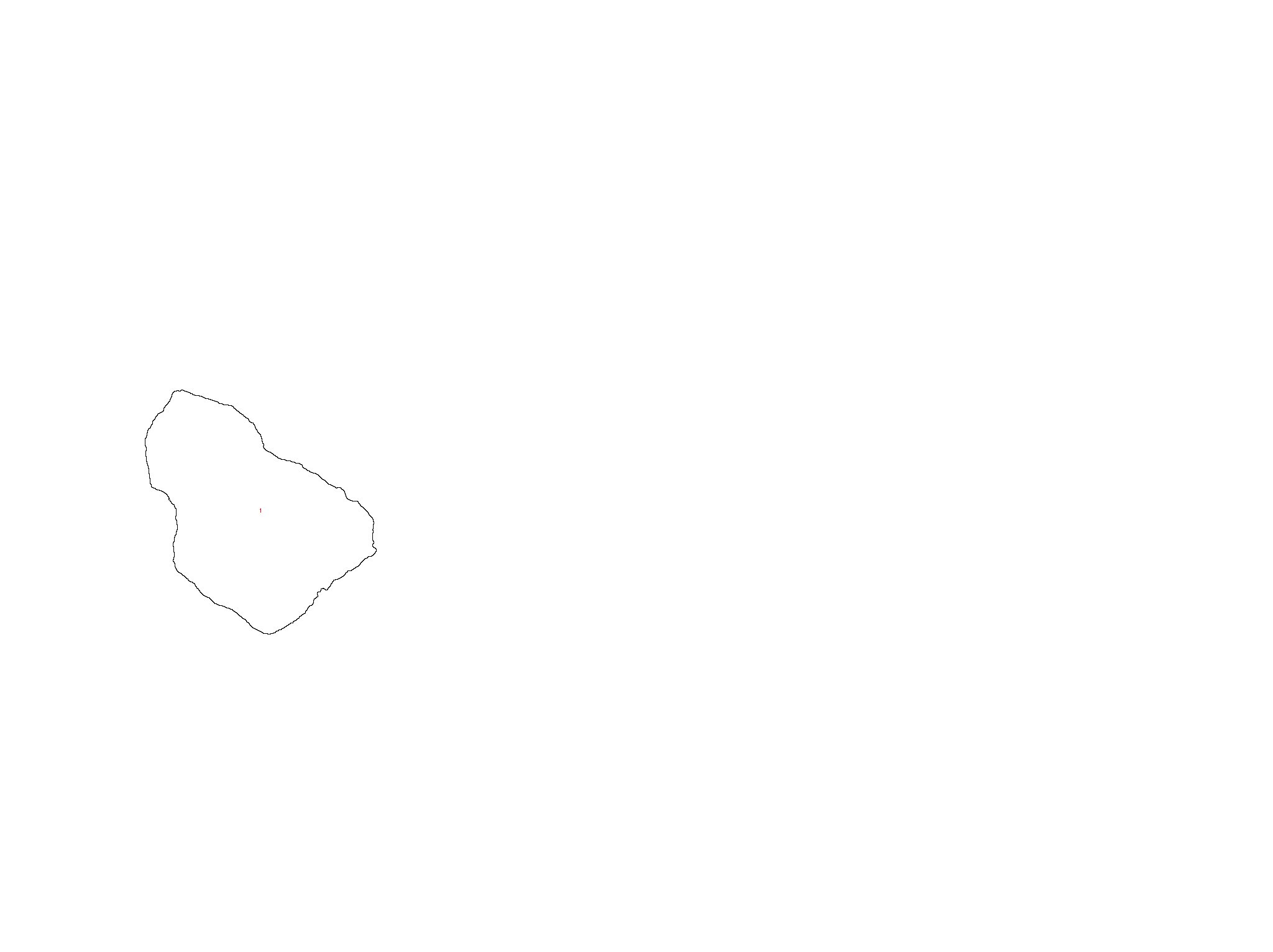

Supplement: S2 Dataset — (ZIP) [file pone.0304198.s005.zip › S2_Dataset_Raw_results_ImageJ/J7_100S_010_1.jpg]

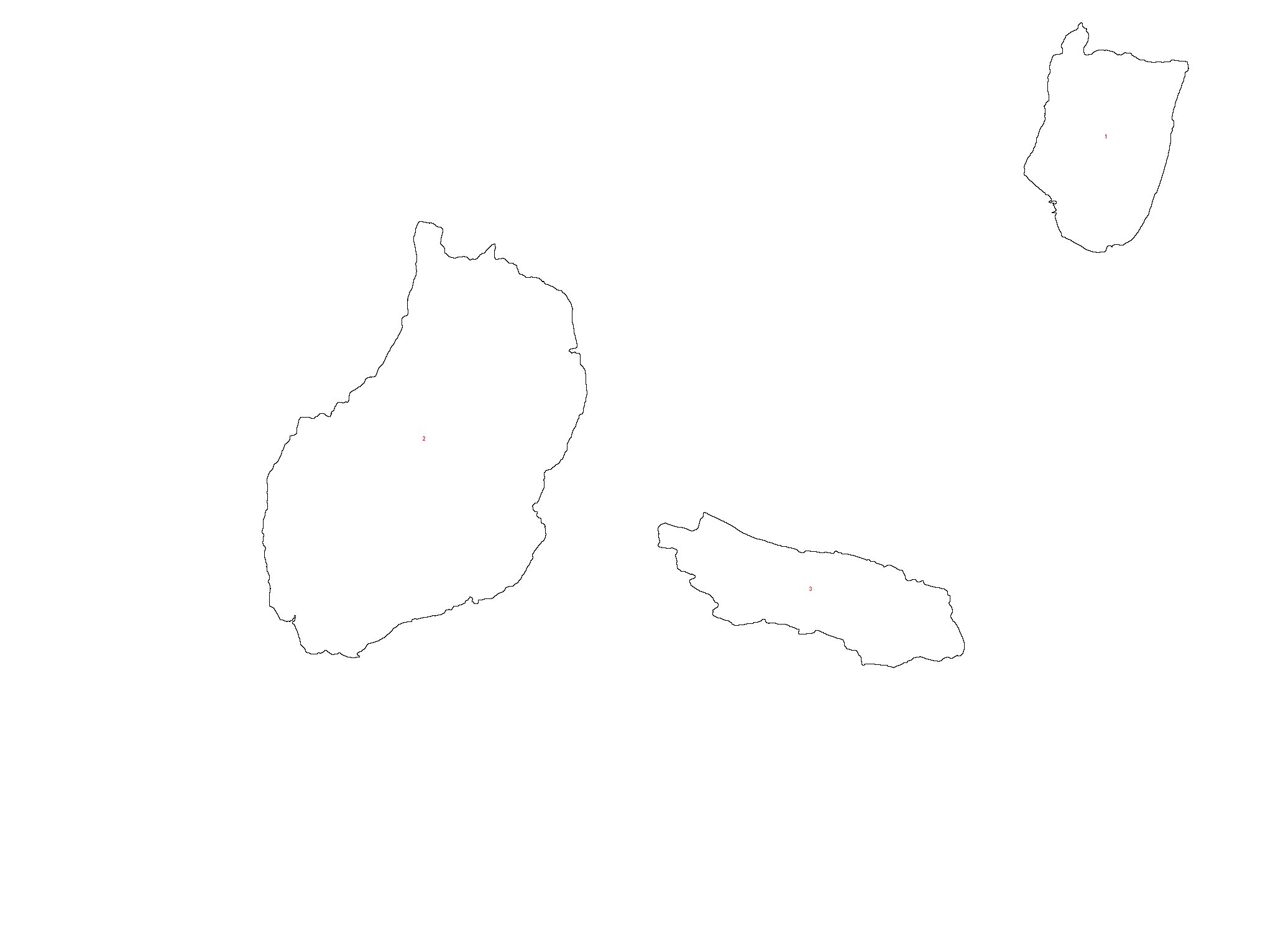

Supplement: S2 Dataset — (ZIP) [file pone.0304198.s005.zip › S2_Dataset_Raw_results_ImageJ/J7_100S_010_10.jpg]

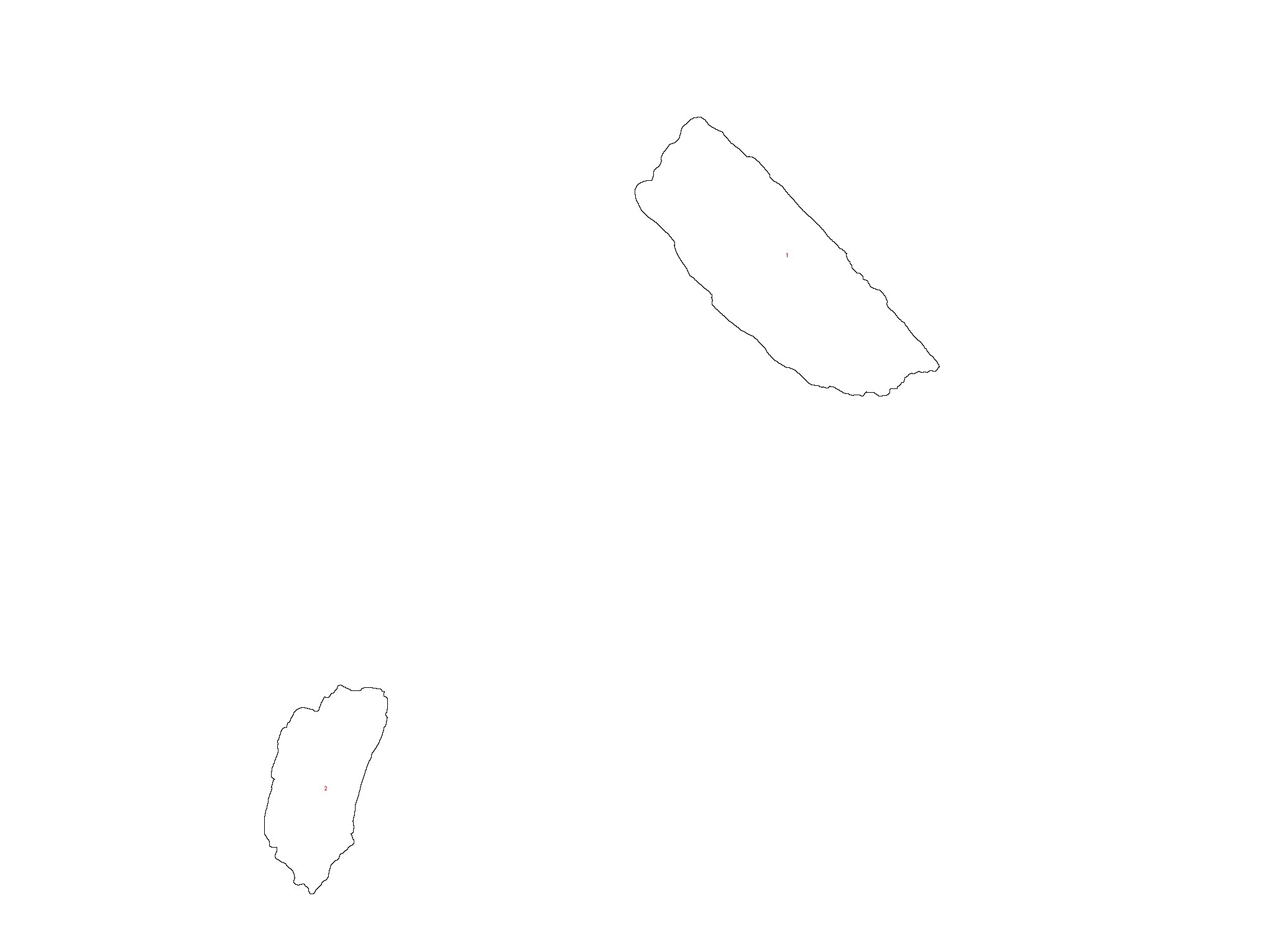

Supplement: S2 Dataset — (ZIP) [file pone.0304198.s005.zip › S2_Dataset_Raw_results_ImageJ/J7_100S_010_11.jpg]

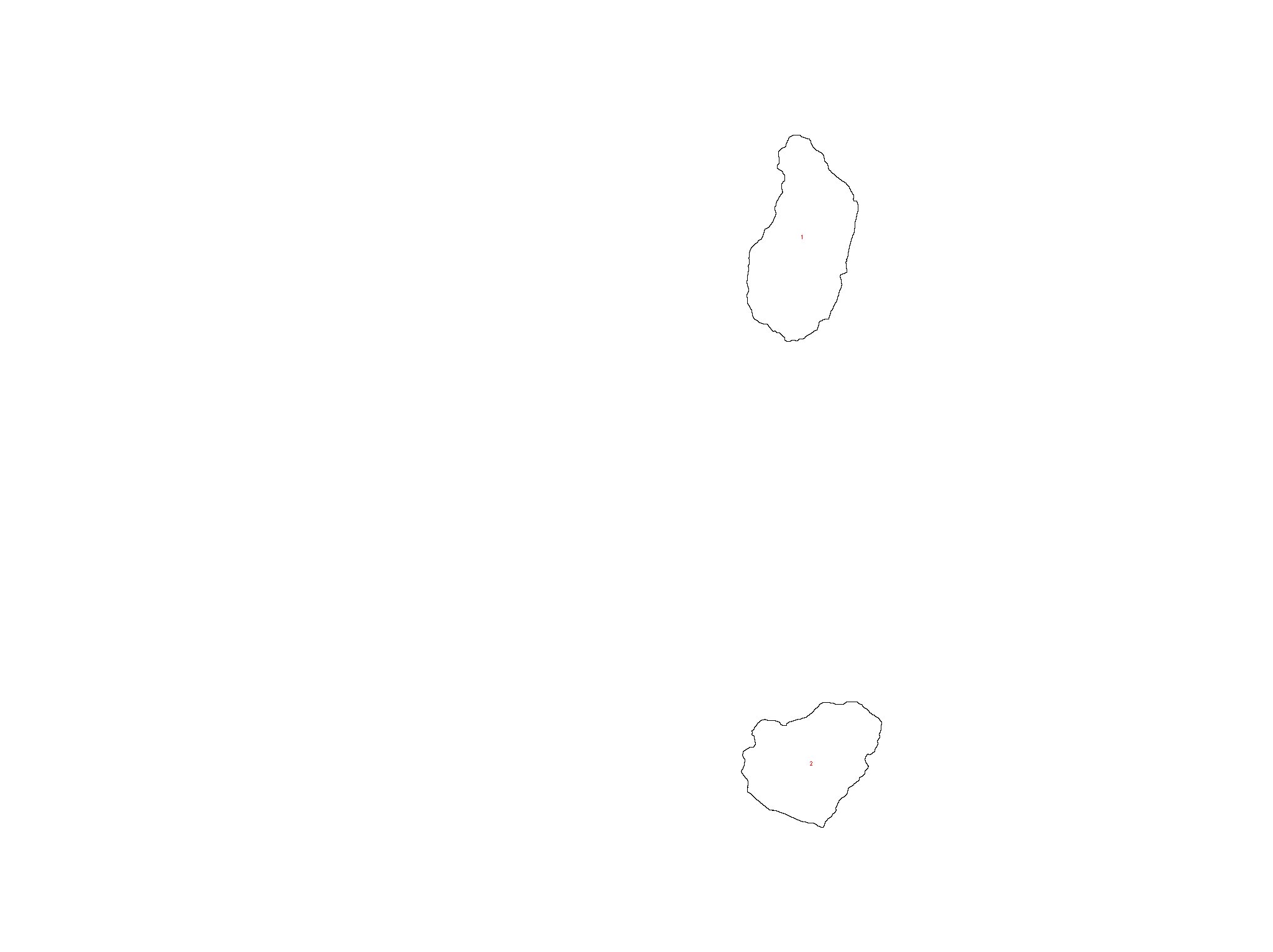

Supplement: S2 Dataset — (ZIP) [file pone.0304198.s005.zip › S2_Dataset_Raw_results_ImageJ/J7_100S_010_12.jpg]

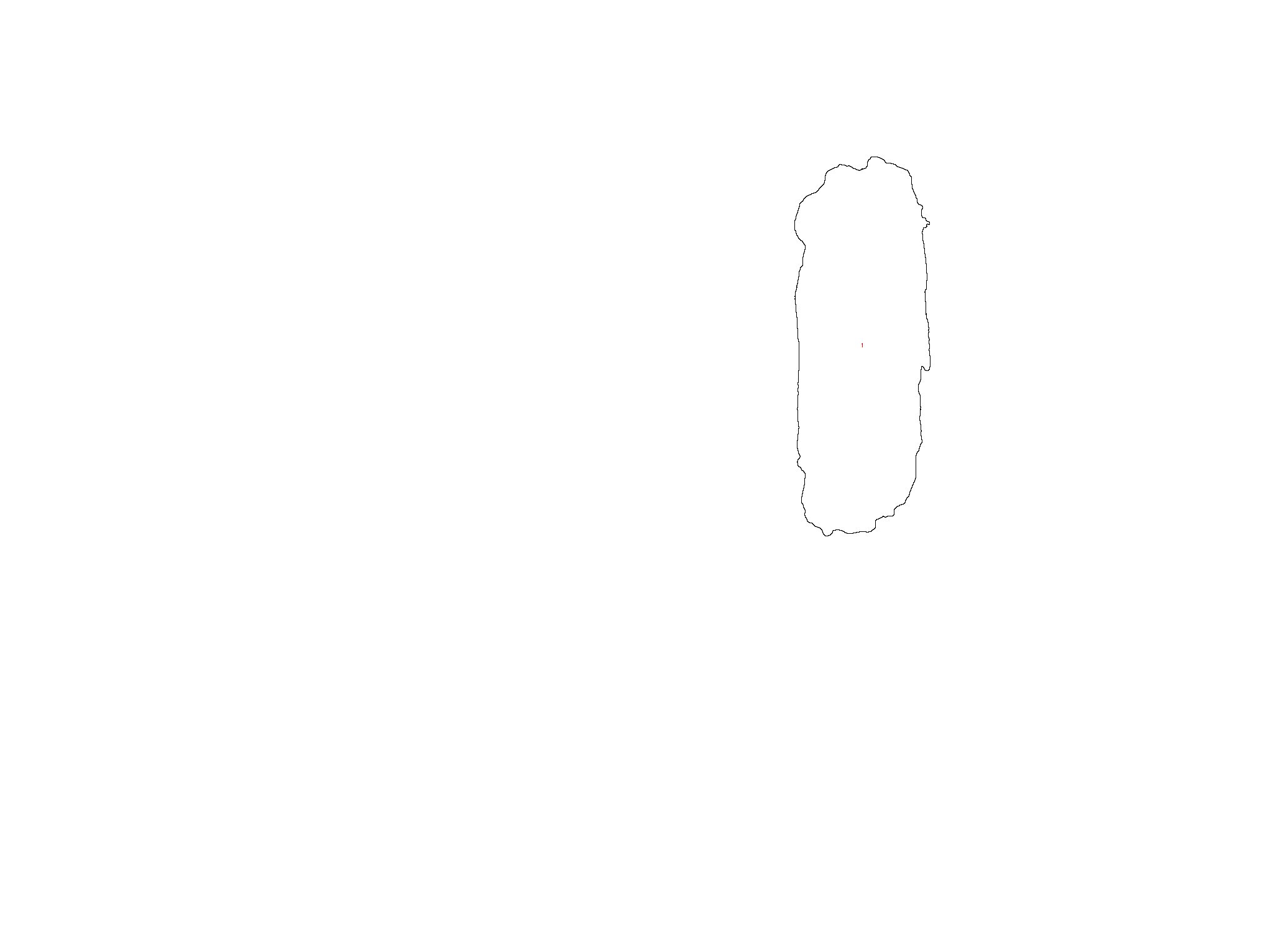

Supplement: S2 Dataset — (ZIP) [file pone.0304198.s005.zip › S2_Dataset_Raw_results_ImageJ/J7_100S_010_13.jpg]

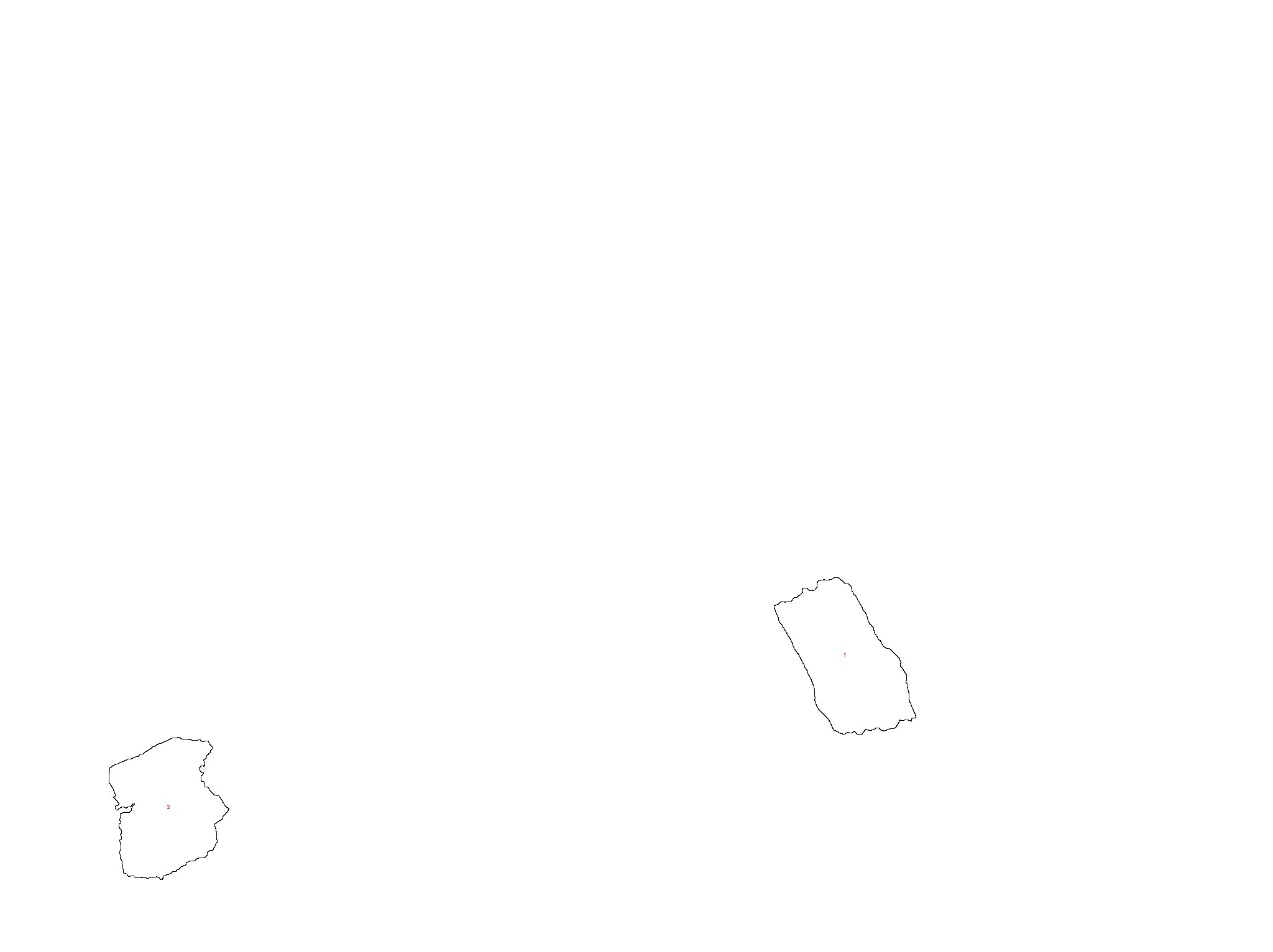

Supplement: S2 Dataset — (ZIP) [file pone.0304198.s005.zip › S2_Dataset_Raw_results_ImageJ/J7_100S_010_14.jpg]

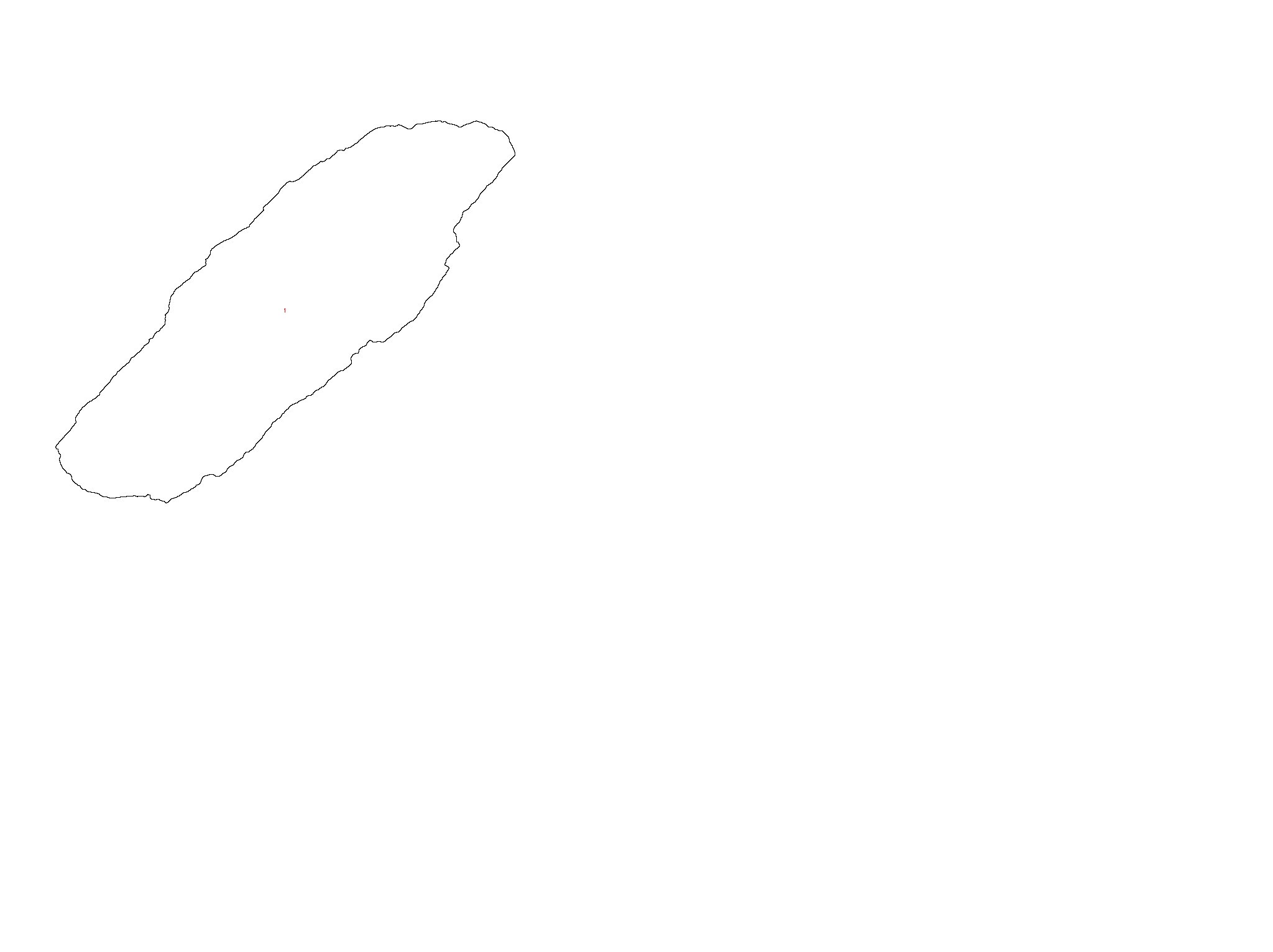

Supplement: S2 Dataset — (ZIP) [file pone.0304198.s005.zip › S2_Dataset_Raw_results_ImageJ/J7_100S_010_15.jpg]

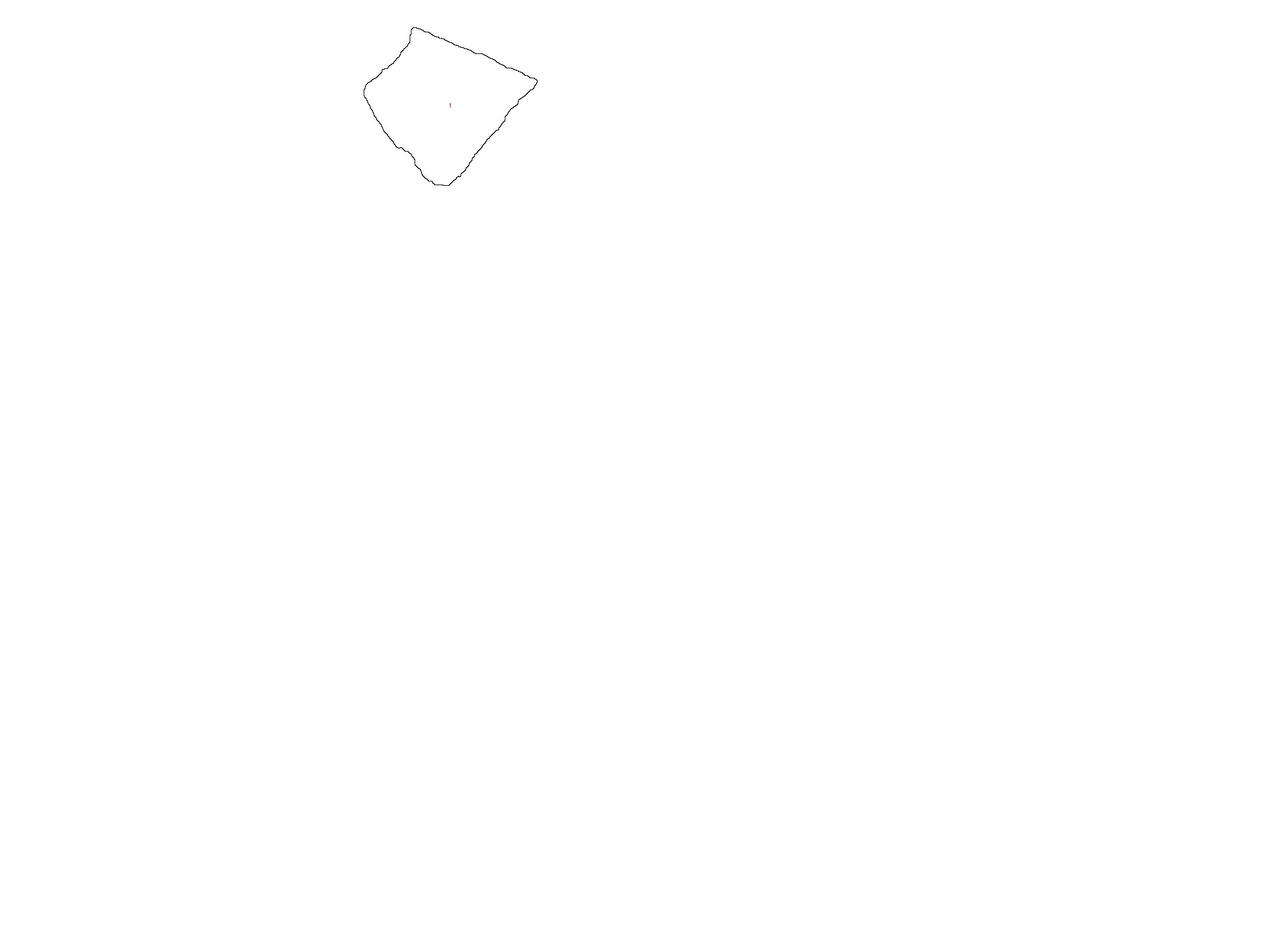

Supplement: S2 Dataset — (ZIP) [file pone.0304198.s005.zip › S2_Dataset_Raw_results_ImageJ/J7_100S_010_16.jpg]

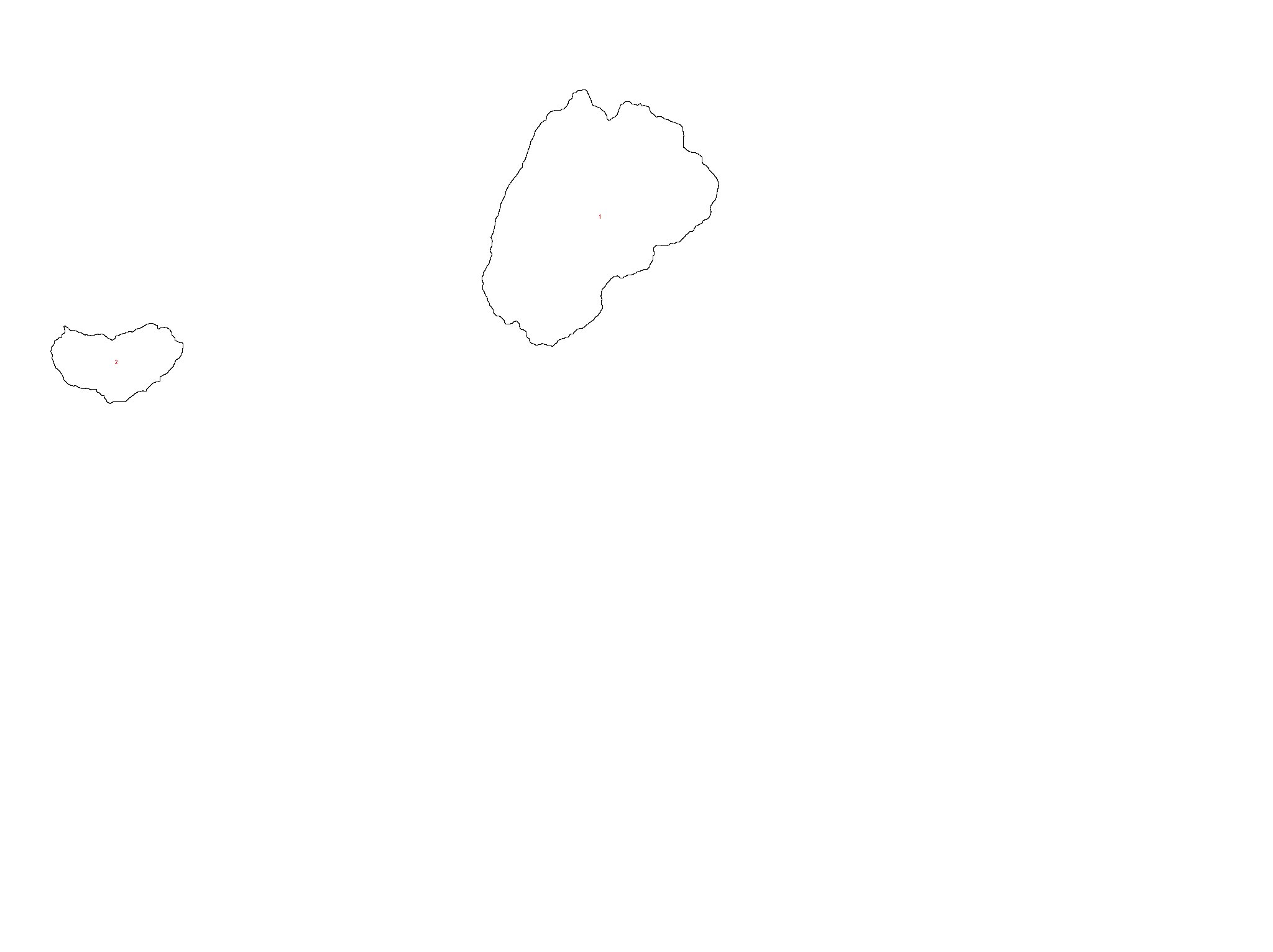

Supplement: S2 Dataset — (ZIP) [file pone.0304198.s005.zip › S2_Dataset_Raw_results_ImageJ/J7_100S_010_17.jpg]

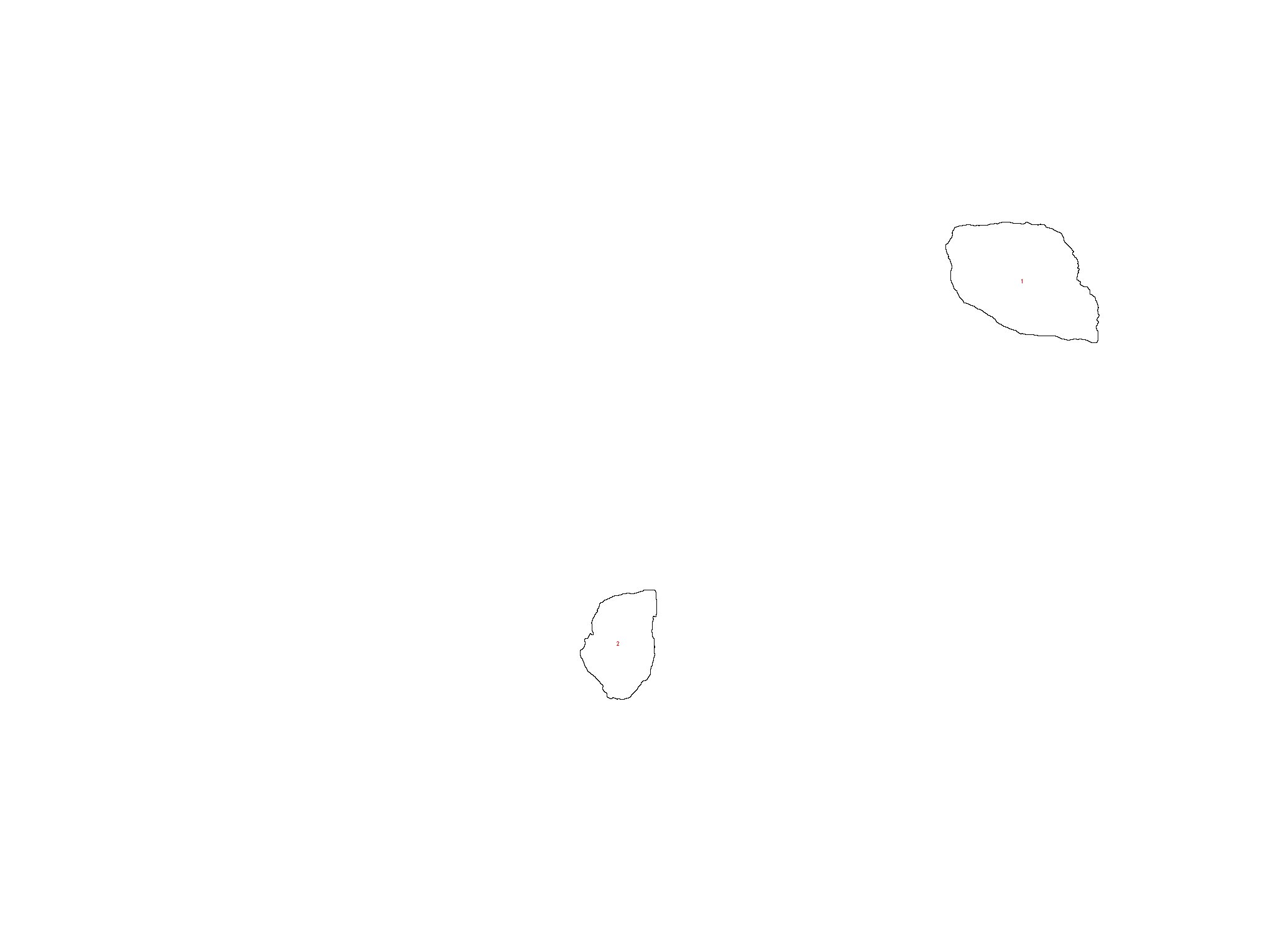

Supplement: S2 Dataset — (ZIP) [file pone.0304198.s005.zip › S2_Dataset_Raw_results_ImageJ/J7_100S_010_2.jpg]

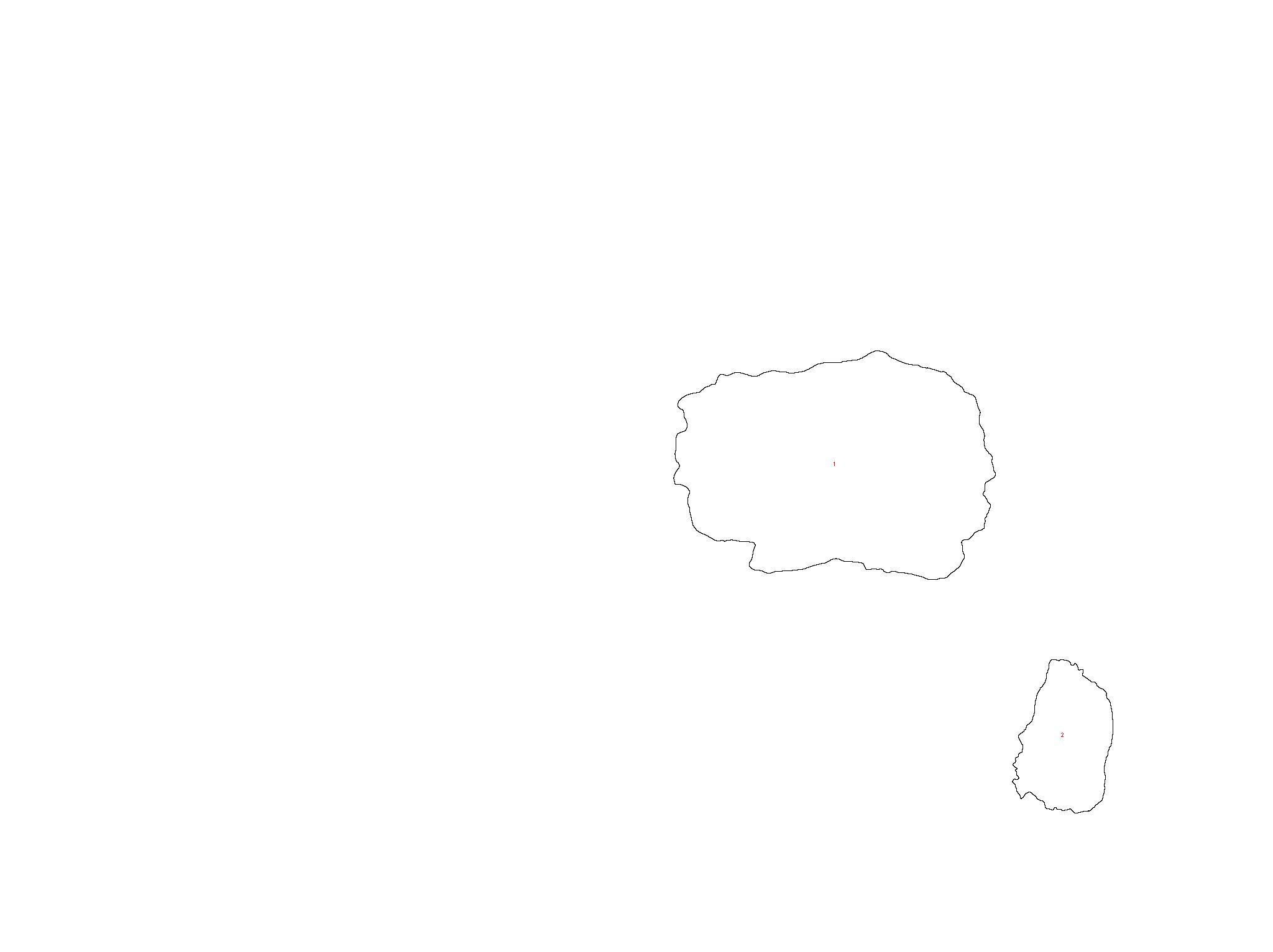

Supplement: S2 Dataset — (ZIP) [file pone.0304198.s005.zip › S2_Dataset_Raw_results_ImageJ/J7_100S_010_3.jpg]

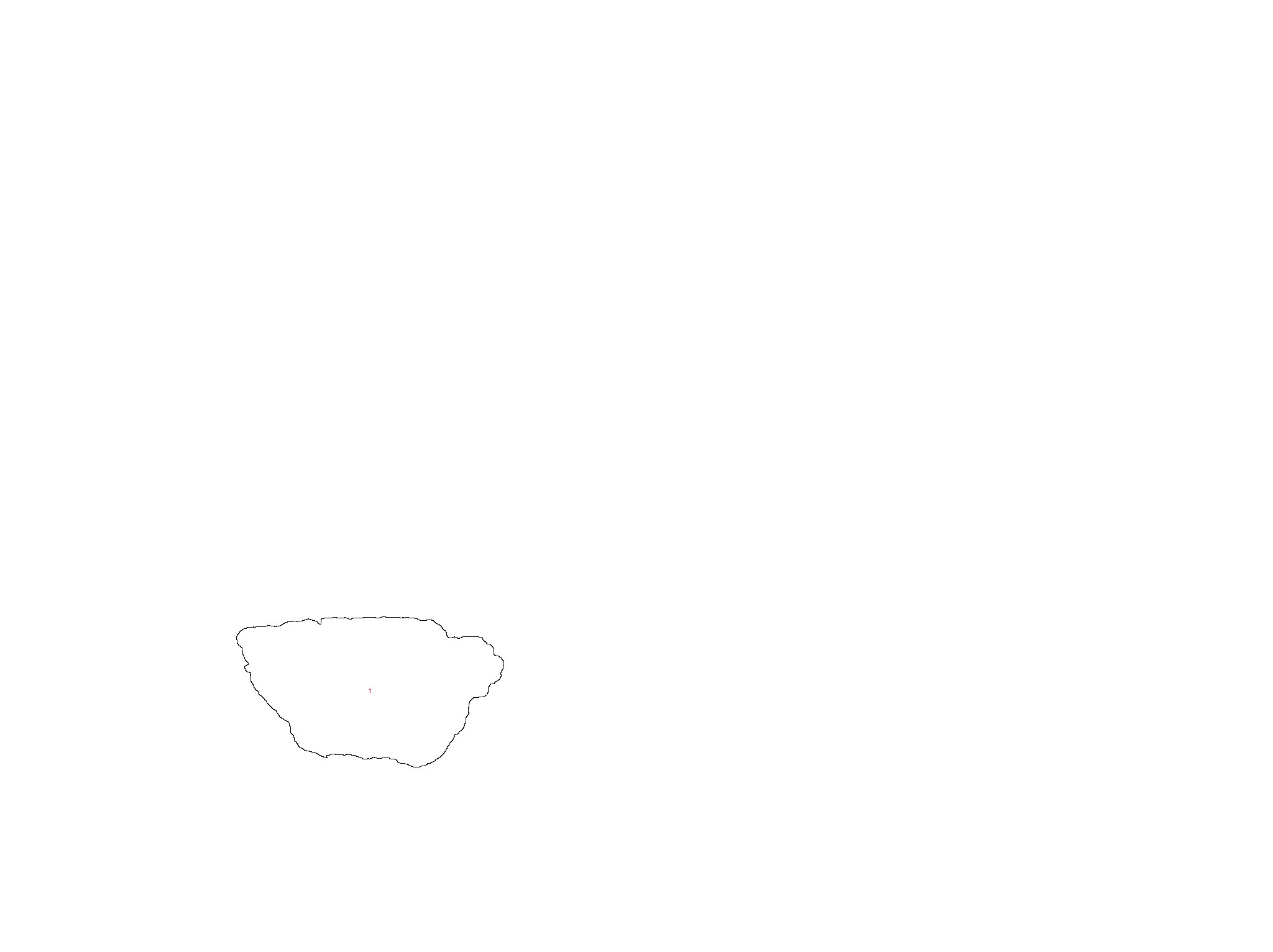

Supplement: S2 Dataset — (ZIP) [file pone.0304198.s005.zip › S2_Dataset_Raw_results_ImageJ/J7_100S_010_4.jpg]

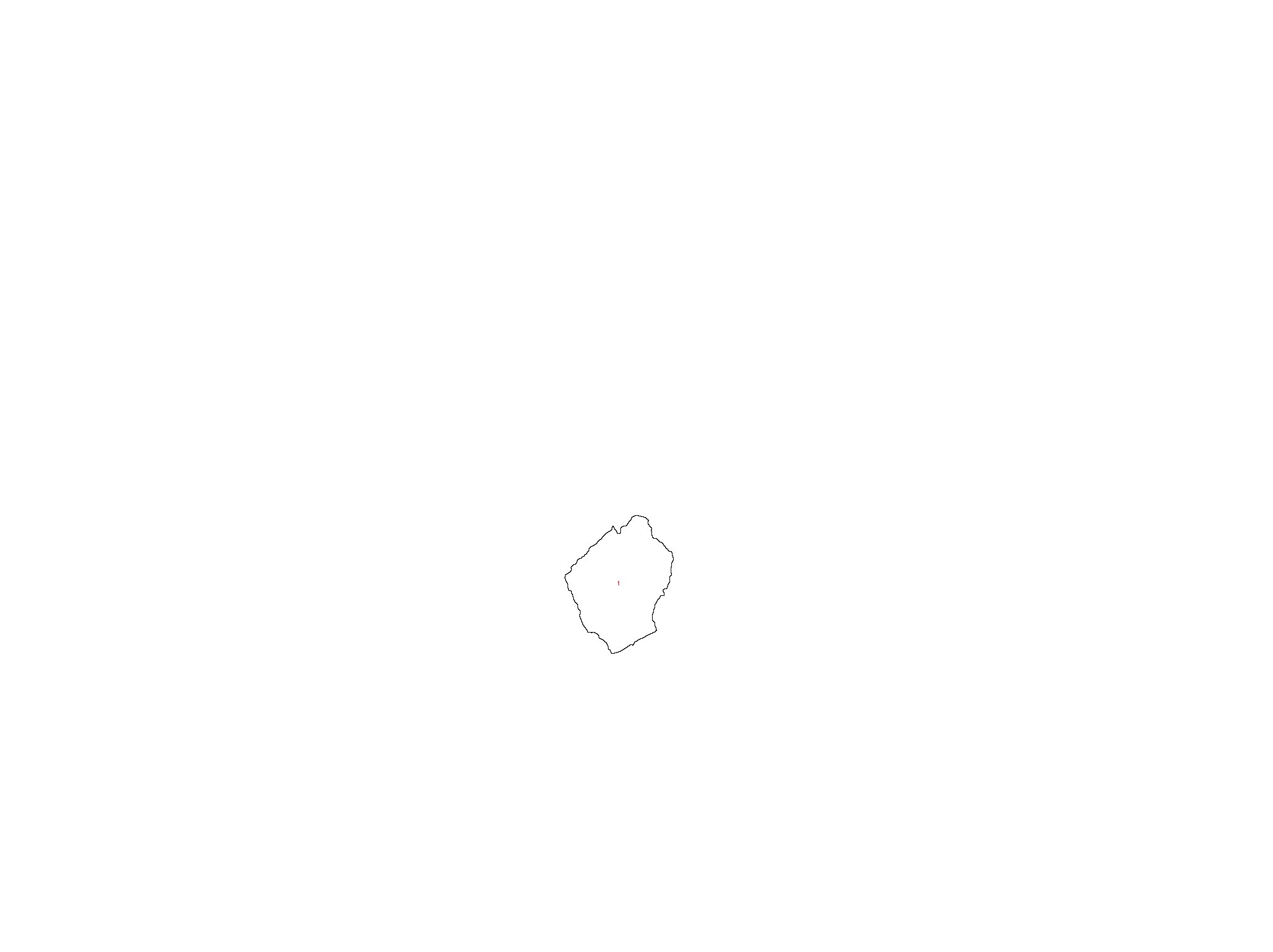

Supplement: S2 Dataset — (ZIP) [file pone.0304198.s005.zip › S2_Dataset_Raw_results_ImageJ/J7_100S_010_5.jpg]

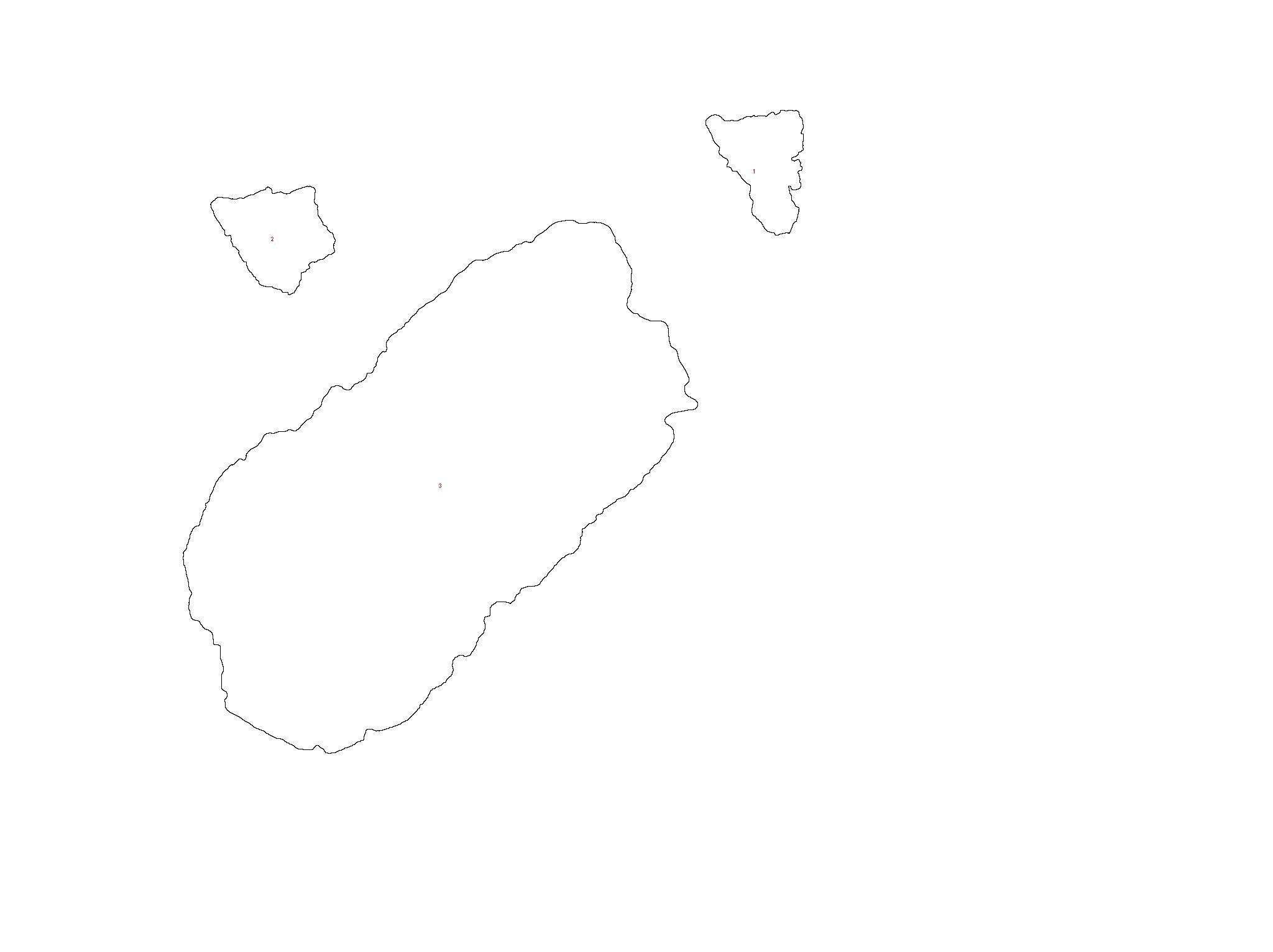

Supplement: S2 Dataset — (ZIP) [file pone.0304198.s005.zip › S2_Dataset_Raw_results_ImageJ/J7_100S_010_6.jpg]

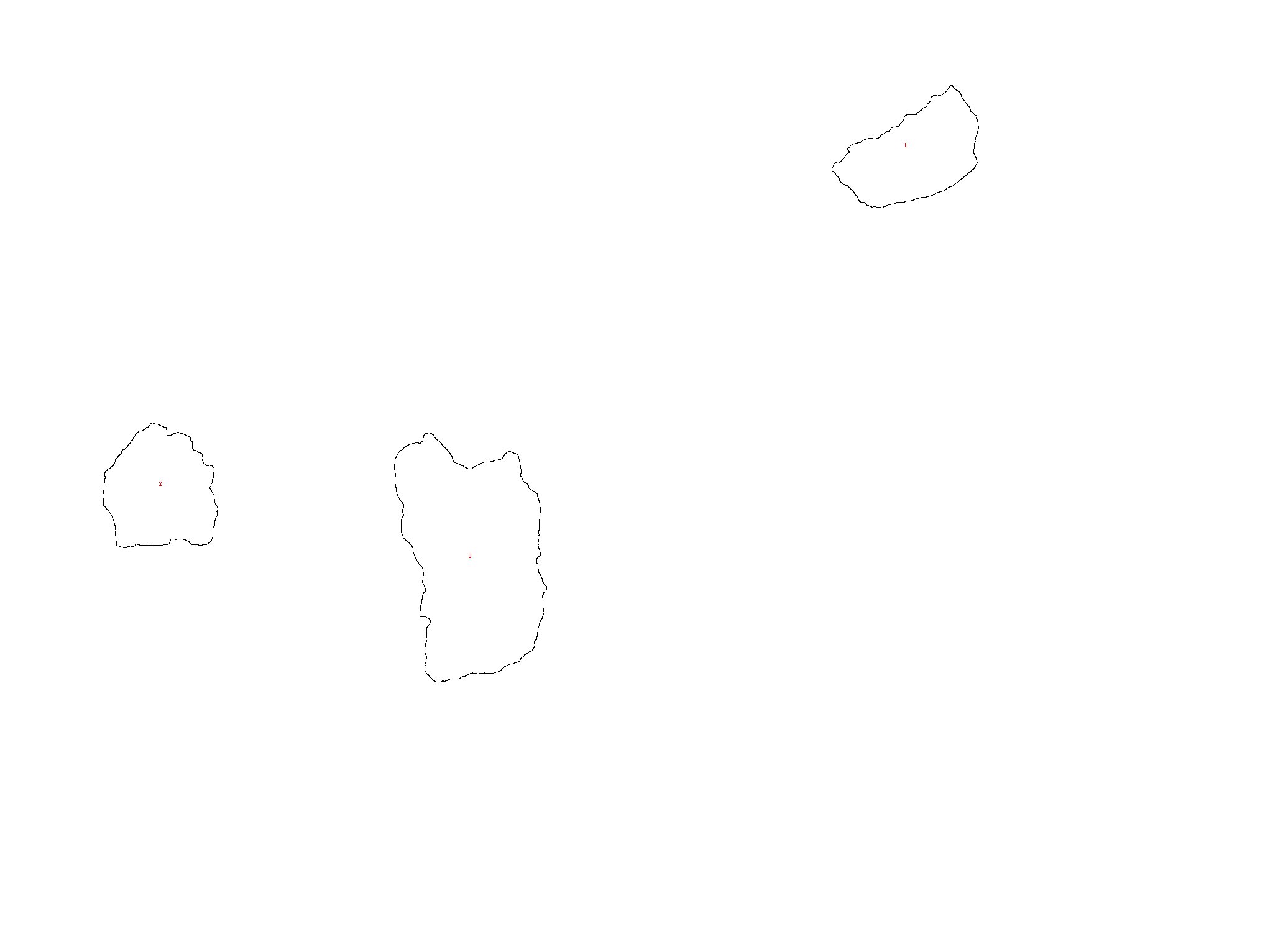

Supplement: S2 Dataset — (ZIP) [file pone.0304198.s005.zip › S2_Dataset_Raw_results_ImageJ/J7_100S_010_7.jpg]

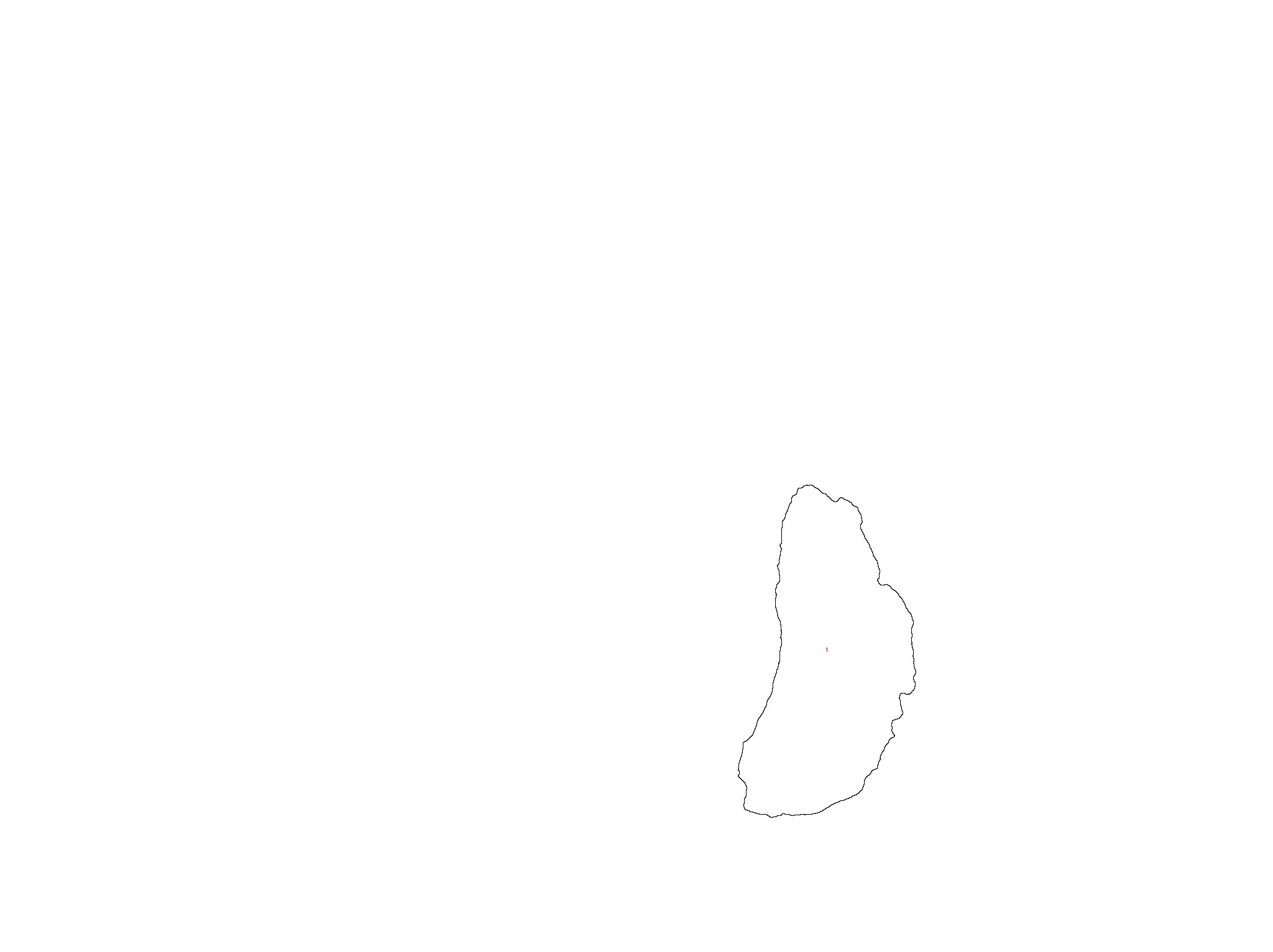

Supplement: S2 Dataset — (ZIP) [file pone.0304198.s005.zip › S2_Dataset_Raw_results_ImageJ/J7_100S_010_8.jpg]

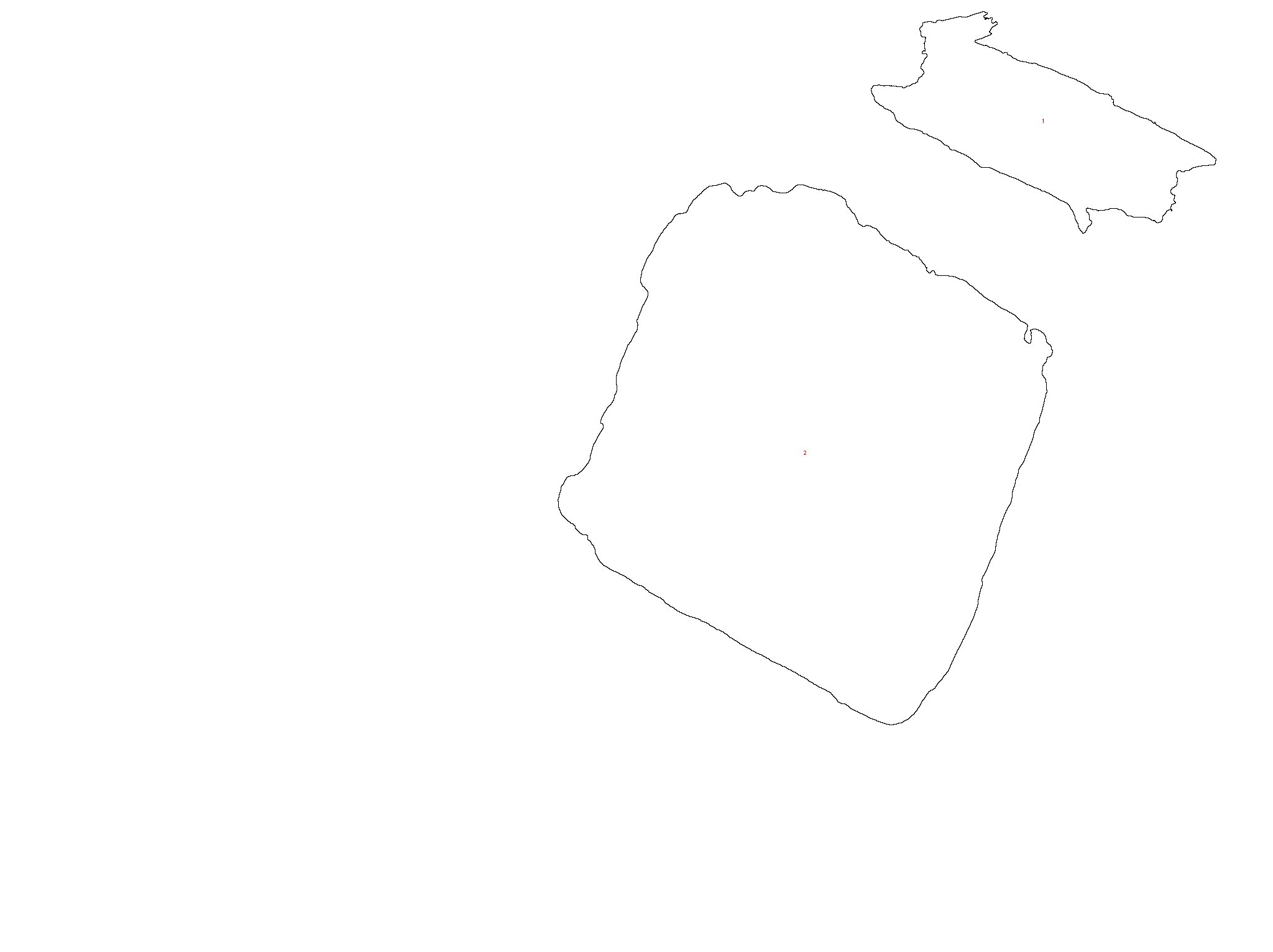

Supplement: S2 Dataset — (ZIP) [file pone.0304198.s005.zip › S2_Dataset_Raw_results_ImageJ/J7_100S_010_9.jpg]

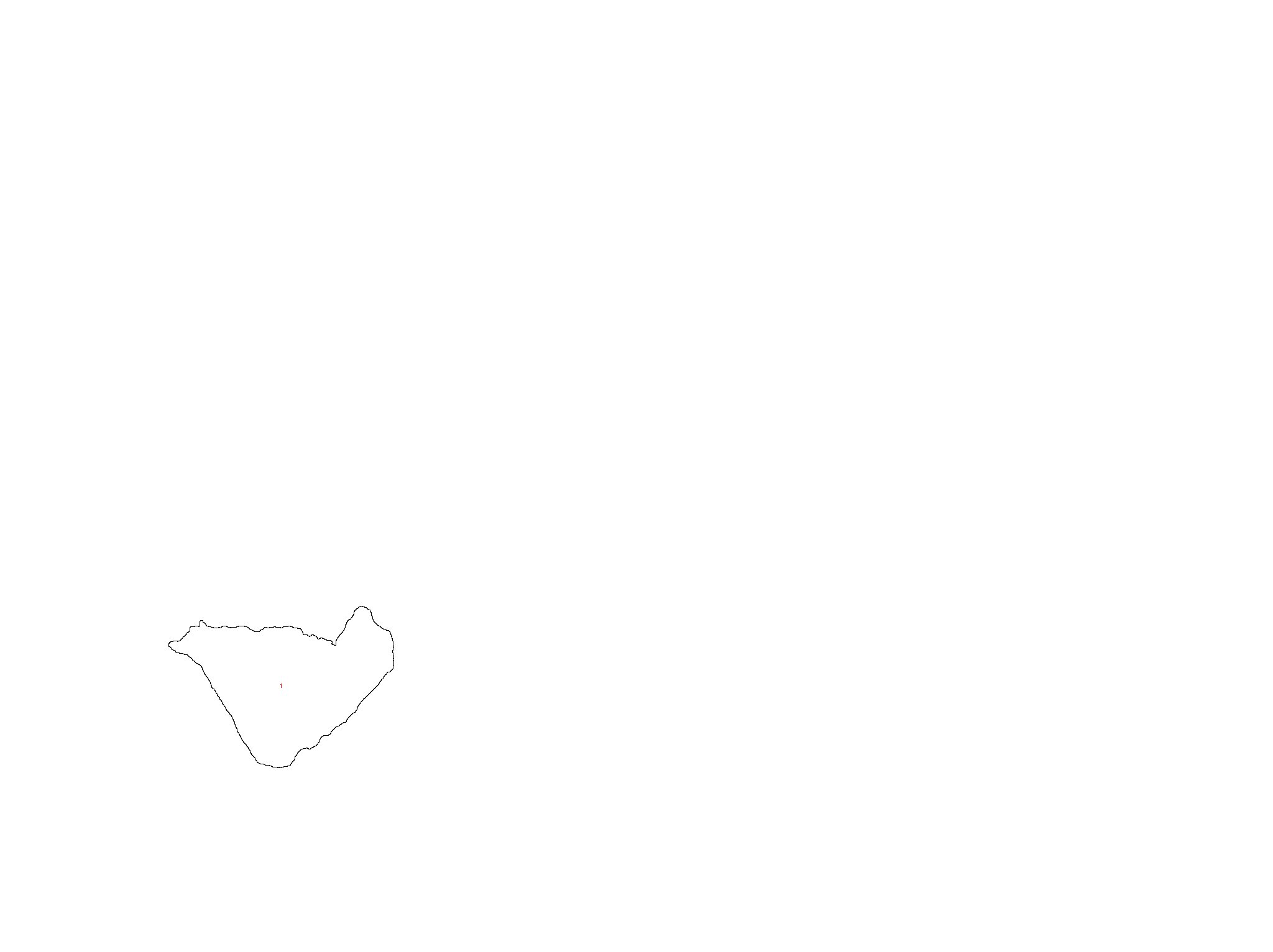

Supplement: S2 Dataset — (ZIP) [file pone.0304198.s005.zip › S2_Dataset_Raw_results_ImageJ/J7_100S_110120_1.jpg]

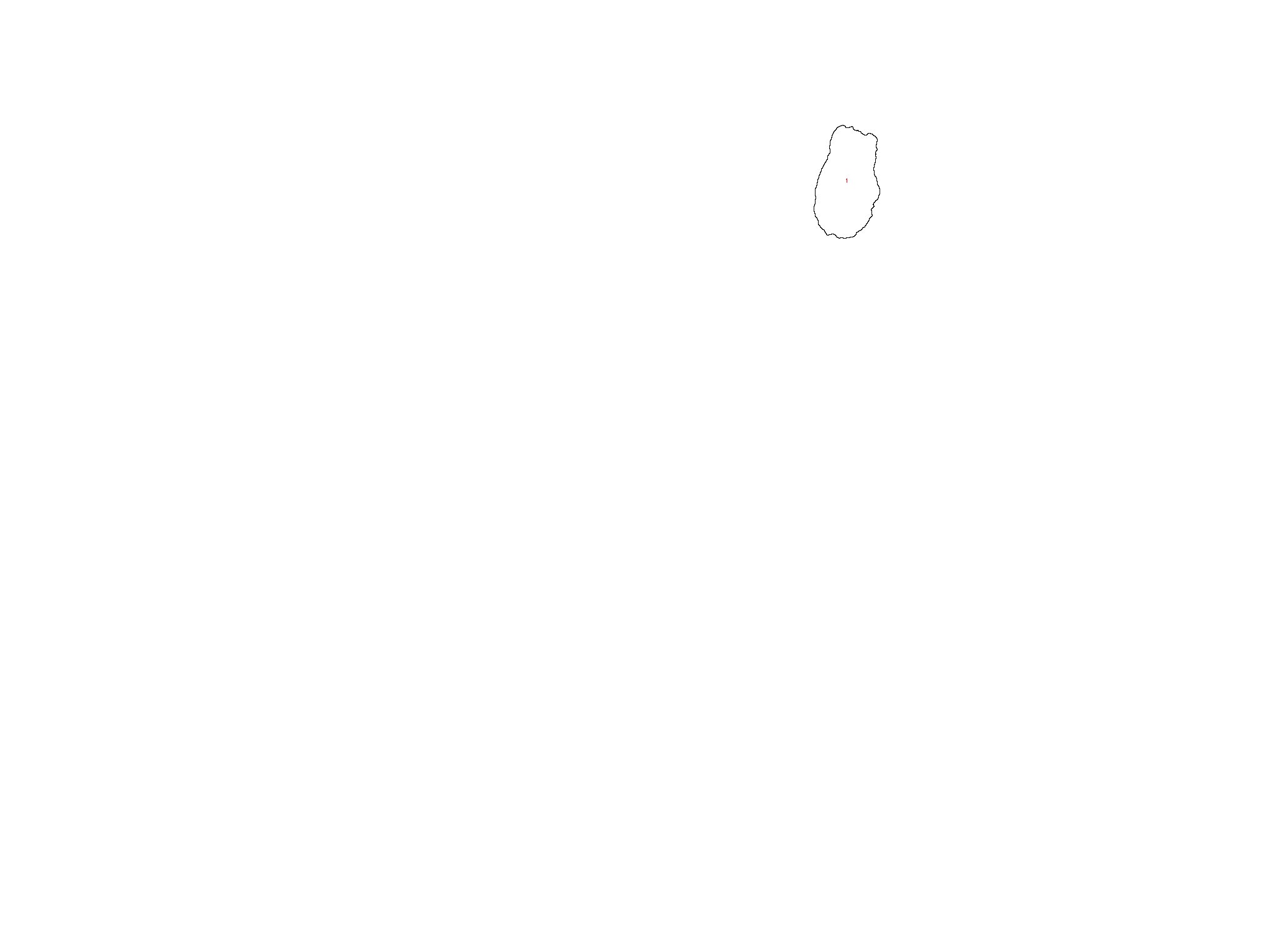

Supplement: S2 Dataset — (ZIP) [file pone.0304198.s005.zip › S2_Dataset_Raw_results_ImageJ/J7_100S_110120_2.jpg]

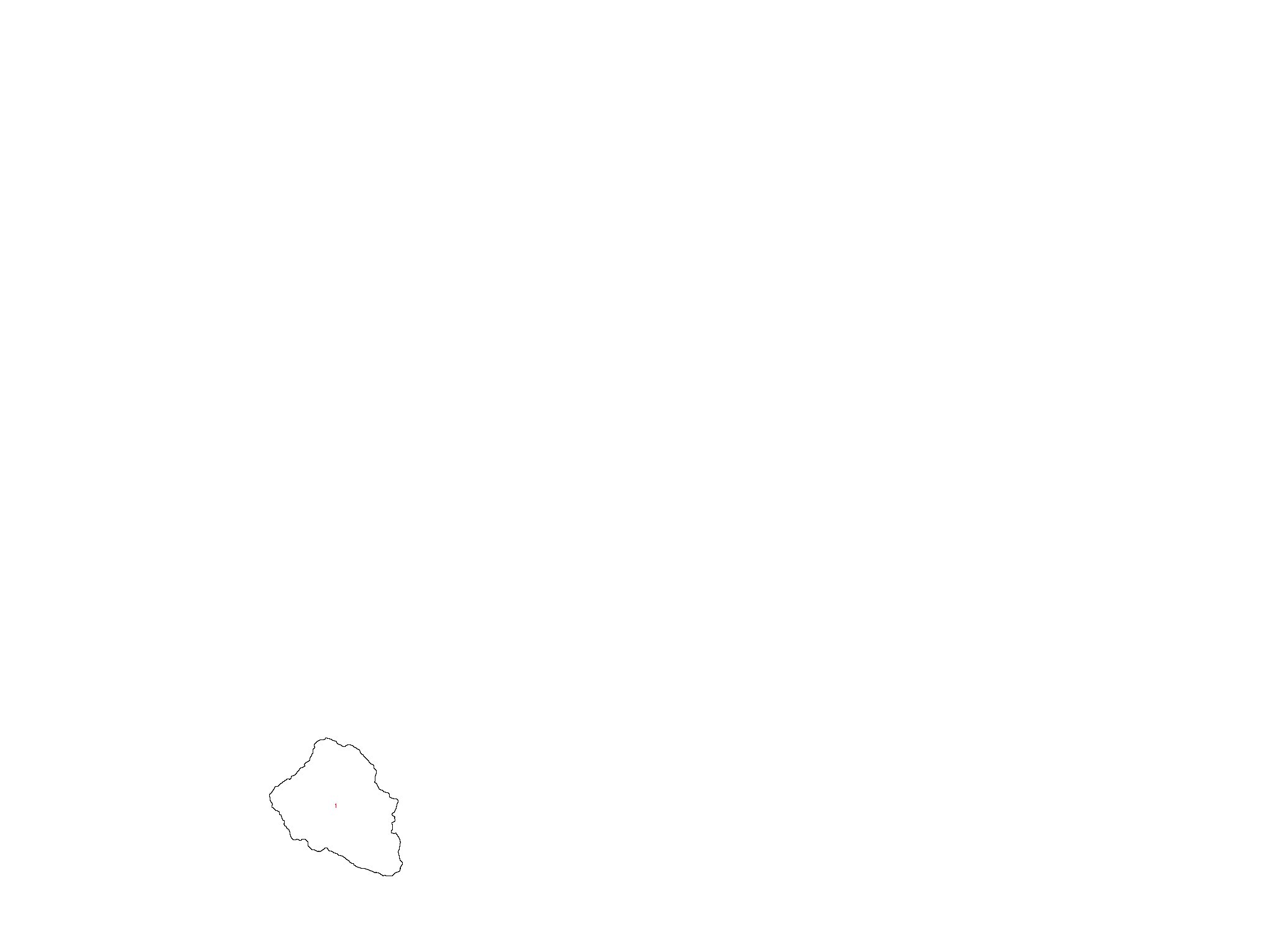

Supplement: S2 Dataset — (ZIP) [file pone.0304198.s005.zip › S2_Dataset_Raw_results_ImageJ/J7_100S_4050_1.jpg]

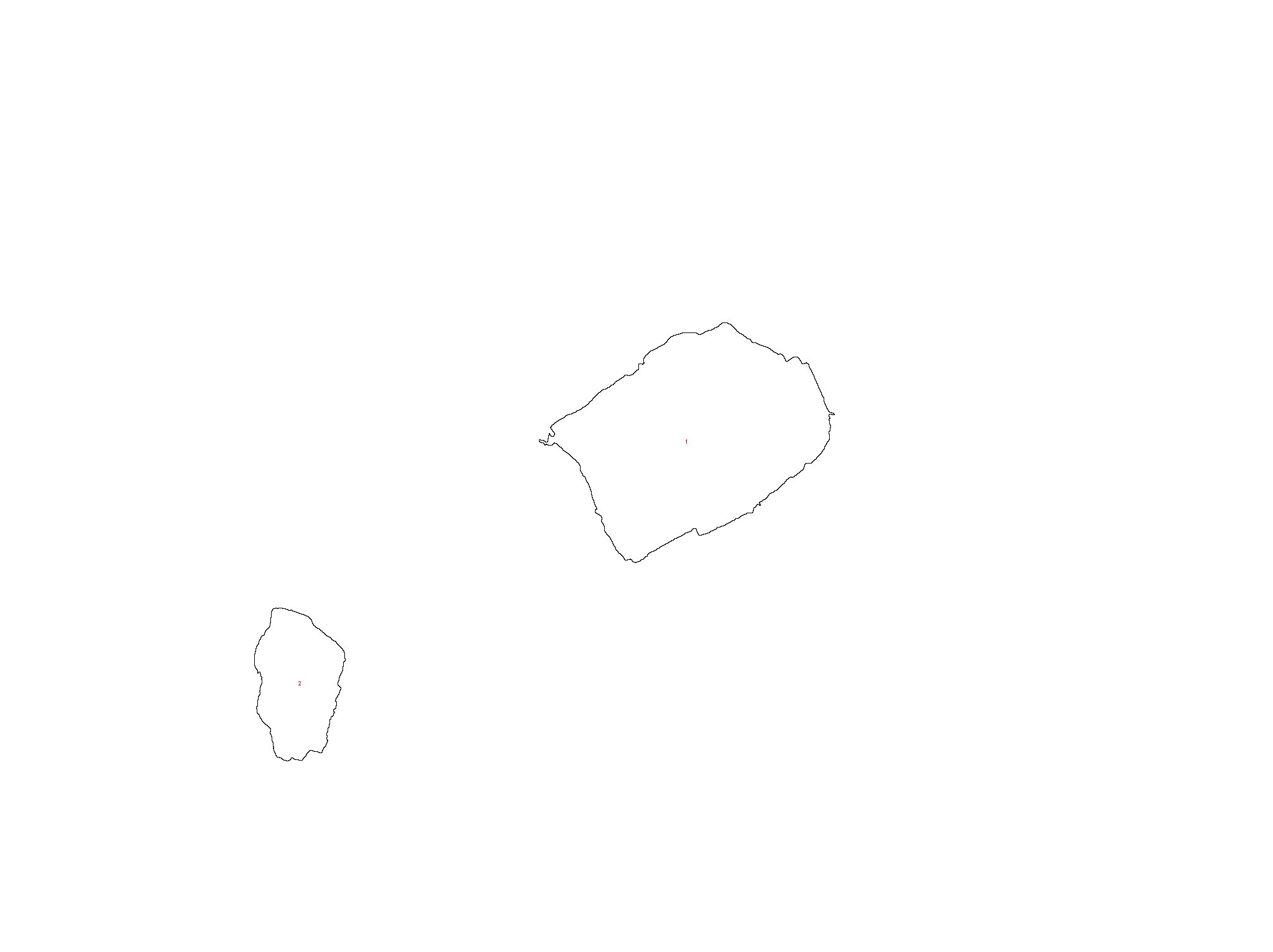

Supplement: S2 Dataset — (ZIP) [file pone.0304198.s005.zip › S2_Dataset_Raw_results_ImageJ/J7_100S_4050_2.jpg]

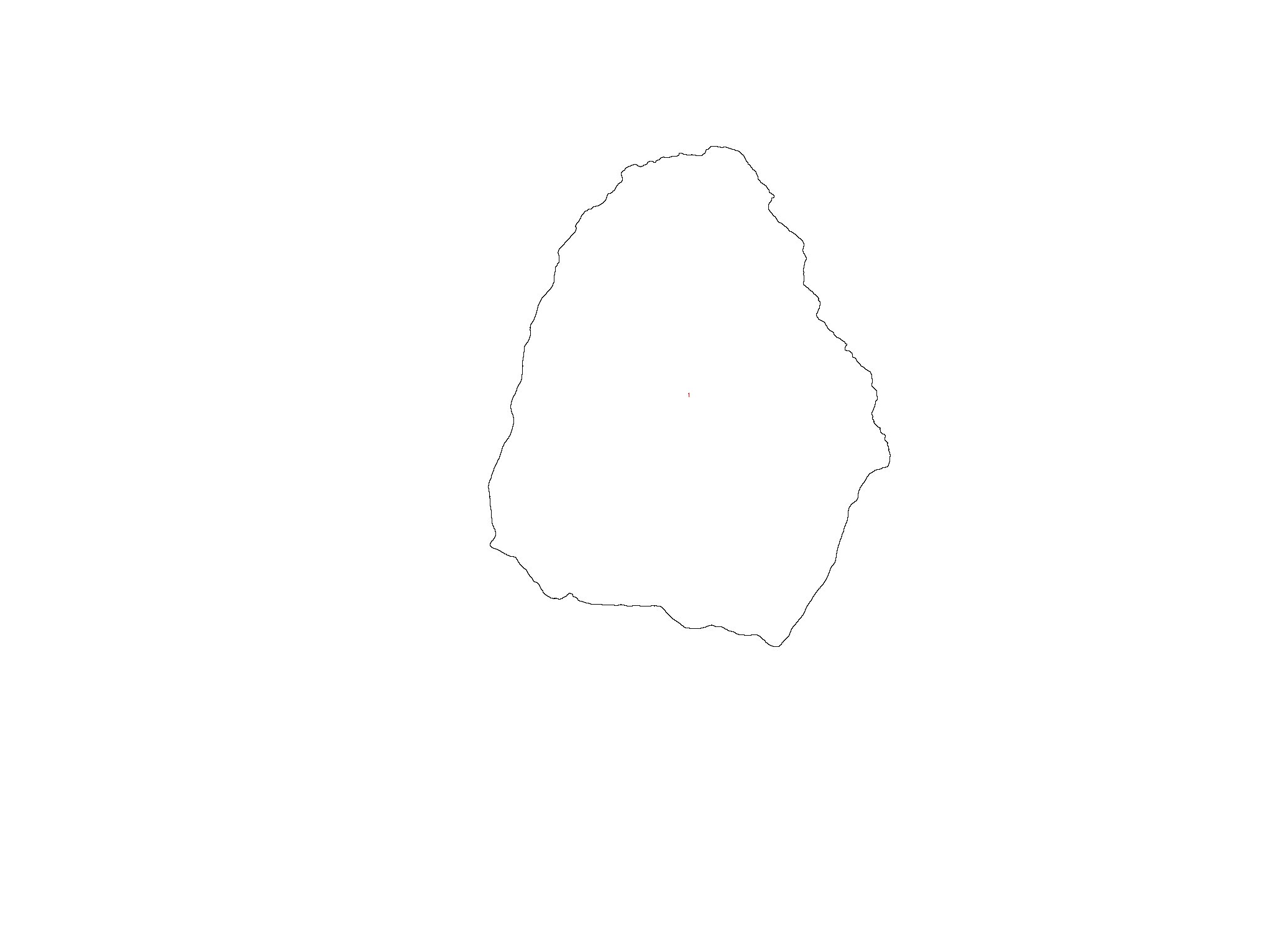

Supplement: S2 Dataset — (ZIP) [file pone.0304198.s005.zip › S2_Dataset_Raw_results_ImageJ/J7_100S_4050_3.jpg]

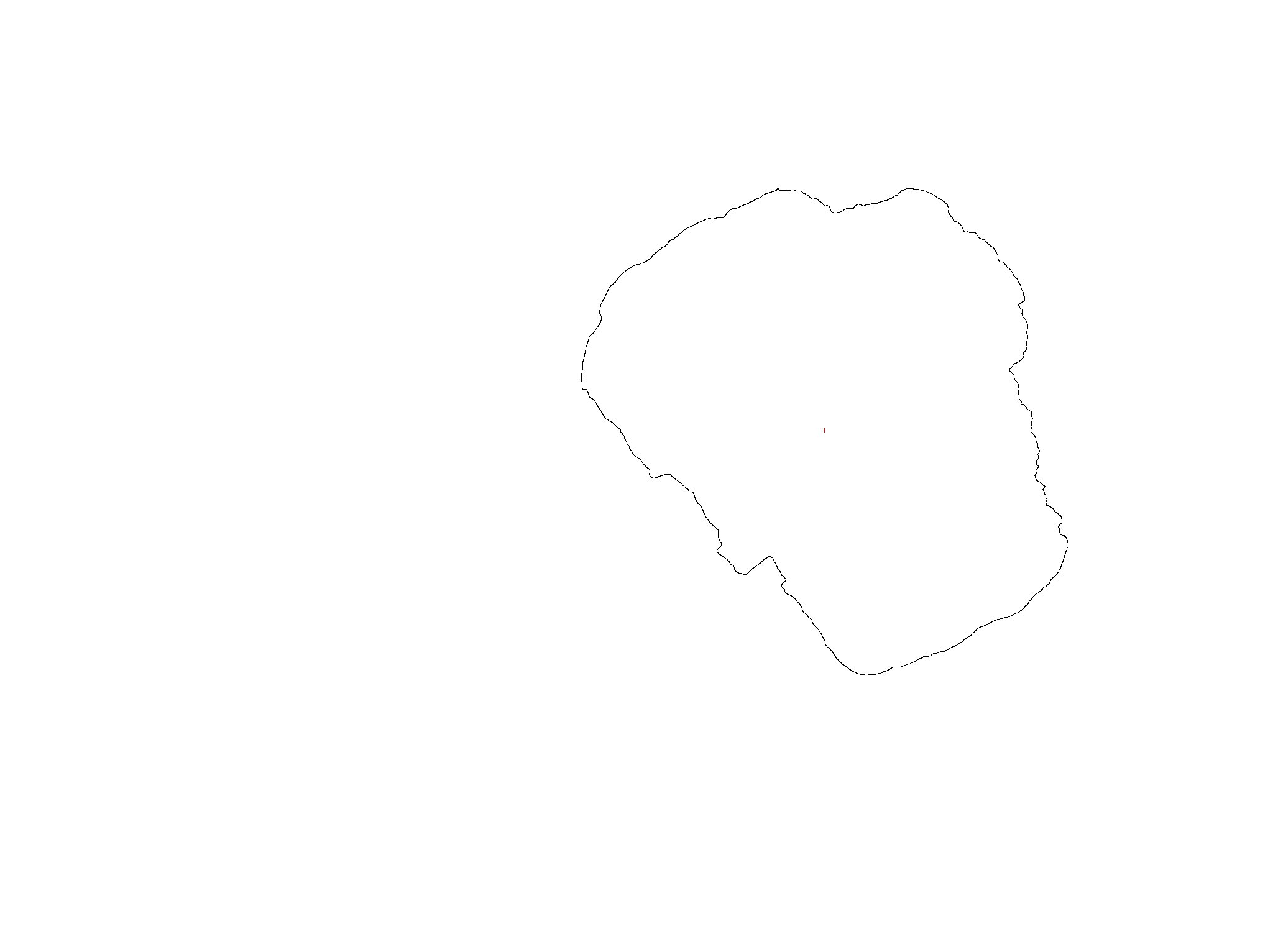

Supplement: S2 Dataset — (ZIP) [file pone.0304198.s005.zip › S2_Dataset_Raw_results_ImageJ/J7_100S_4050_4.jpg]

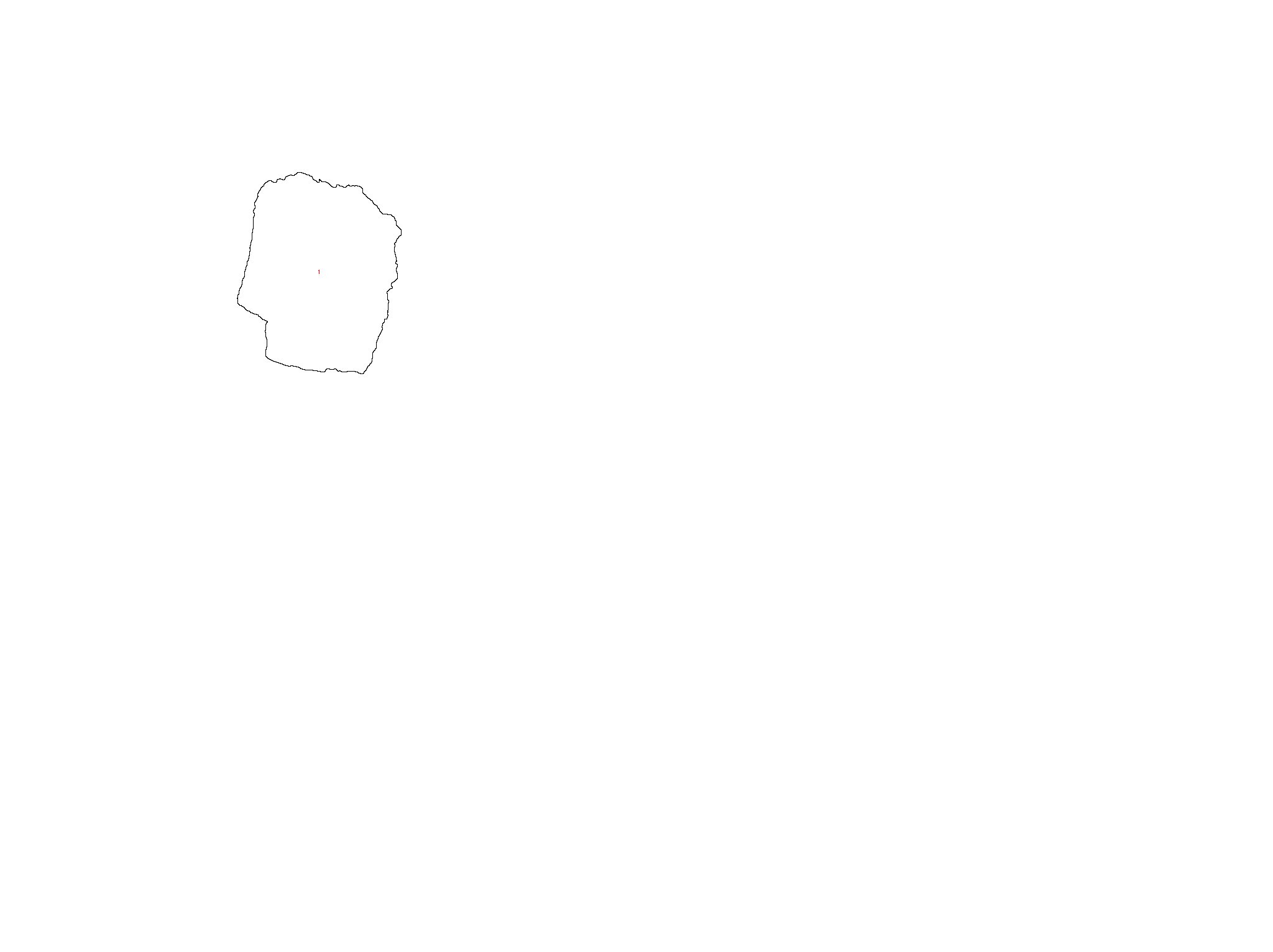

Supplement: S2 Dataset — (ZIP) [file pone.0304198.s005.zip › S2_Dataset_Raw_results_ImageJ/J7_100S_4050_5.jpg]

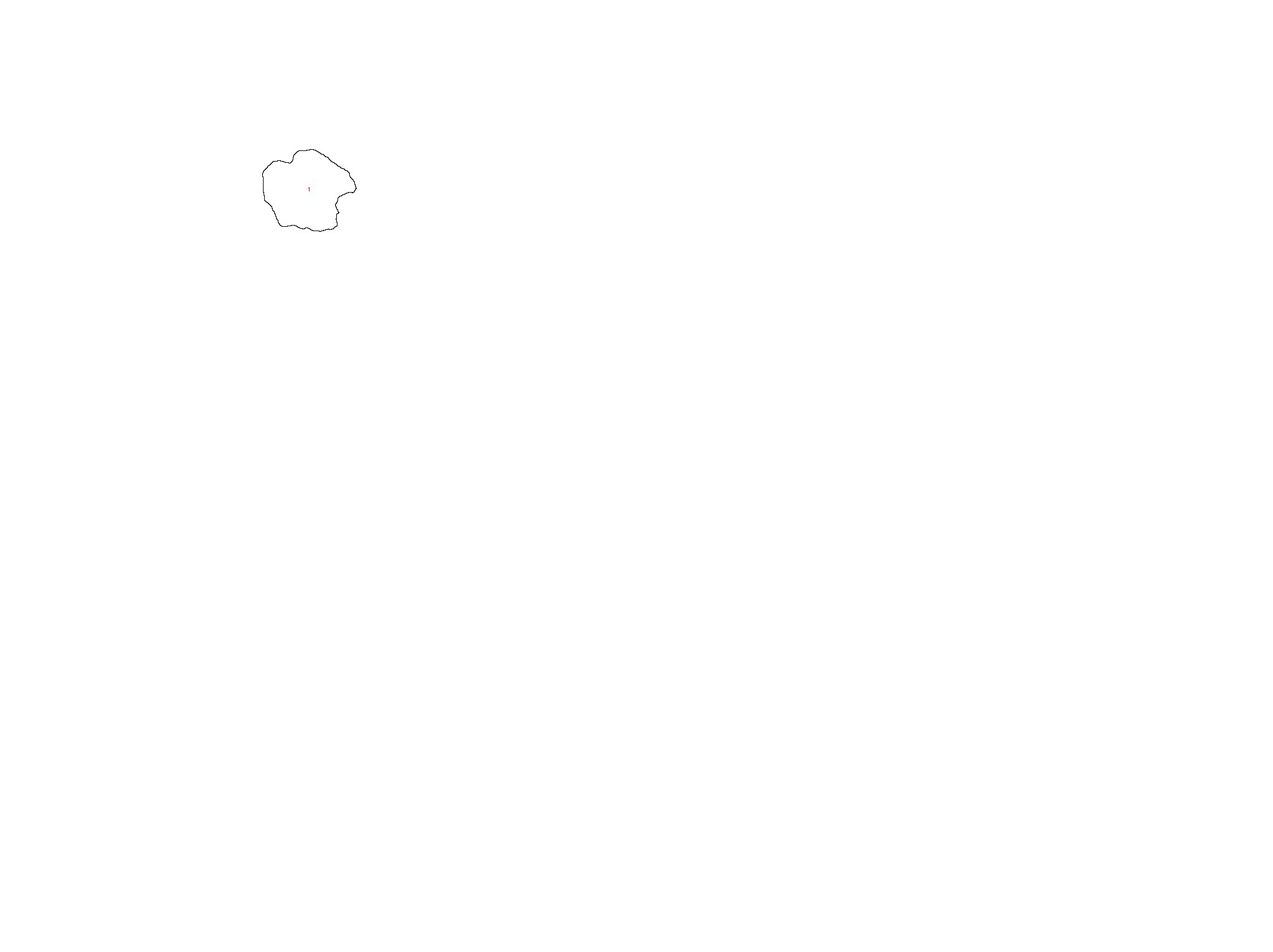

Supplement: S2 Dataset — (ZIP) [file pone.0304198.s005.zip › S2_Dataset_Raw_results_ImageJ/J7_100S_4050_6.jpg]

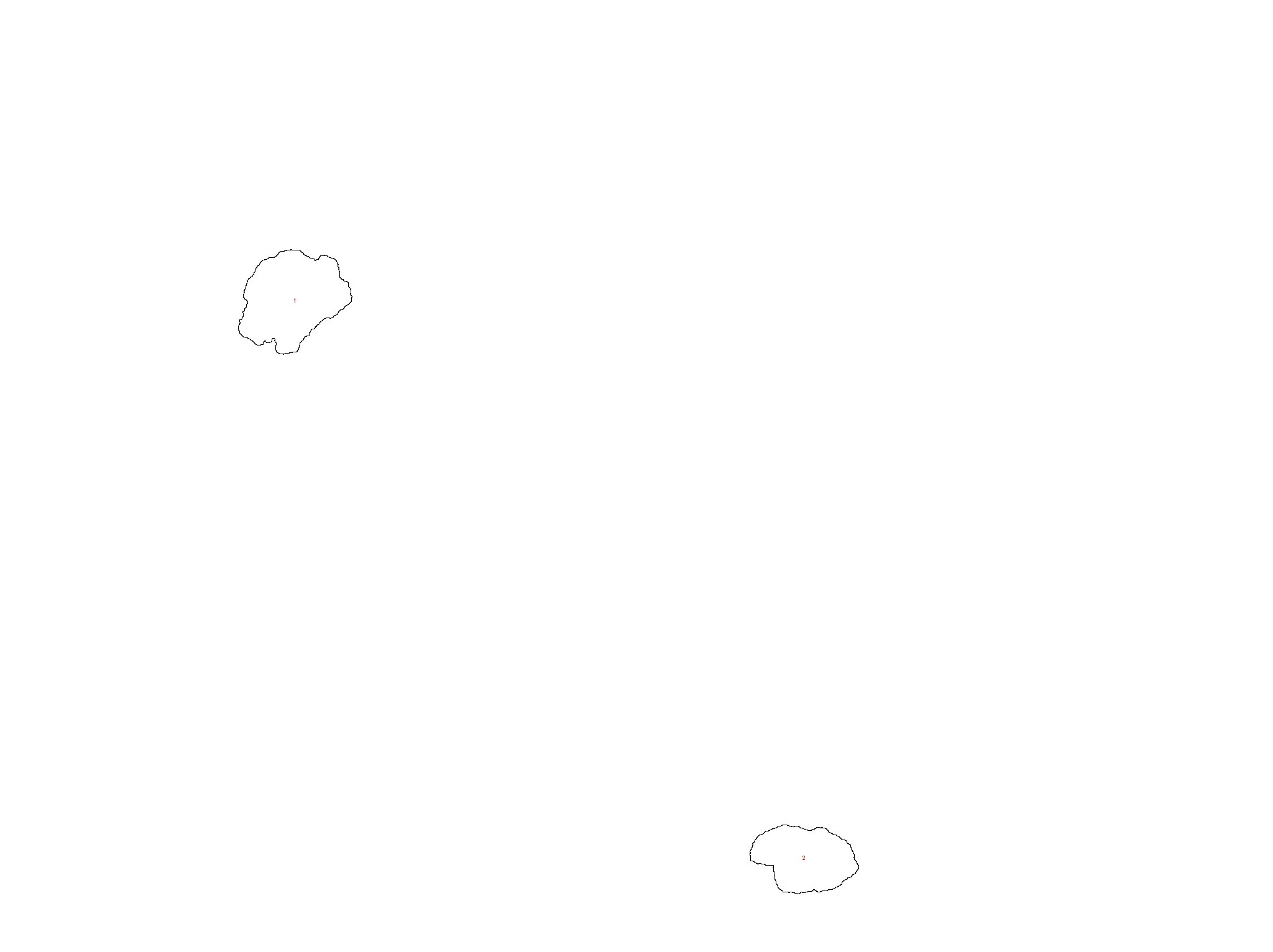

Supplement: S2 Dataset — (ZIP) [file pone.0304198.s005.zip › S2_Dataset_Raw_results_ImageJ/J7_100S_4050_7.jpg]

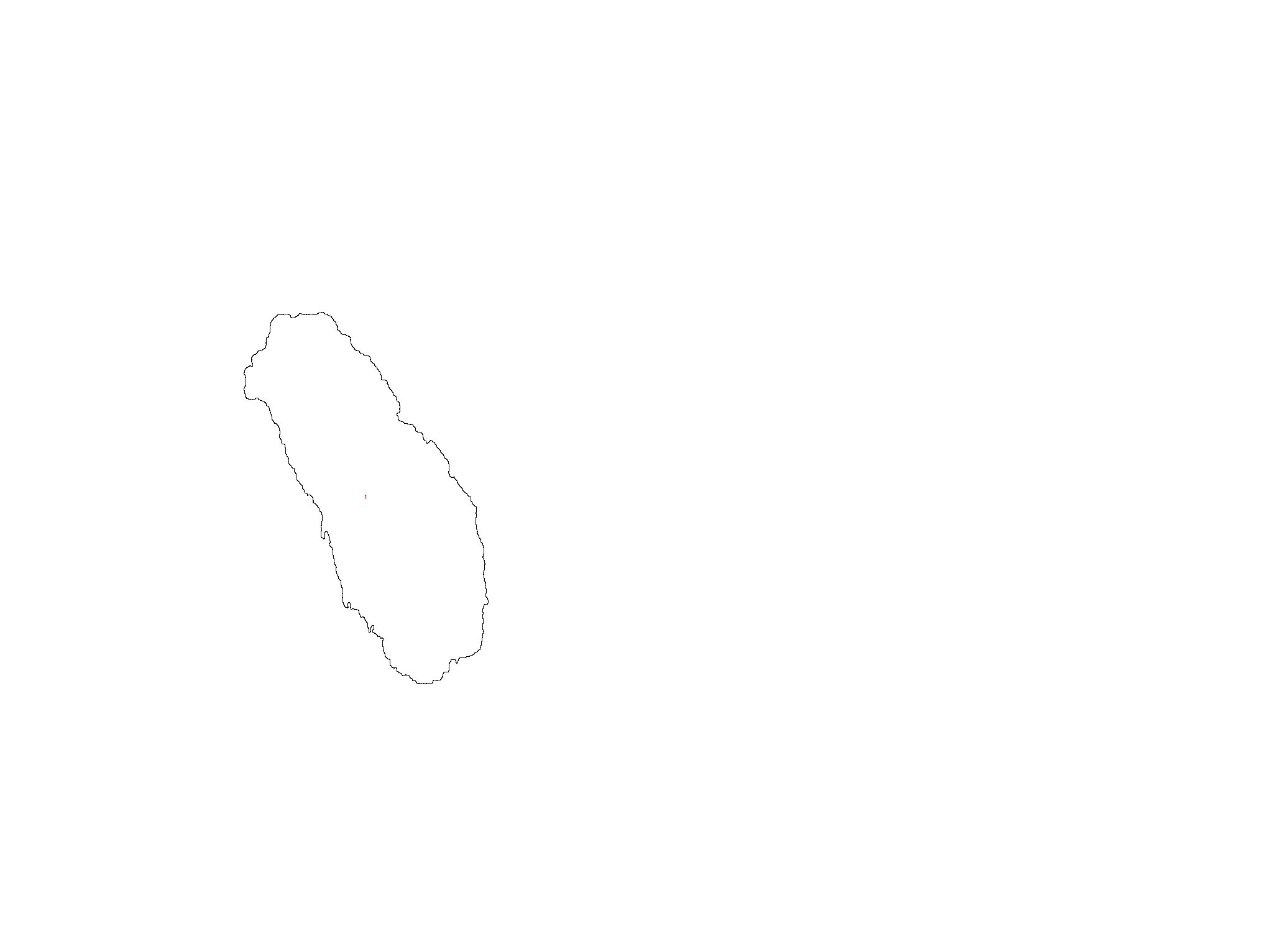

Supplement: S2 Dataset — (ZIP) [file pone.0304198.s005.zip › S2_Dataset_Raw_results_ImageJ/J7_100S_4050_8.jpg]

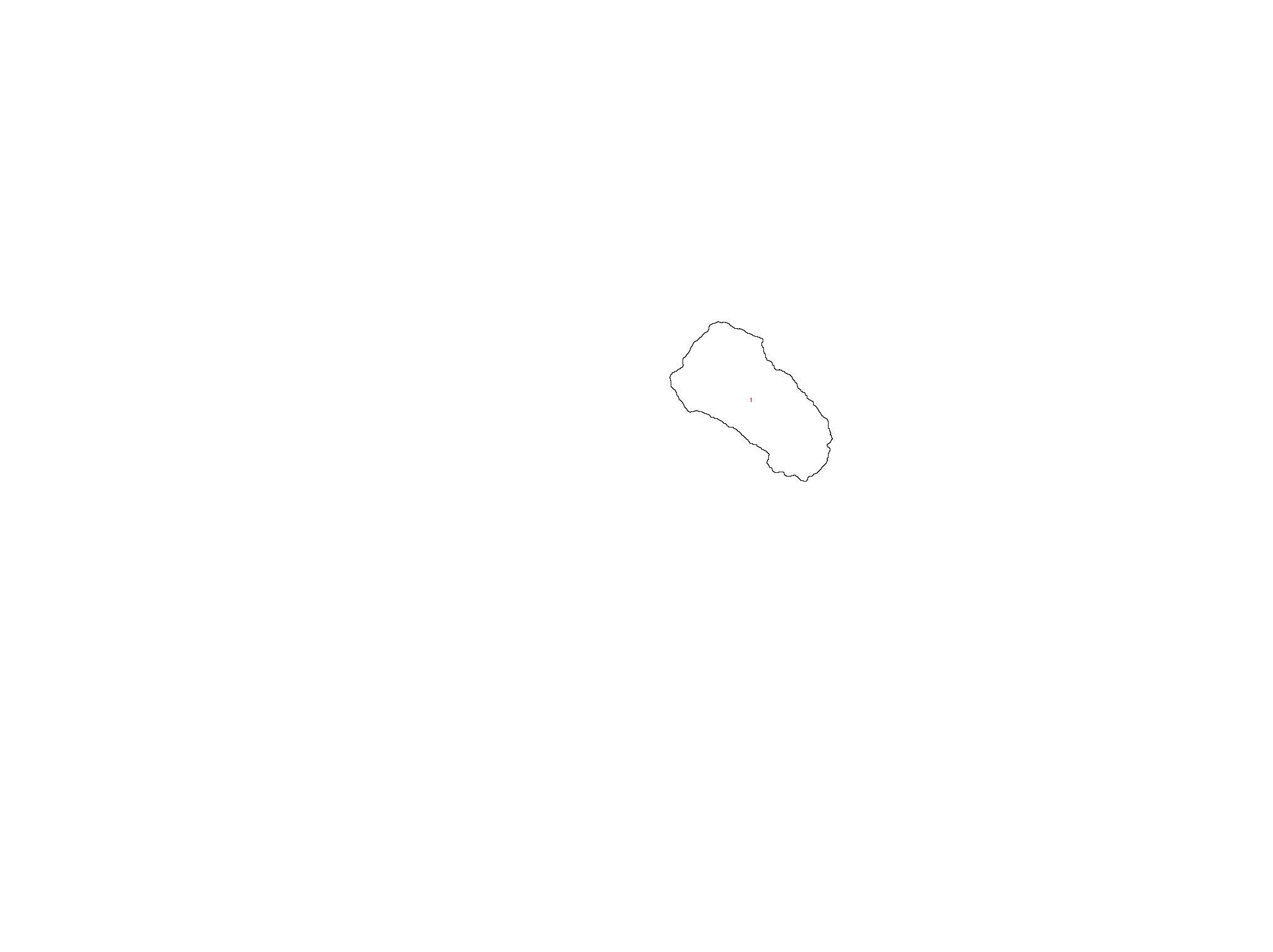

Supplement: S2 Dataset — (ZIP) [file pone.0304198.s005.zip › S2_Dataset_Raw_results_ImageJ/J7_200S_100110_1.jpg]
