# Supplementary figures and images for: Reproducible protocol for the extraction and semi-automated quantification of macroscopic charcoal from soil (part 1 of 2)
Source: PLoS One. 2024 Jul 12;19(7):e0304198. doi: 10.1371/journal.pone.0304198 (PMC11244820; doi:10.1371/journal.pone.0304198)

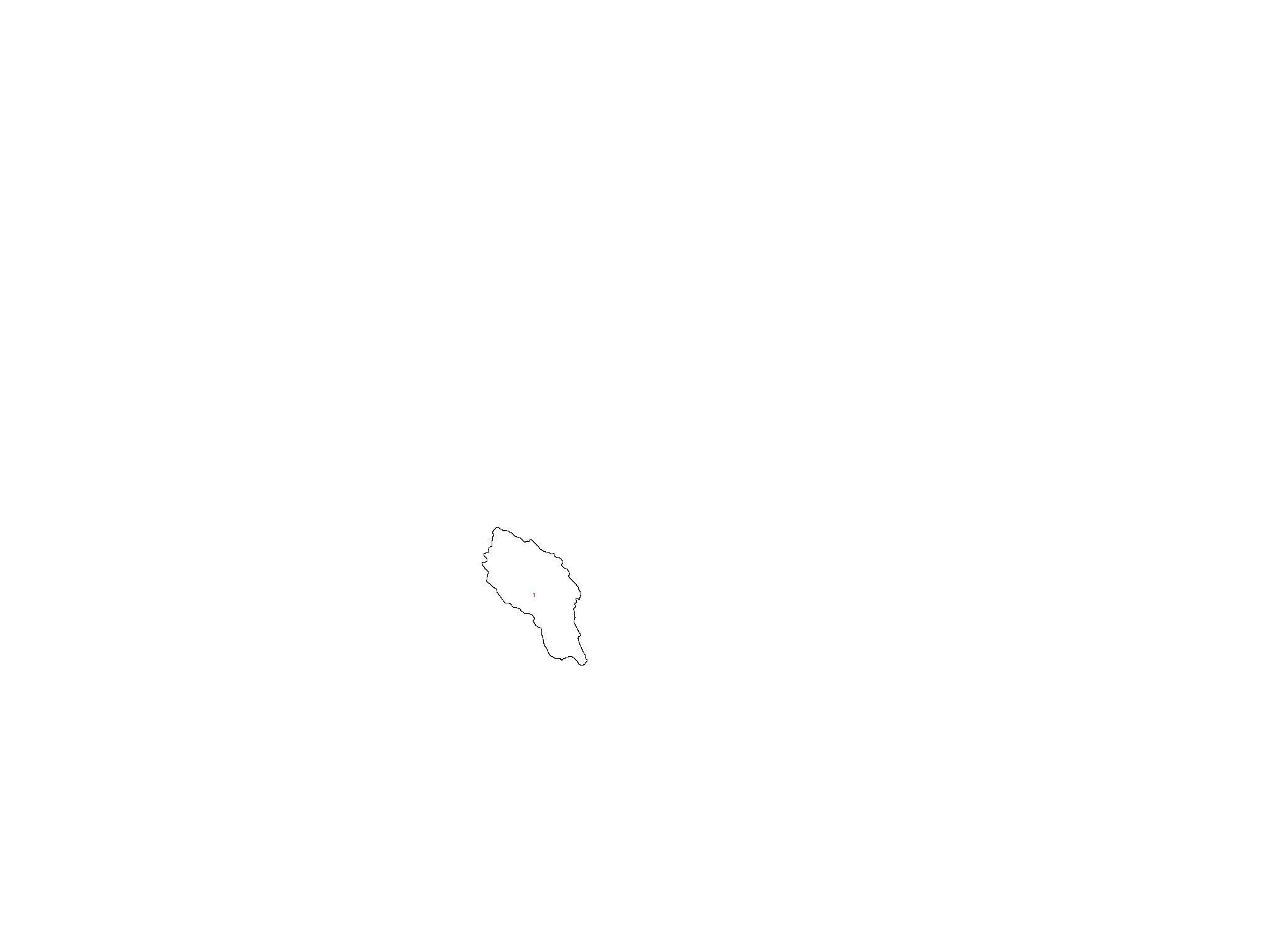

Supplement: S2 Dataset — (ZIP) [file pone.0304198.s005.zip › S2_Dataset_Raw_results_ImageJ/J2_0E_1020_1.jpg]

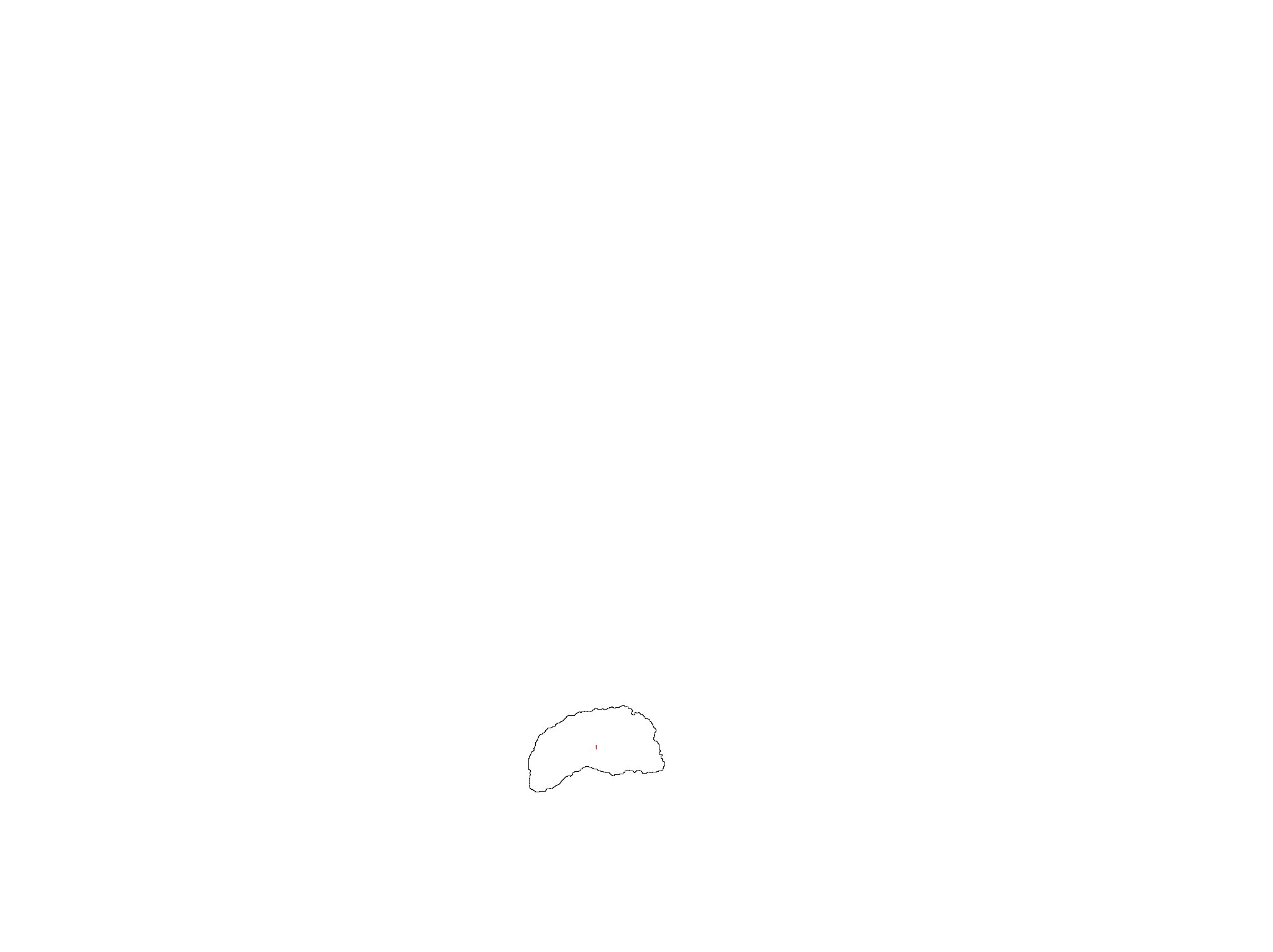

Supplement: S2 Dataset — (ZIP) [file pone.0304198.s005.zip › S2_Dataset_Raw_results_ImageJ/J2_0E_1020_2.jpg]

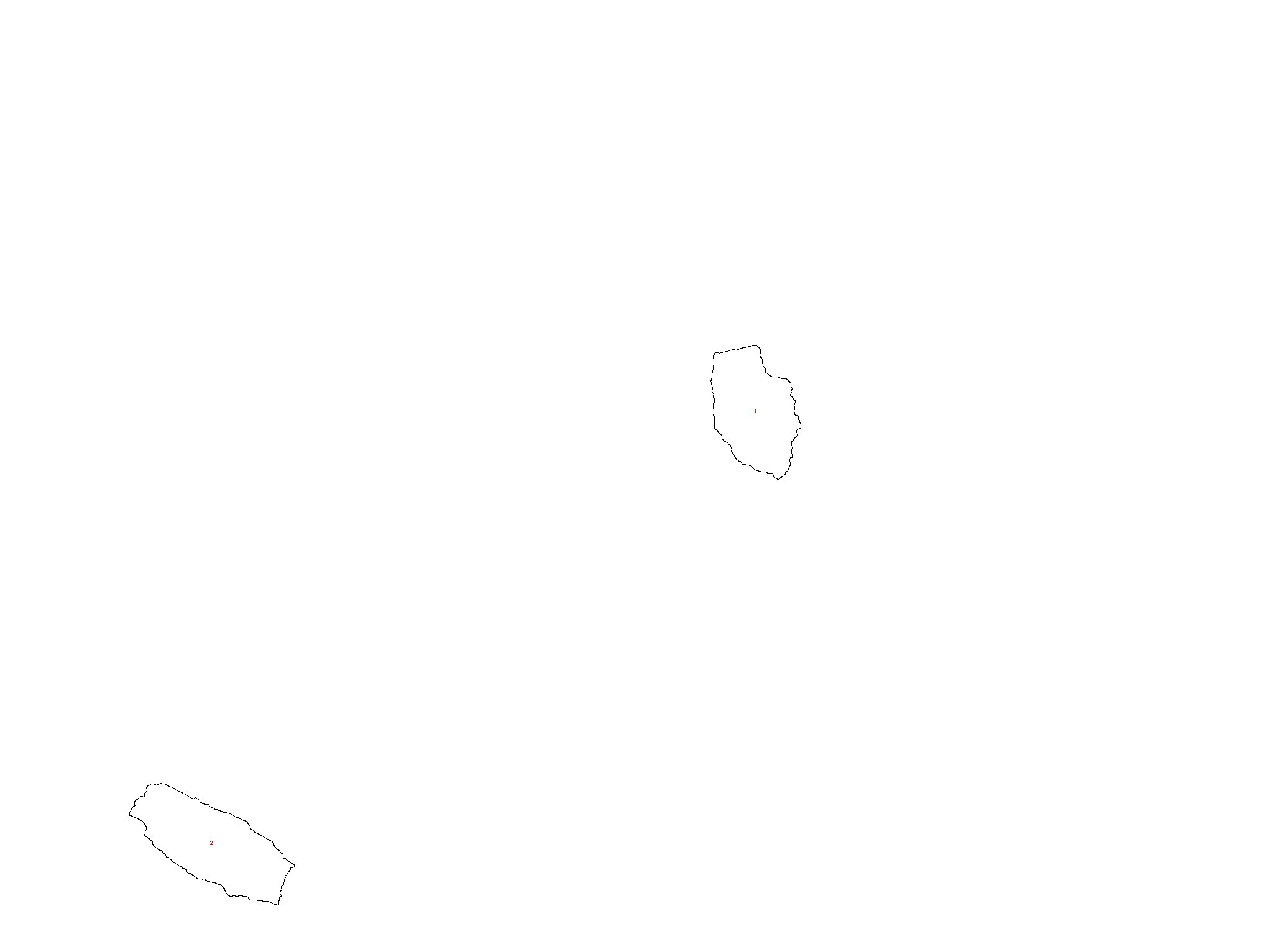

Supplement: S2 Dataset — (ZIP) [file pone.0304198.s005.zip › S2_Dataset_Raw_results_ImageJ/J2_0E_1020_3.jpg]

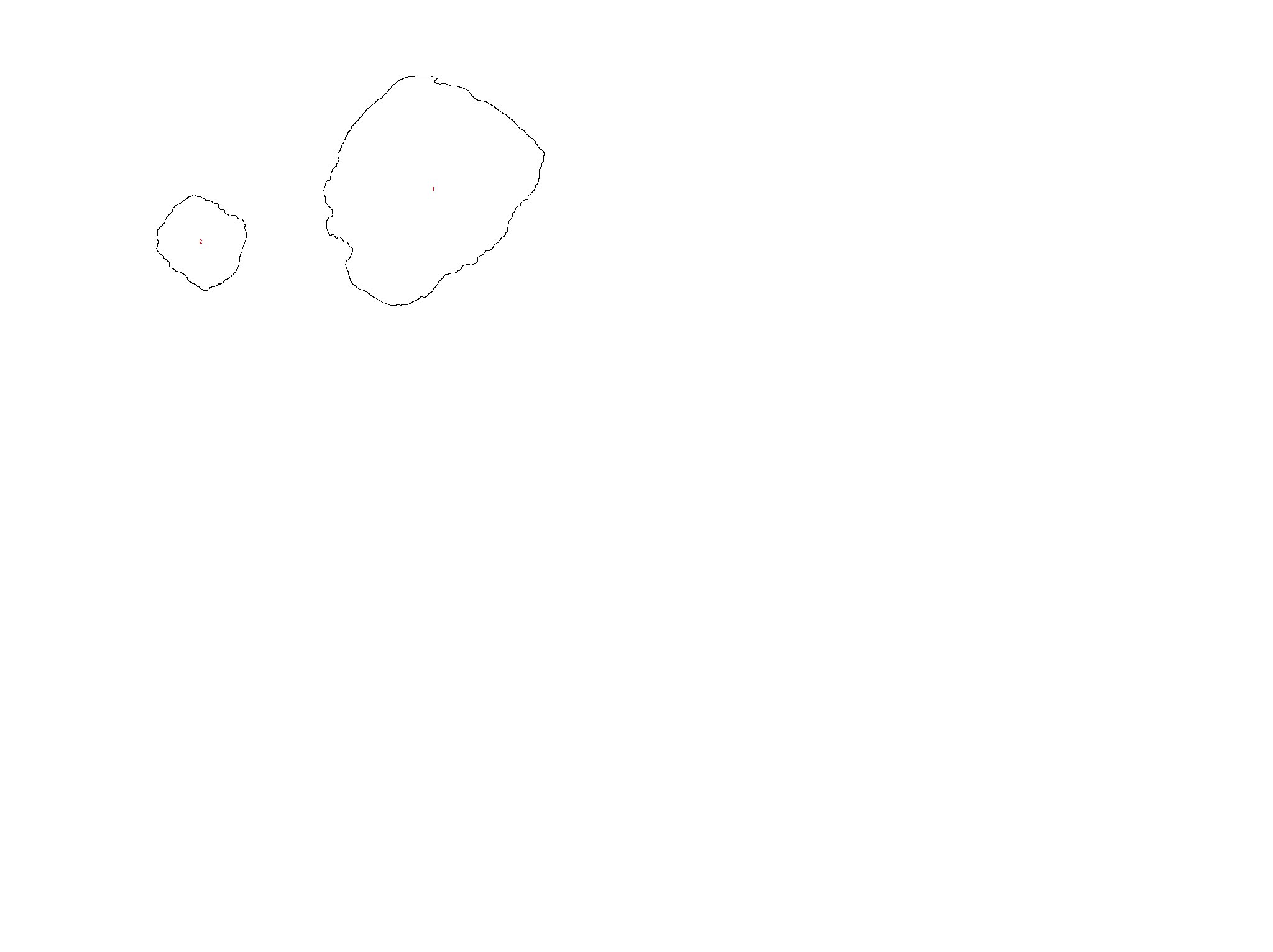

Supplement: S2 Dataset — (ZIP) [file pone.0304198.s005.zip › S2_Dataset_Raw_results_ImageJ/J2_0E_1020_4.jpg]

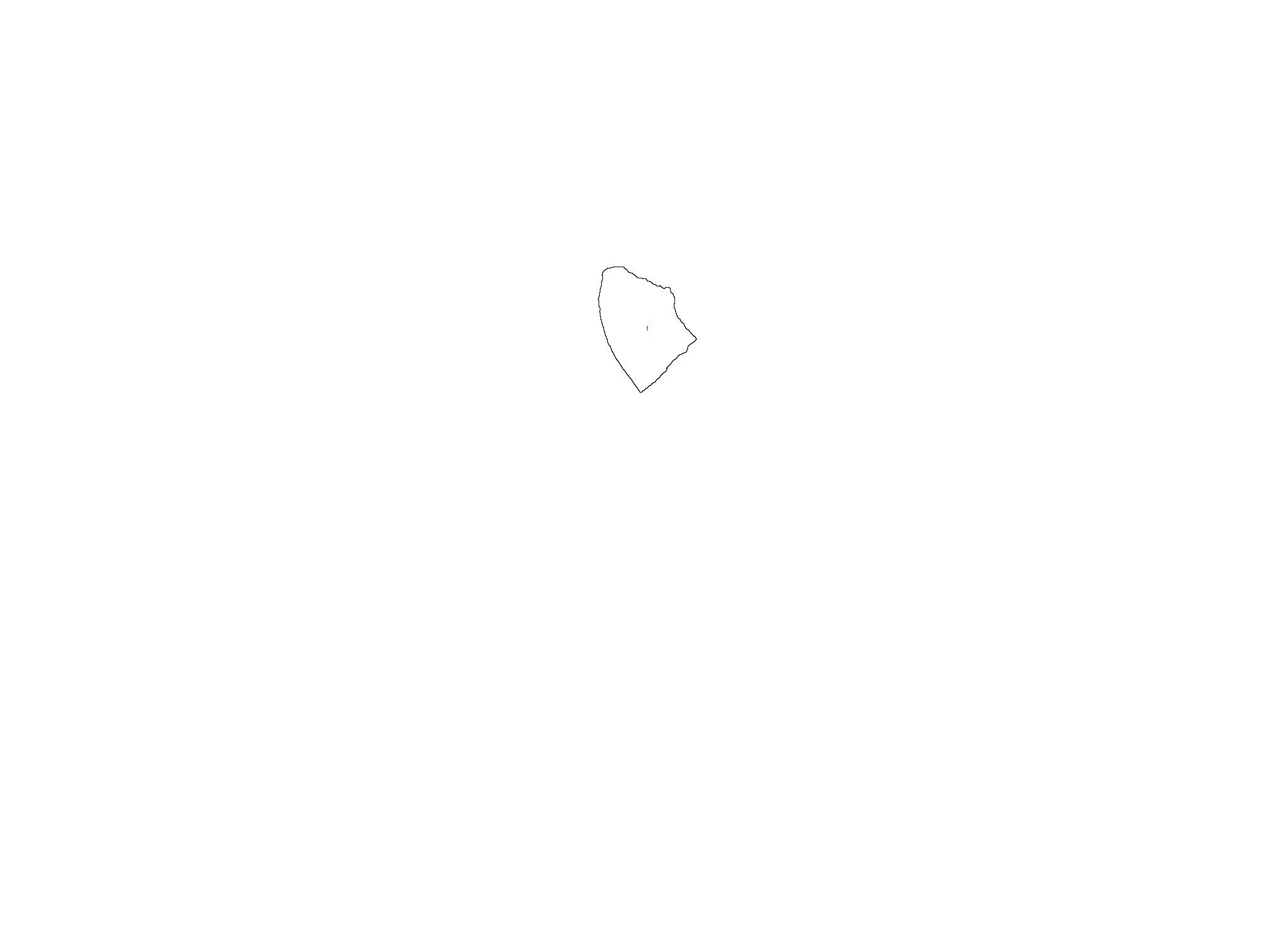

Supplement: S2 Dataset — (ZIP) [file pone.0304198.s005.zip › S2_Dataset_Raw_results_ImageJ/J2_0E_1020_5.jpg]

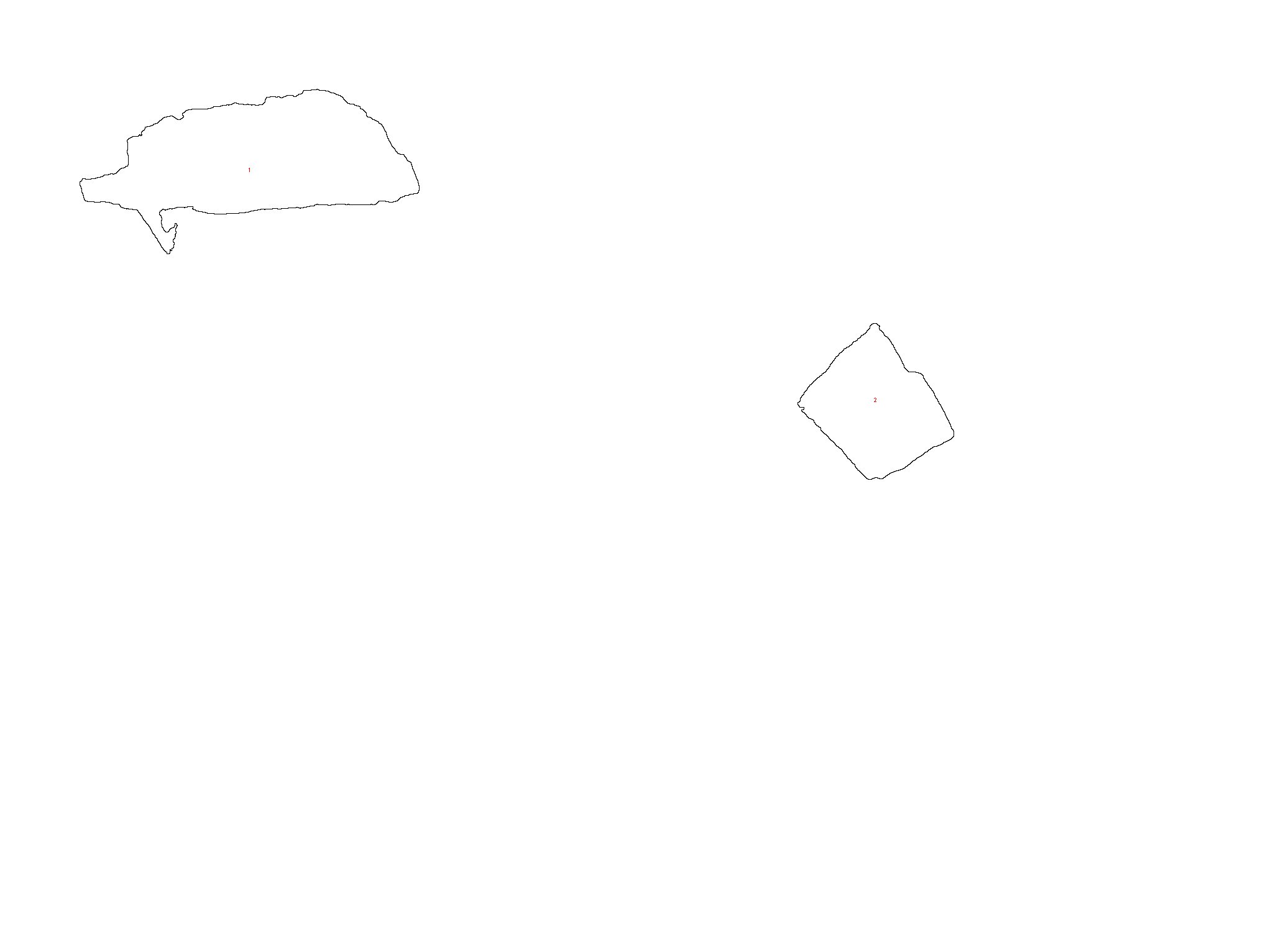

Supplement: S2 Dataset — (ZIP) [file pone.0304198.s005.zip › S2_Dataset_Raw_results_ImageJ/J2_0E_1020_6.jpg]

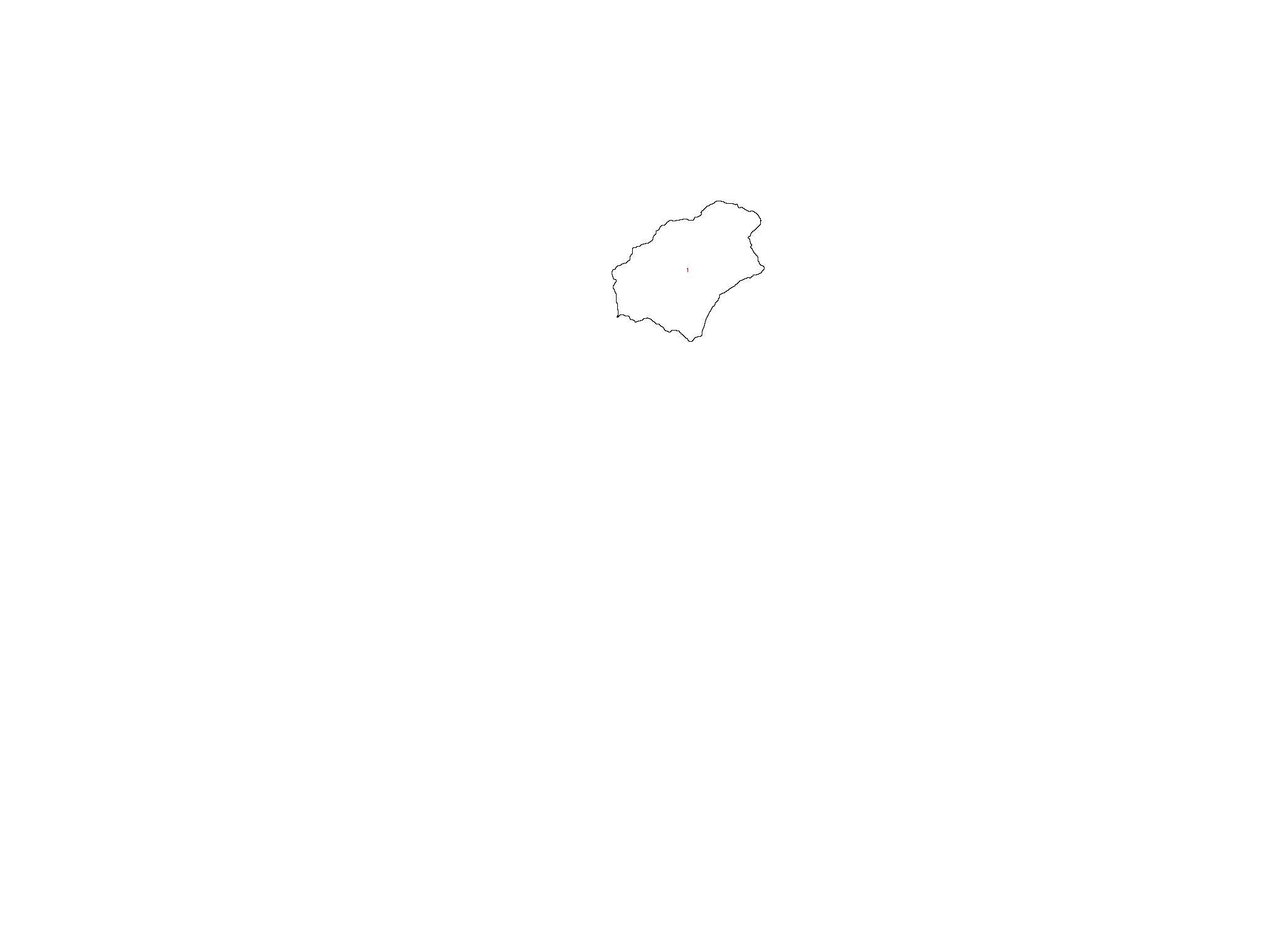

Supplement: S2 Dataset — (ZIP) [file pone.0304198.s005.zip › S2_Dataset_Raw_results_ImageJ/J2_0E_110120_1.jpg]

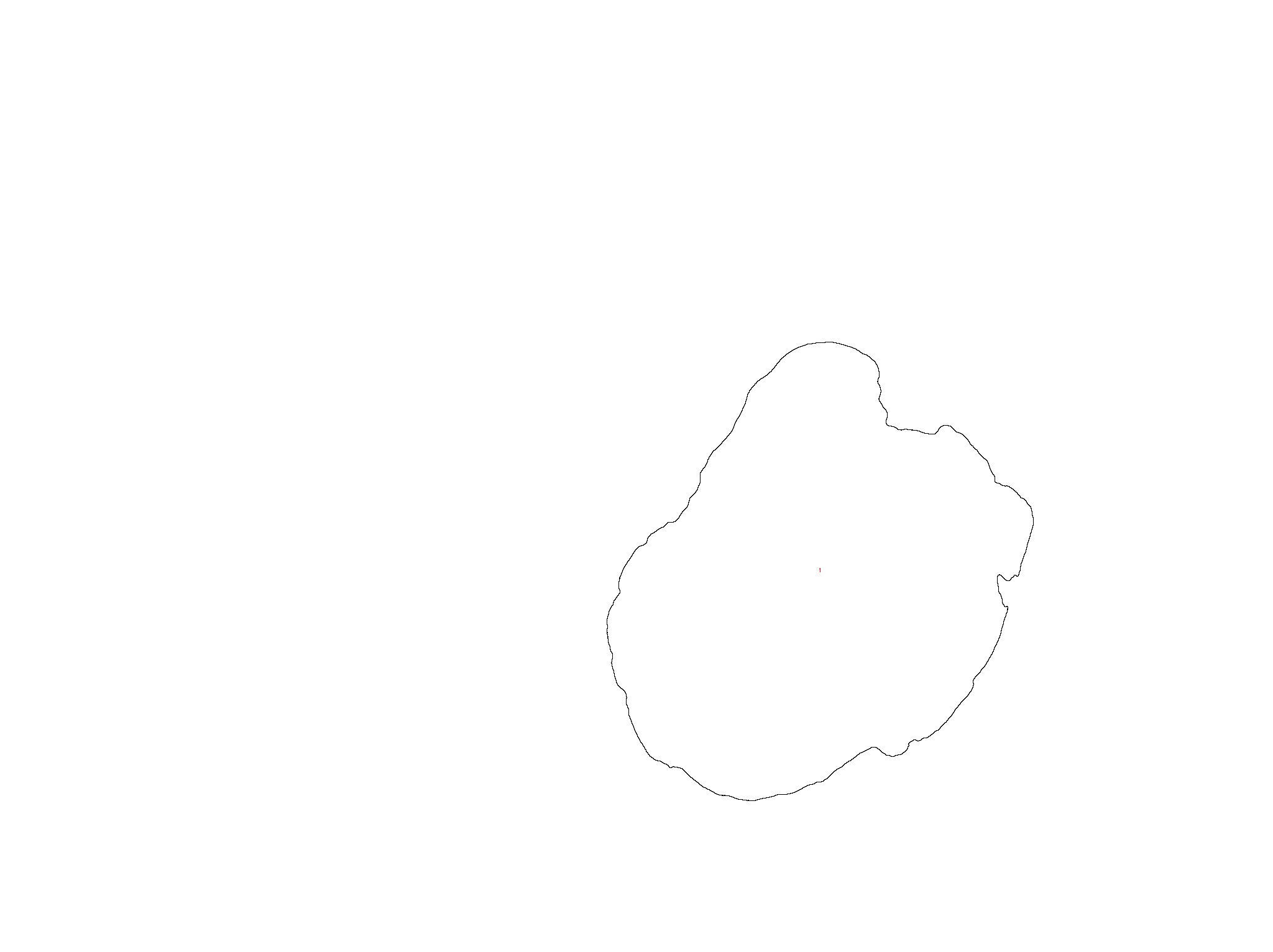

Supplement: S2 Dataset — (ZIP) [file pone.0304198.s005.zip › S2_Dataset_Raw_results_ImageJ/J2_0E_130140_1.jpg]

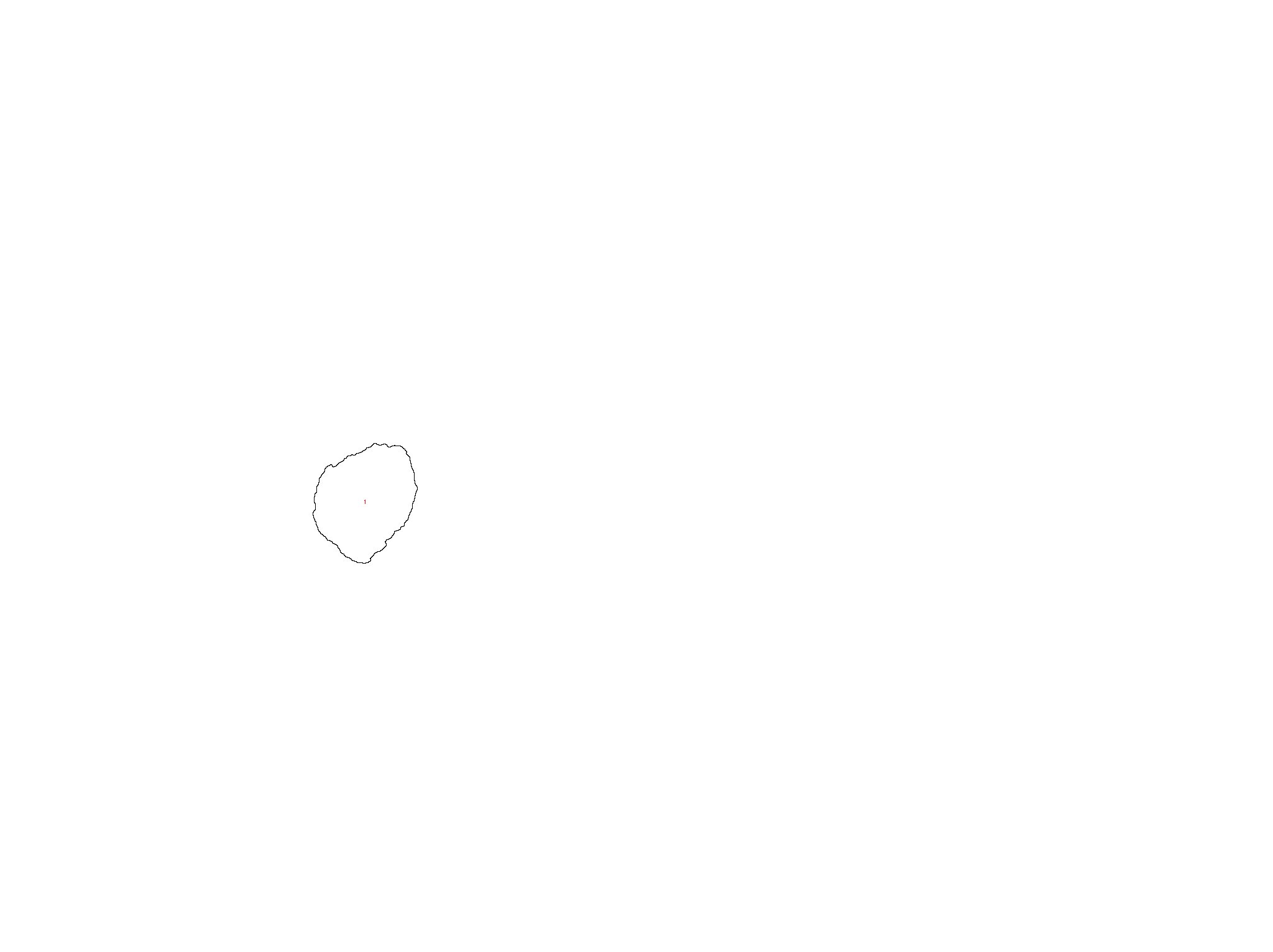

Supplement: S2 Dataset — (ZIP) [file pone.0304198.s005.zip › S2_Dataset_Raw_results_ImageJ/J2_0E_130140_2.jpg]

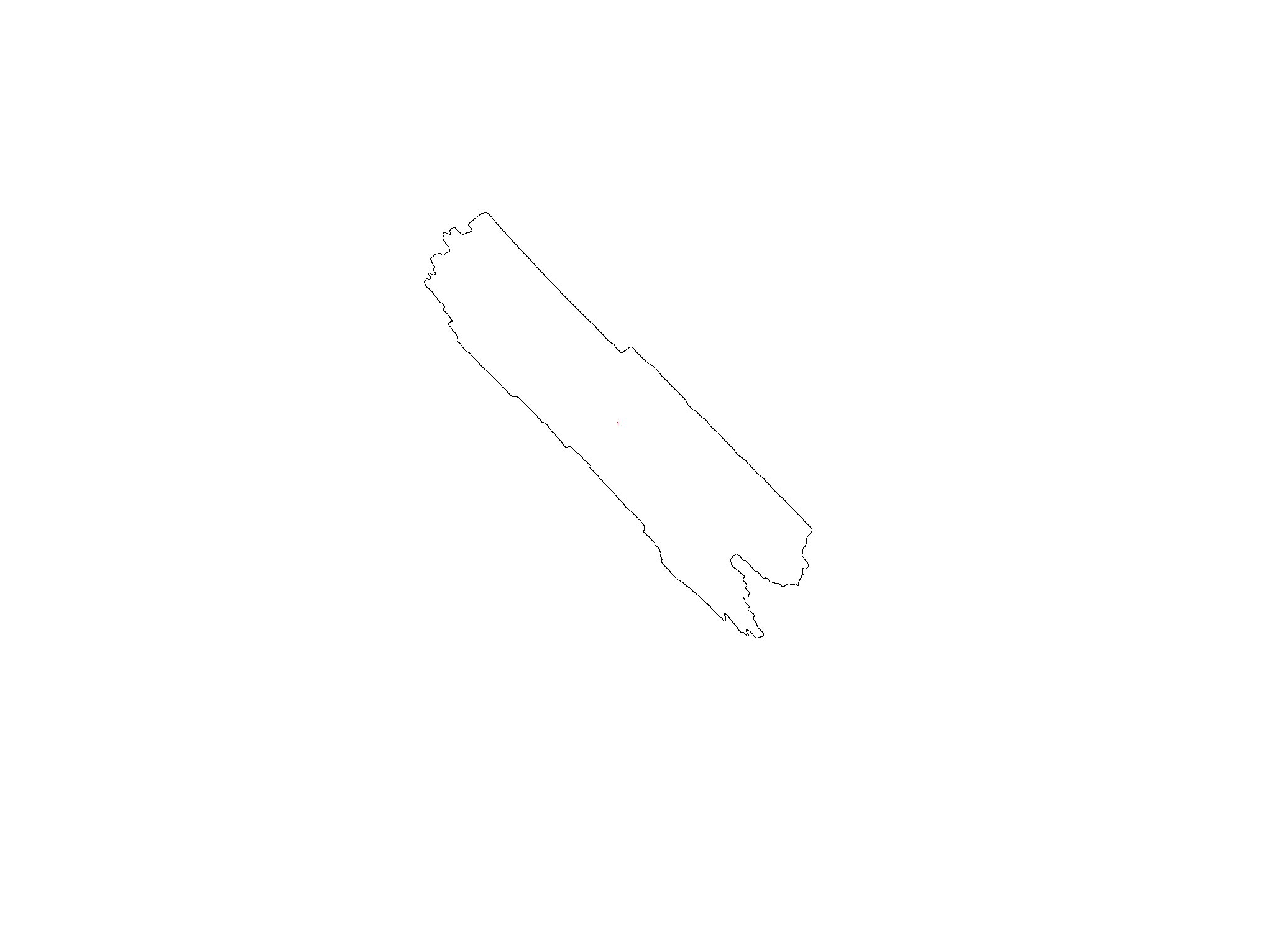

Supplement: S2 Dataset — (ZIP) [file pone.0304198.s005.zip › S2_Dataset_Raw_results_ImageJ/J2_0E_150160_1.jpg]

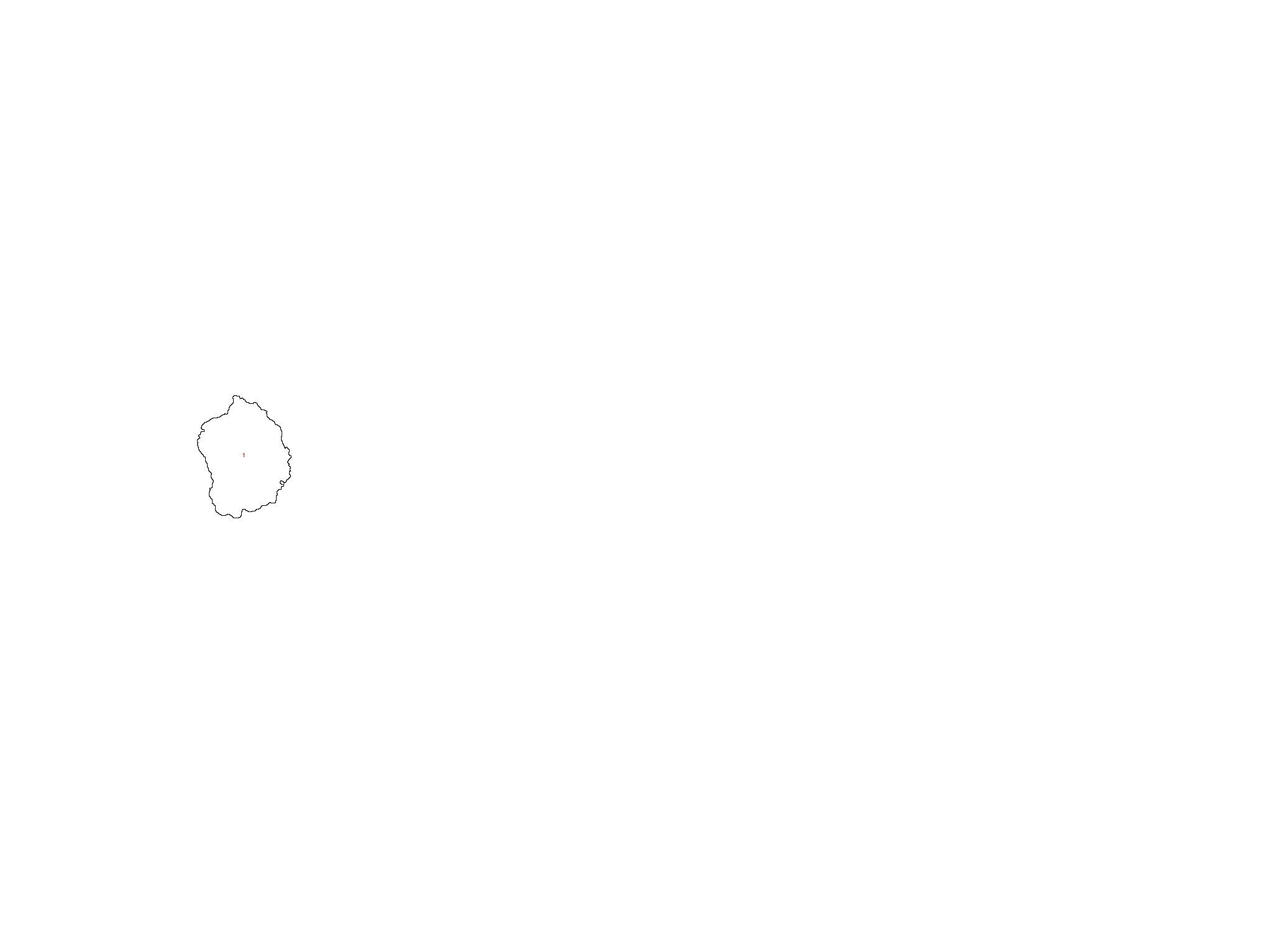

Supplement: S2 Dataset — (ZIP) [file pone.0304198.s005.zip › S2_Dataset_Raw_results_ImageJ/J2_0E_150160_2.jpg]

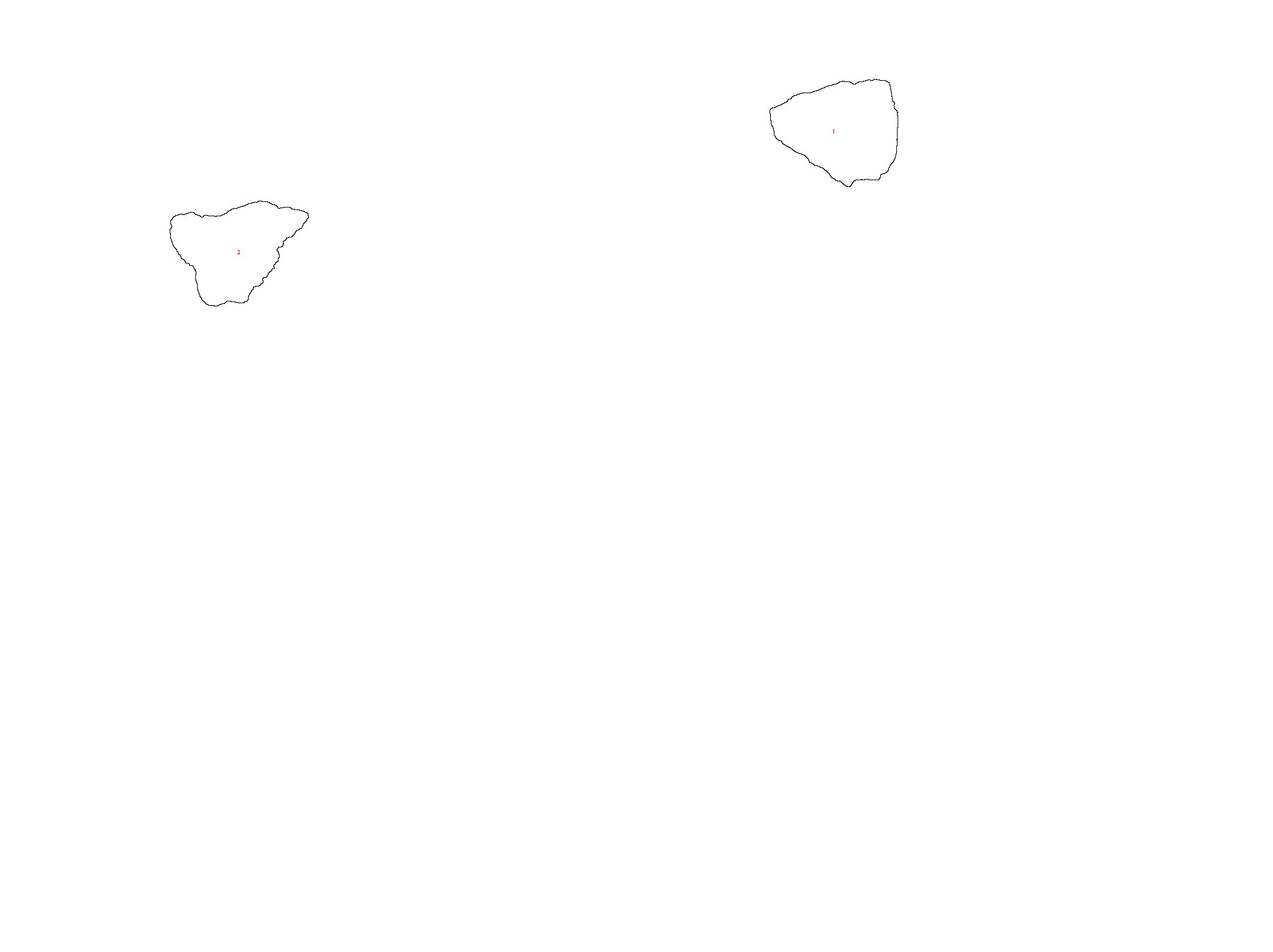

Supplement: S2 Dataset — (ZIP) [file pone.0304198.s005.zip › S2_Dataset_Raw_results_ImageJ/J2_0E_180190_1.jpg]

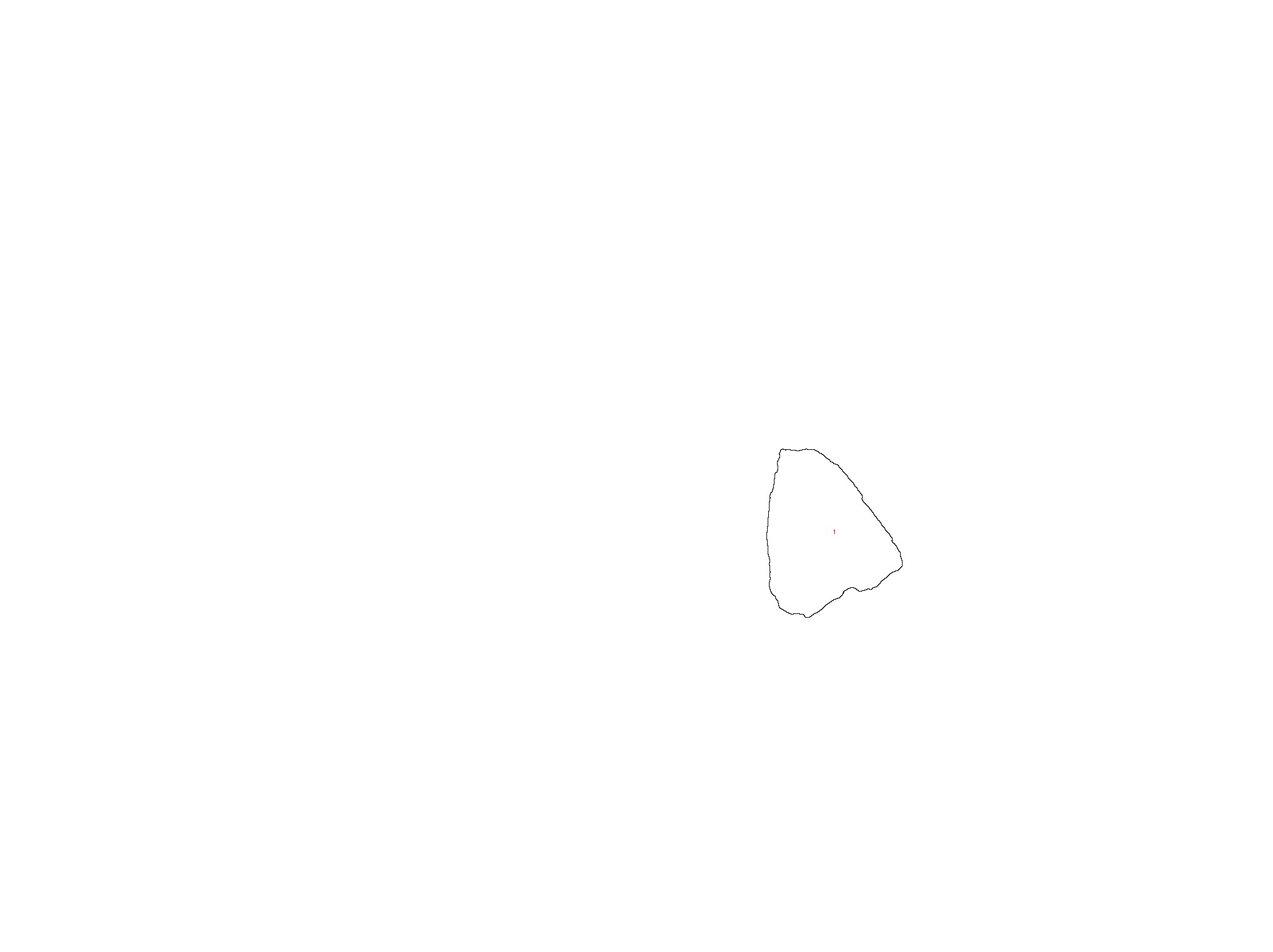

Supplement: S2 Dataset — (ZIP) [file pone.0304198.s005.zip › S2_Dataset_Raw_results_ImageJ/J2_0E_180190_2.jpg]

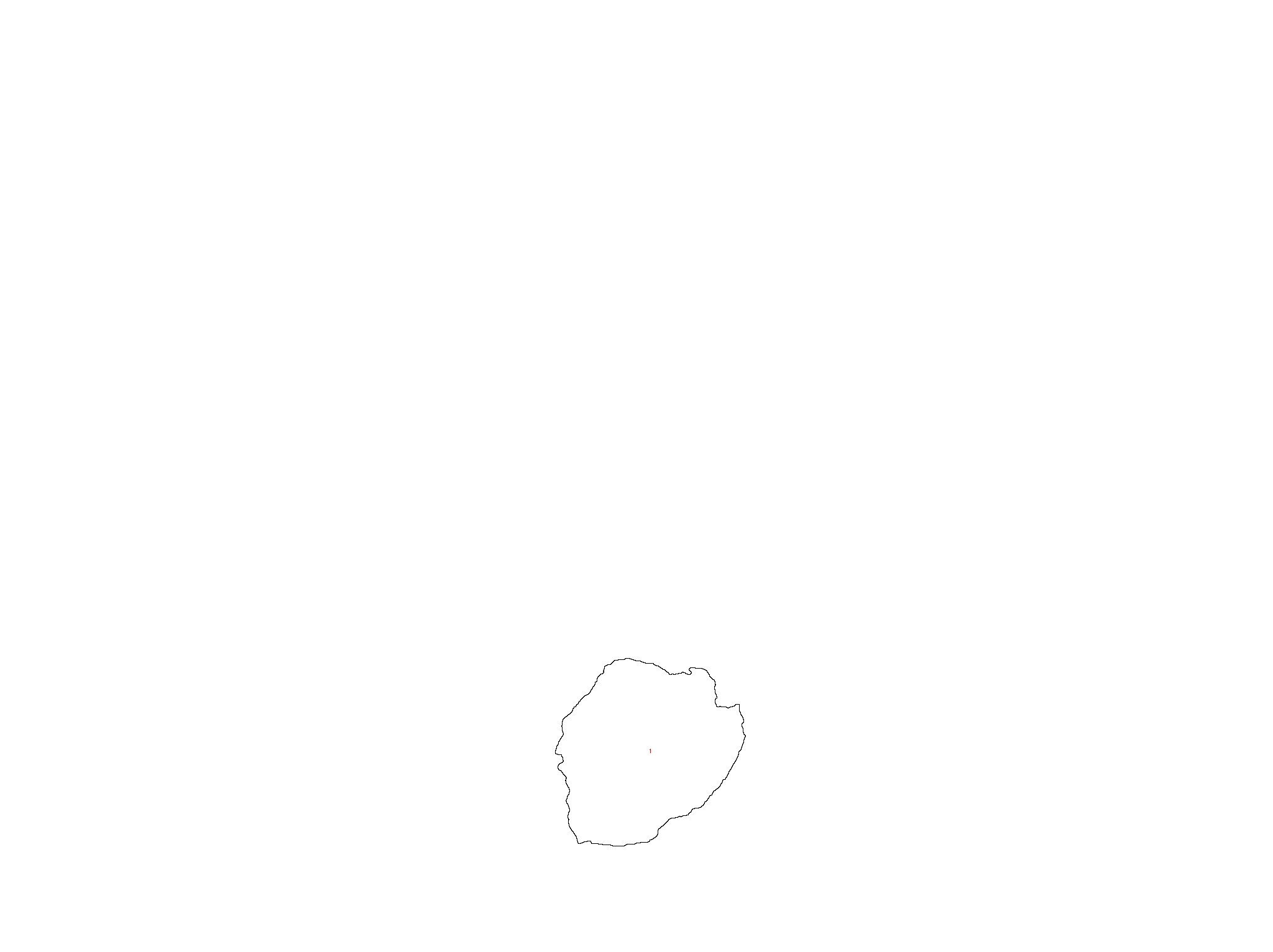

Supplement: S2 Dataset — (ZIP) [file pone.0304198.s005.zip › S2_Dataset_Raw_results_ImageJ/J2_0E_180190_3.jpg]

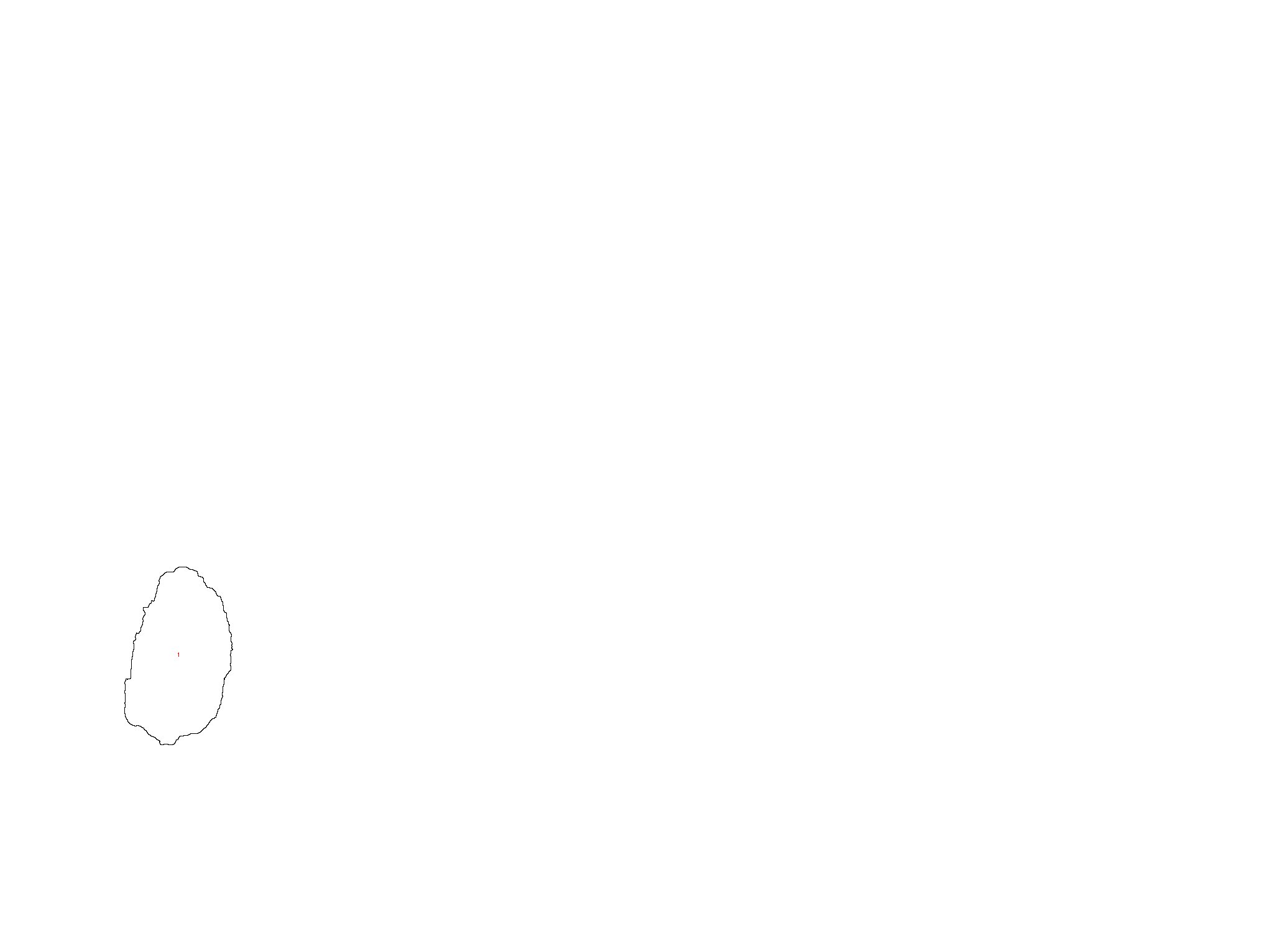

Supplement: S2 Dataset — (ZIP) [file pone.0304198.s005.zip › S2_Dataset_Raw_results_ImageJ/J2_0E_180190_4.jpg]

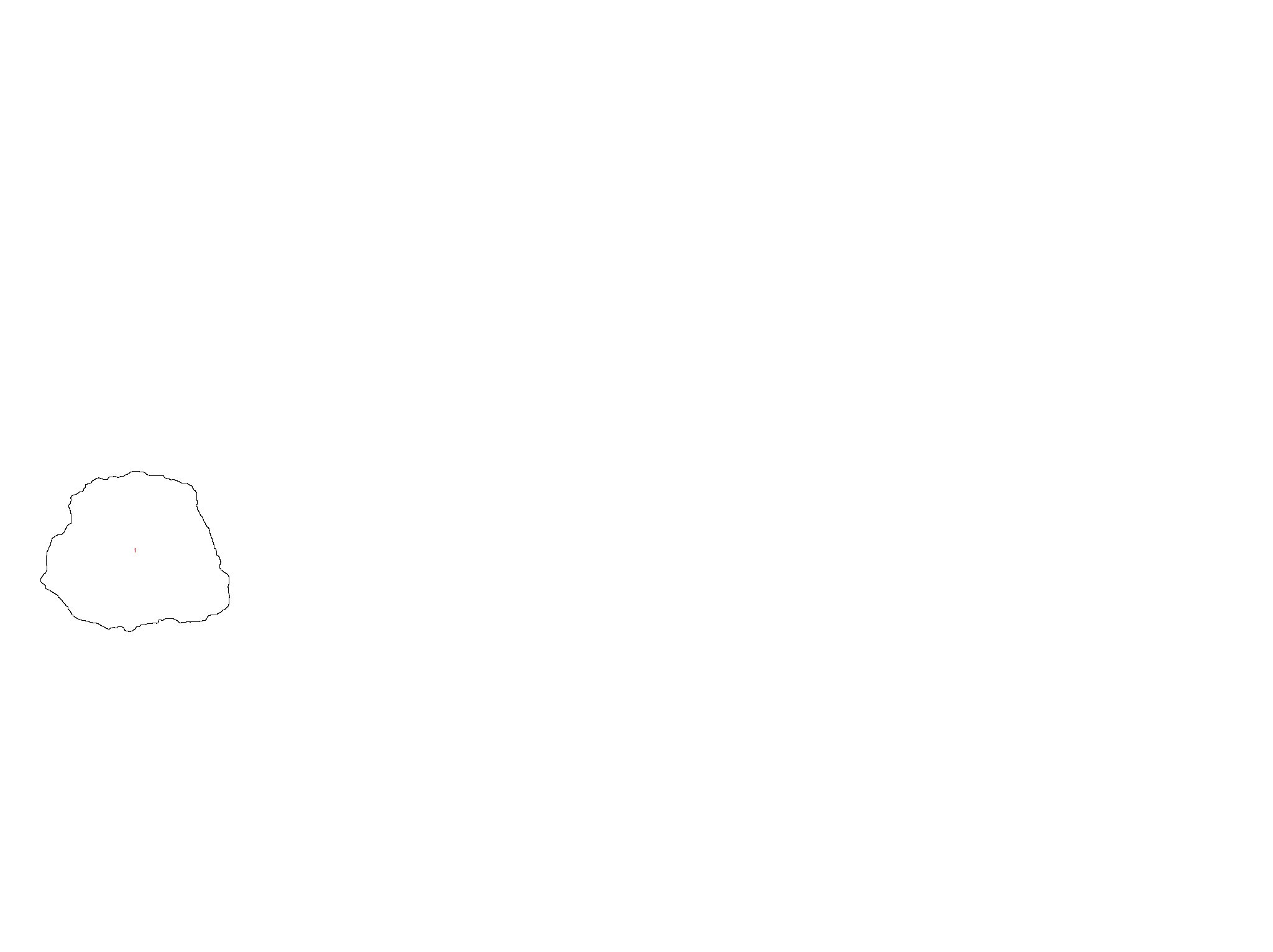

Supplement: S2 Dataset — (ZIP) [file pone.0304198.s005.zip › S2_Dataset_Raw_results_ImageJ/J2_0E_180190_5.jpg]

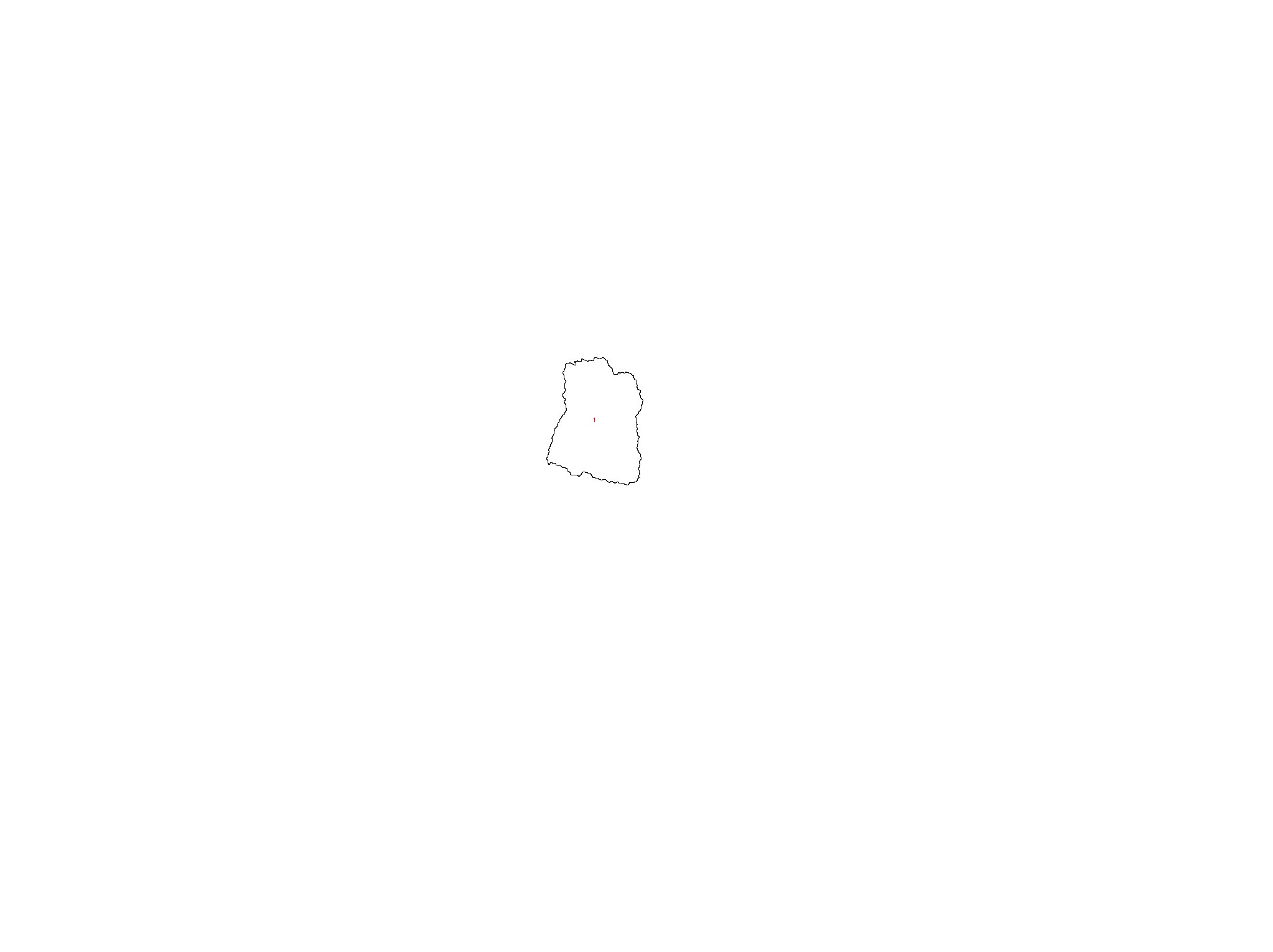

Supplement: S2 Dataset — (ZIP) [file pone.0304198.s005.zip › S2_Dataset_Raw_results_ImageJ/J2_0E_5060_1.jpg]

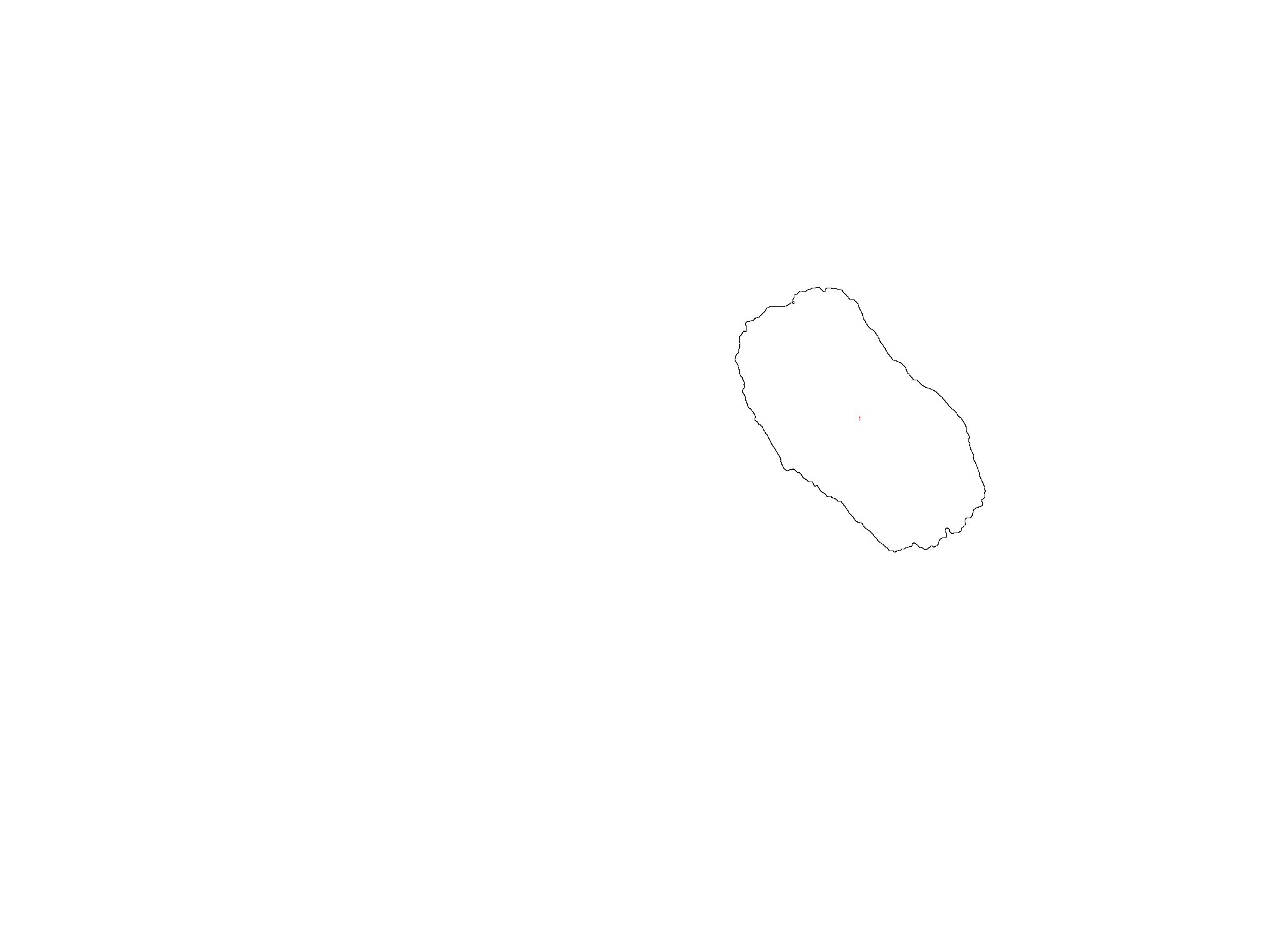

Supplement: S2 Dataset — (ZIP) [file pone.0304198.s005.zip › S2_Dataset_Raw_results_ImageJ/J2_0E_5060_2.jpg]

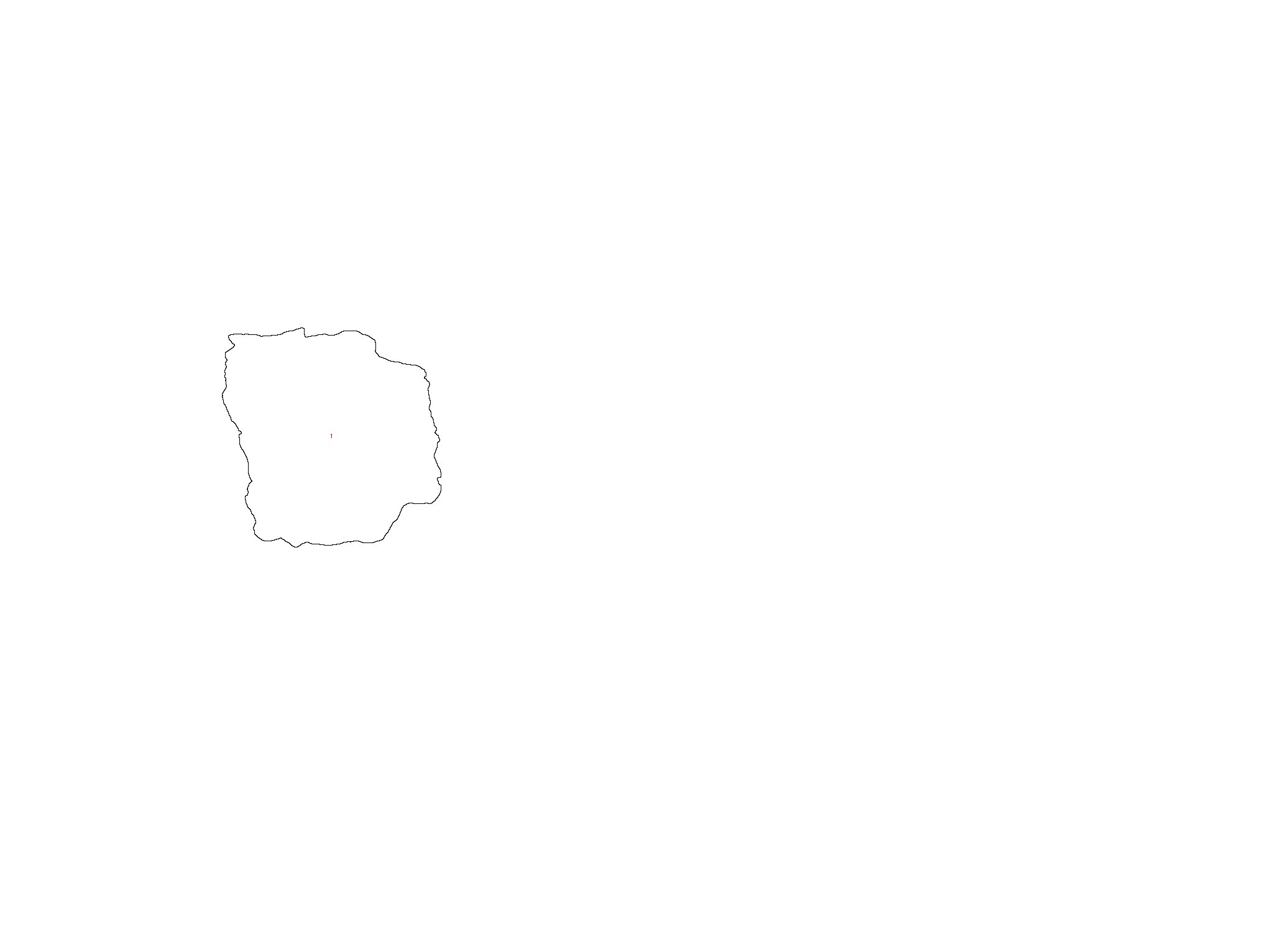

Supplement: S2 Dataset — (ZIP) [file pone.0304198.s005.zip › S2_Dataset_Raw_results_ImageJ/J2_0E_5060_3.jpg]

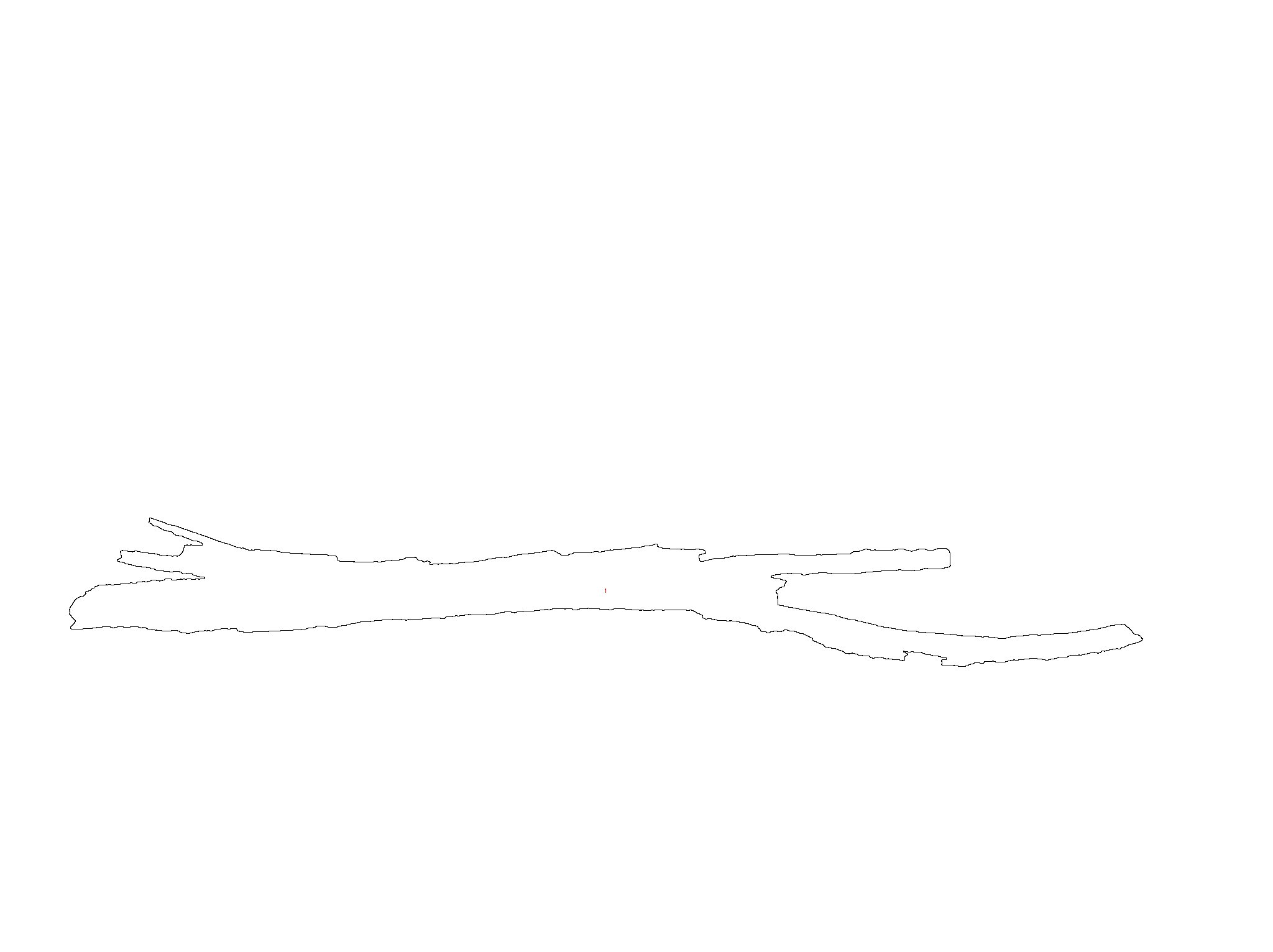

Supplement: S2 Dataset — (ZIP) [file pone.0304198.s005.zip › S2_Dataset_Raw_results_ImageJ/J2_0E_5060_4.jpg]

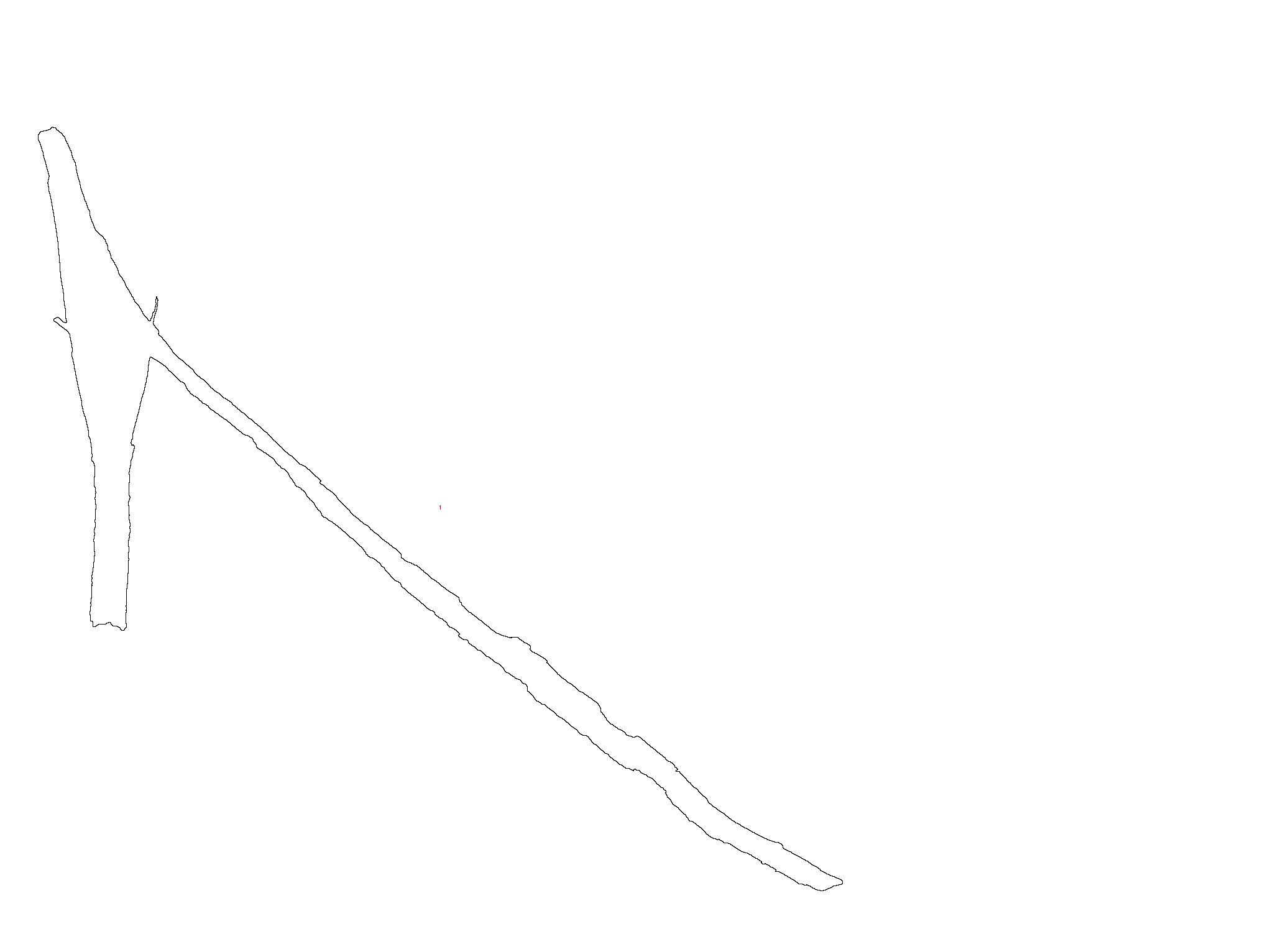

Supplement: S2 Dataset — (ZIP) [file pone.0304198.s005.zip › S2_Dataset_Raw_results_ImageJ/J2_0E_5060_5.jpg]

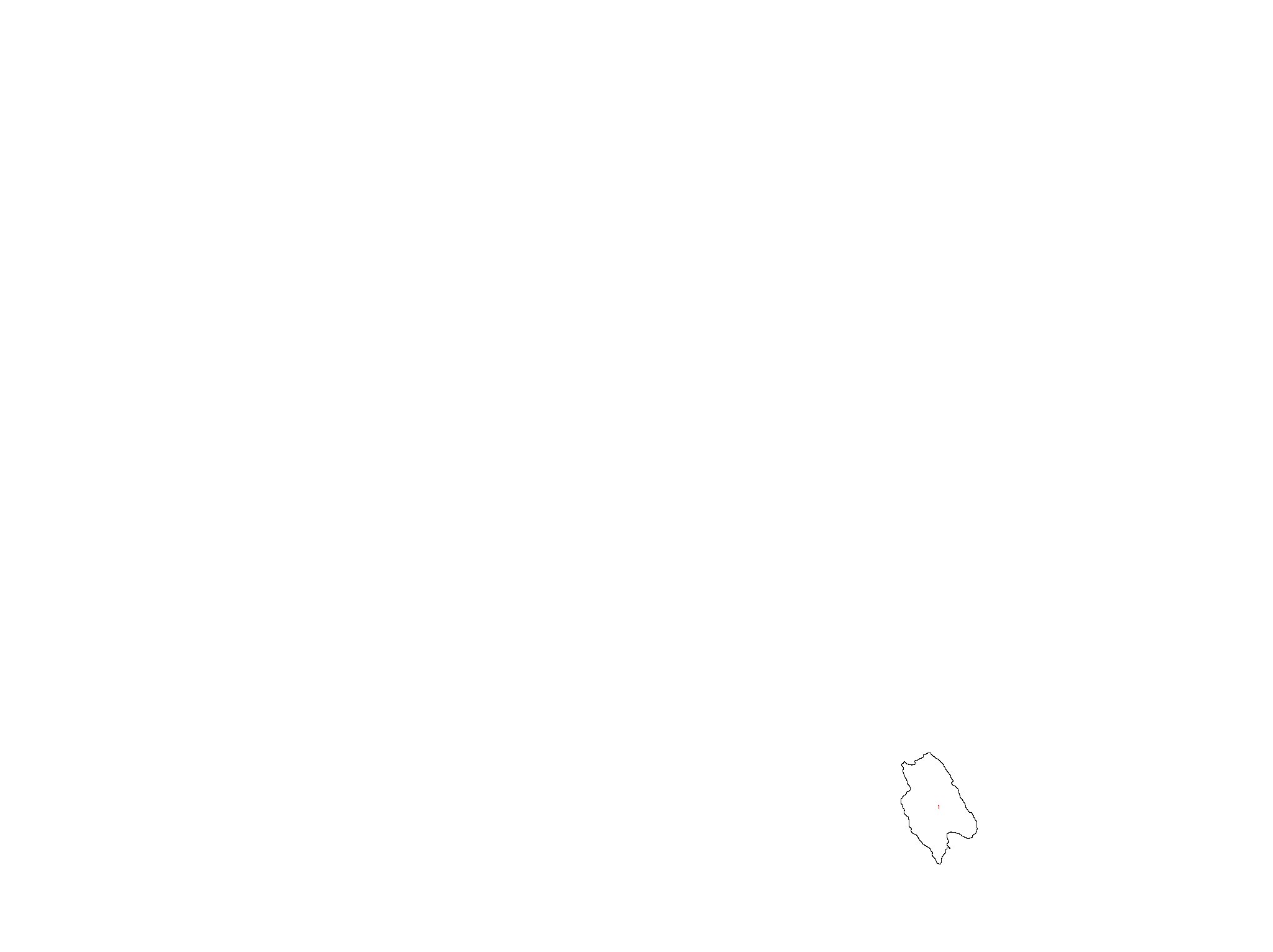

Supplement: S2 Dataset — (ZIP) [file pone.0304198.s005.zip › S2_Dataset_Raw_results_ImageJ/J2_0E_5060_6.jpg]

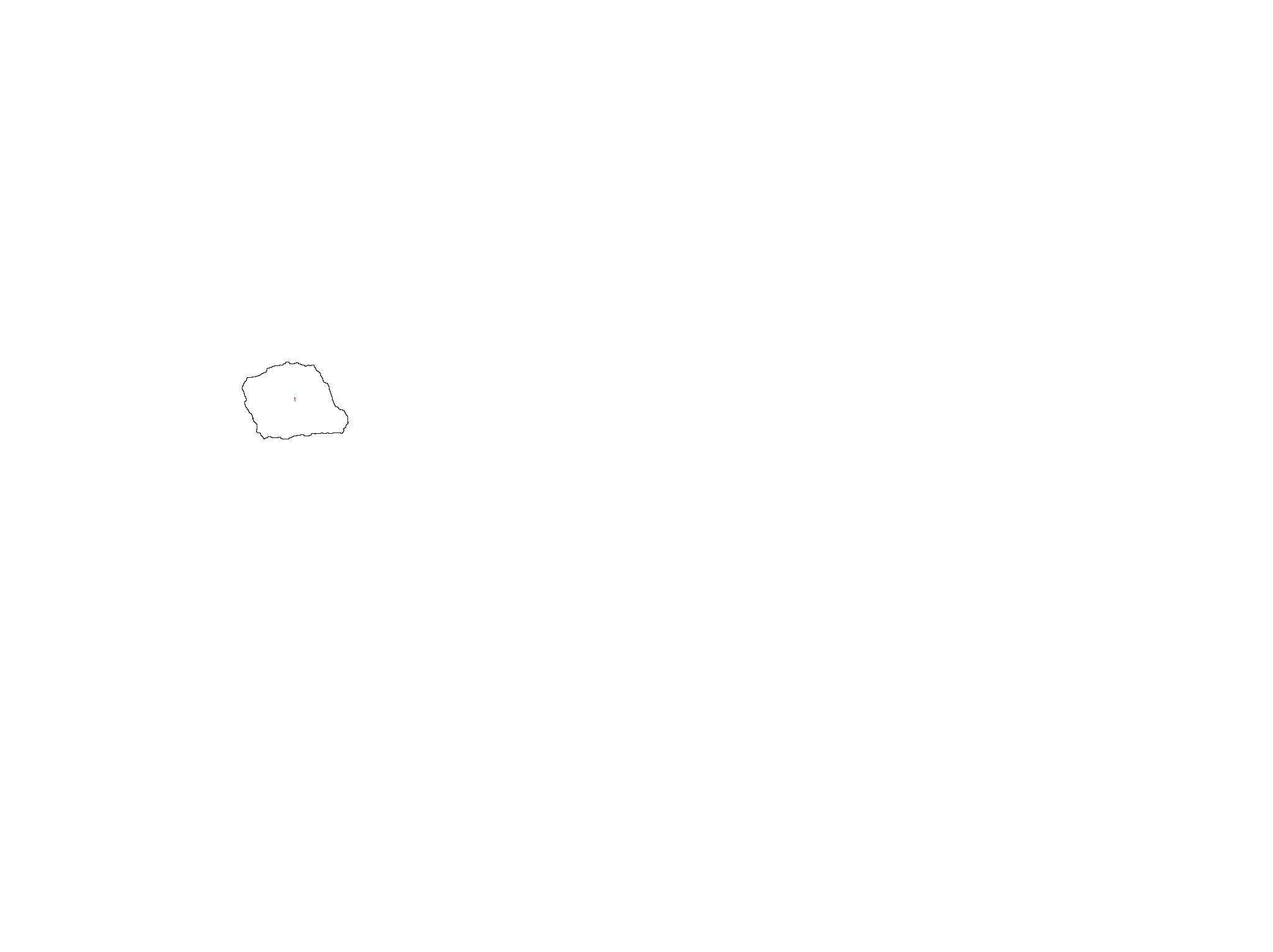

Supplement: S2 Dataset — (ZIP) [file pone.0304198.s005.zip › S2_Dataset_Raw_results_ImageJ/J2_0E_5060_7.jpg]

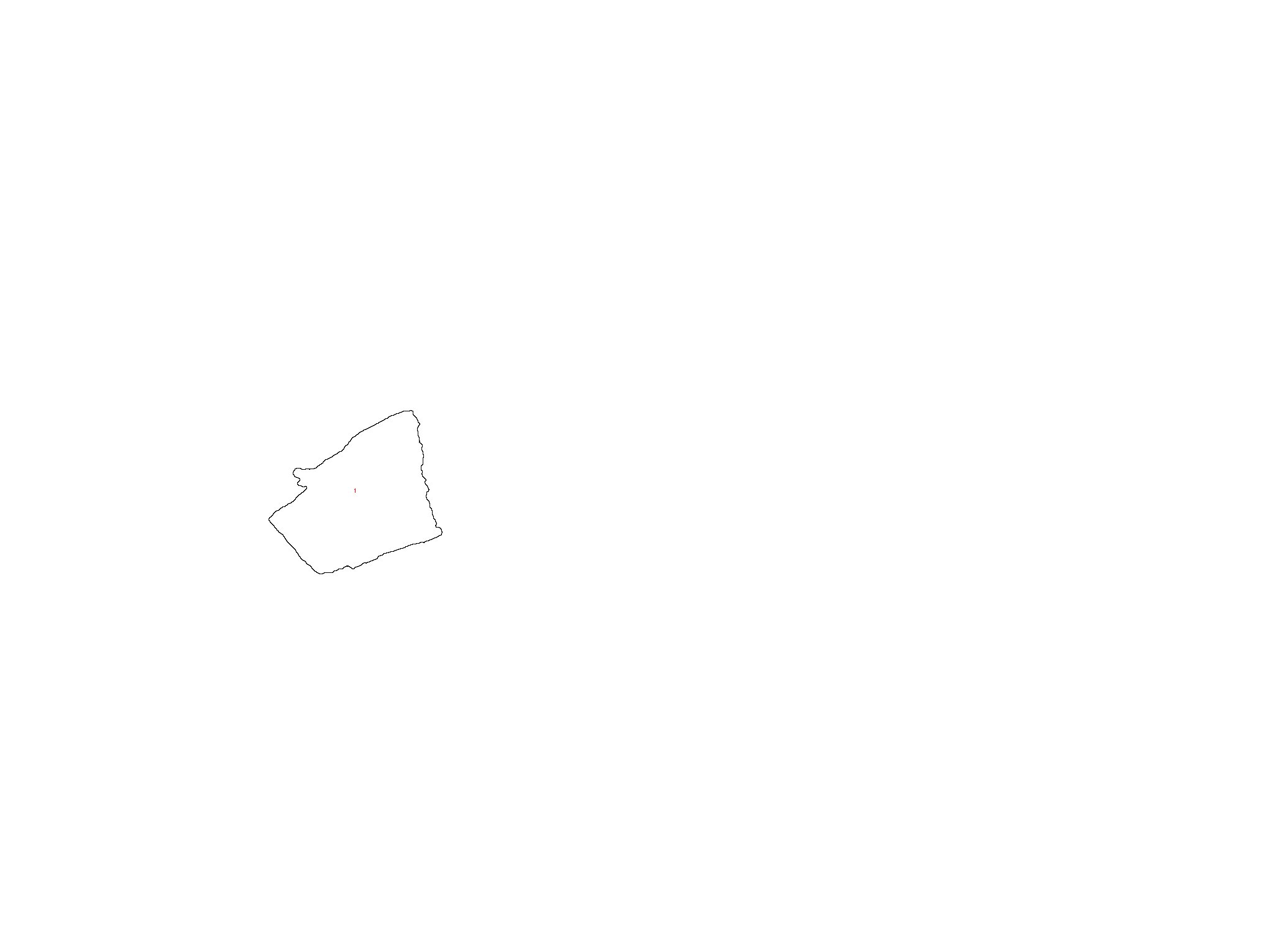

Supplement: S2 Dataset — (ZIP) [file pone.0304198.s005.zip › S2_Dataset_Raw_results_ImageJ/J2_0E_5060_8.jpg]

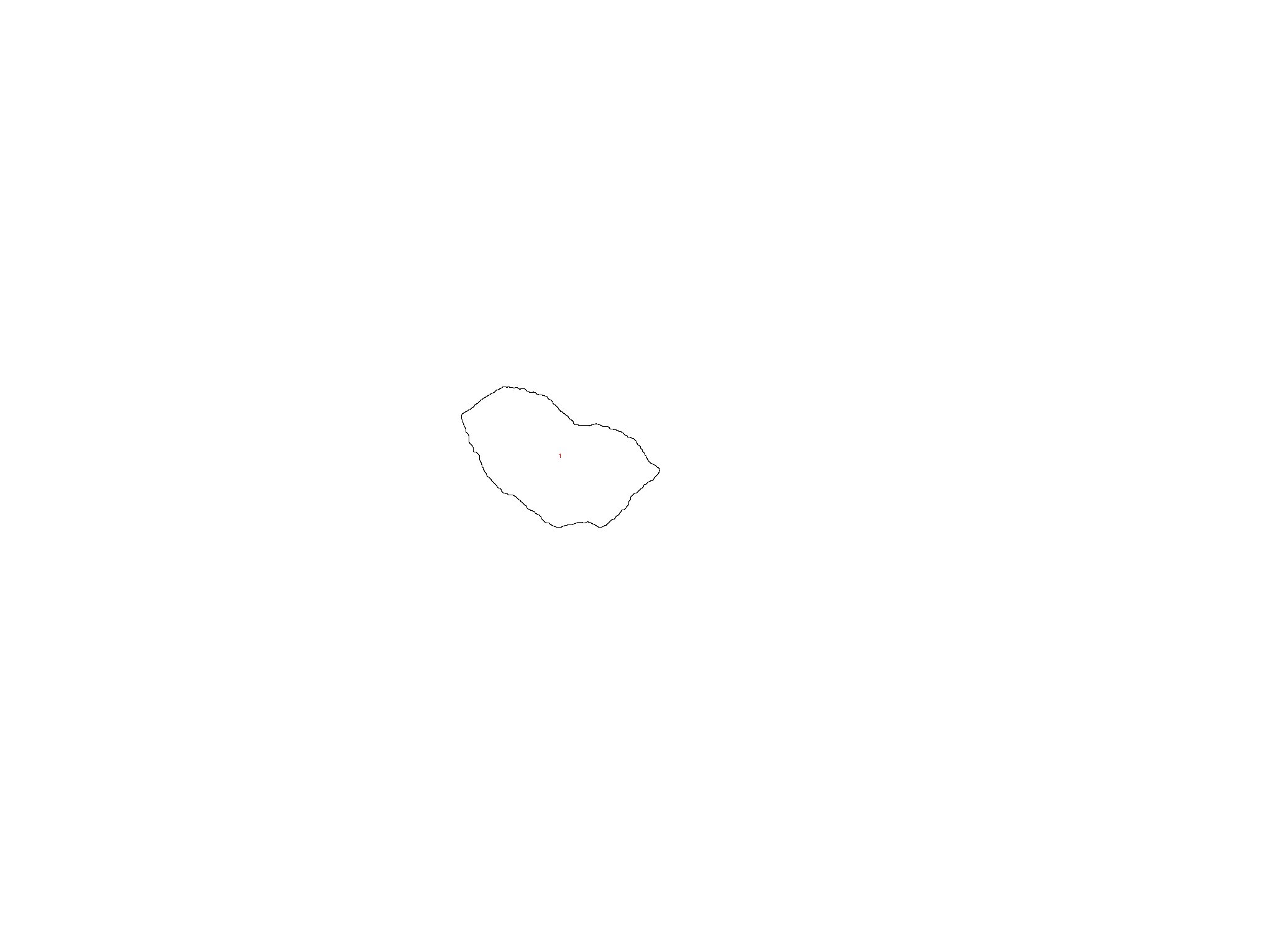

Supplement: S2 Dataset — (ZIP) [file pone.0304198.s005.zip › S2_Dataset_Raw_results_ImageJ/J2_0E_5060_9.jpg]

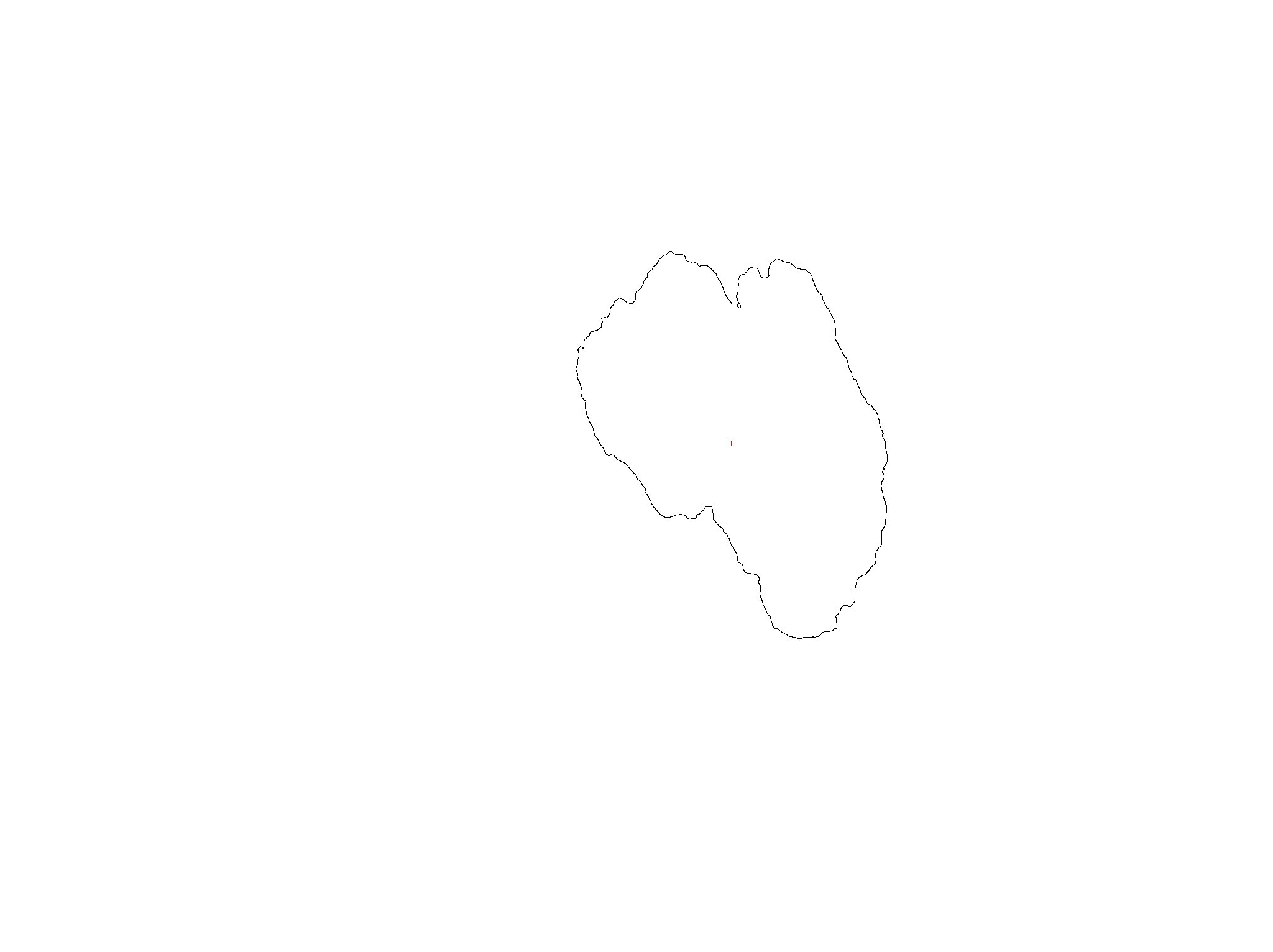

Supplement: S2 Dataset — (ZIP) [file pone.0304198.s005.zip › S2_Dataset_Raw_results_ImageJ/J2_0E_6070_1.jpg]

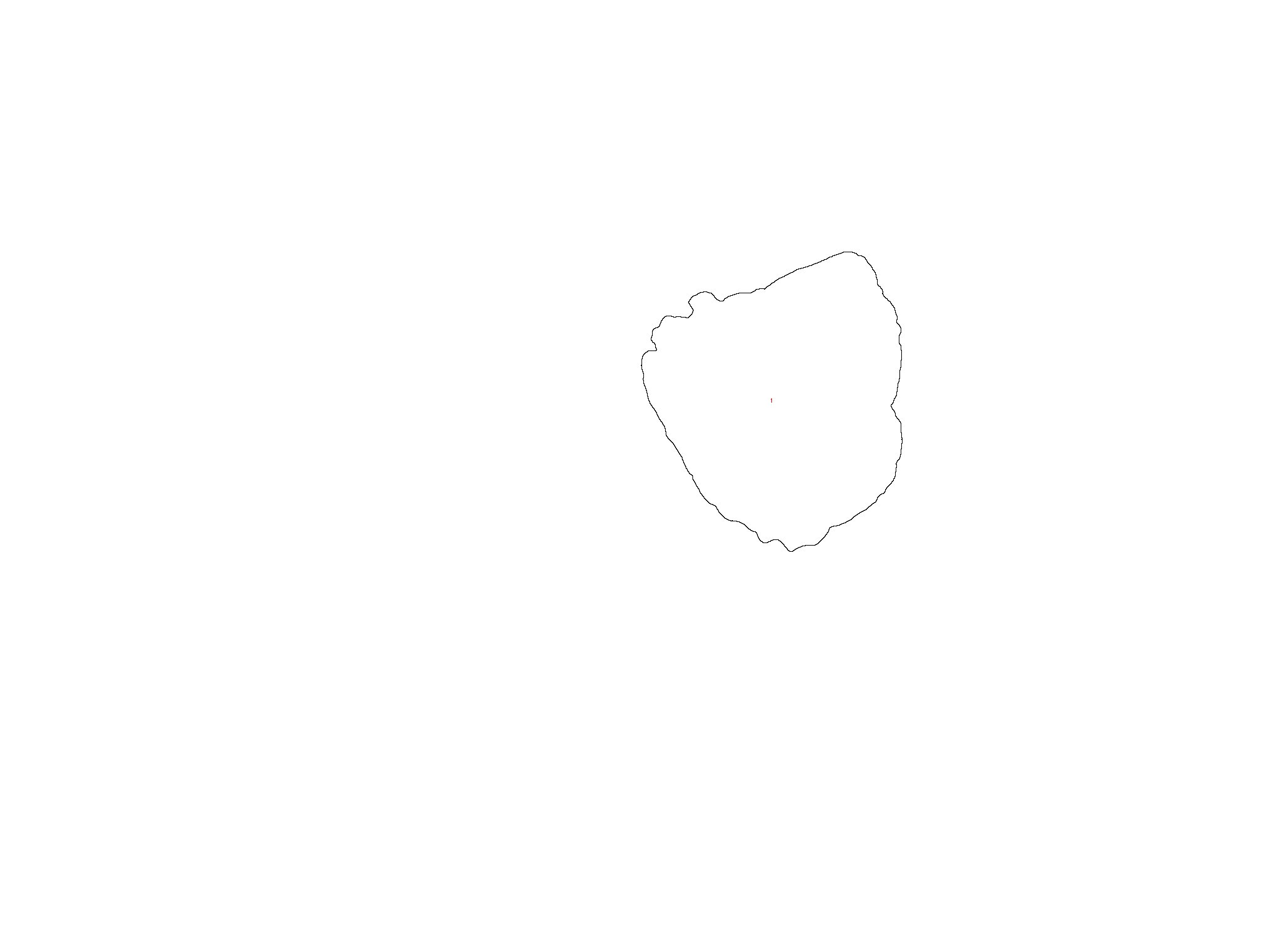

Supplement: S2 Dataset — (ZIP) [file pone.0304198.s005.zip › S2_Dataset_Raw_results_ImageJ/J2_0E_6070_2.jpg]

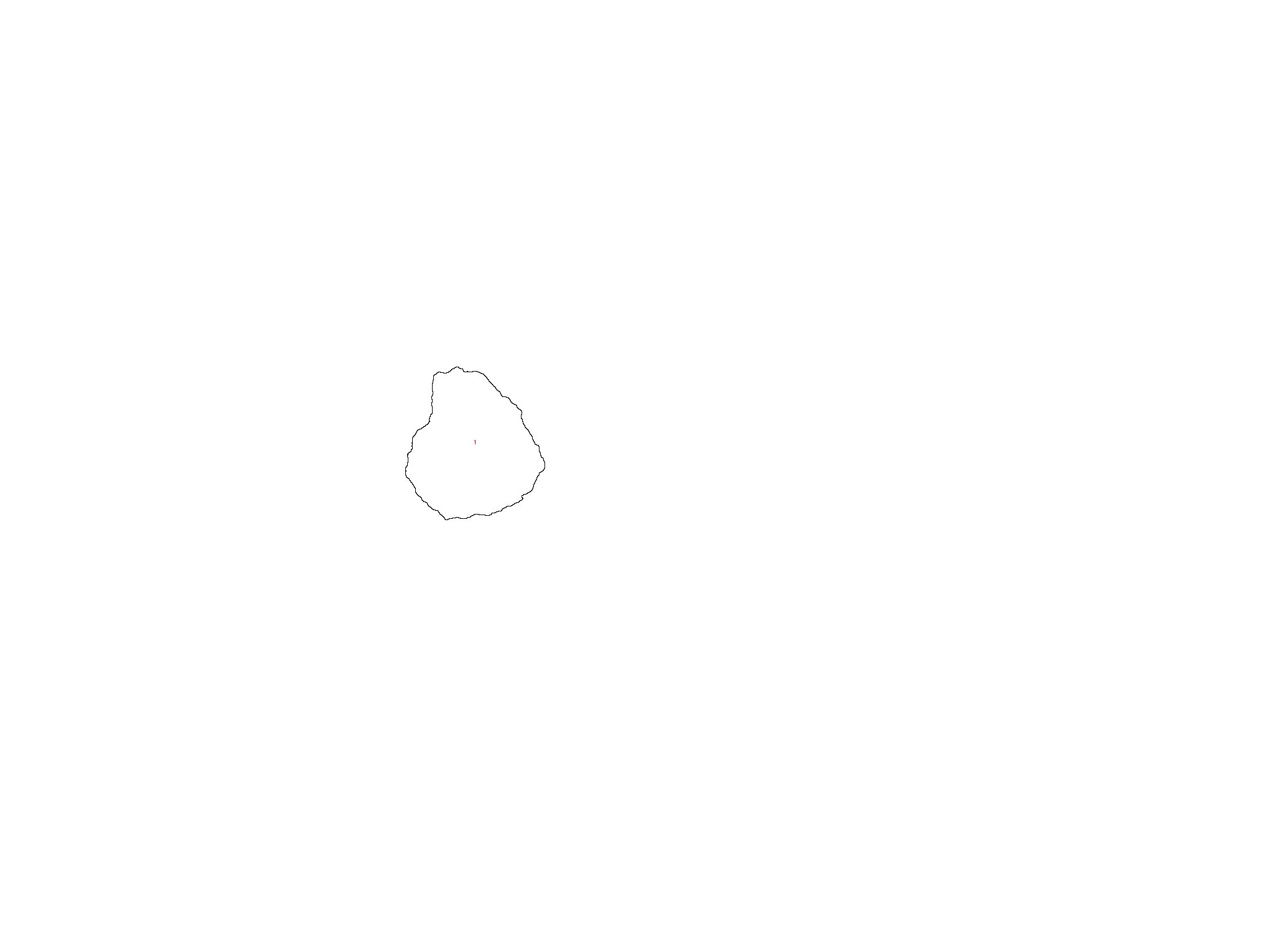

Supplement: S2 Dataset — (ZIP) [file pone.0304198.s005.zip › S2_Dataset_Raw_results_ImageJ/J2_0E_6070_3.jpg]

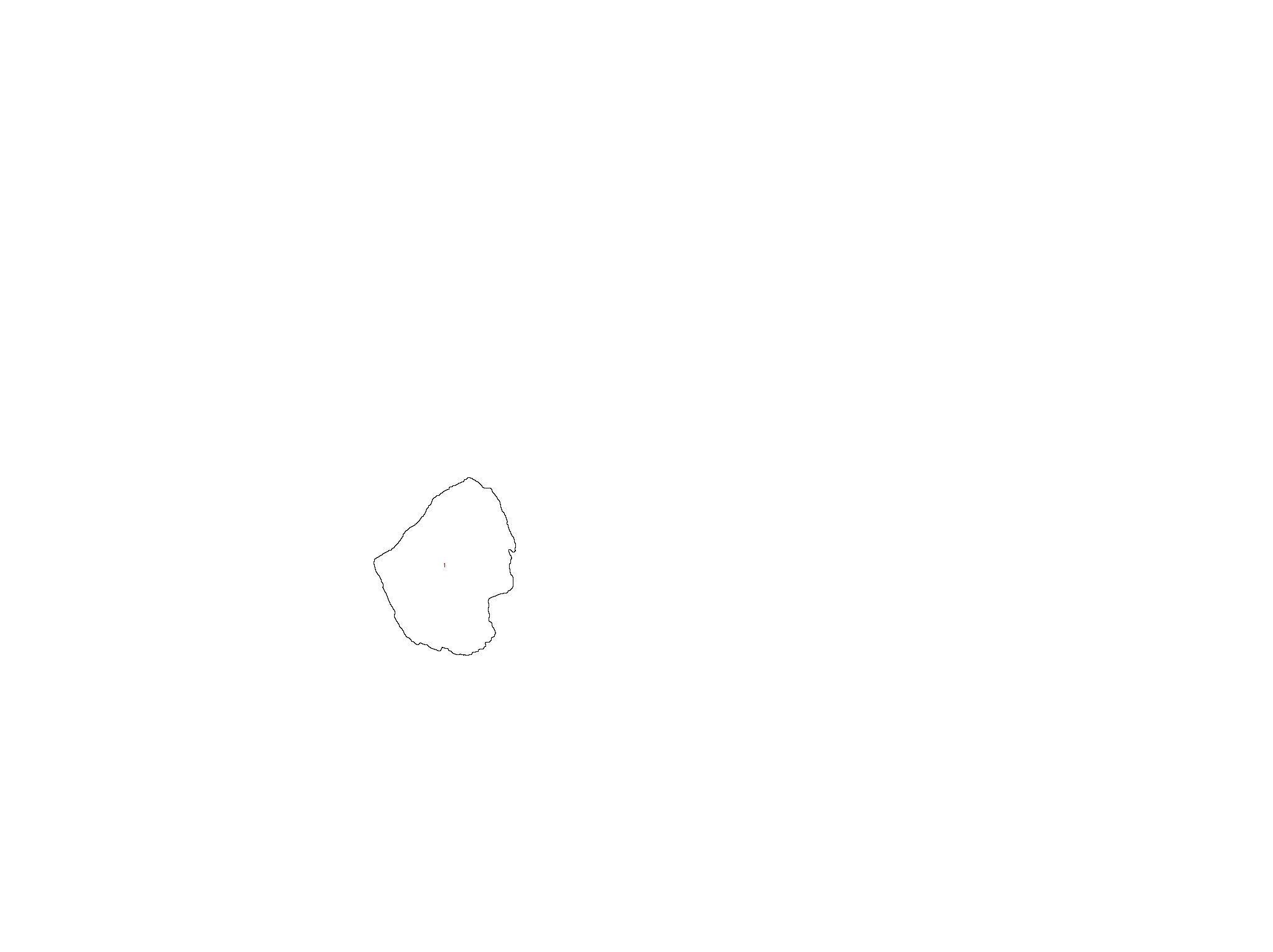

Supplement: S2 Dataset — (ZIP) [file pone.0304198.s005.zip › S2_Dataset_Raw_results_ImageJ/J2_0E_6070_4.jpg]

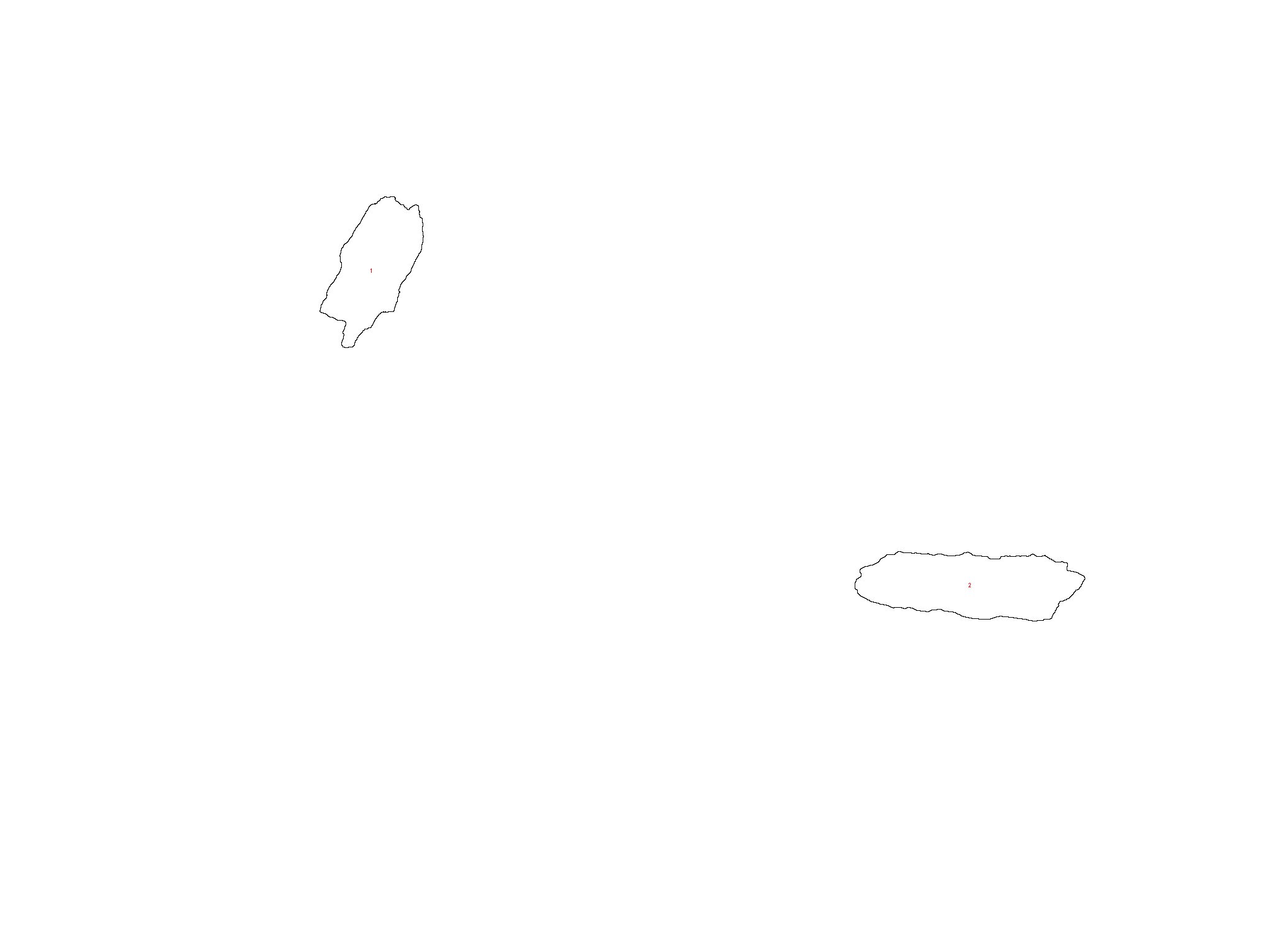

Supplement: S2 Dataset — (ZIP) [file pone.0304198.s005.zip › S2_Dataset_Raw_results_ImageJ/J2_0E_6070_5.jpg]

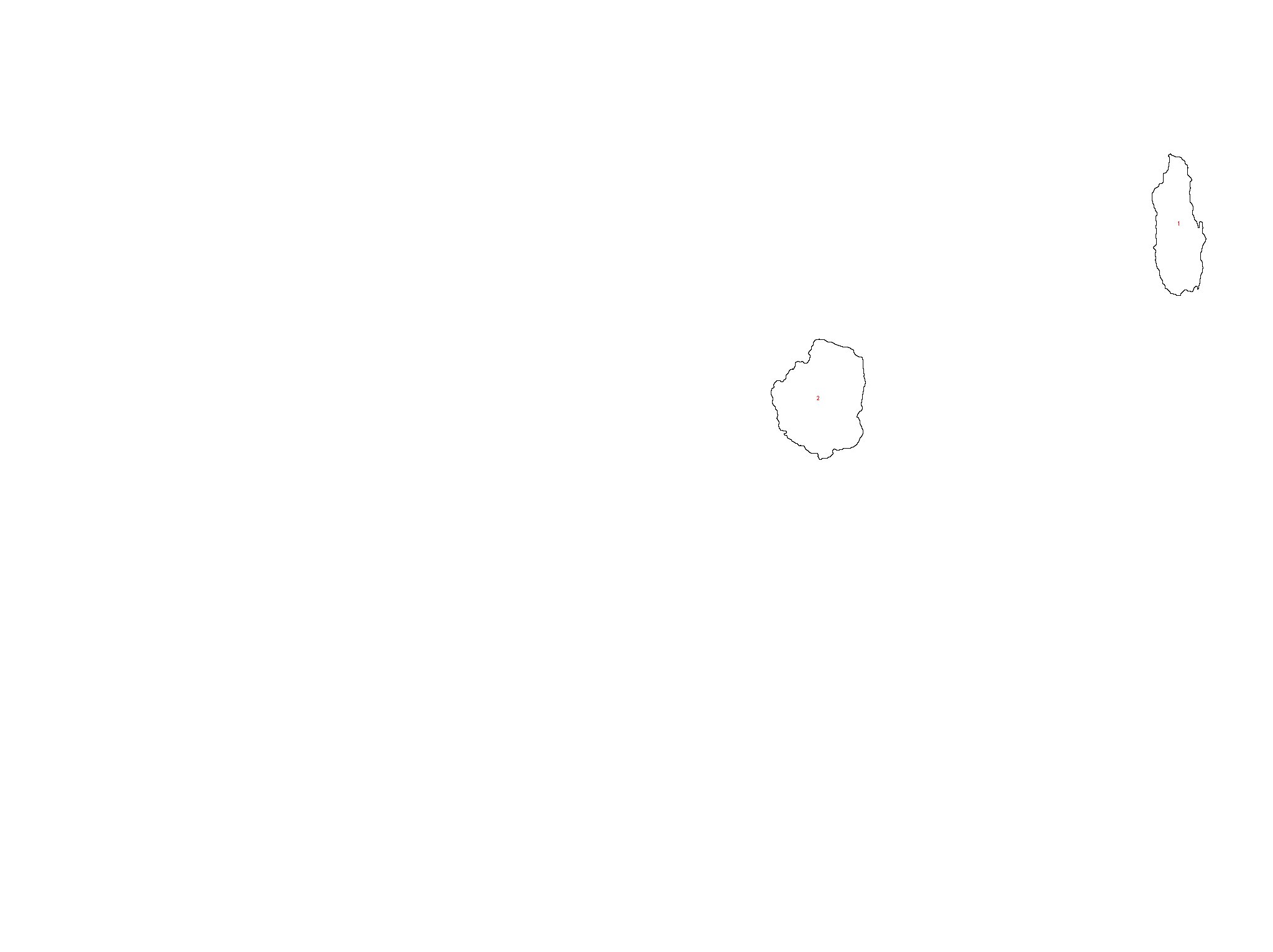

Supplement: S2 Dataset — (ZIP) [file pone.0304198.s005.zip › S2_Dataset_Raw_results_ImageJ/J2_0E_6070_6.jpg]

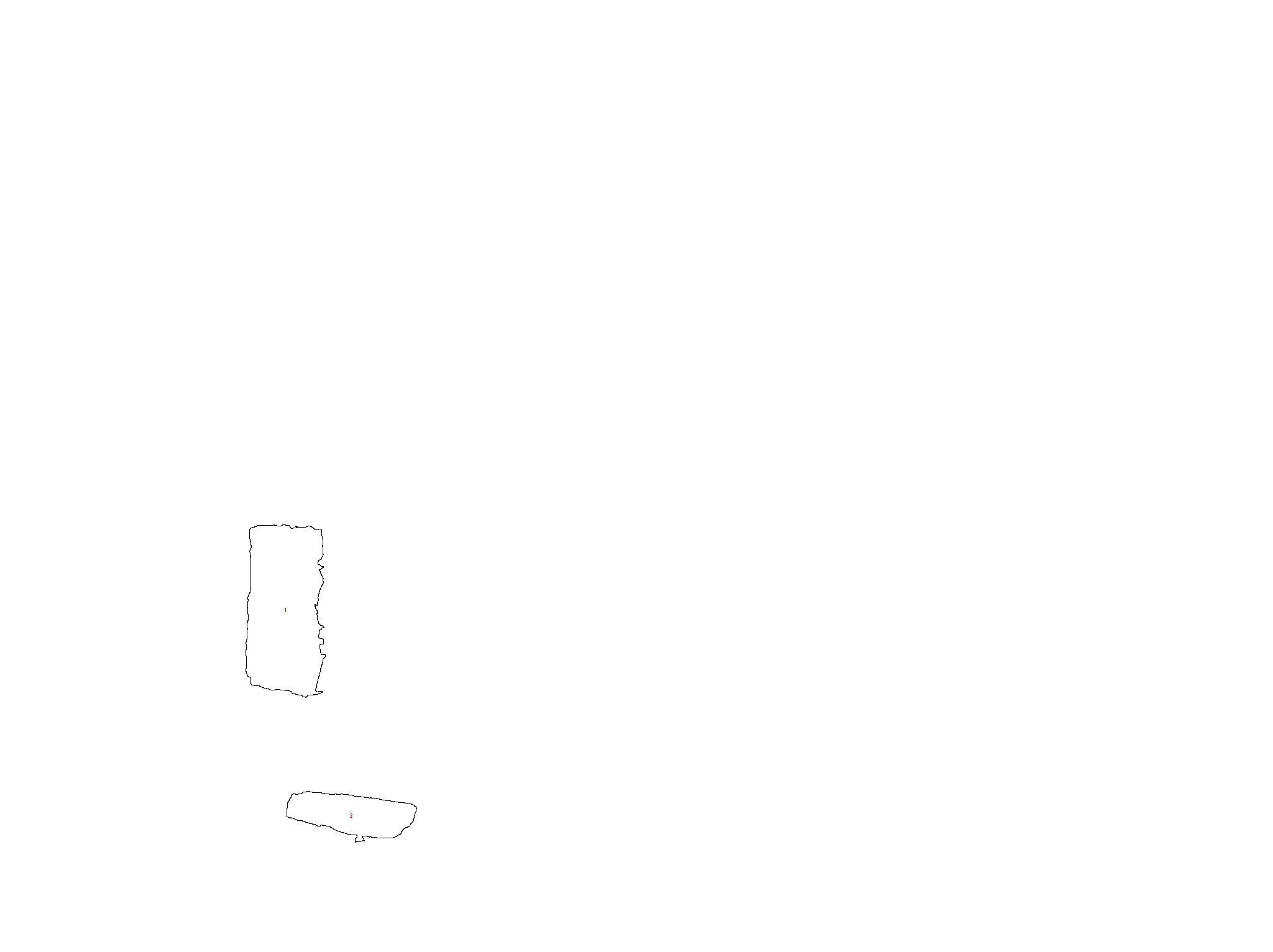

Supplement: S2 Dataset — (ZIP) [file pone.0304198.s005.zip › S2_Dataset_Raw_results_ImageJ/J2_100F_1020_1.jpg]

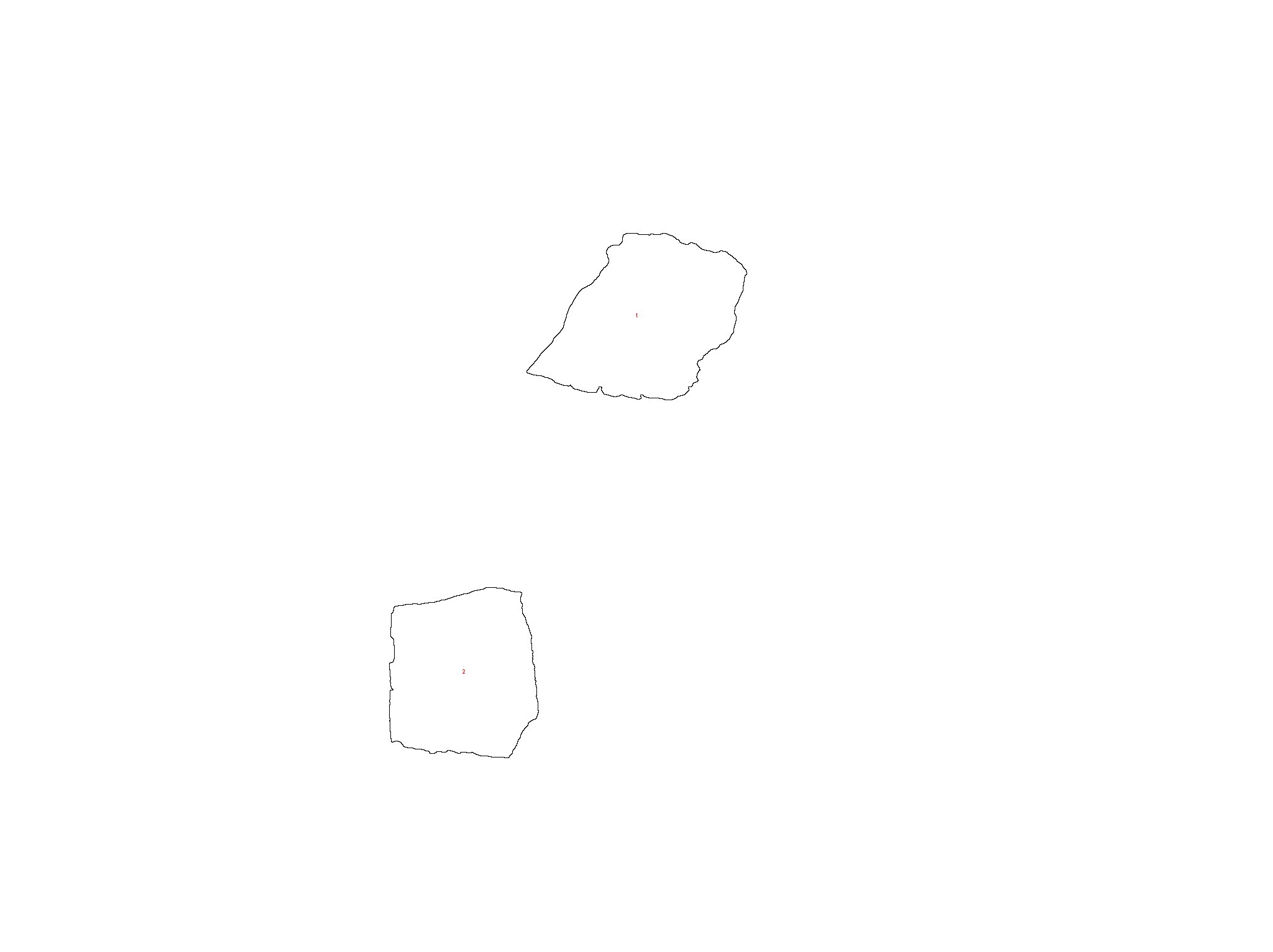

Supplement: S2 Dataset — (ZIP) [file pone.0304198.s005.zip › S2_Dataset_Raw_results_ImageJ/J2_100F_1020_2.jpg]

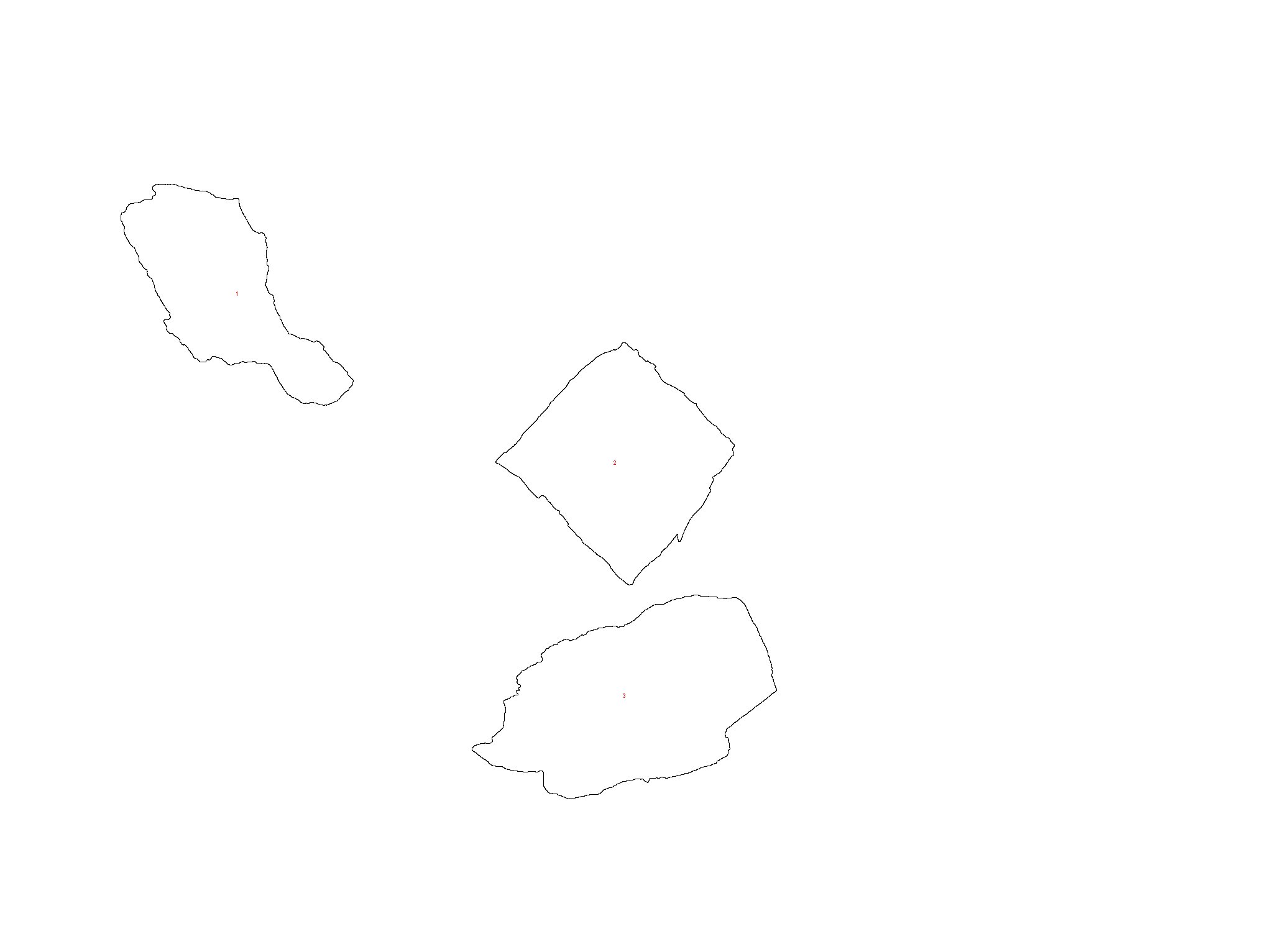

Supplement: S2 Dataset — (ZIP) [file pone.0304198.s005.zip › S2_Dataset_Raw_results_ImageJ/J2_100F_1020_3.jpg]

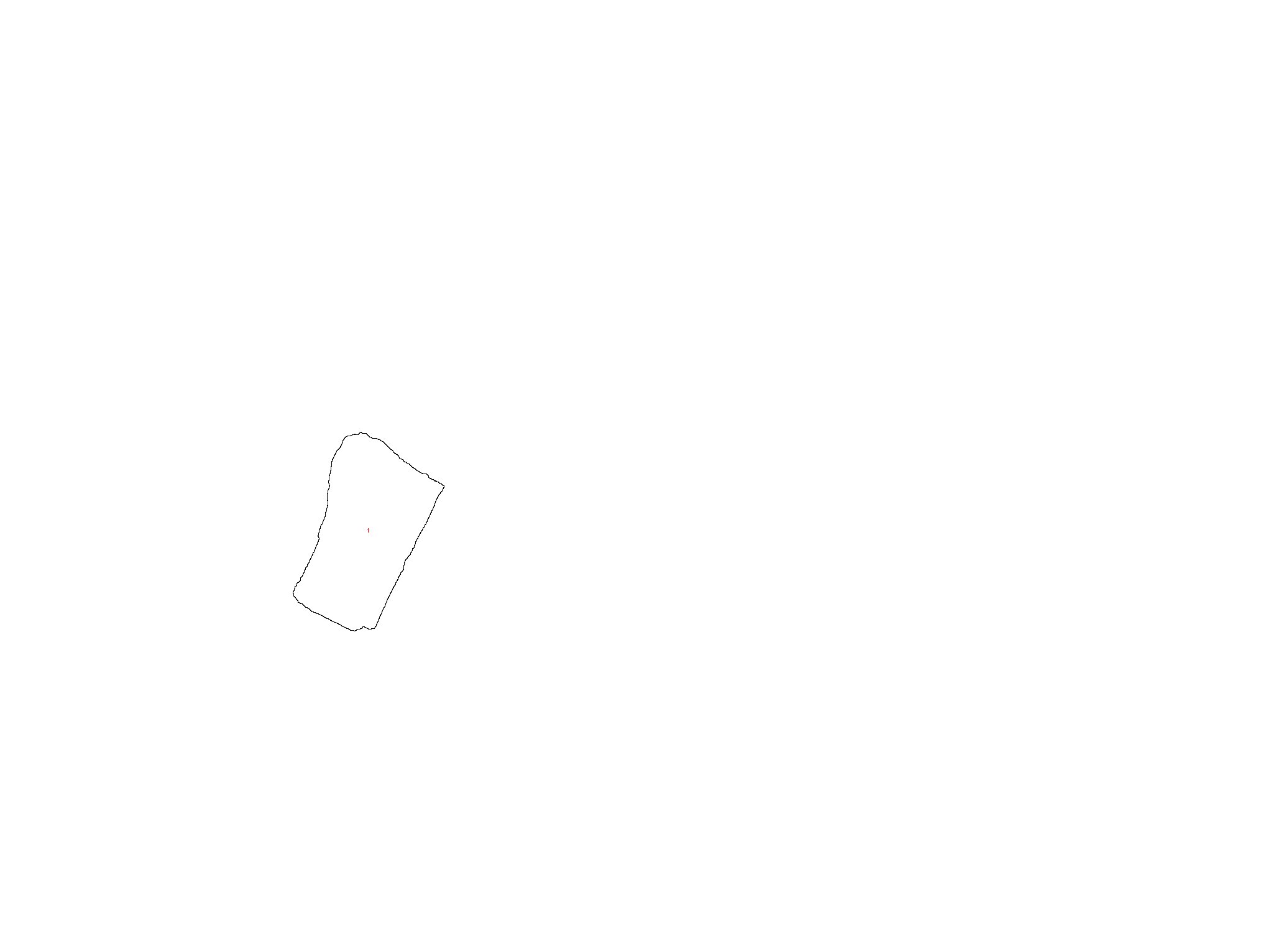

Supplement: S2 Dataset — (ZIP) [file pone.0304198.s005.zip › S2_Dataset_Raw_results_ImageJ/J2_100F_1020_4.jpg]

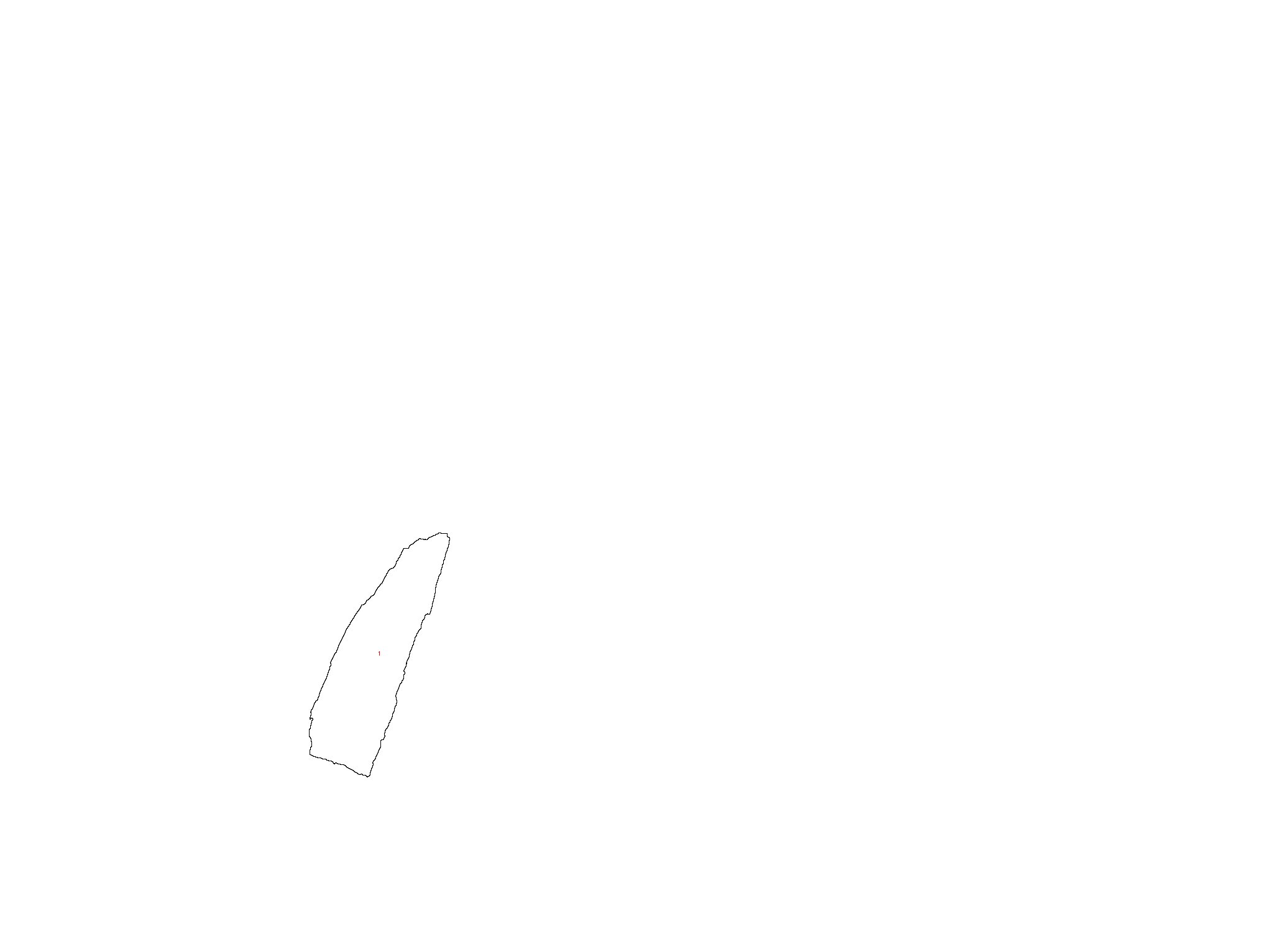

Supplement: S2 Dataset — (ZIP) [file pone.0304198.s005.zip › S2_Dataset_Raw_results_ImageJ/J2_100F_1020_5.jpg]

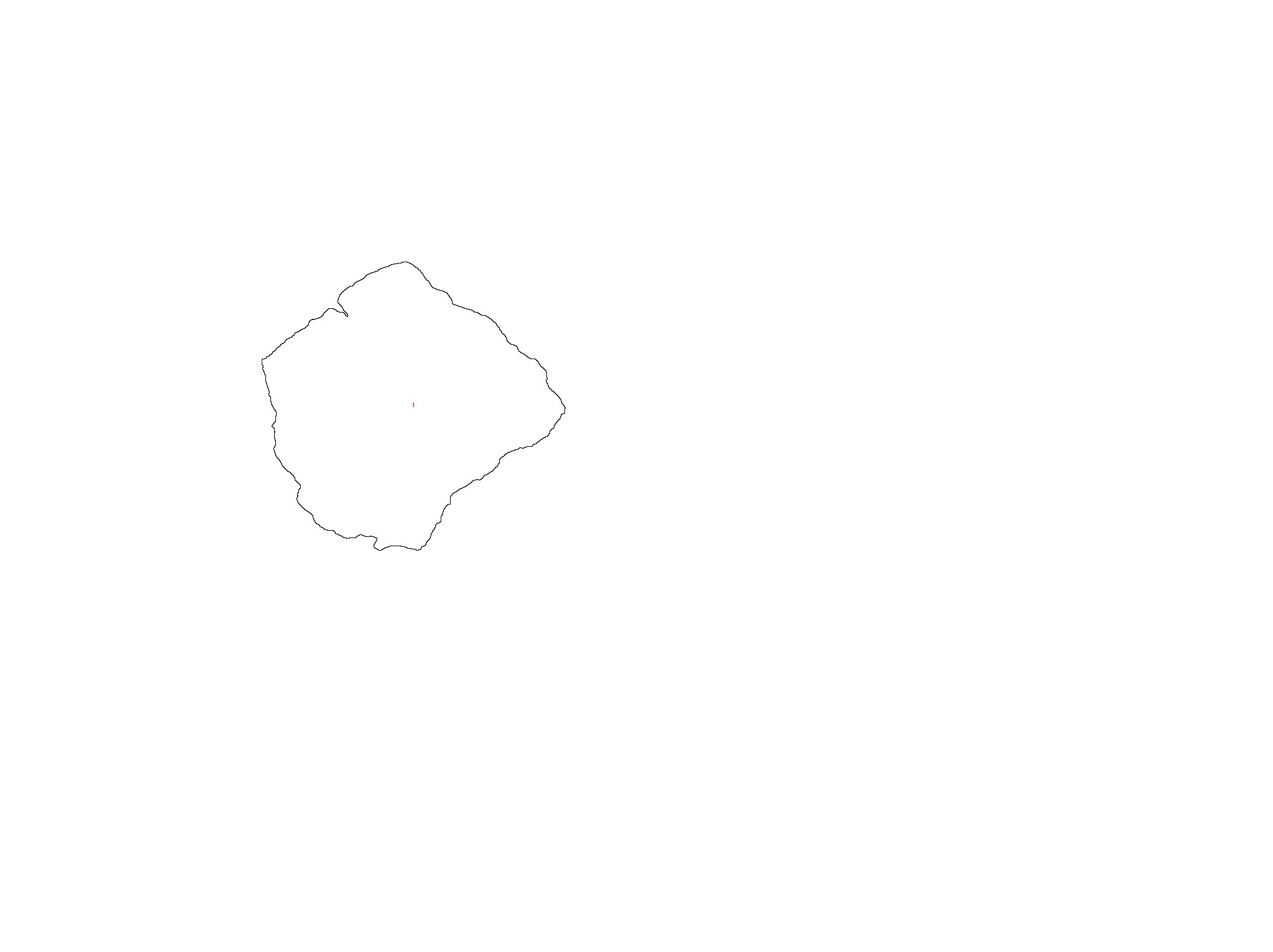

Supplement: S2 Dataset — (ZIP) [file pone.0304198.s005.zip › S2_Dataset_Raw_results_ImageJ/J2_100F_1020_6.jpg]

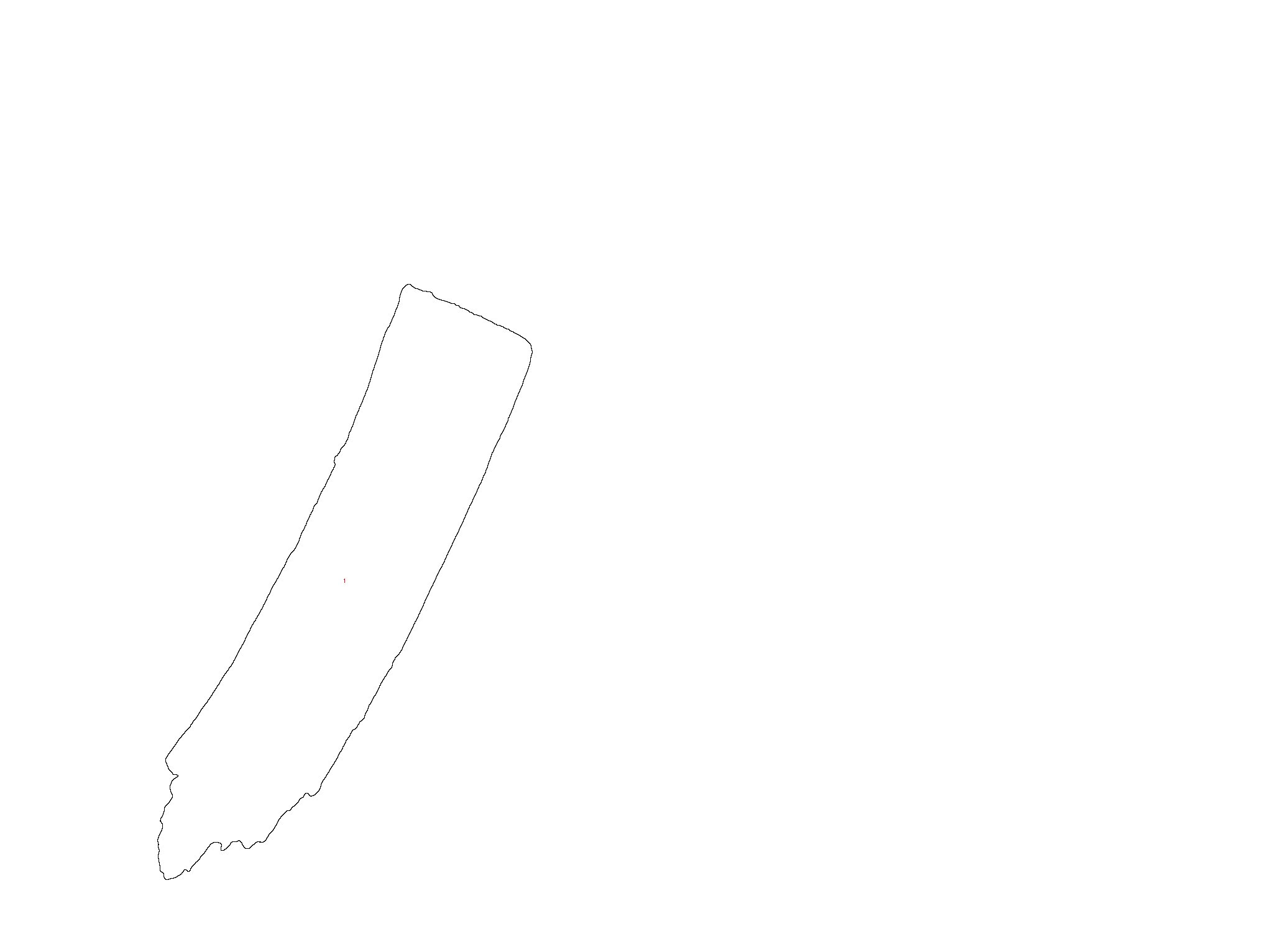

Supplement: S2 Dataset — (ZIP) [file pone.0304198.s005.zip › S2_Dataset_Raw_results_ImageJ/J2_100F_1020_7.jpg]

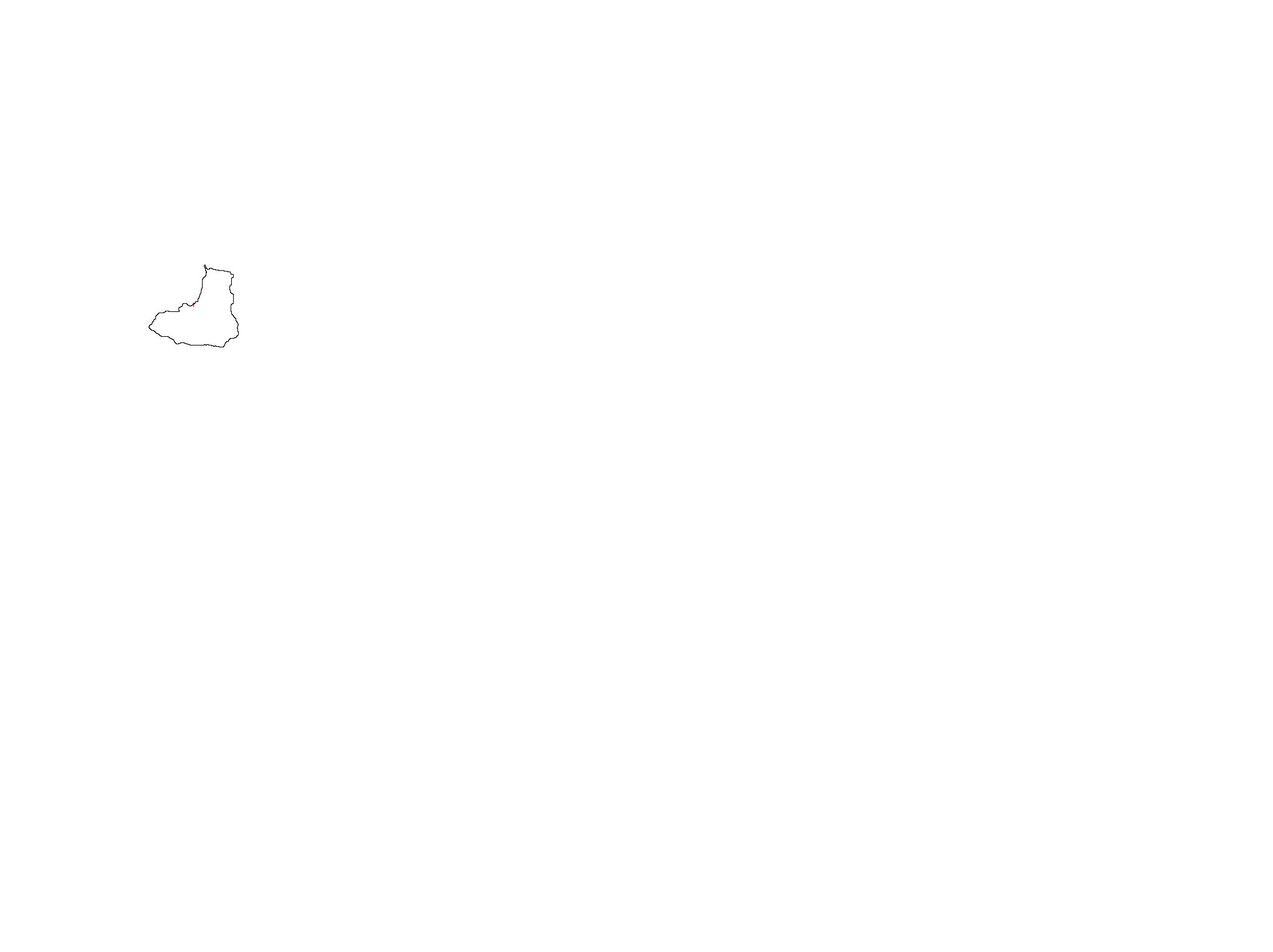

Supplement: S2 Dataset — (ZIP) [file pone.0304198.s005.zip › S2_Dataset_Raw_results_ImageJ/J2_100F_1020_8.jpg]

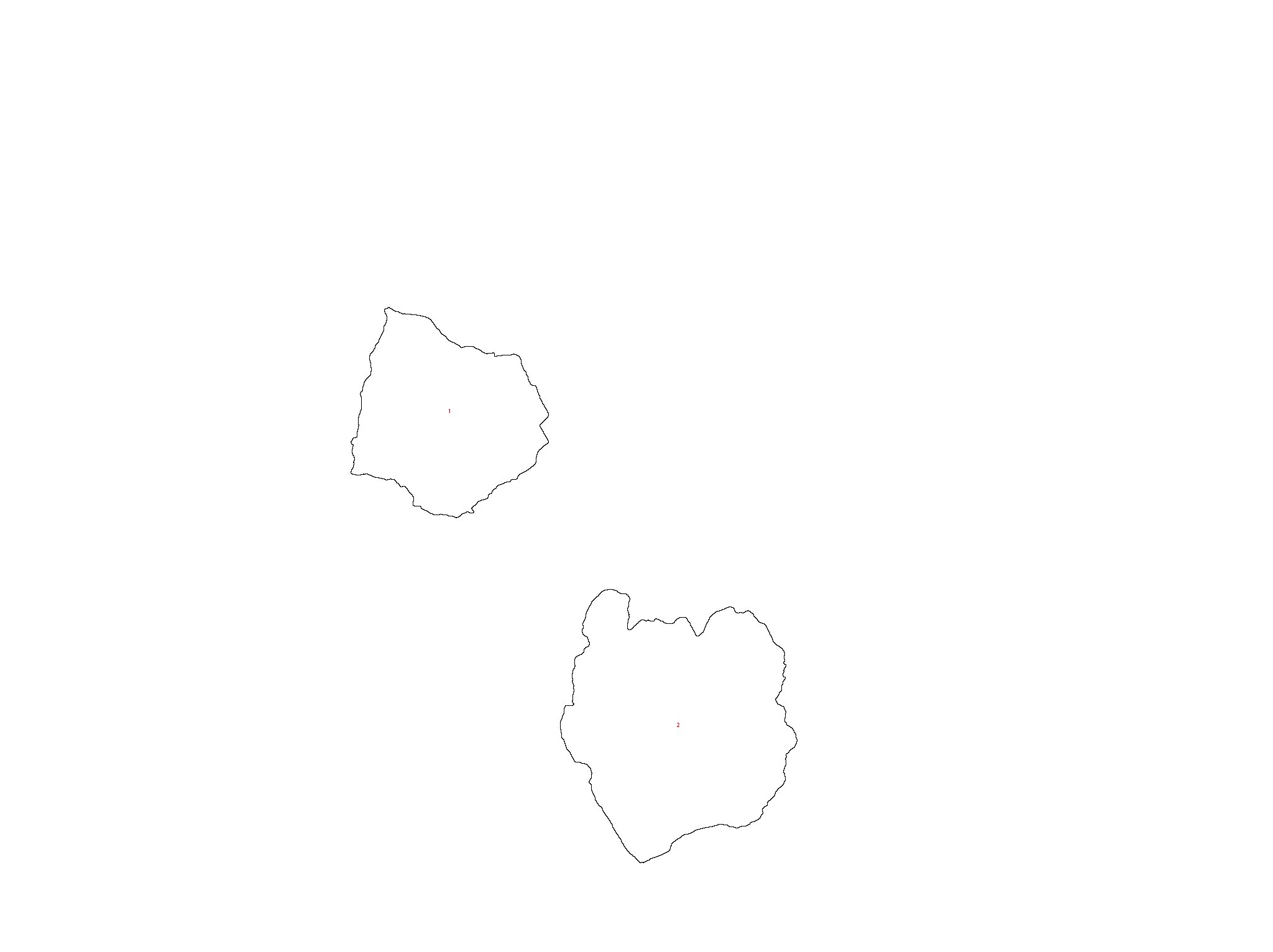

Supplement: S2 Dataset — (ZIP) [file pone.0304198.s005.zip › S2_Dataset_Raw_results_ImageJ/J2_100F_1020_9.jpg]

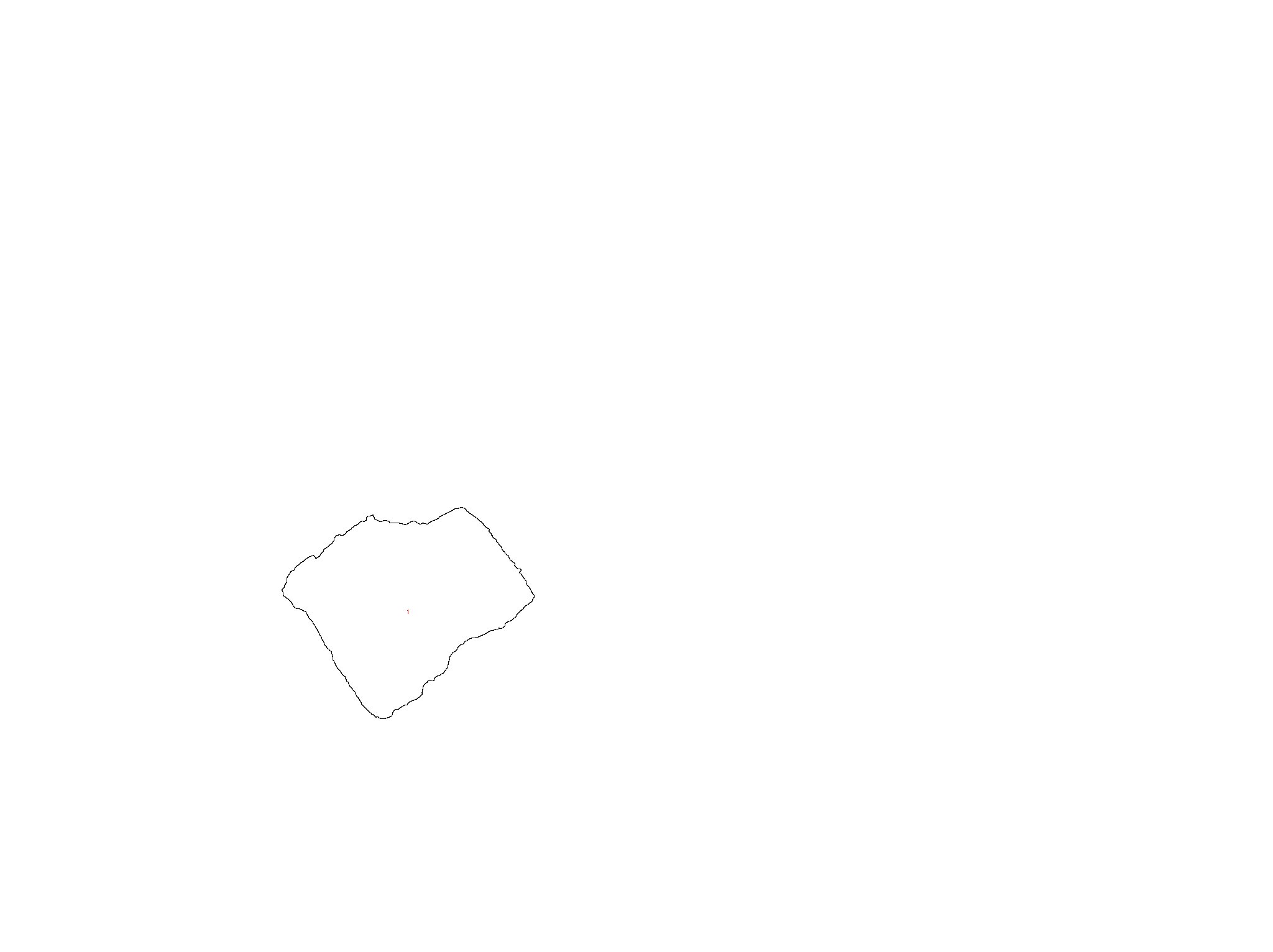

Supplement: S2 Dataset — (ZIP) [file pone.0304198.s005.zip › S2_Dataset_Raw_results_ImageJ/J2_100F_130140_1.jpg]

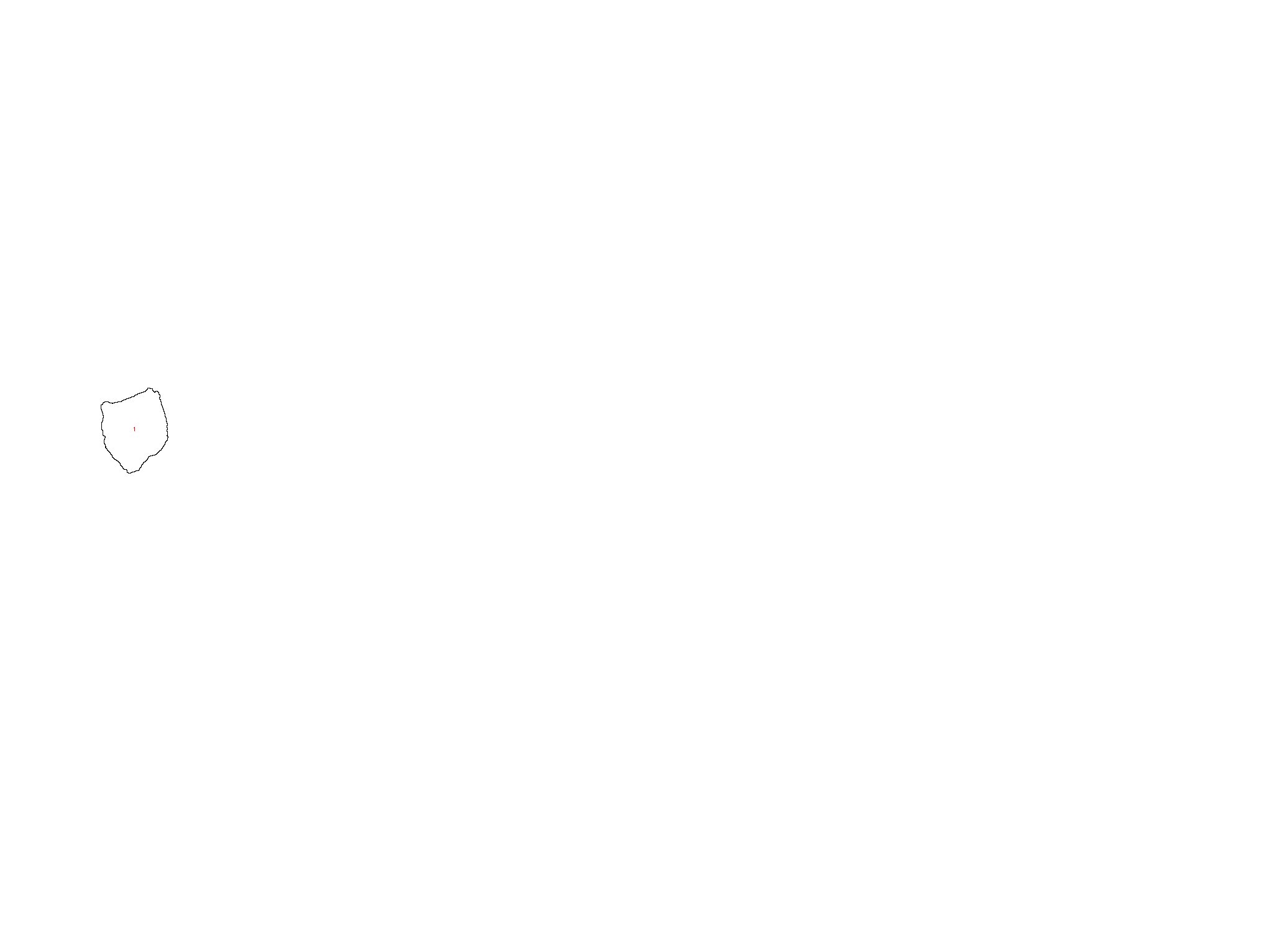

Supplement: S2 Dataset — (ZIP) [file pone.0304198.s005.zip › S2_Dataset_Raw_results_ImageJ/J2_100F_130140_2.jpg]

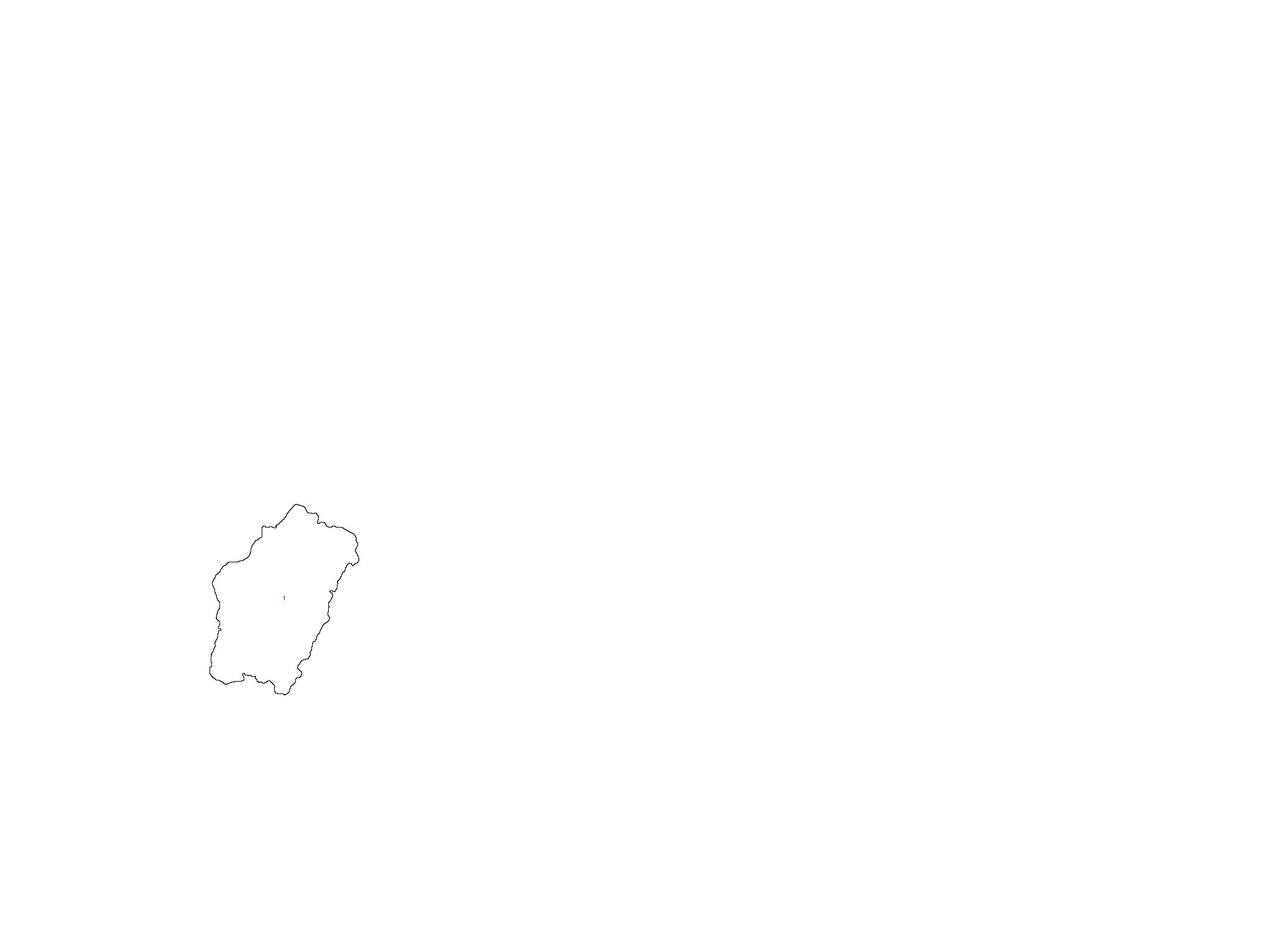

Supplement: S2 Dataset — (ZIP) [file pone.0304198.s005.zip › S2_Dataset_Raw_results_ImageJ/J2_100F_140150_1.jpg]

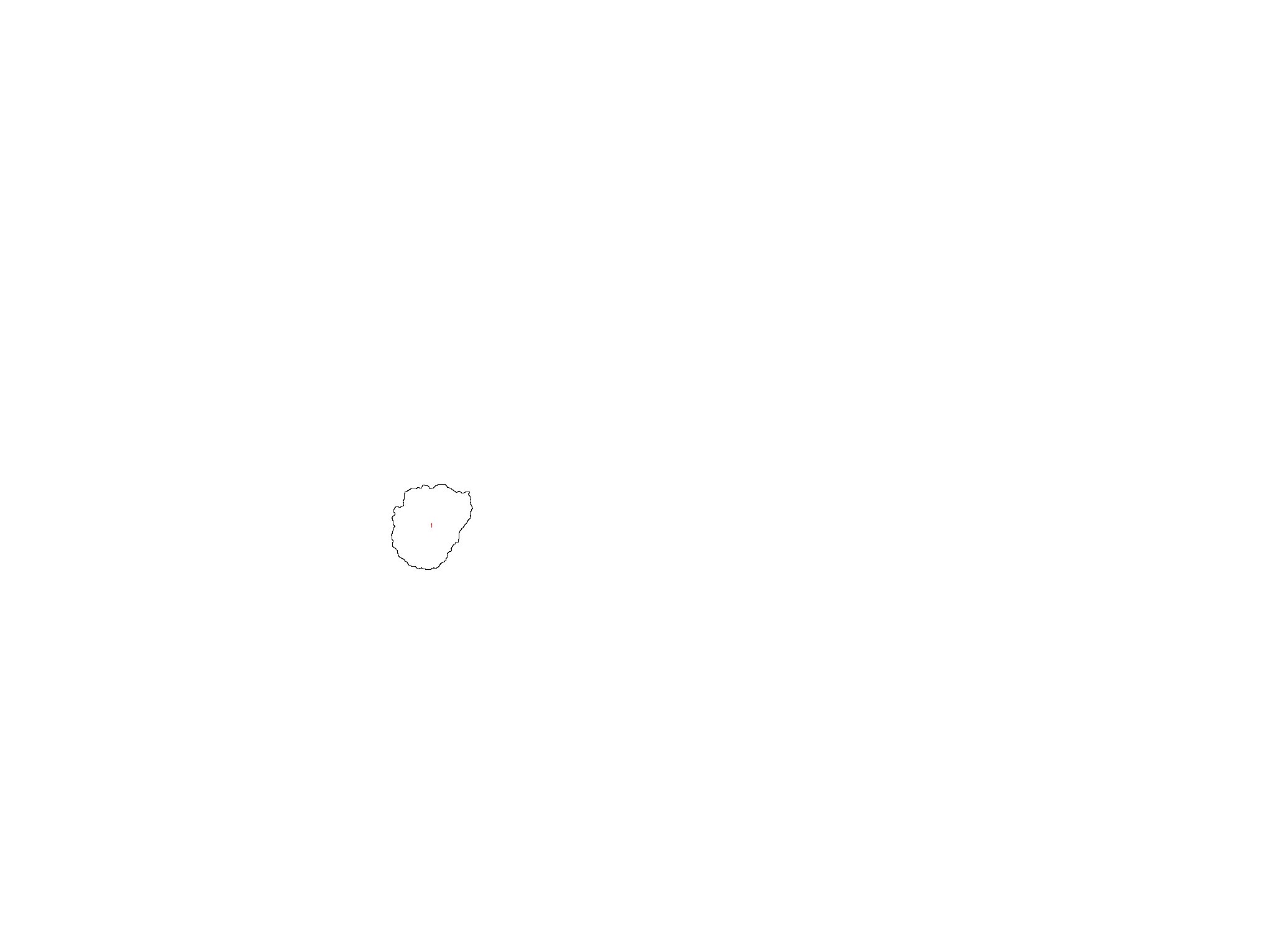

Supplement: S2 Dataset — (ZIP) [file pone.0304198.s005.zip › S2_Dataset_Raw_results_ImageJ/J2_100F_140150_3.jpg]

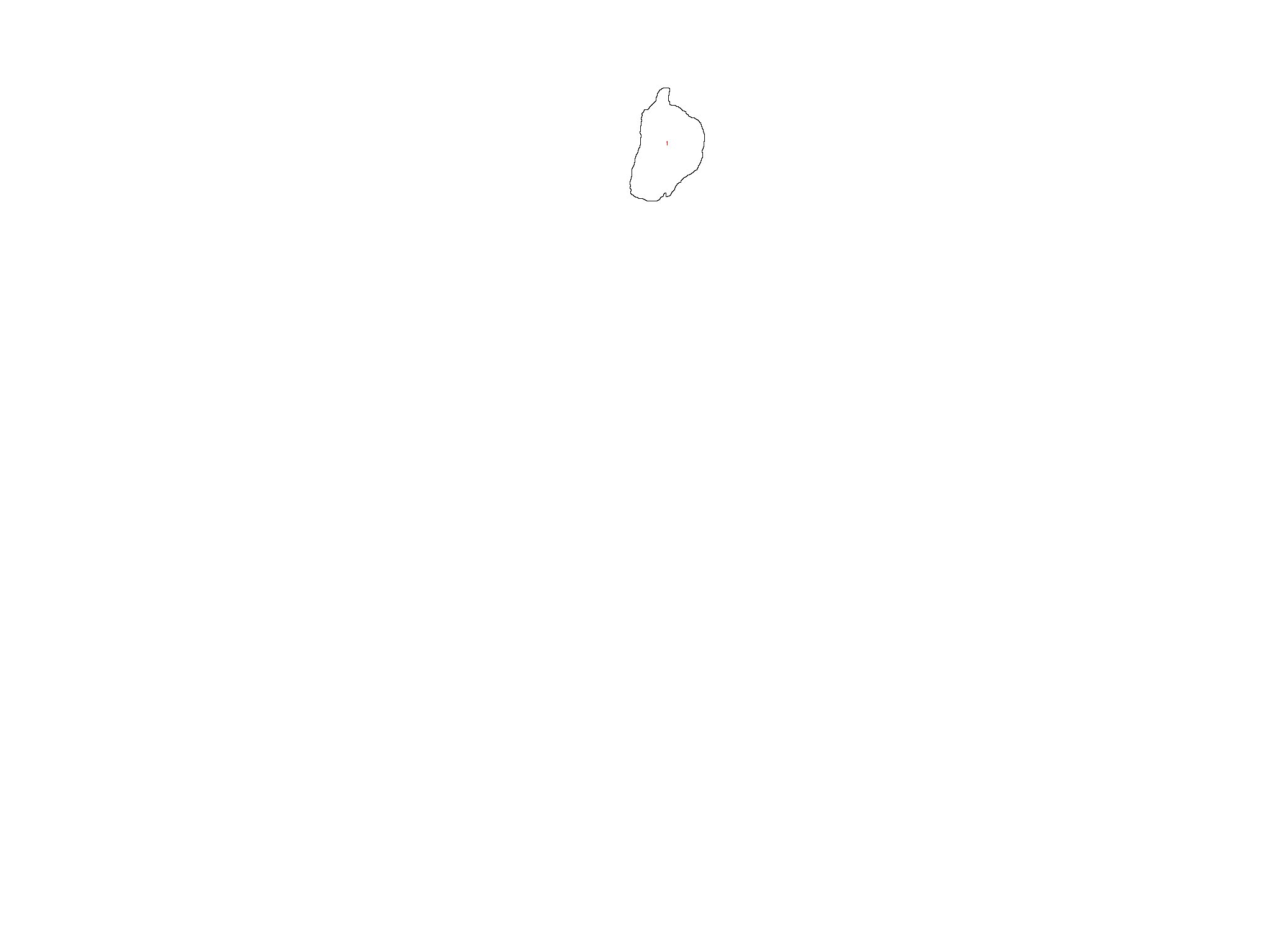

Supplement: S2 Dataset — (ZIP) [file pone.0304198.s005.zip › S2_Dataset_Raw_results_ImageJ/J2_100F_190200_1.jpg]

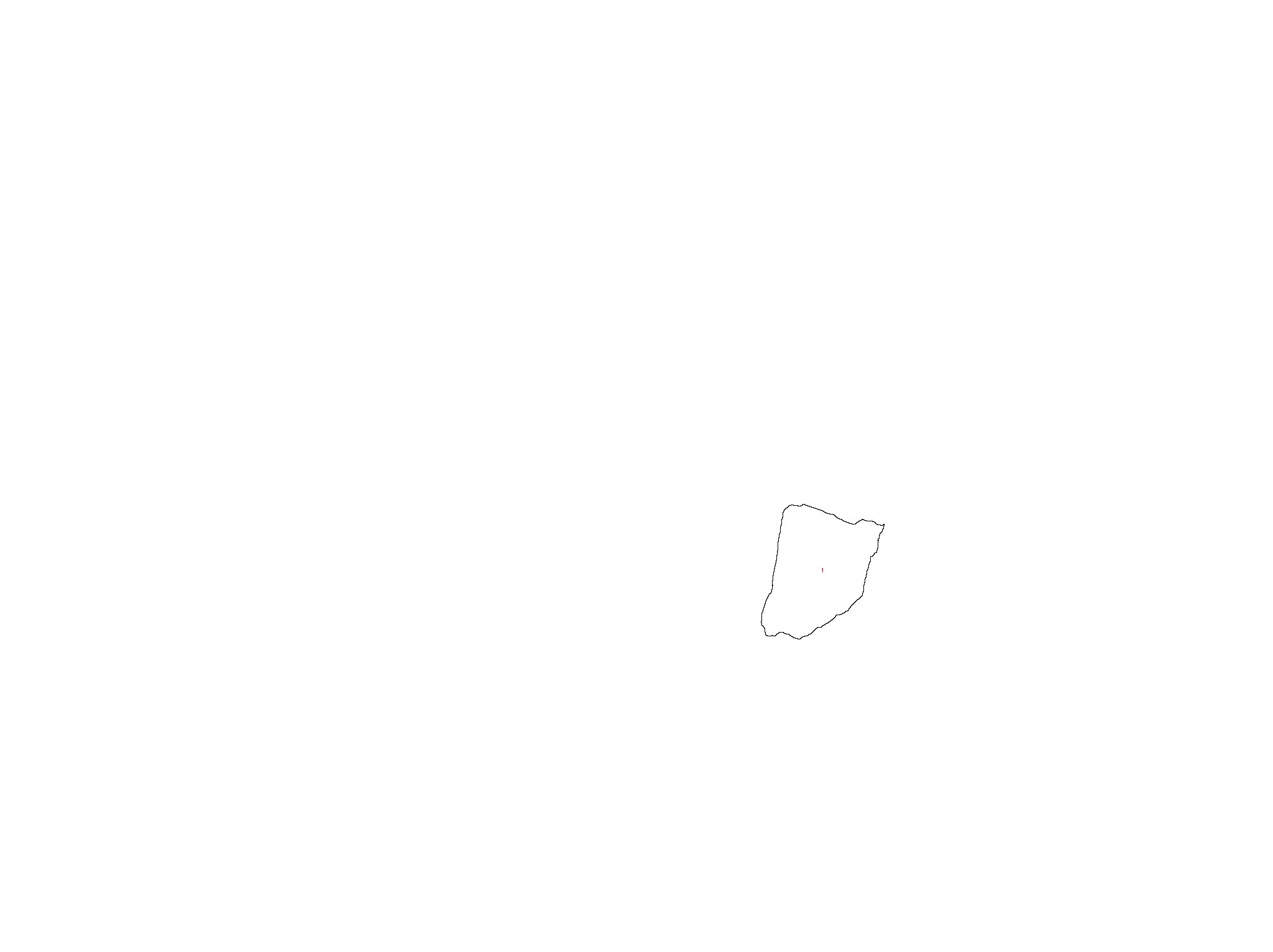

Supplement: S2 Dataset — (ZIP) [file pone.0304198.s005.zip › S2_Dataset_Raw_results_ImageJ/J2_100F_5060_1.jpg]

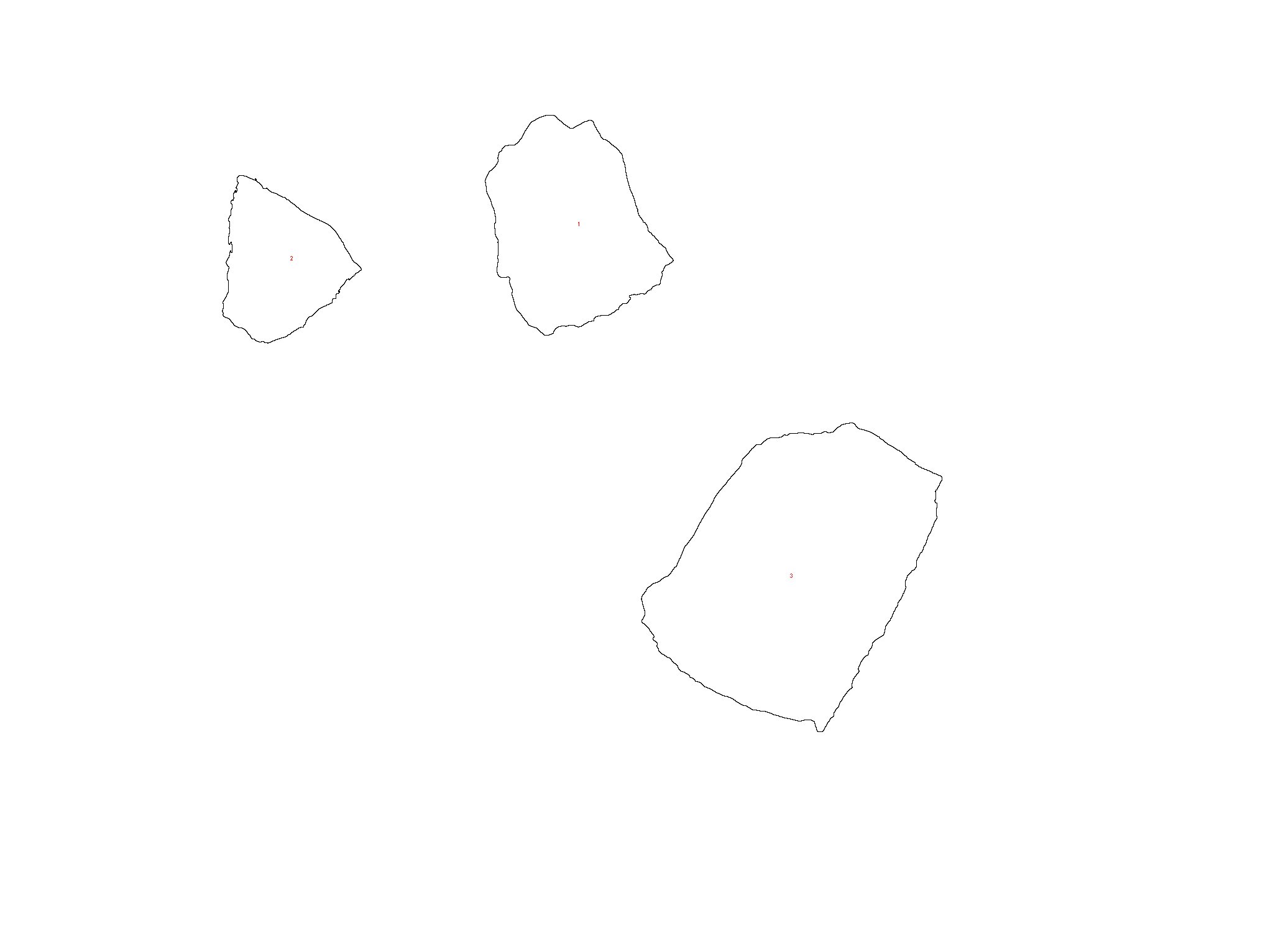

Supplement: S2 Dataset — (ZIP) [file pone.0304198.s005.zip › S2_Dataset_Raw_results_ImageJ/J2_100F_5060_10.jpg]

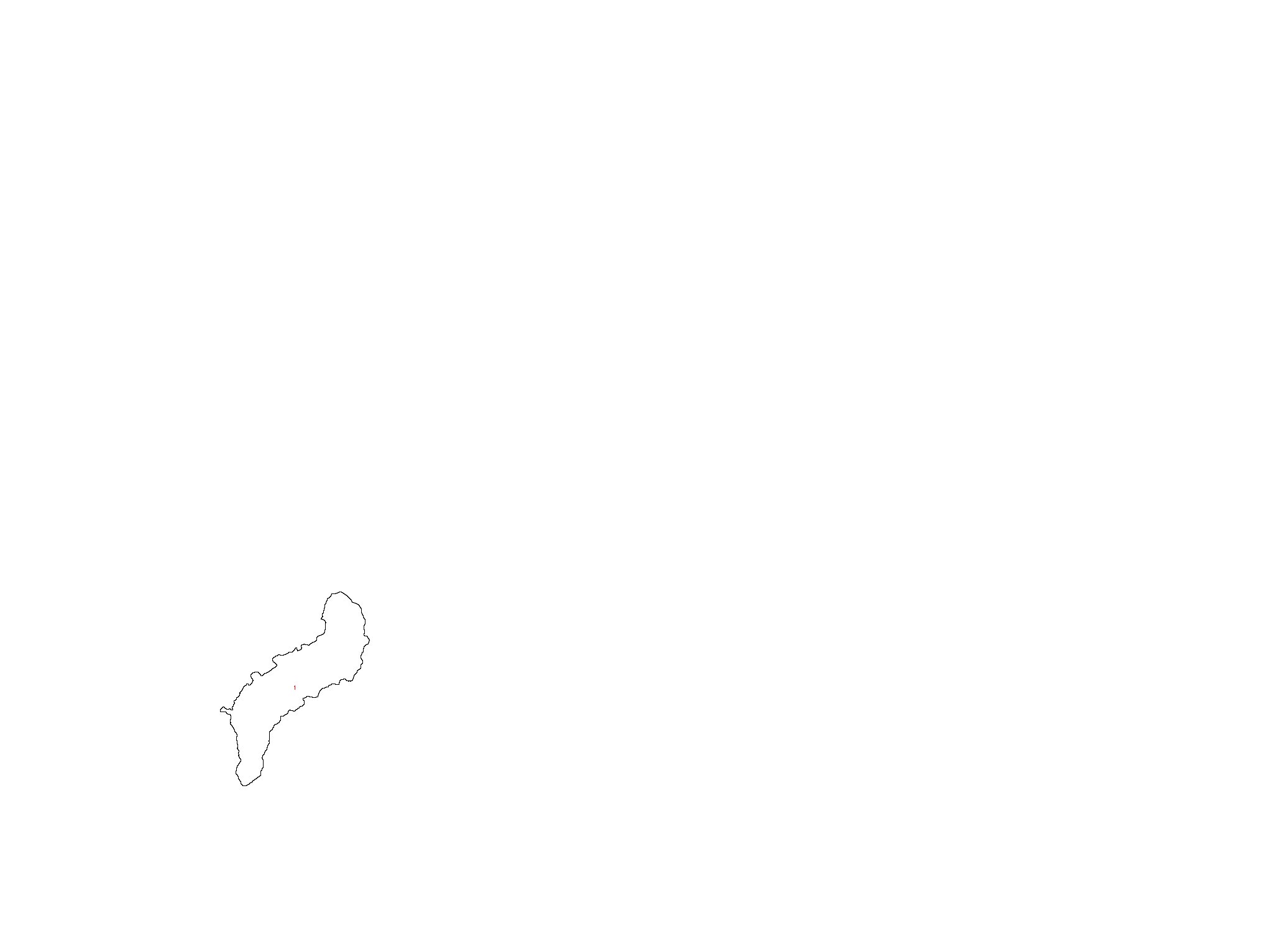

Supplement: S2 Dataset — (ZIP) [file pone.0304198.s005.zip › S2_Dataset_Raw_results_ImageJ/J2_100F_5060_11.jpg]

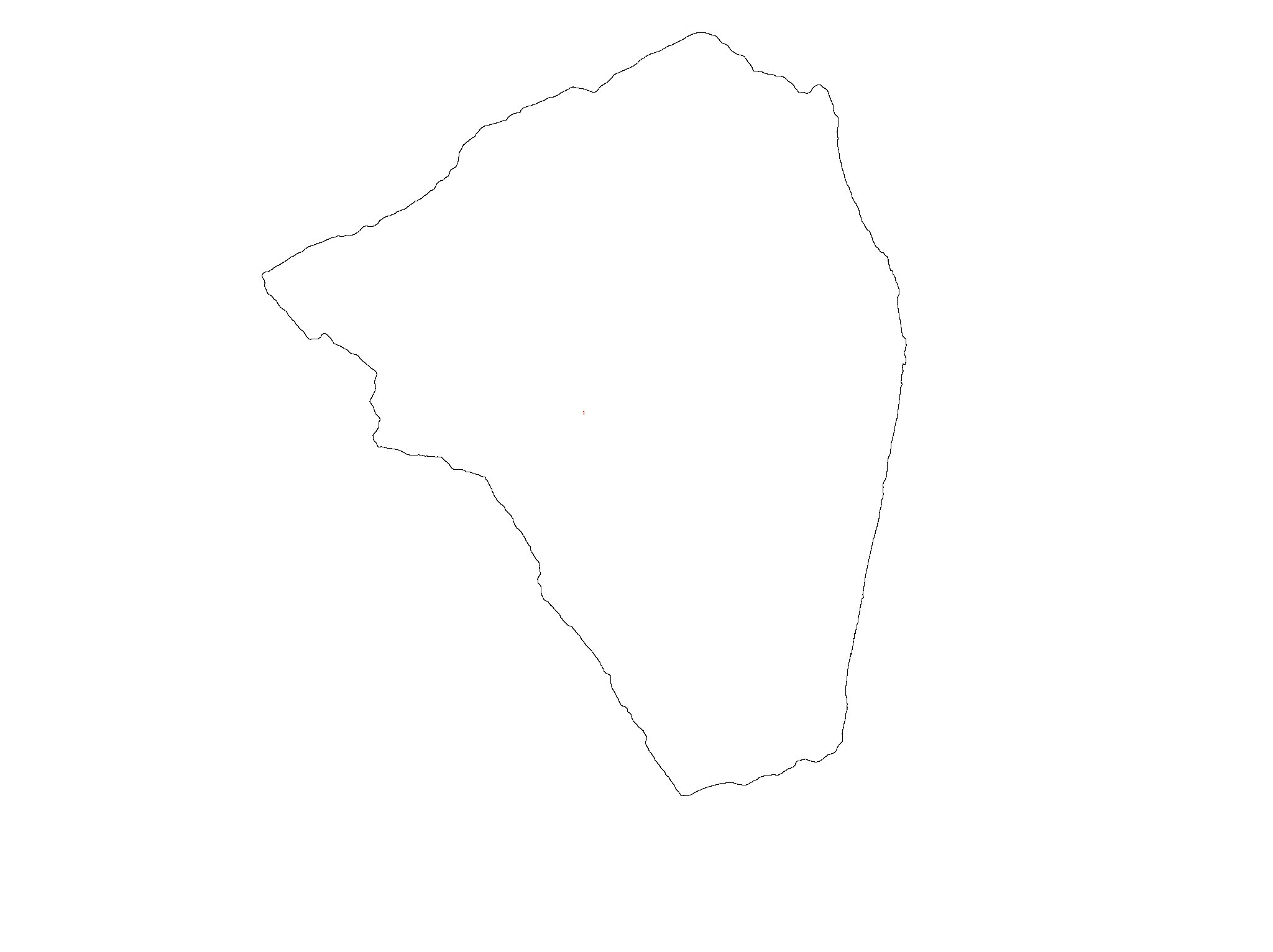

Supplement: S2 Dataset — (ZIP) [file pone.0304198.s005.zip › S2_Dataset_Raw_results_ImageJ/J2_100F_5060_12.jpg]

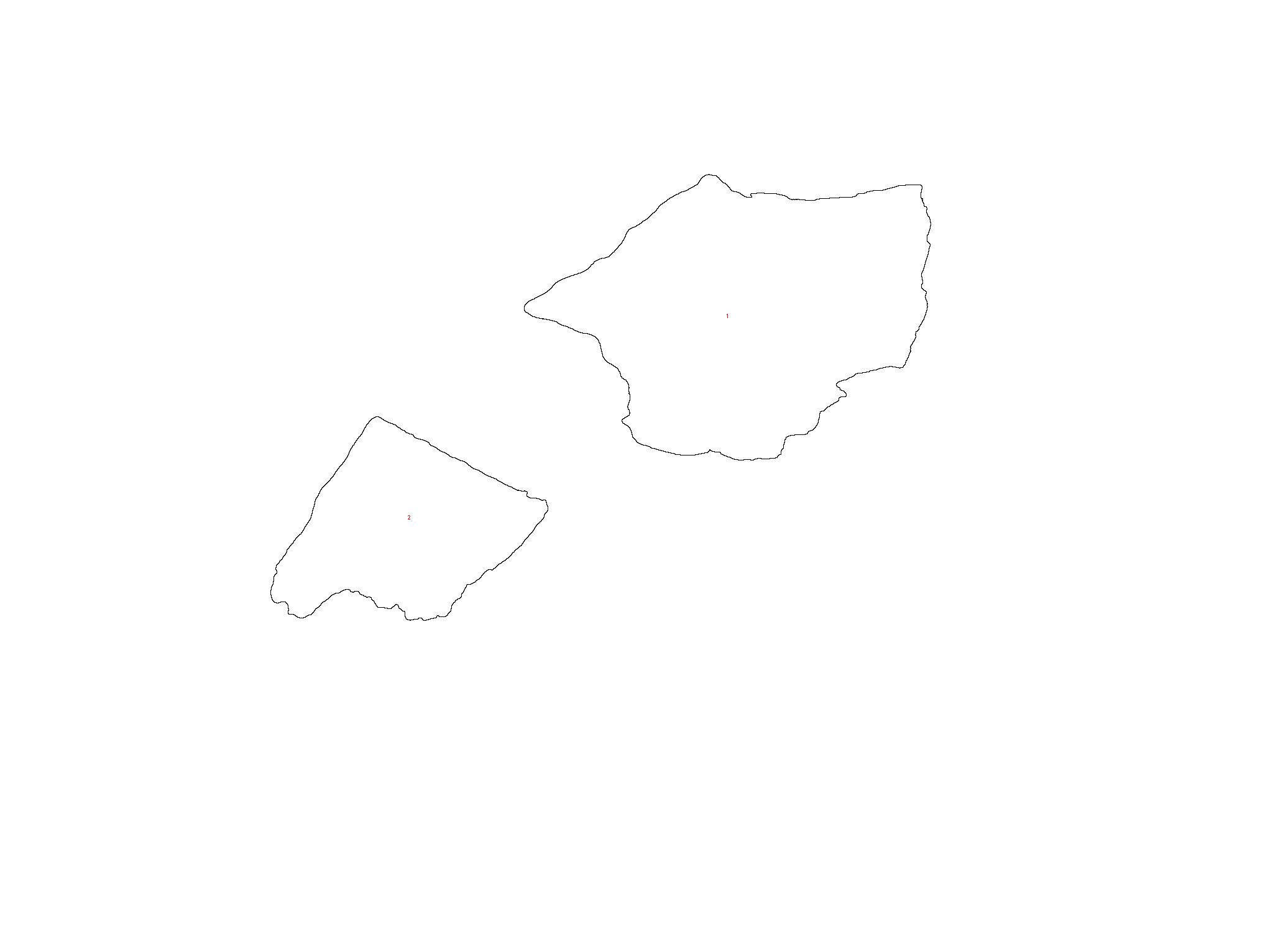

Supplement: S2 Dataset — (ZIP) [file pone.0304198.s005.zip › S2_Dataset_Raw_results_ImageJ/J2_100F_5060_13.jpg]

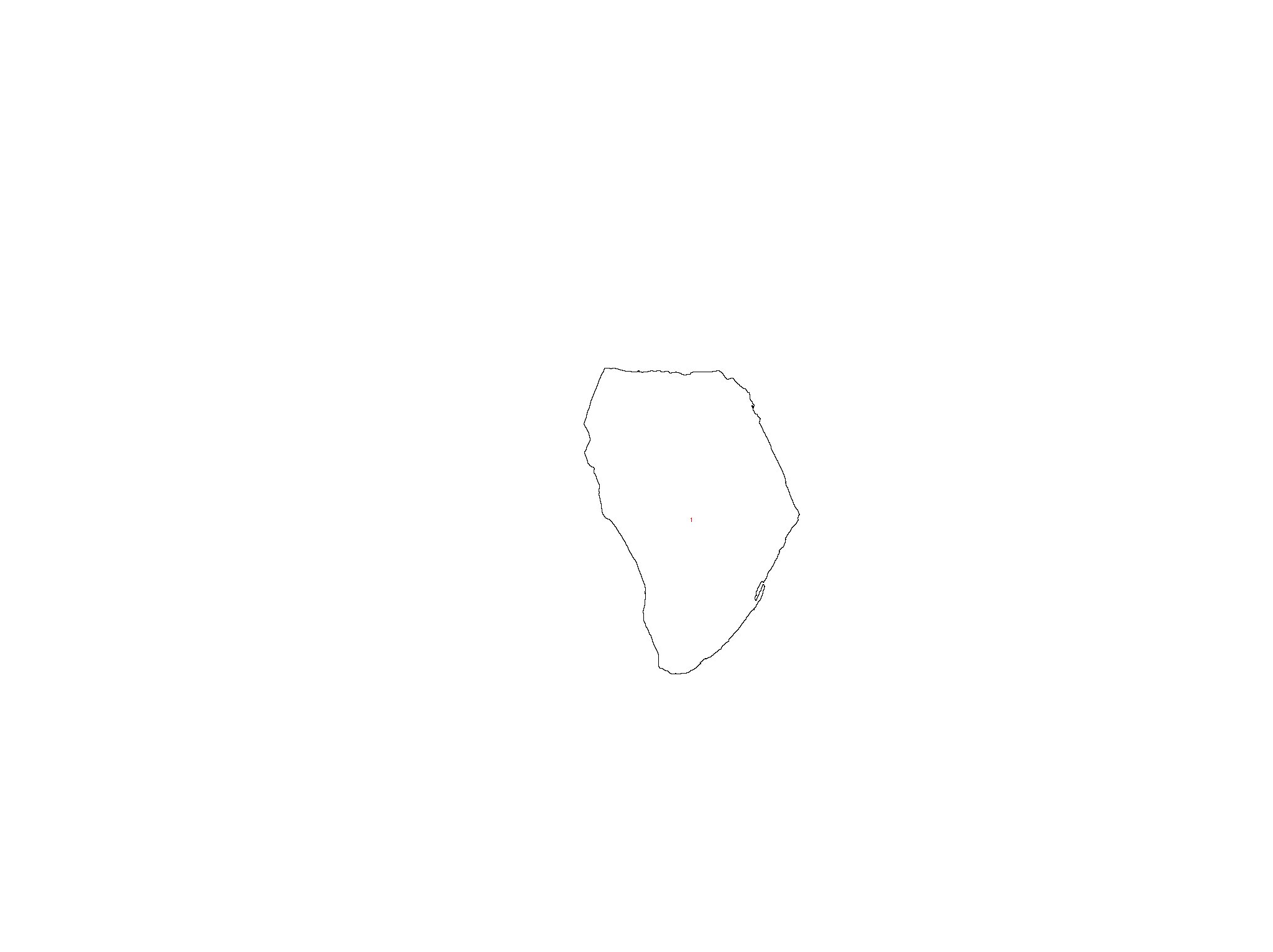

Supplement: S2 Dataset — (ZIP) [file pone.0304198.s005.zip › S2_Dataset_Raw_results_ImageJ/J2_100F_5060_14.jpg]

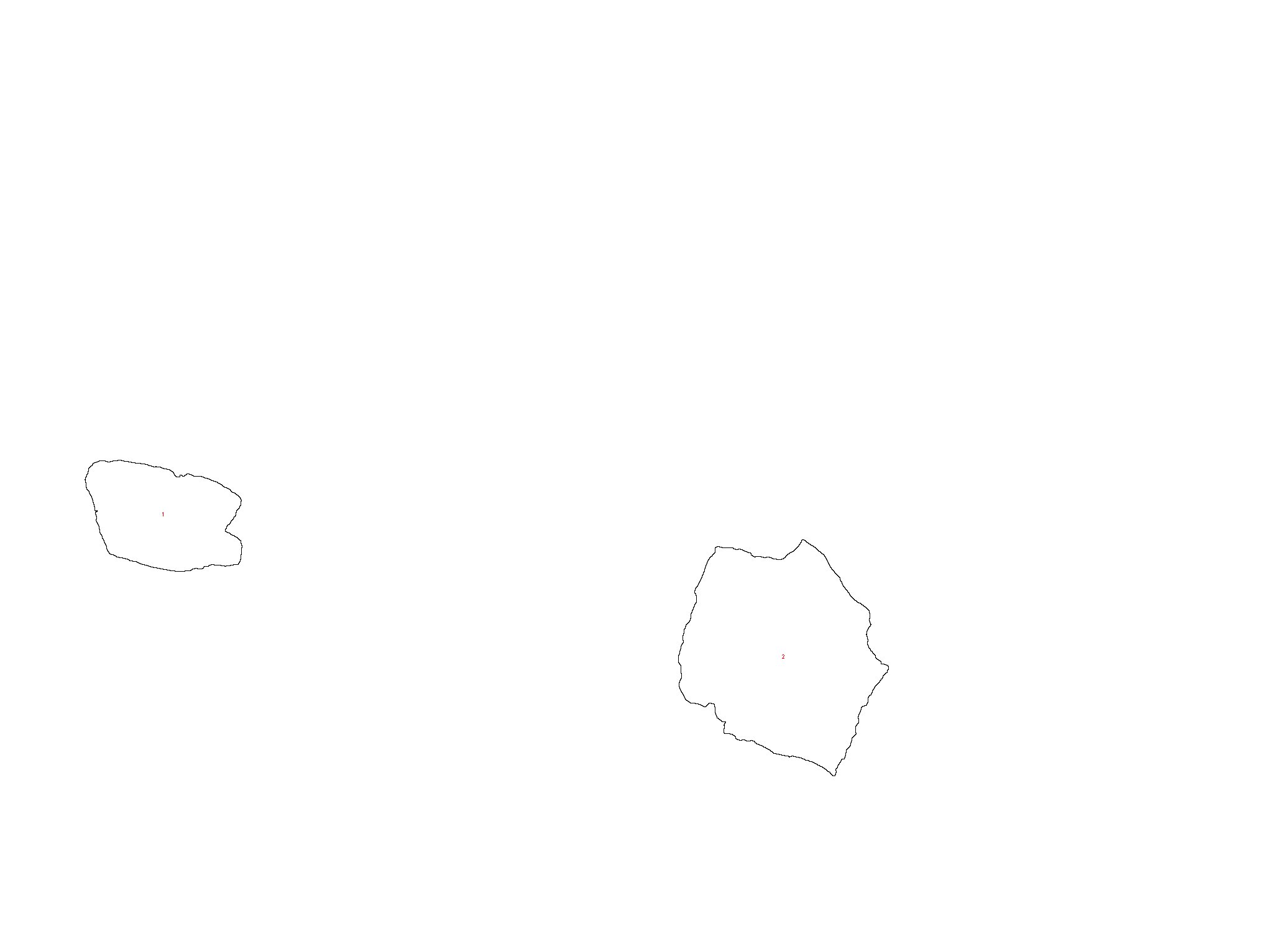

Supplement: S2 Dataset — (ZIP) [file pone.0304198.s005.zip › S2_Dataset_Raw_results_ImageJ/J2_100F_5060_15.jpg]

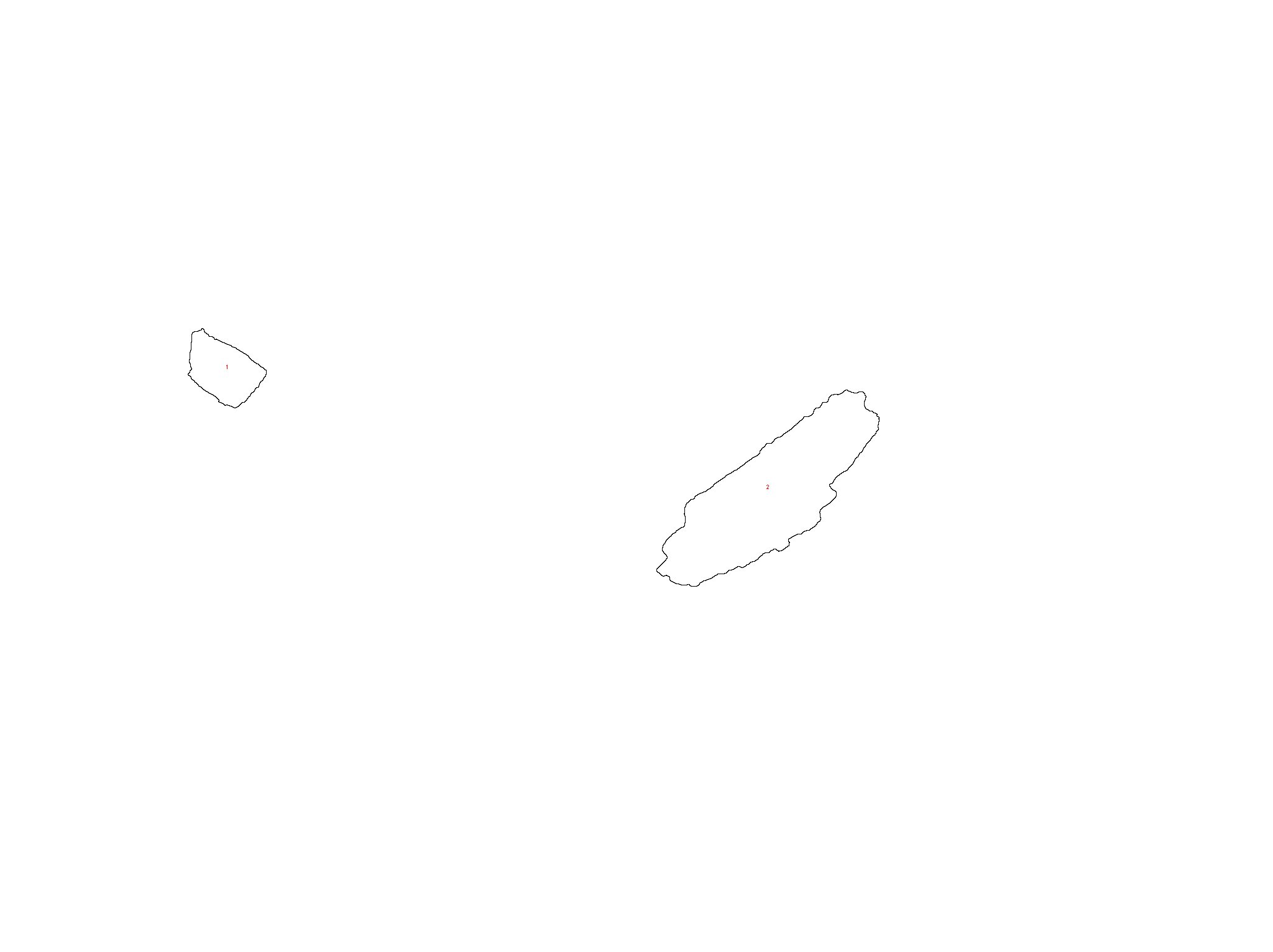

Supplement: S2 Dataset — (ZIP) [file pone.0304198.s005.zip › S2_Dataset_Raw_results_ImageJ/J2_100F_5060_16.jpg]

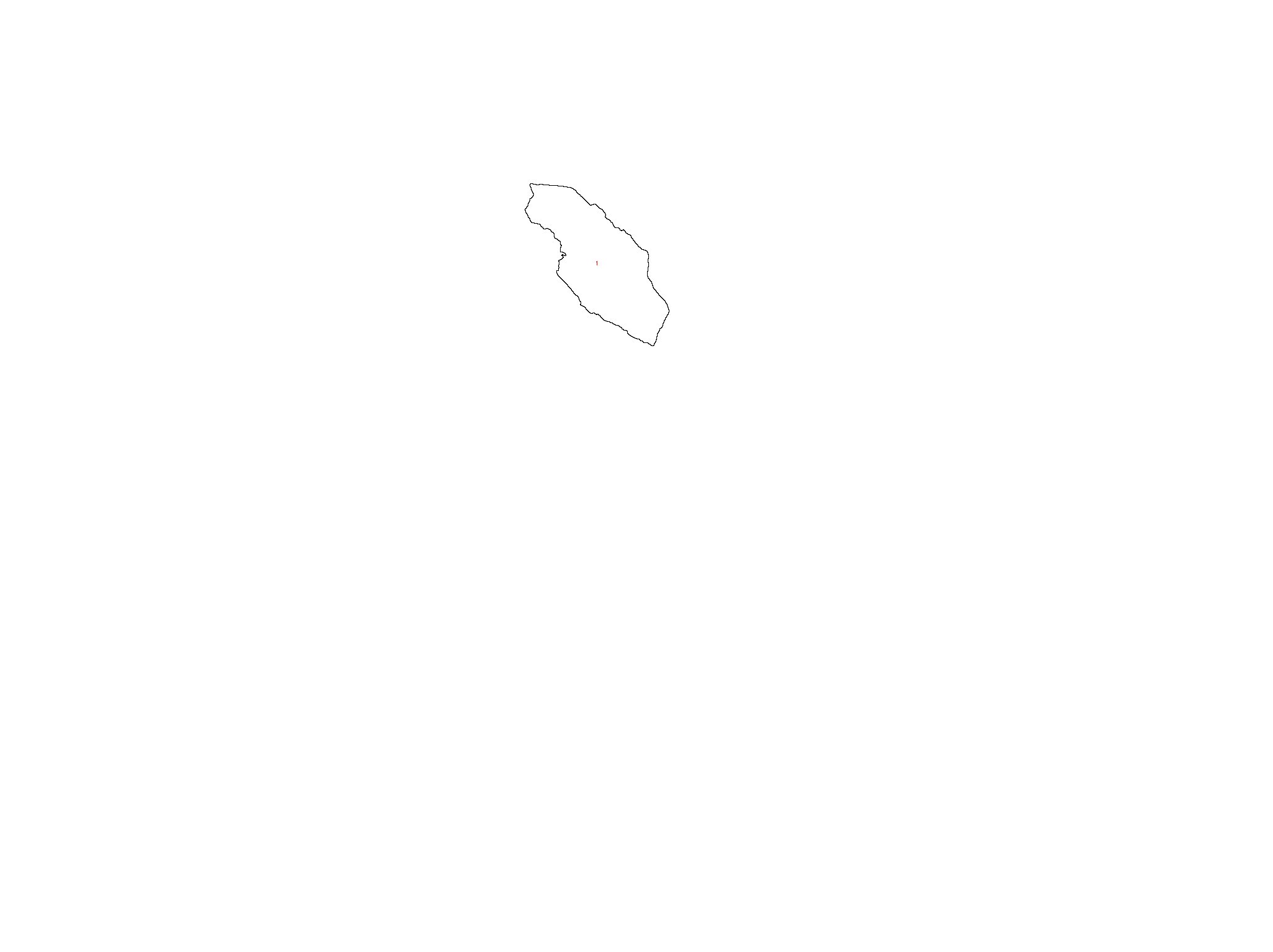

Supplement: S2 Dataset — (ZIP) [file pone.0304198.s005.zip › S2_Dataset_Raw_results_ImageJ/J2_100F_5060_17.jpg]

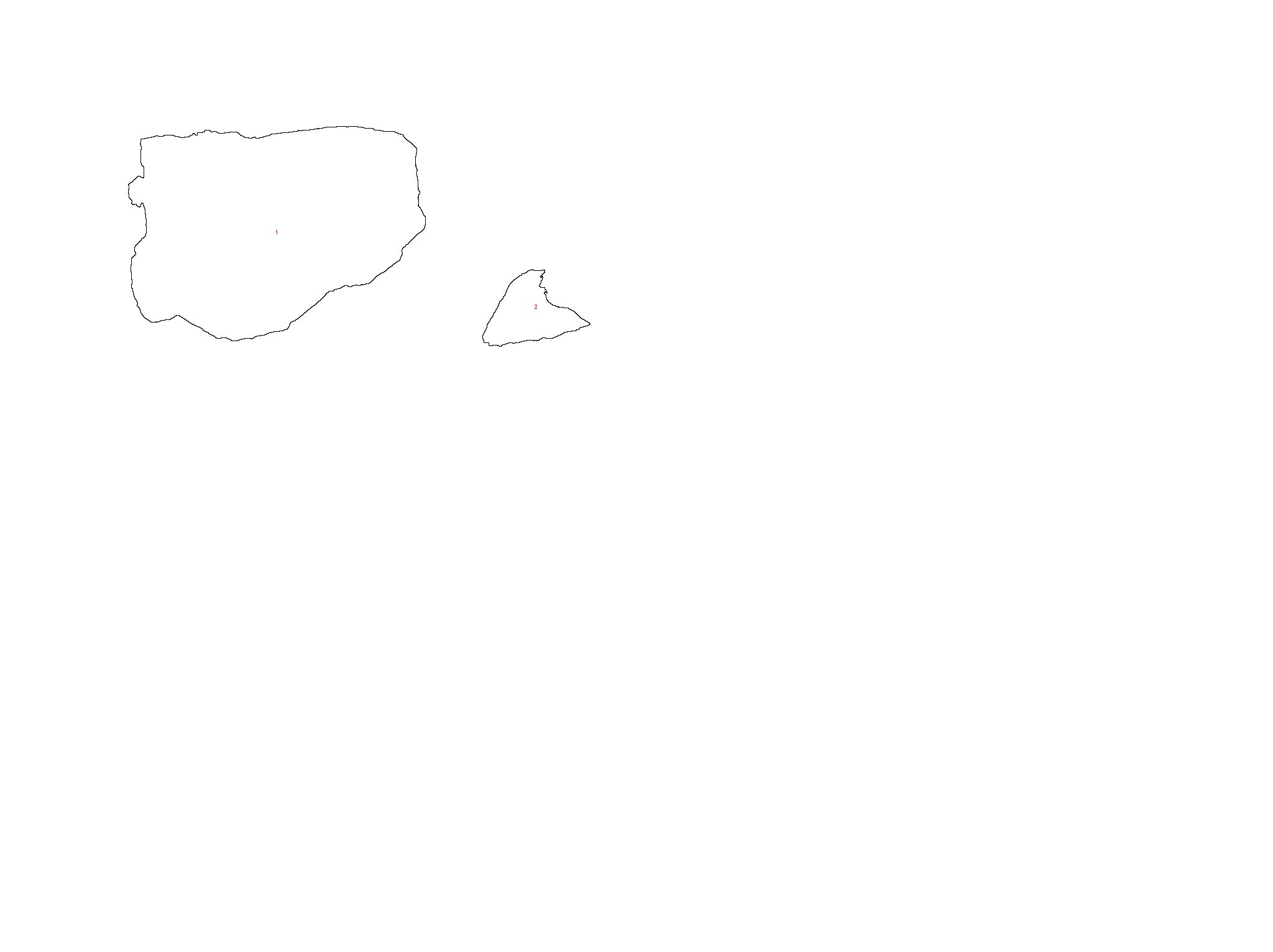

Supplement: S2 Dataset — (ZIP) [file pone.0304198.s005.zip › S2_Dataset_Raw_results_ImageJ/J2_100F_5060_18.jpg]

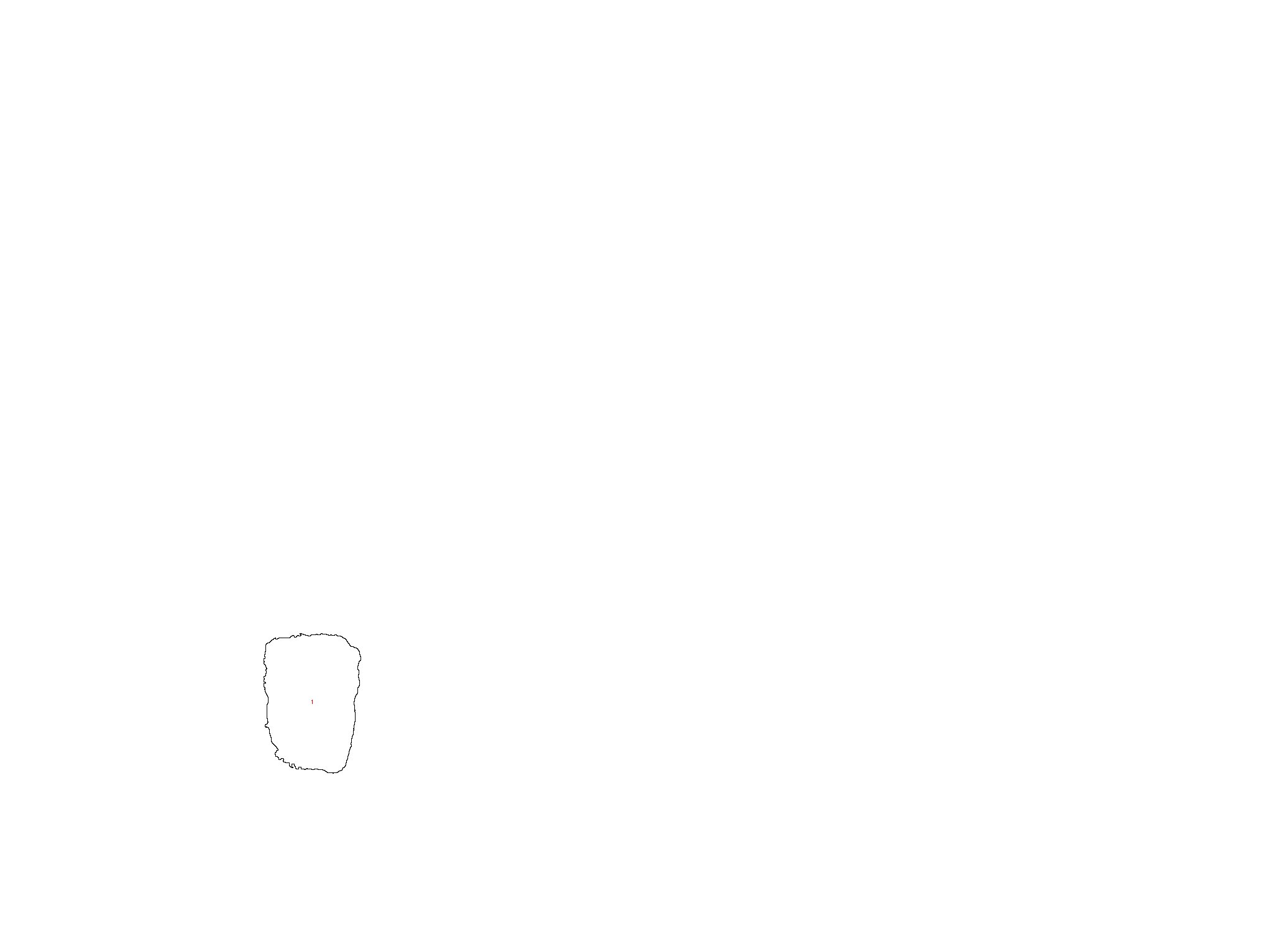

Supplement: S2 Dataset — (ZIP) [file pone.0304198.s005.zip › S2_Dataset_Raw_results_ImageJ/J2_100F_5060_2.jpg]

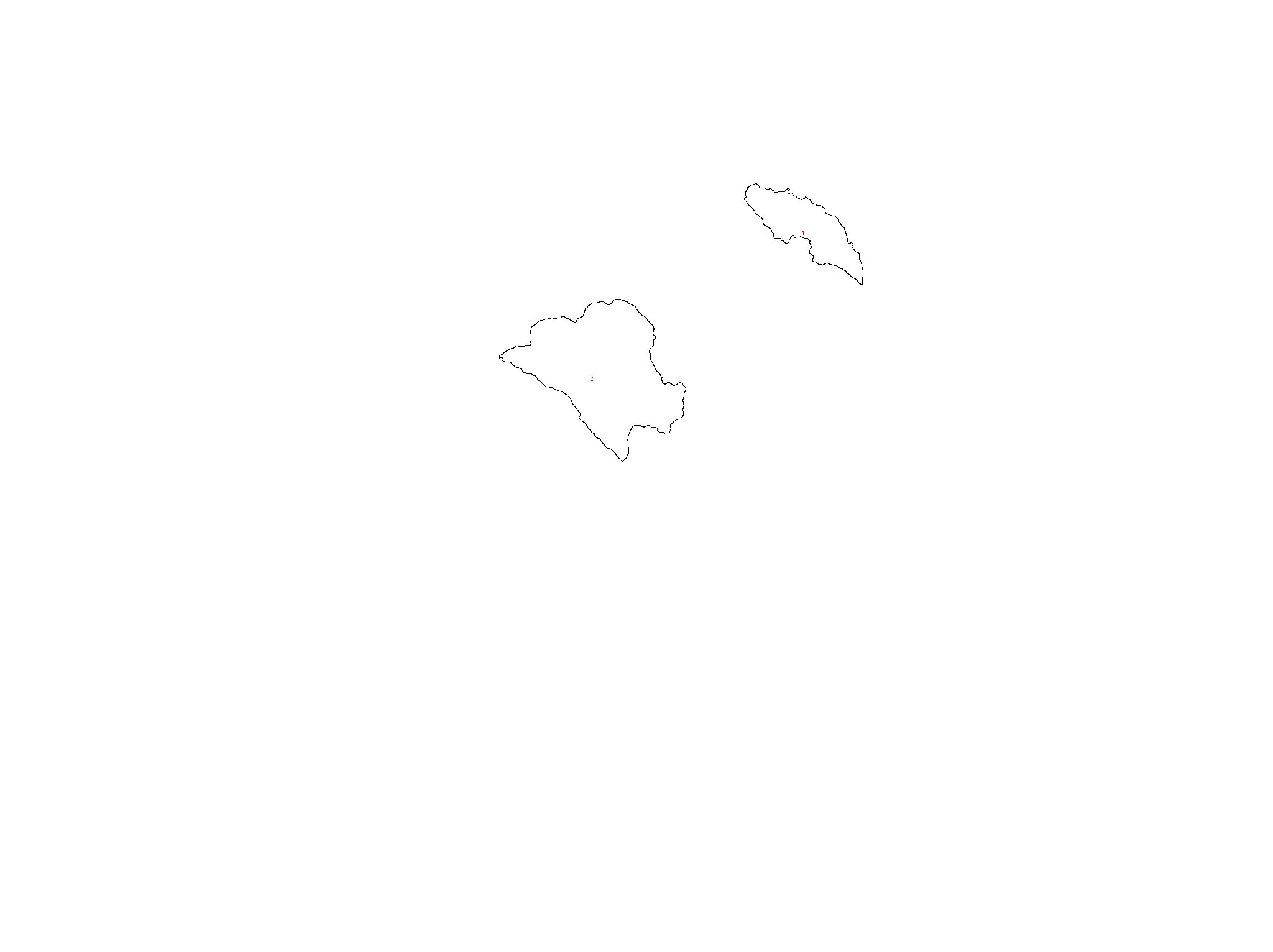

Supplement: S2 Dataset — (ZIP) [file pone.0304198.s005.zip › S2_Dataset_Raw_results_ImageJ/J2_100F_5060_3.jpg]

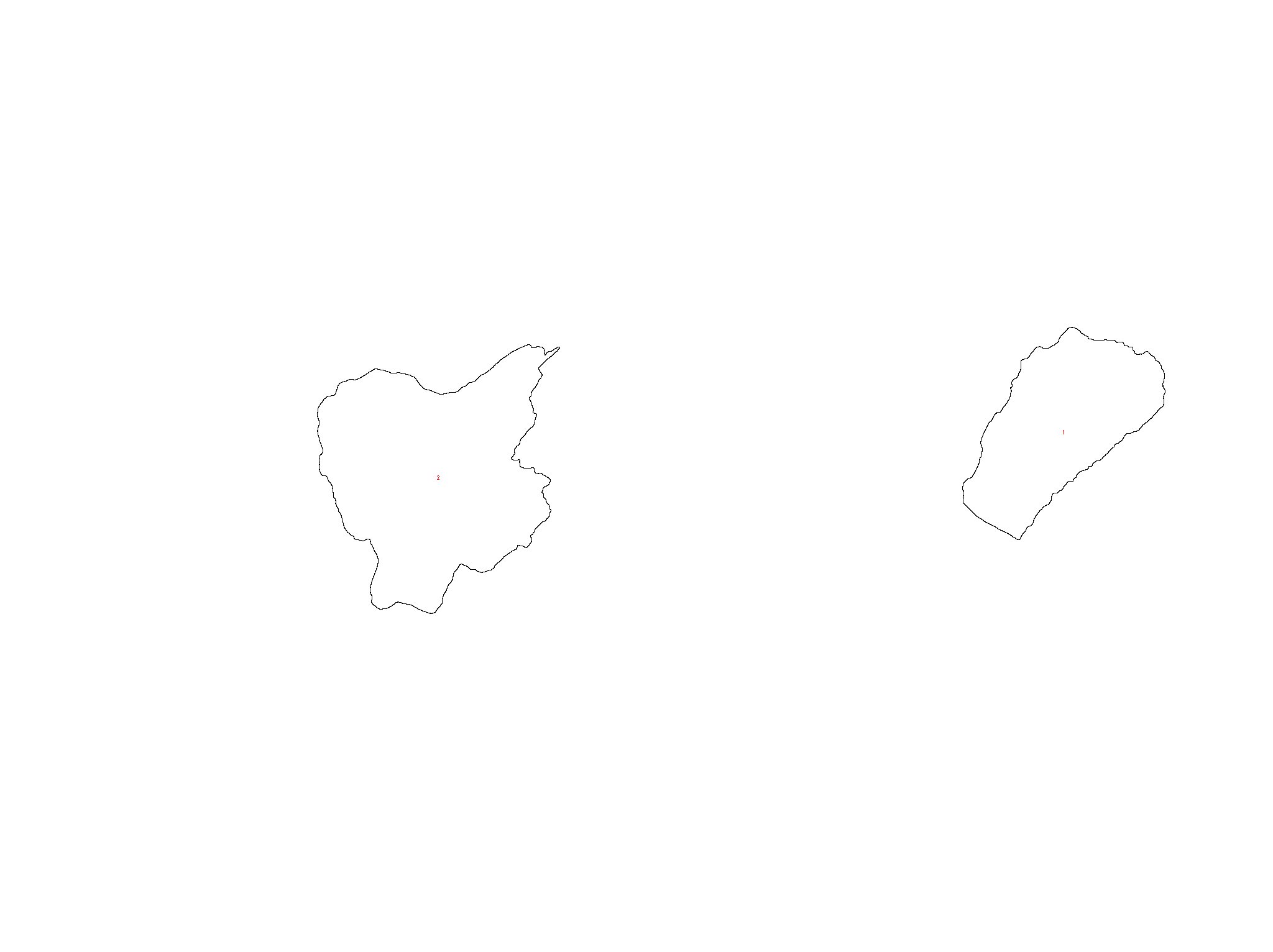

Supplement: S2 Dataset — (ZIP) [file pone.0304198.s005.zip › S2_Dataset_Raw_results_ImageJ/J2_100F_5060_4.jpg]

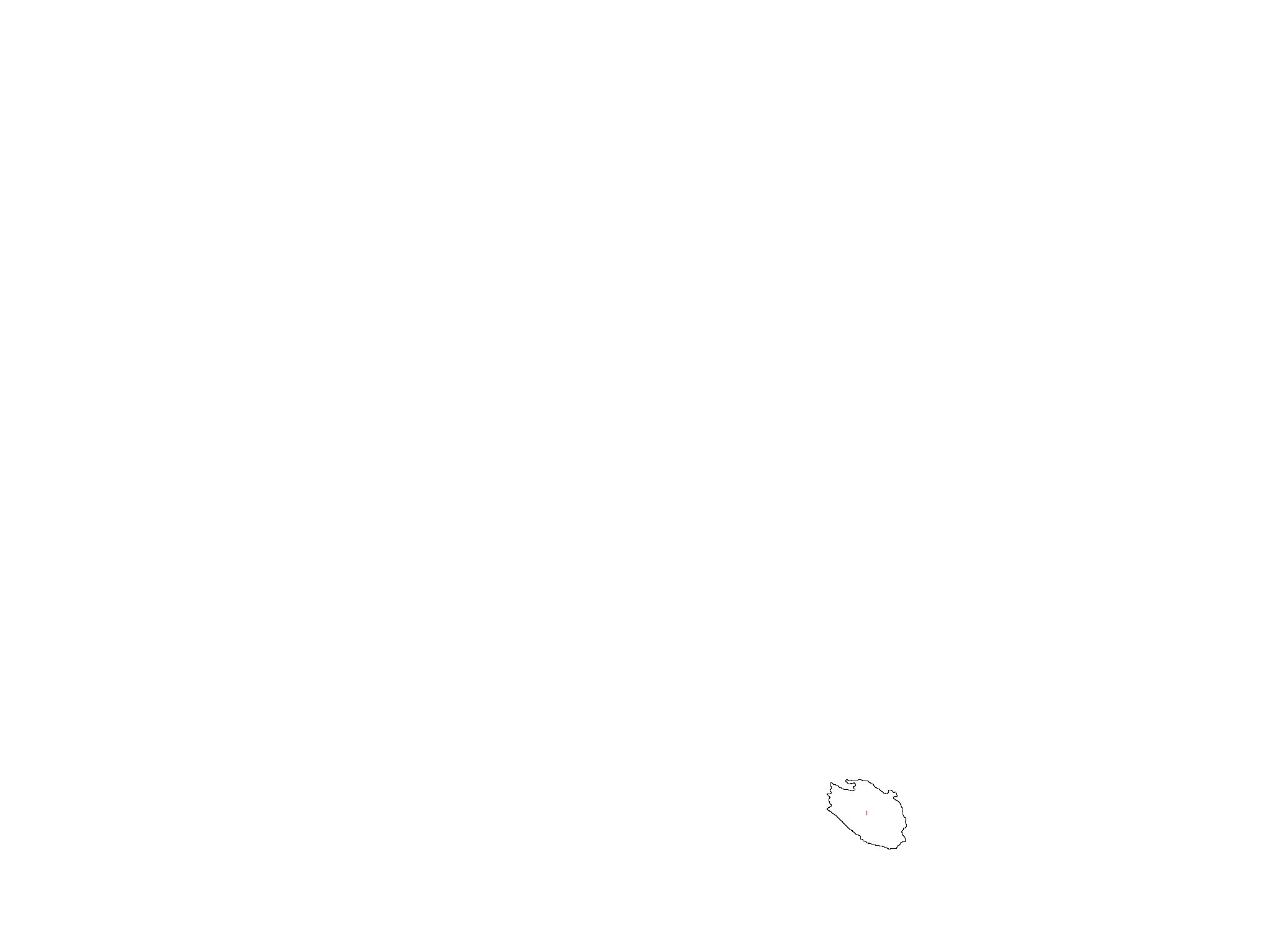

Supplement: S2 Dataset — (ZIP) [file pone.0304198.s005.zip › S2_Dataset_Raw_results_ImageJ/J2_100F_5060_5.jpg]

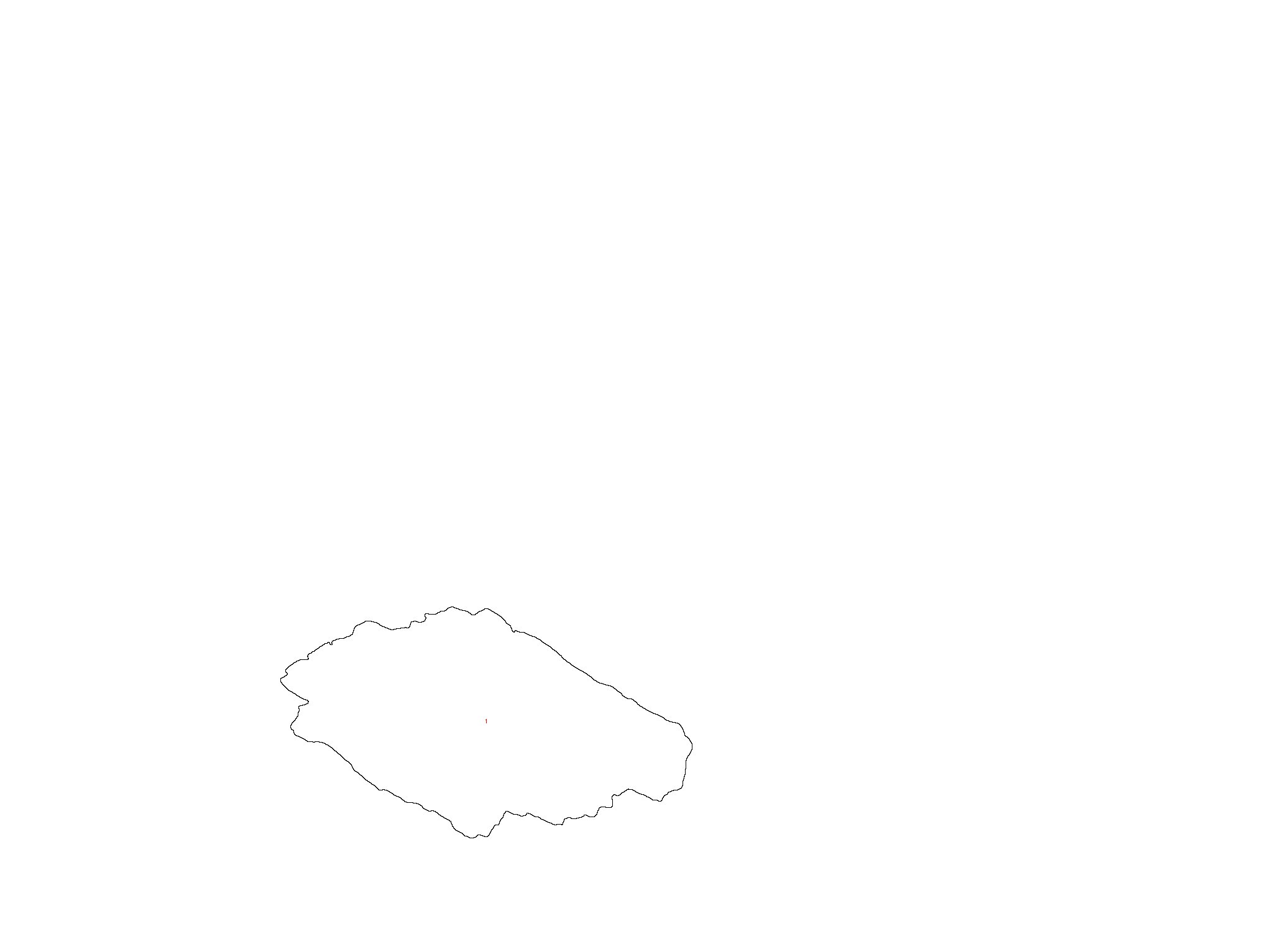

Supplement: S2 Dataset — (ZIP) [file pone.0304198.s005.zip › S2_Dataset_Raw_results_ImageJ/J2_100F_5060_6.jpg]

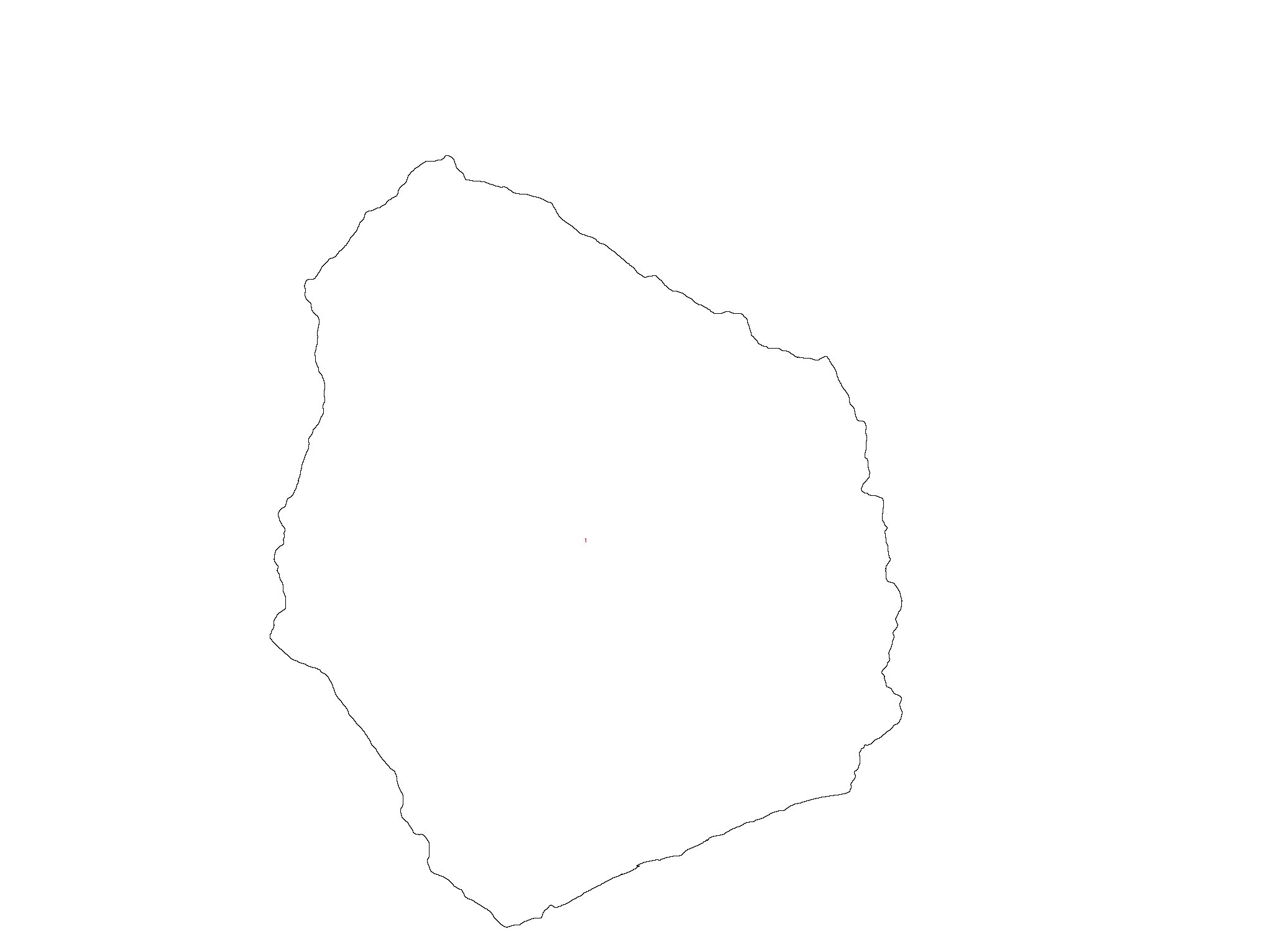

Supplement: S2 Dataset — (ZIP) [file pone.0304198.s005.zip › S2_Dataset_Raw_results_ImageJ/J2_100F_5060_7.jpg]

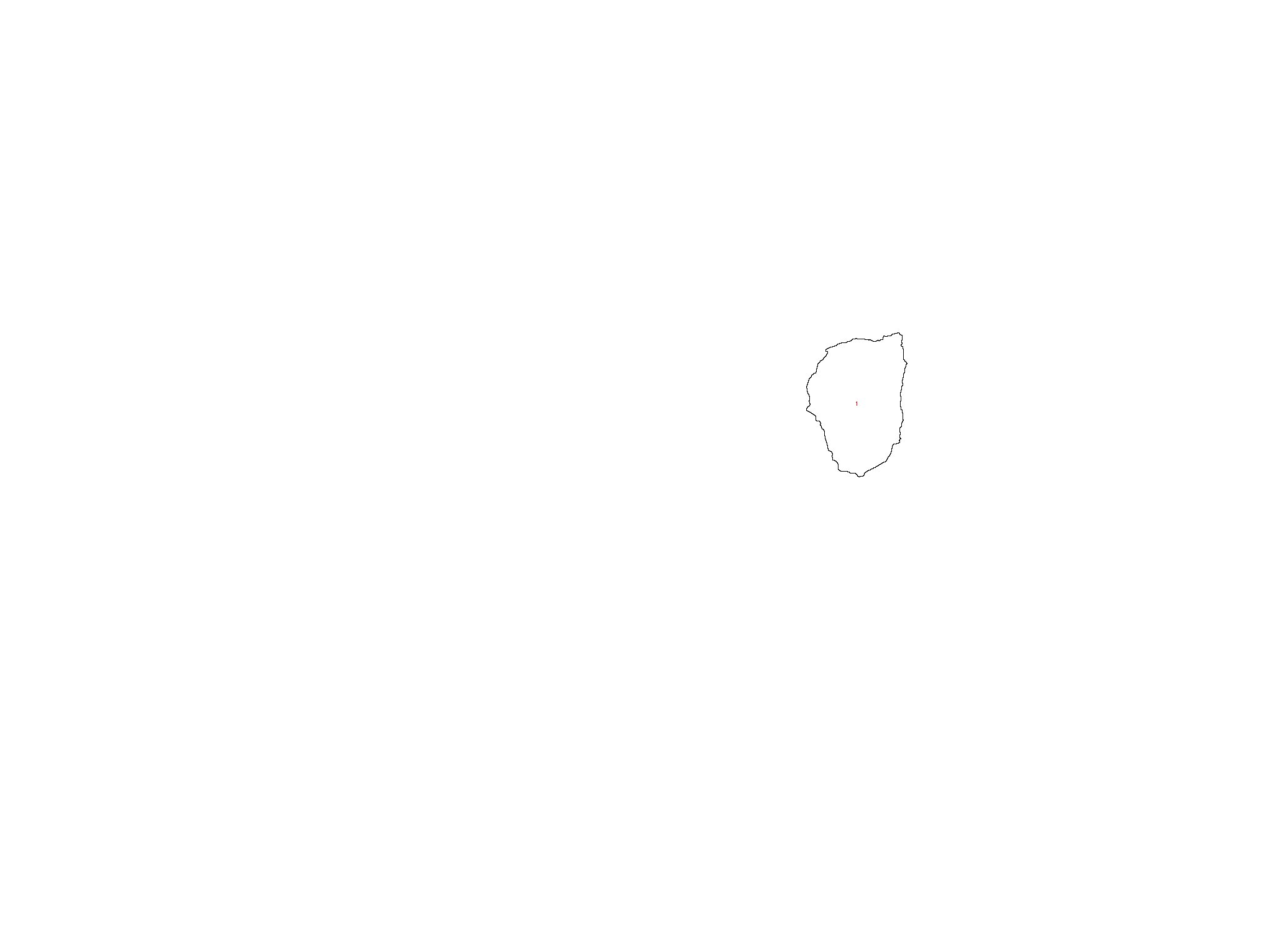

Supplement: S2 Dataset — (ZIP) [file pone.0304198.s005.zip › S2_Dataset_Raw_results_ImageJ/J2_100F_5060_8.jpg]

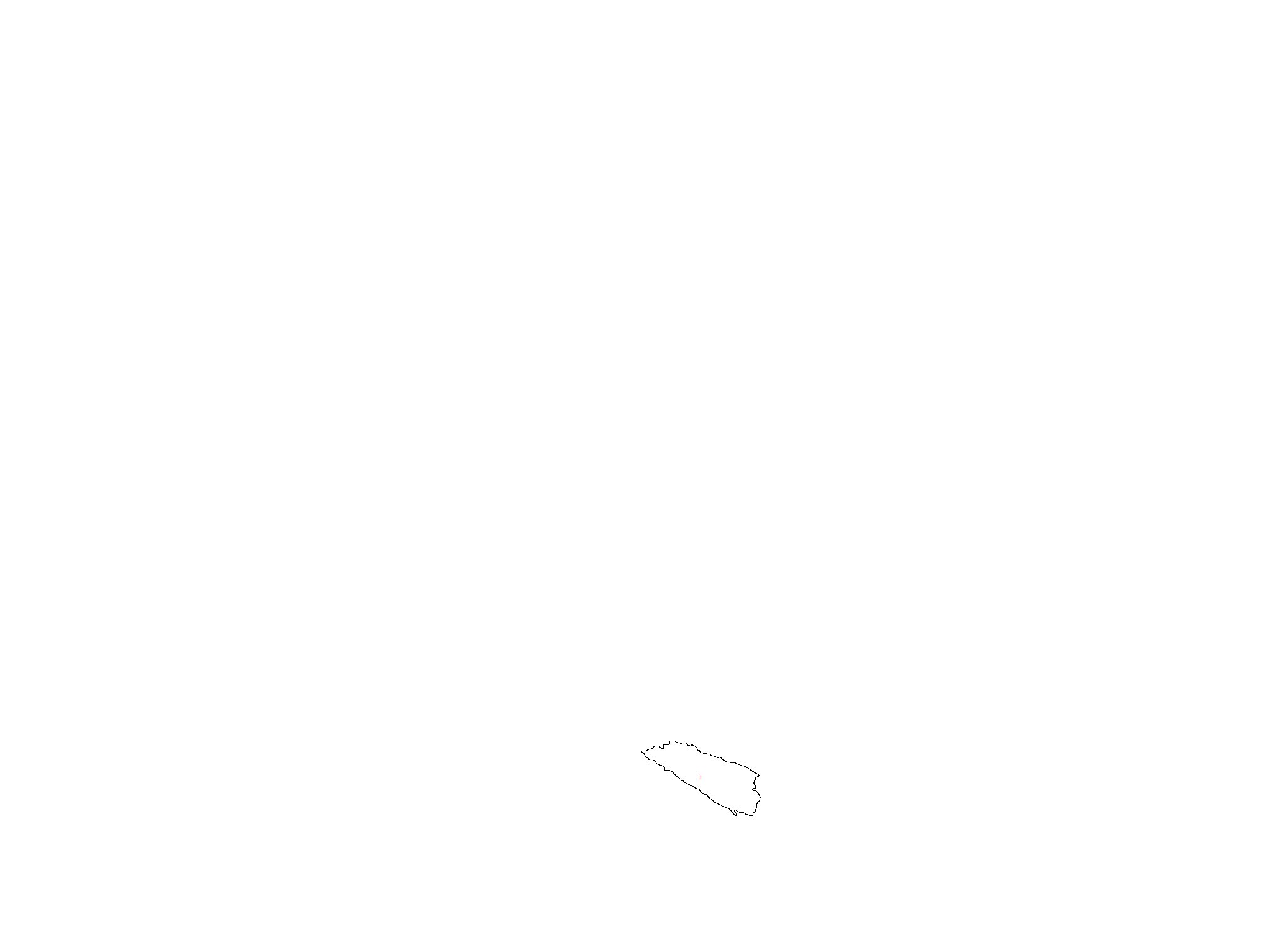

Supplement: S2 Dataset — (ZIP) [file pone.0304198.s005.zip › S2_Dataset_Raw_results_ImageJ/J2_100F_5060_9.jpg]

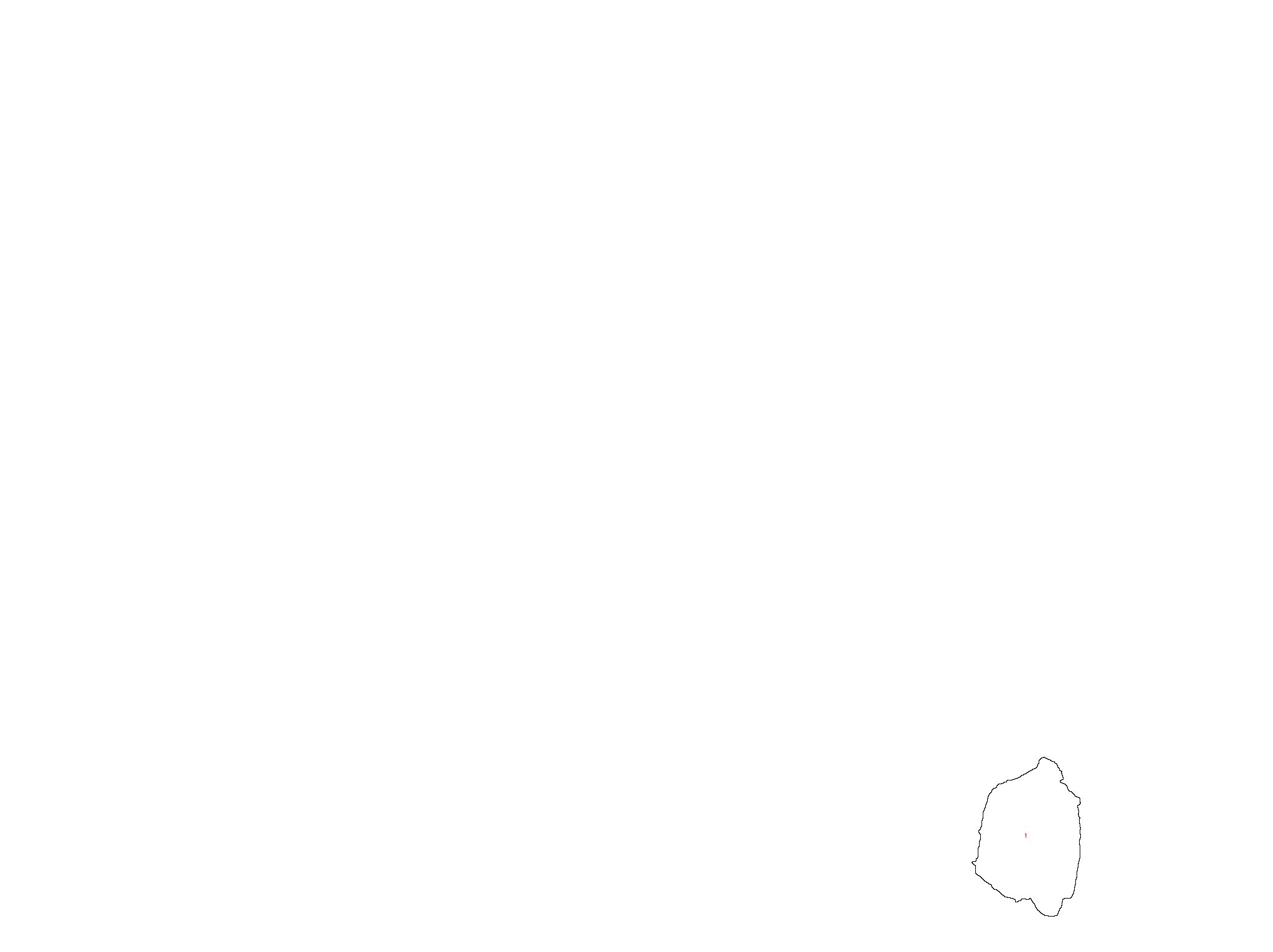

Supplement: S2 Dataset — (ZIP) [file pone.0304198.s005.zip › S2_Dataset_Raw_results_ImageJ/J2_100F_8090_1.jpg]

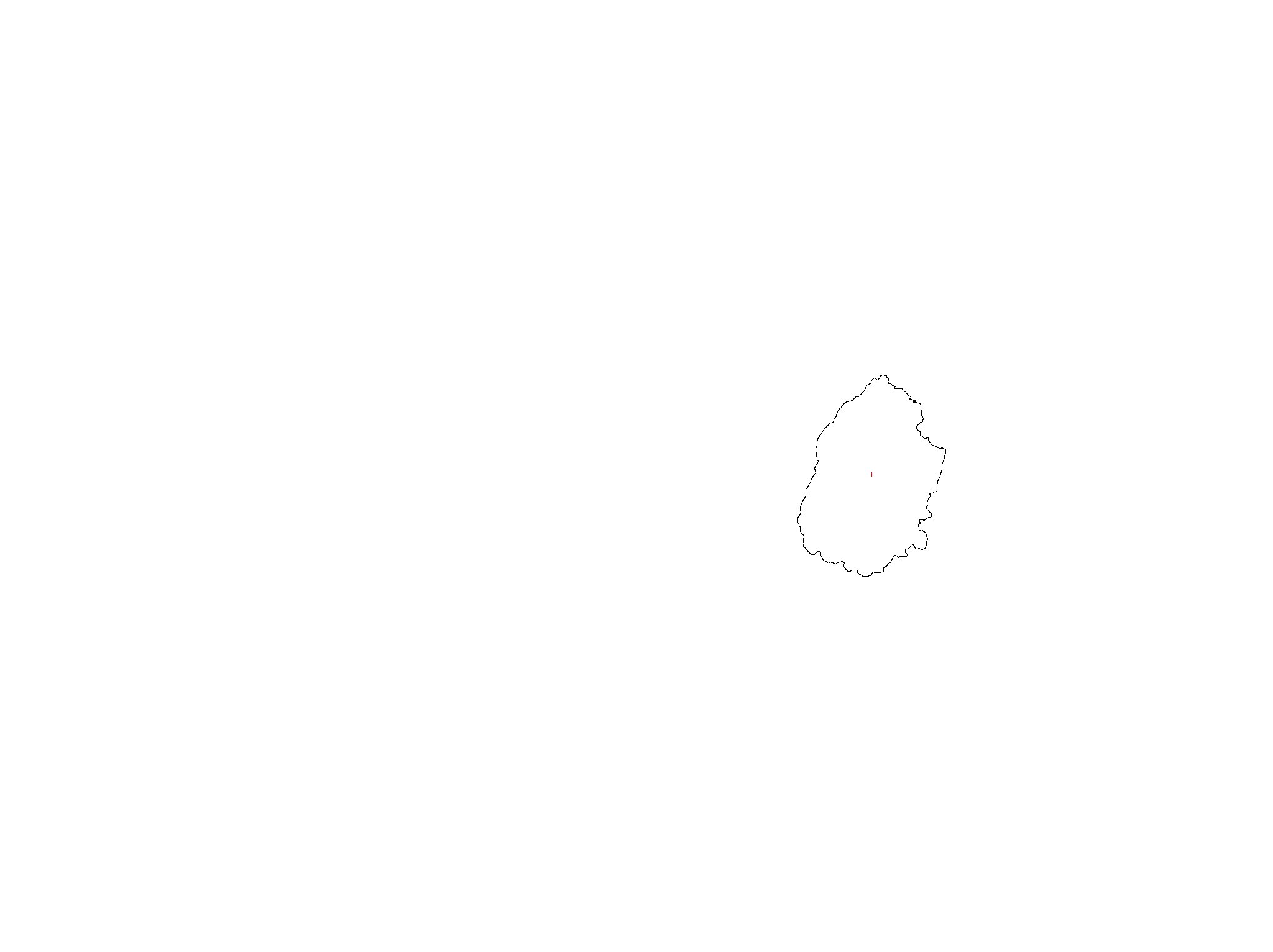

Supplement: S2 Dataset — (ZIP) [file pone.0304198.s005.zip › S2_Dataset_Raw_results_ImageJ/J2_100F_8090_2.jpg]

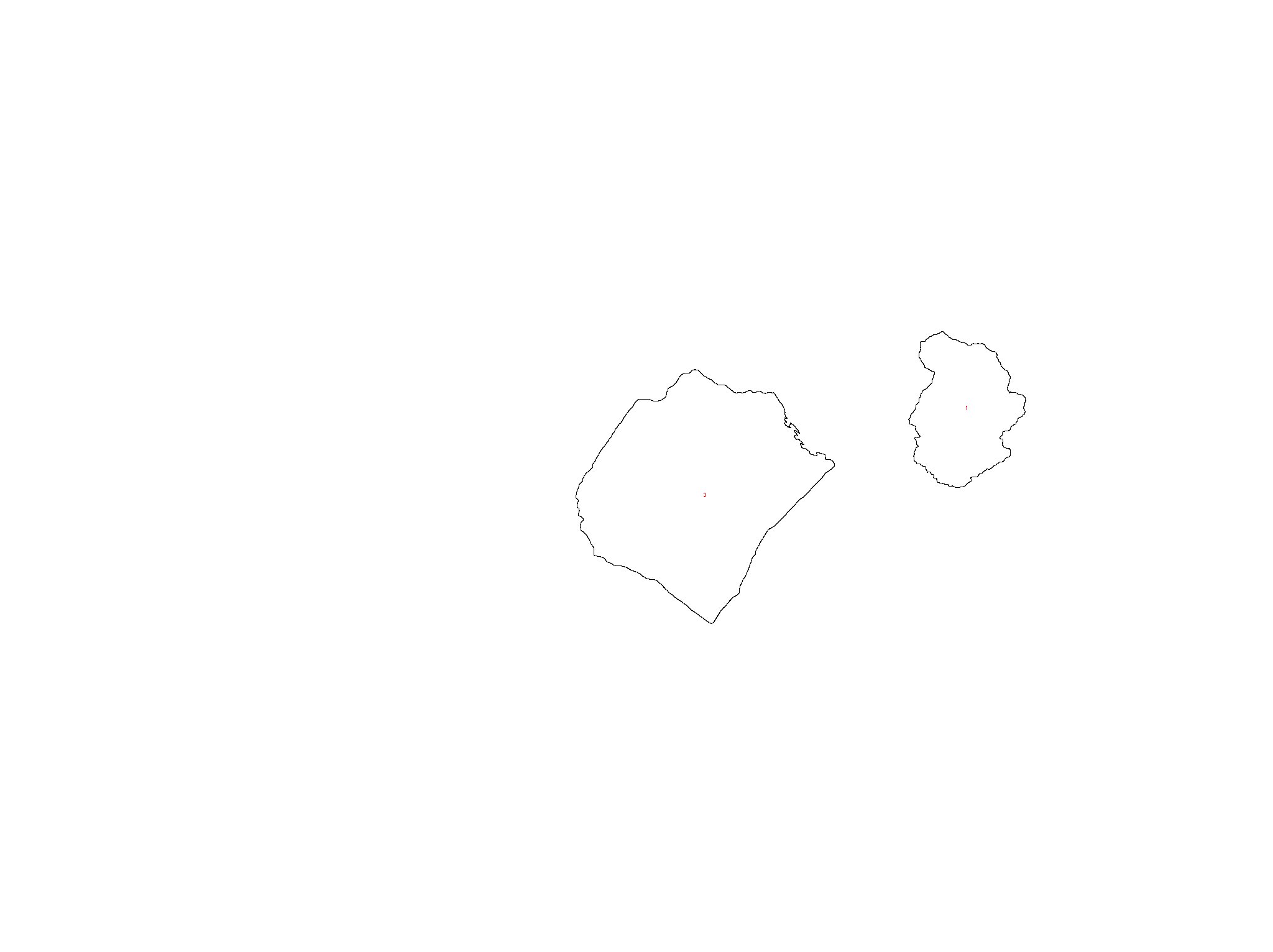

Supplement: S2 Dataset — (ZIP) [file pone.0304198.s005.zip › S2_Dataset_Raw_results_ImageJ/J2_100F_8090_3.jpg]

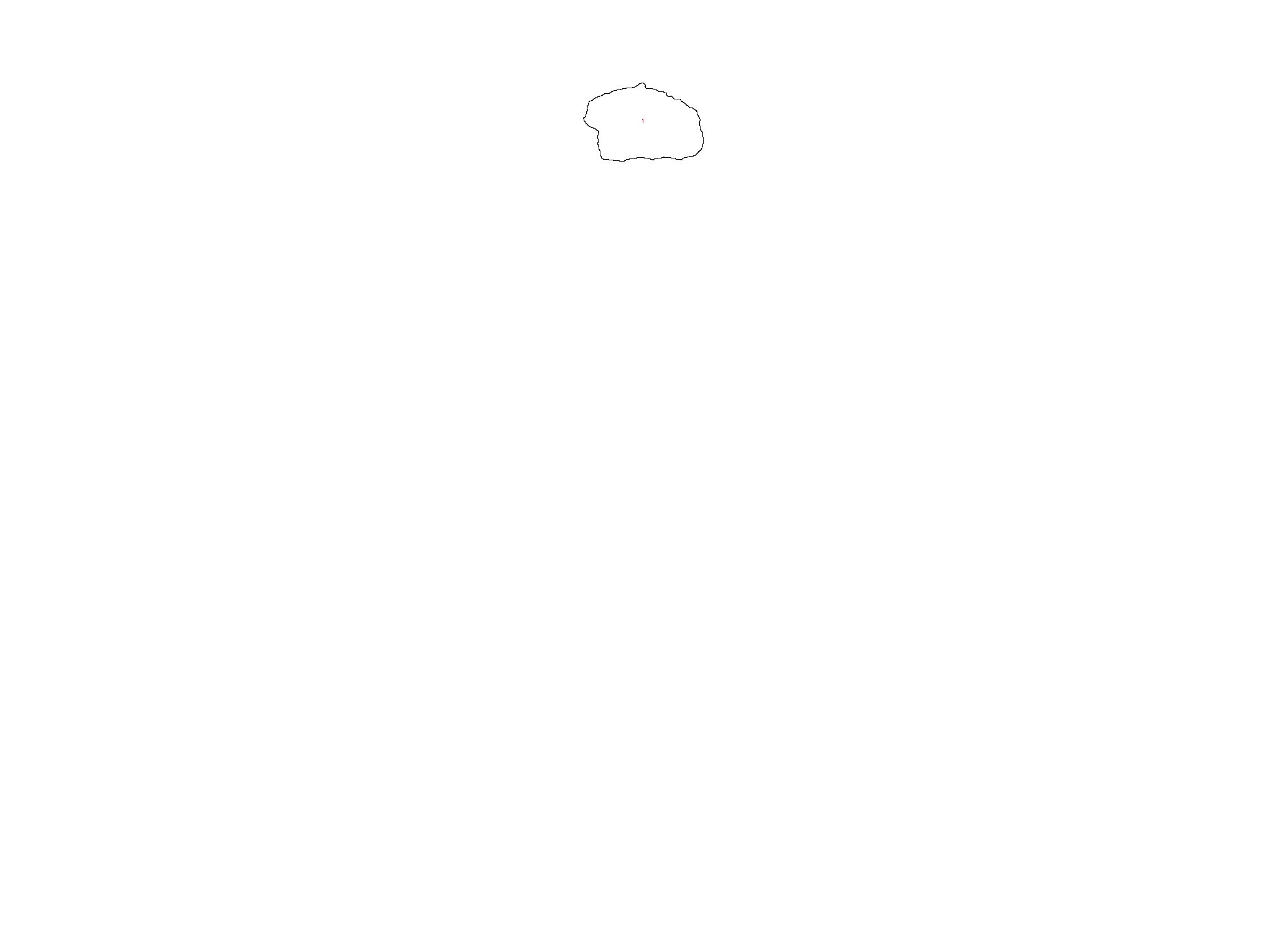

Supplement: S2 Dataset — (ZIP) [file pone.0304198.s005.zip › S2_Dataset_Raw_results_ImageJ/J2_100F_8090_4.jpg]

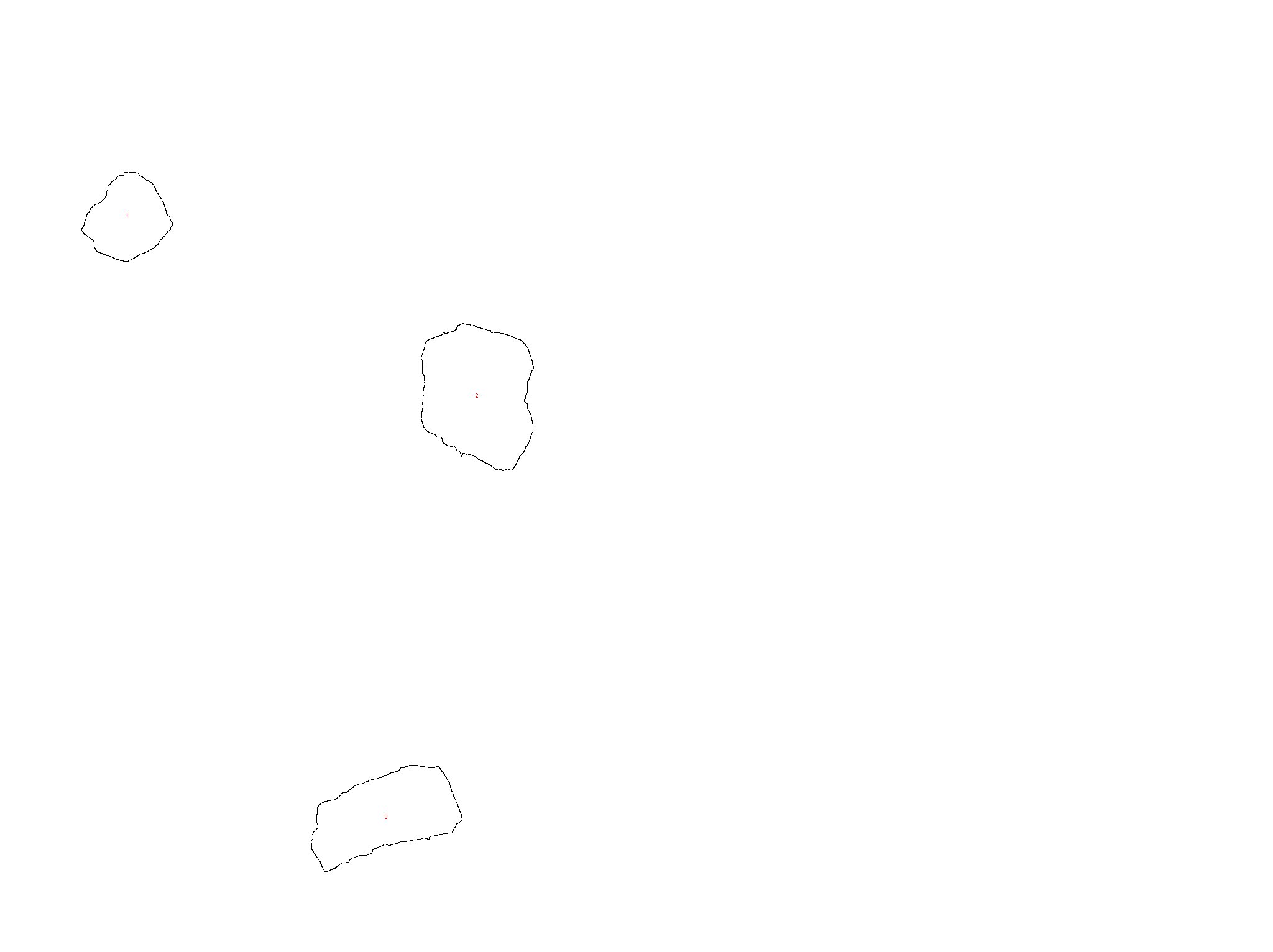

Supplement: S2 Dataset — (ZIP) [file pone.0304198.s005.zip › S2_Dataset_Raw_results_ImageJ/J2_100F_8090_5.jpg]

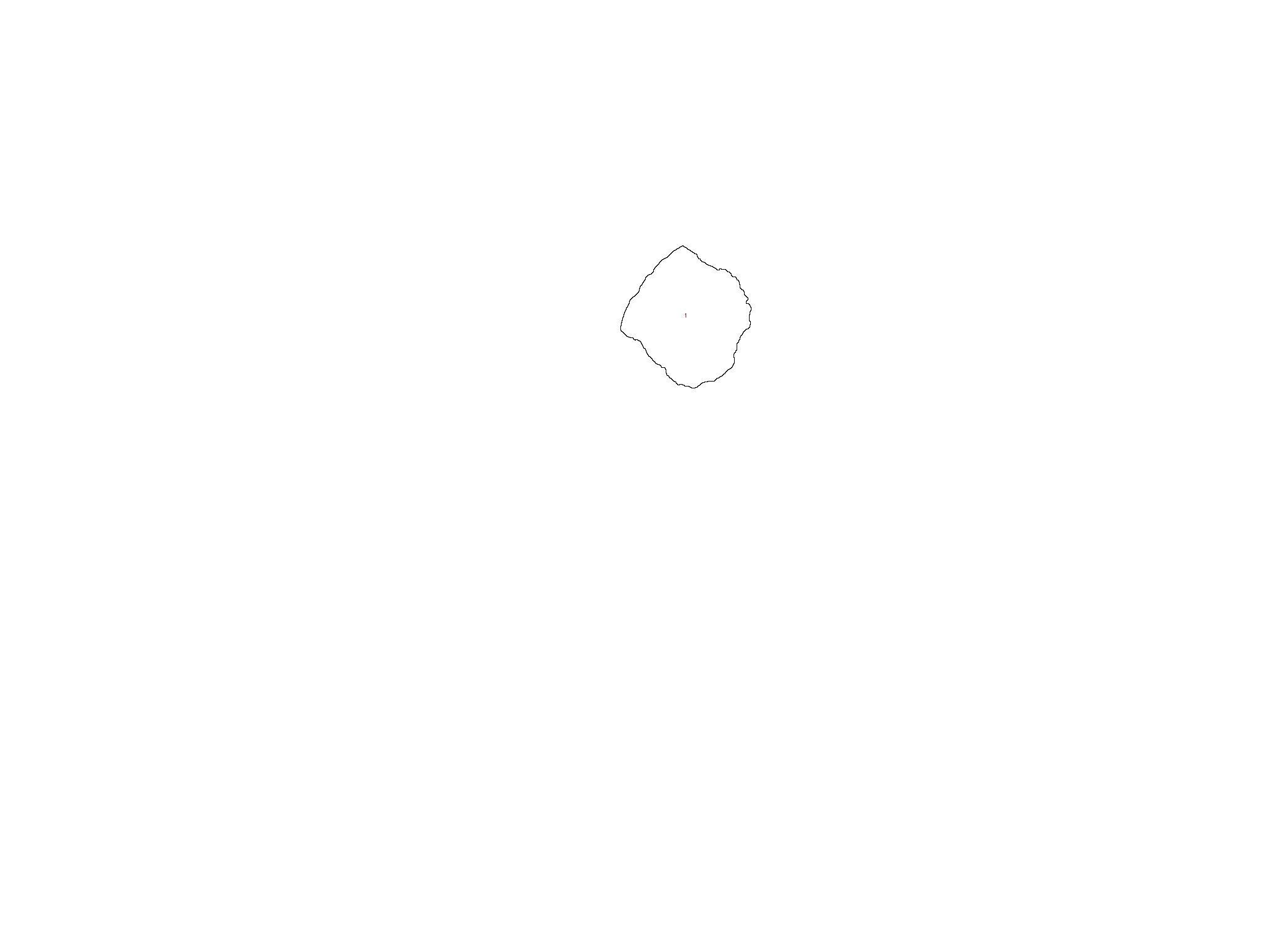

Supplement: S2 Dataset — (ZIP) [file pone.0304198.s005.zip › S2_Dataset_Raw_results_ImageJ/J2_100F_8090_6.jpg]

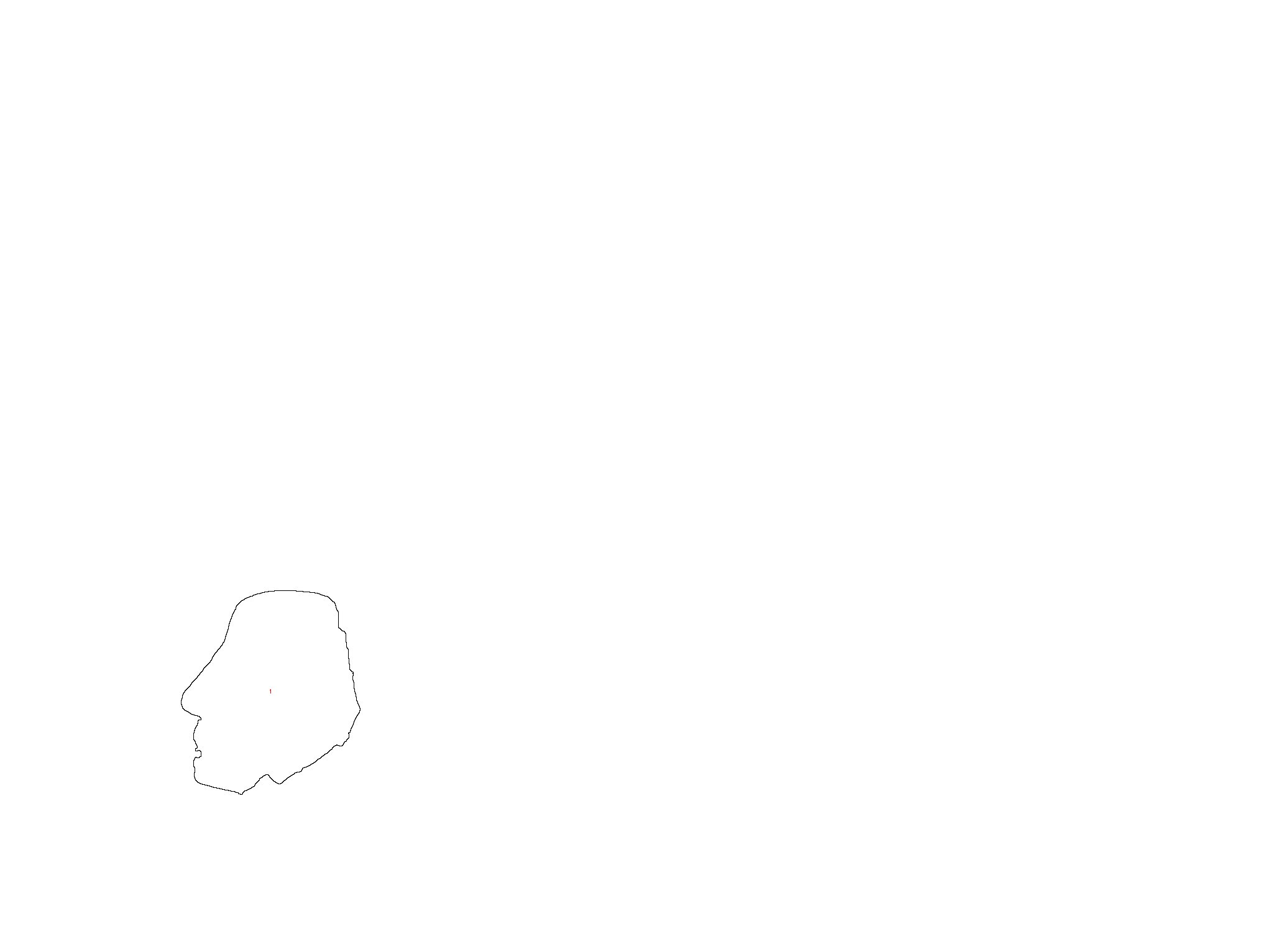

Supplement: S2 Dataset — (ZIP) [file pone.0304198.s005.zip › S2_Dataset_Raw_results_ImageJ/J2_100F_8090_7.jpg]

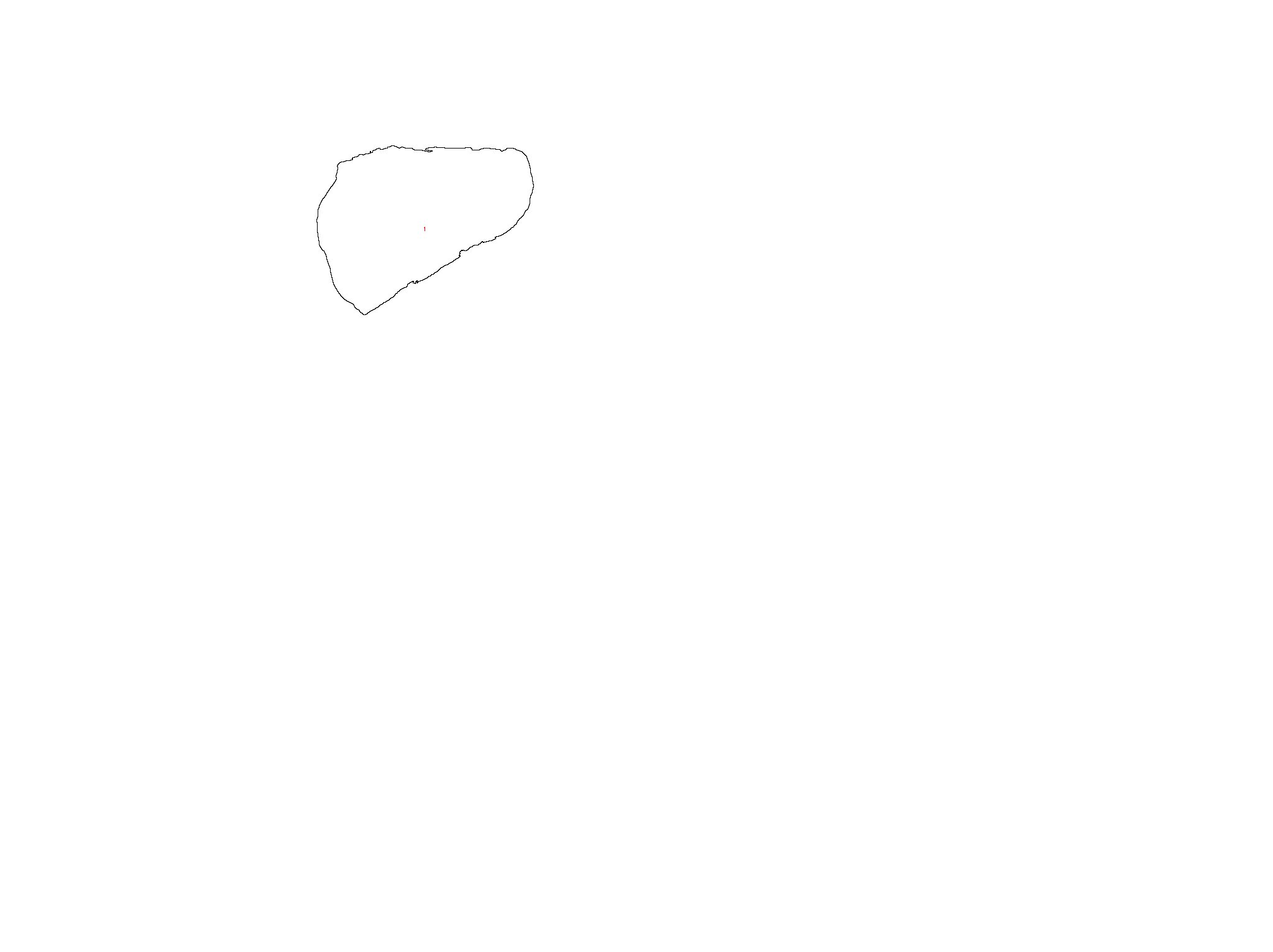

Supplement: S2 Dataset — (ZIP) [file pone.0304198.s005.zip › S2_Dataset_Raw_results_ImageJ/J2_100F_8090_8.jpg]

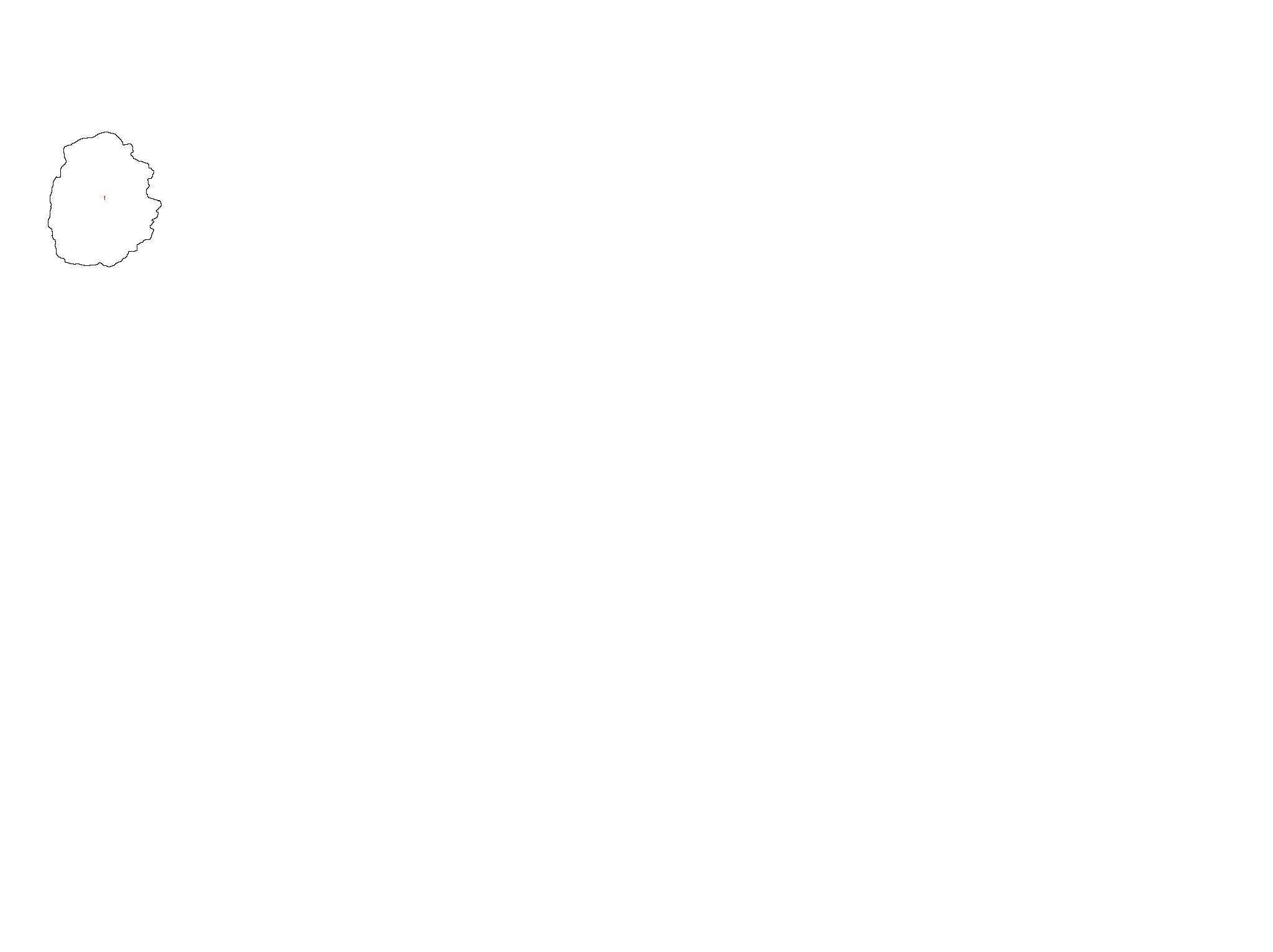

Supplement: S2 Dataset — (ZIP) [file pone.0304198.s005.zip › S2_Dataset_Raw_results_ImageJ/J2_100F_8090_9.jpg]

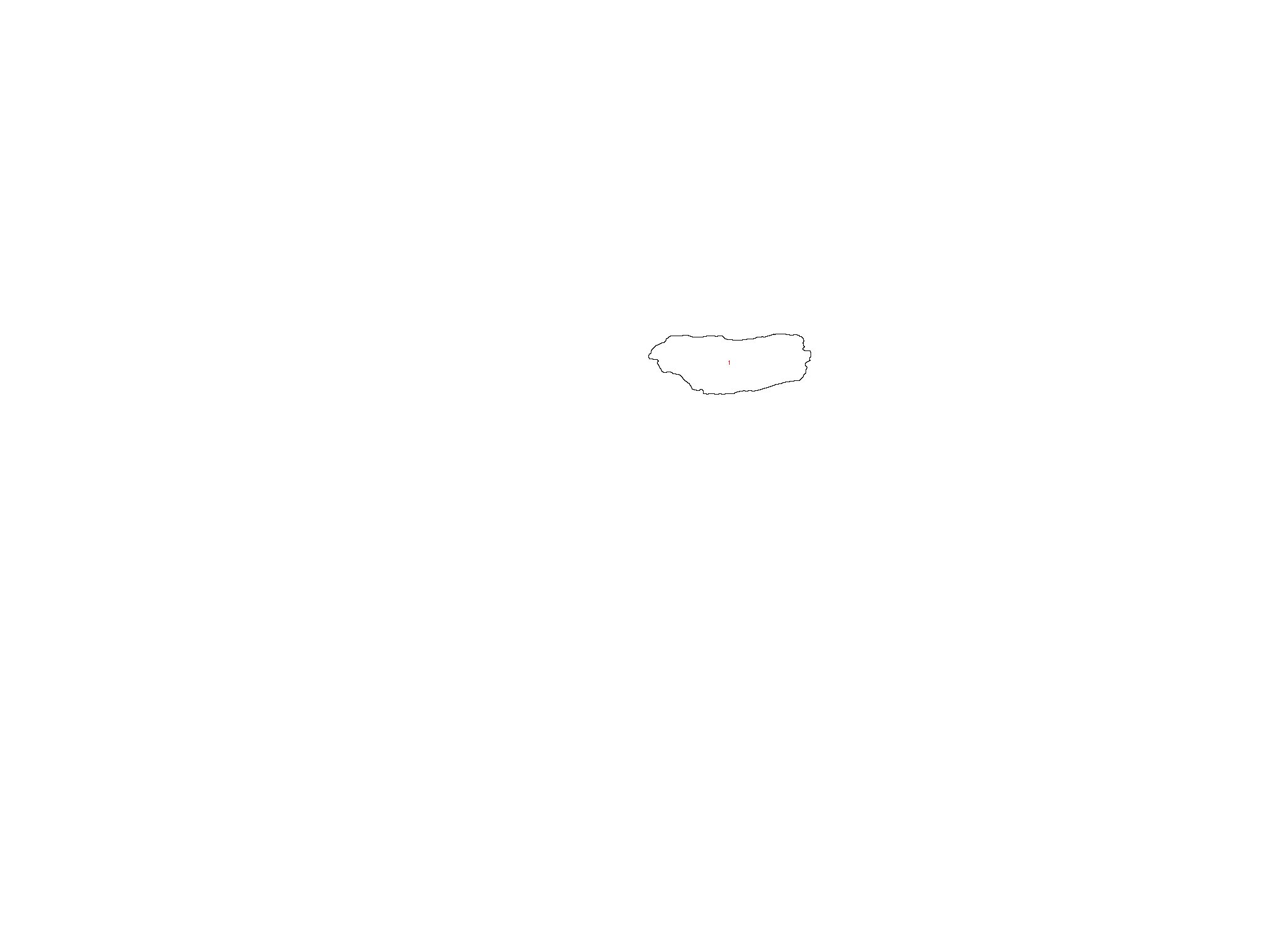

Supplement: S2 Dataset — (ZIP) [file pone.0304198.s005.zip › S2_Dataset_Raw_results_ImageJ/J2_100S_170180_1.jpg]

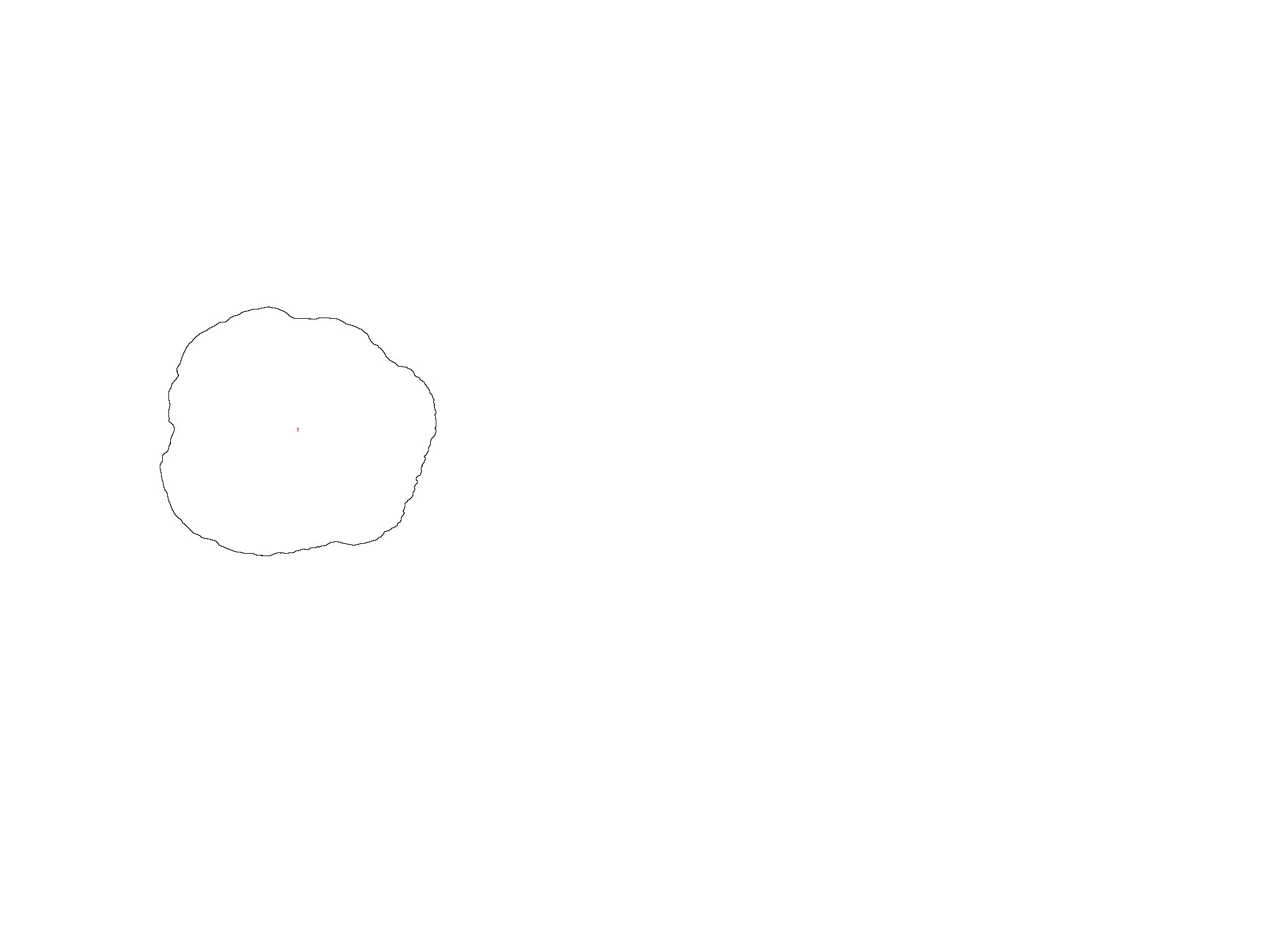

Supplement: S2 Dataset — (ZIP) [file pone.0304198.s005.zip › S2_Dataset_Raw_results_ImageJ/J2_100S_170180_2.jpg]

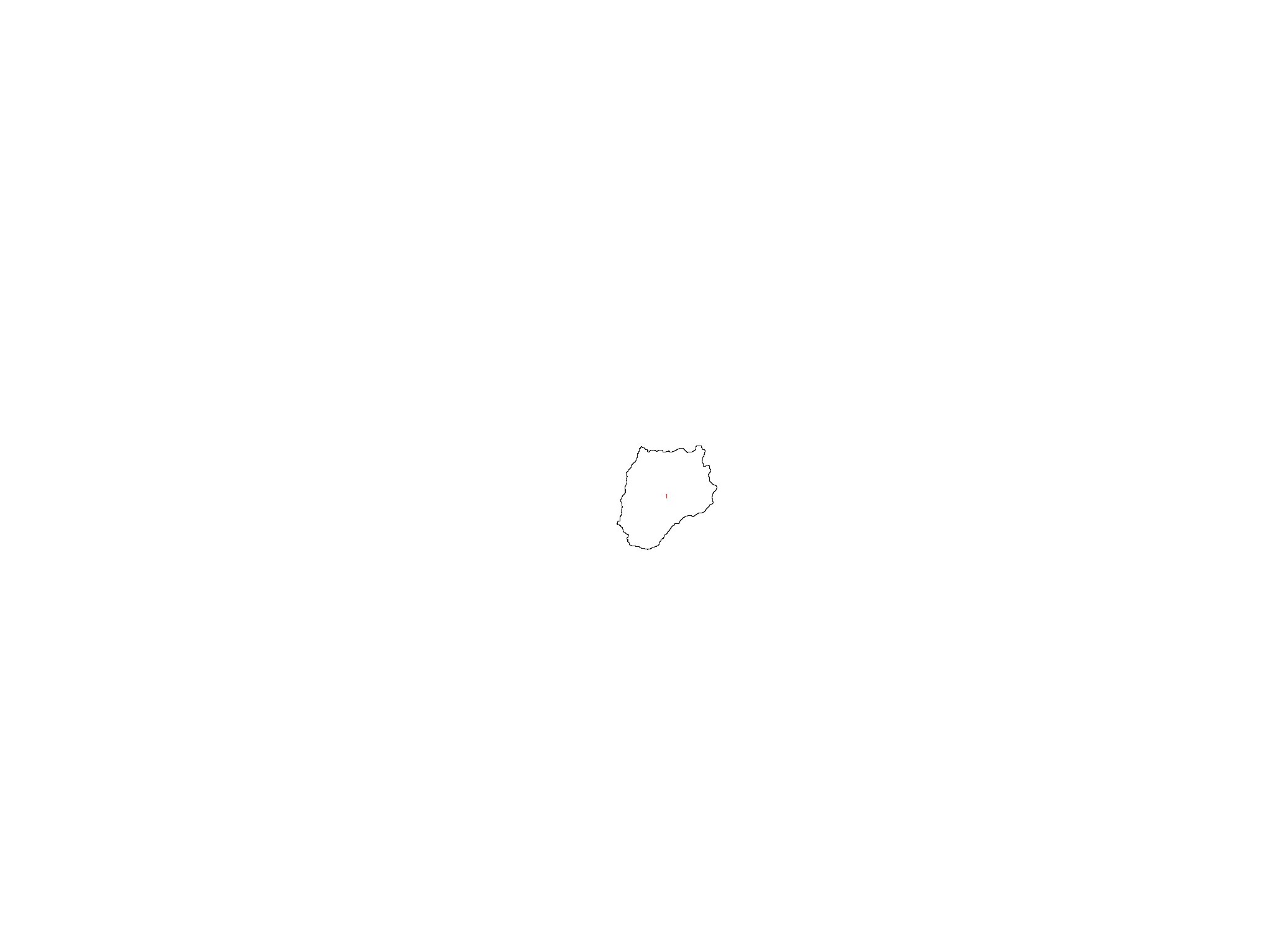

Supplement: S2 Dataset — (ZIP) [file pone.0304198.s005.zip › S2_Dataset_Raw_results_ImageJ/J2_100S_5060_1.jpg]

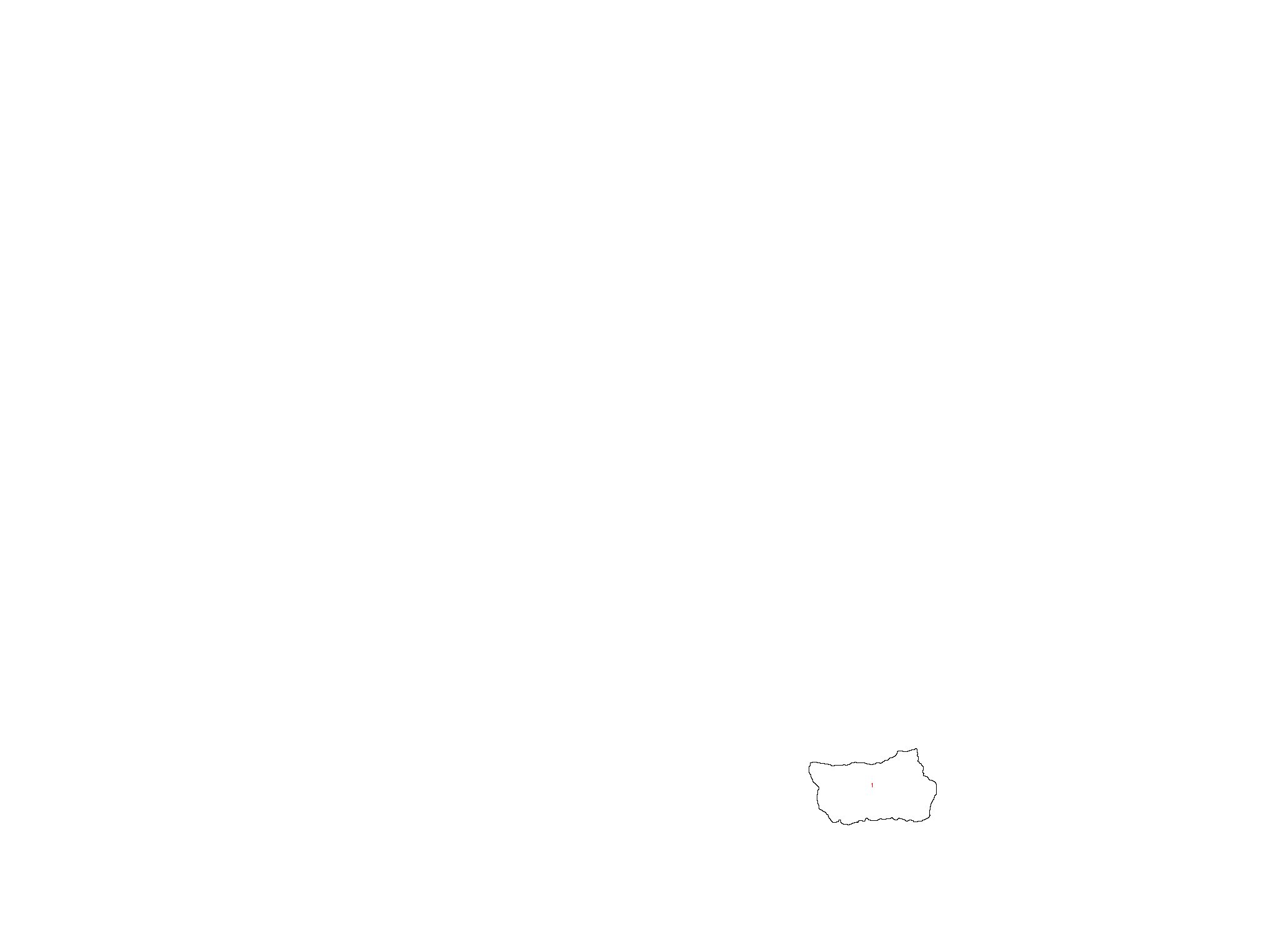

Supplement: S2 Dataset — (ZIP) [file pone.0304198.s005.zip › S2_Dataset_Raw_results_ImageJ/J2_100S_5060_2.jpg]

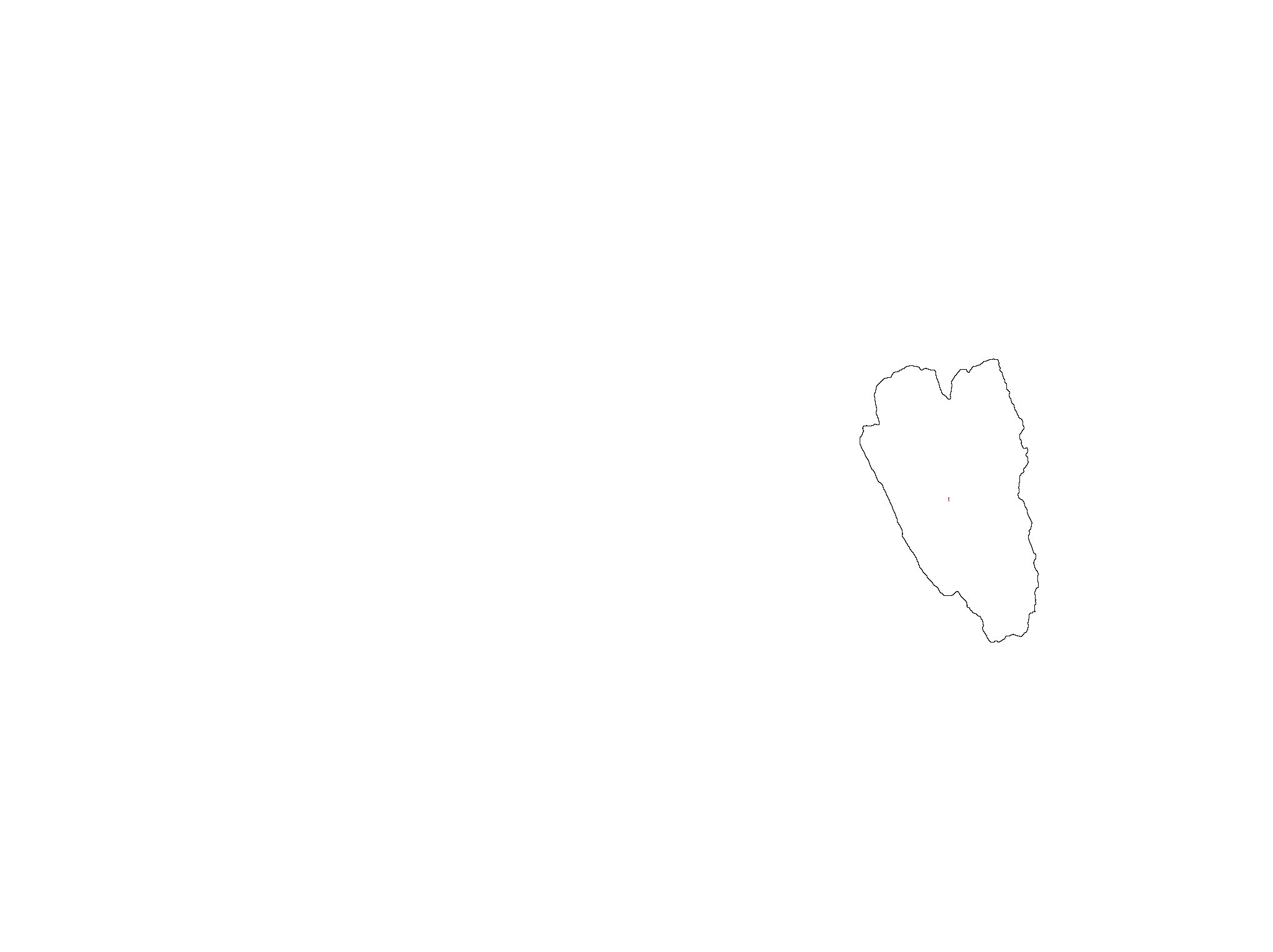

Supplement: S2 Dataset — (ZIP) [file pone.0304198.s005.zip › S2_Dataset_Raw_results_ImageJ/J2_100S_5060_3.jpg]

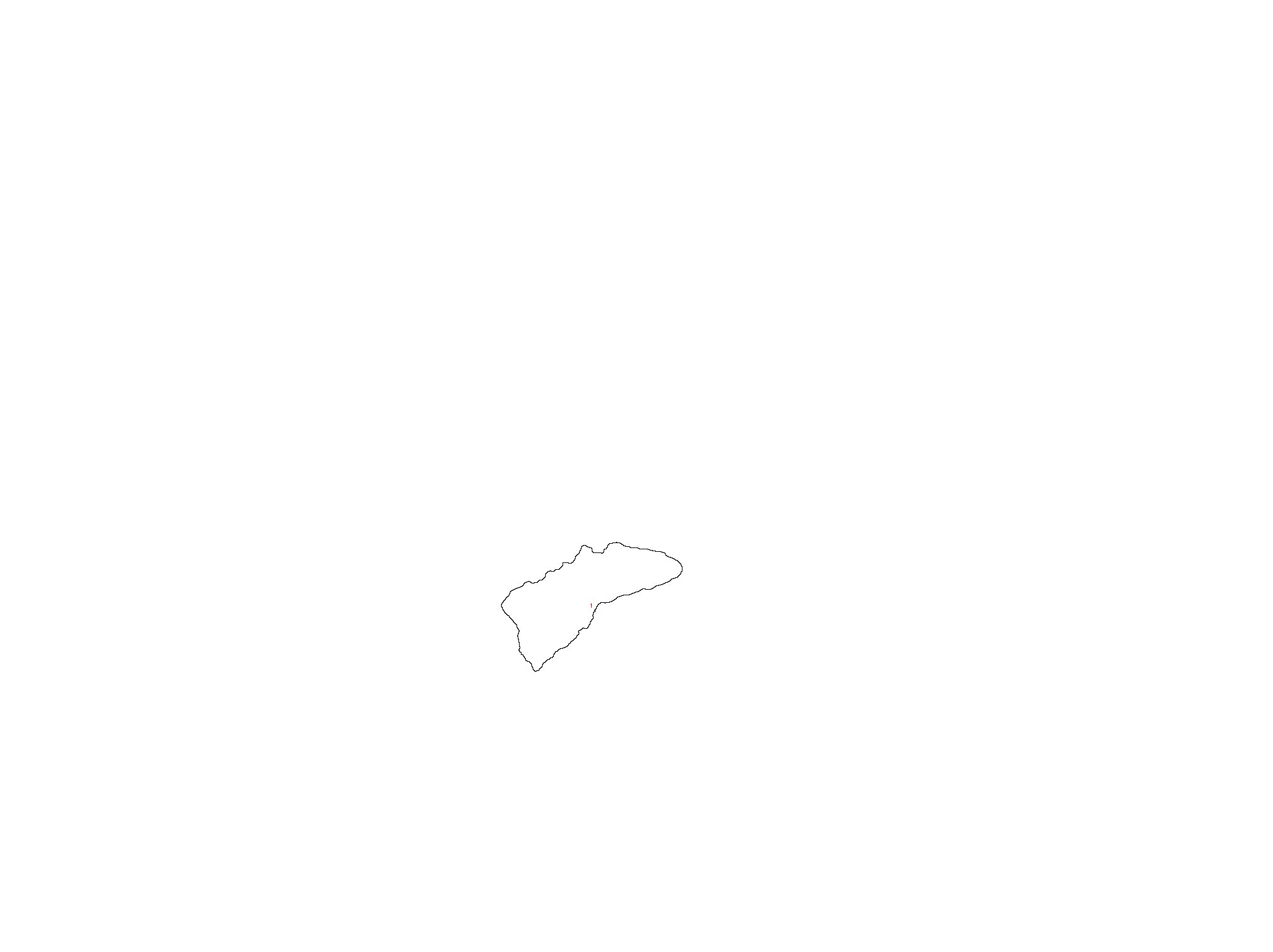

Supplement: S2 Dataset — (ZIP) [file pone.0304198.s005.zip › S2_Dataset_Raw_results_ImageJ/J2_100S_5060_4.jpg]

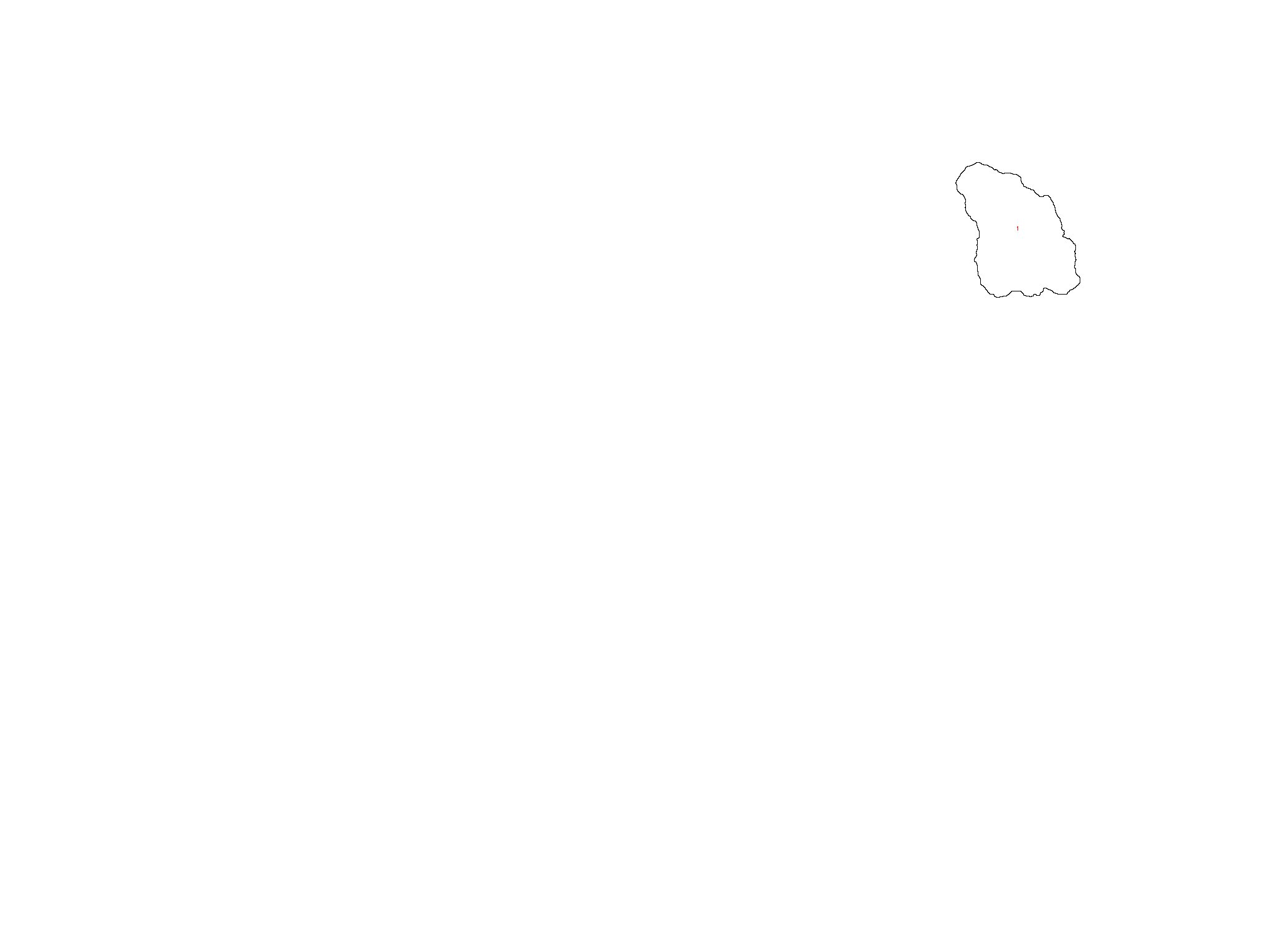

Supplement: S2 Dataset — (ZIP) [file pone.0304198.s005.zip › S2_Dataset_Raw_results_ImageJ/J2_100S_5060_5.jpg]

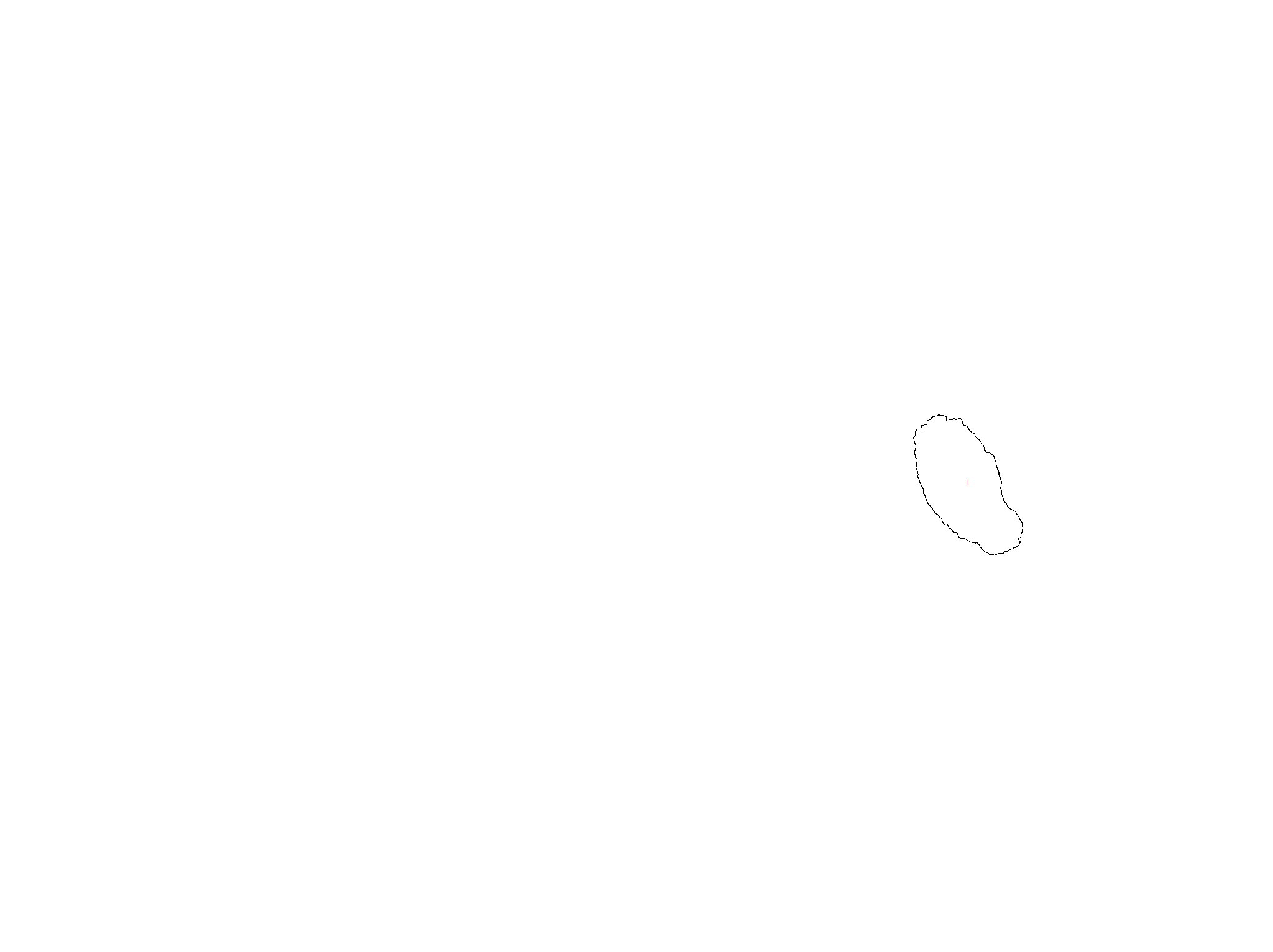

Supplement: S2 Dataset — (ZIP) [file pone.0304198.s005.zip › S2_Dataset_Raw_results_ImageJ/J2_100S_6070_1.jpg]

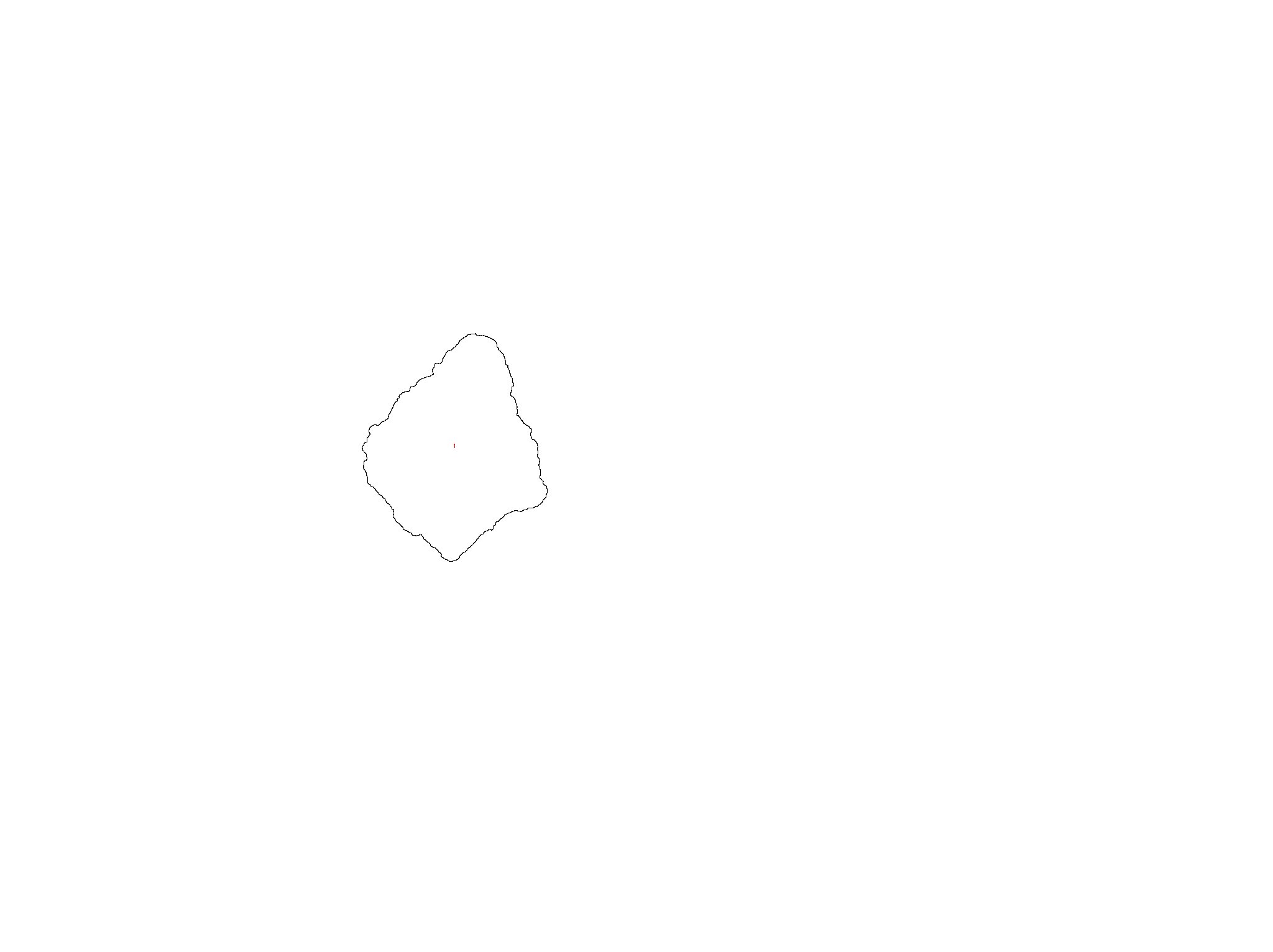

Supplement: S2 Dataset — (ZIP) [file pone.0304198.s005.zip › S2_Dataset_Raw_results_ImageJ/J2_100S_6070_2.jpg]

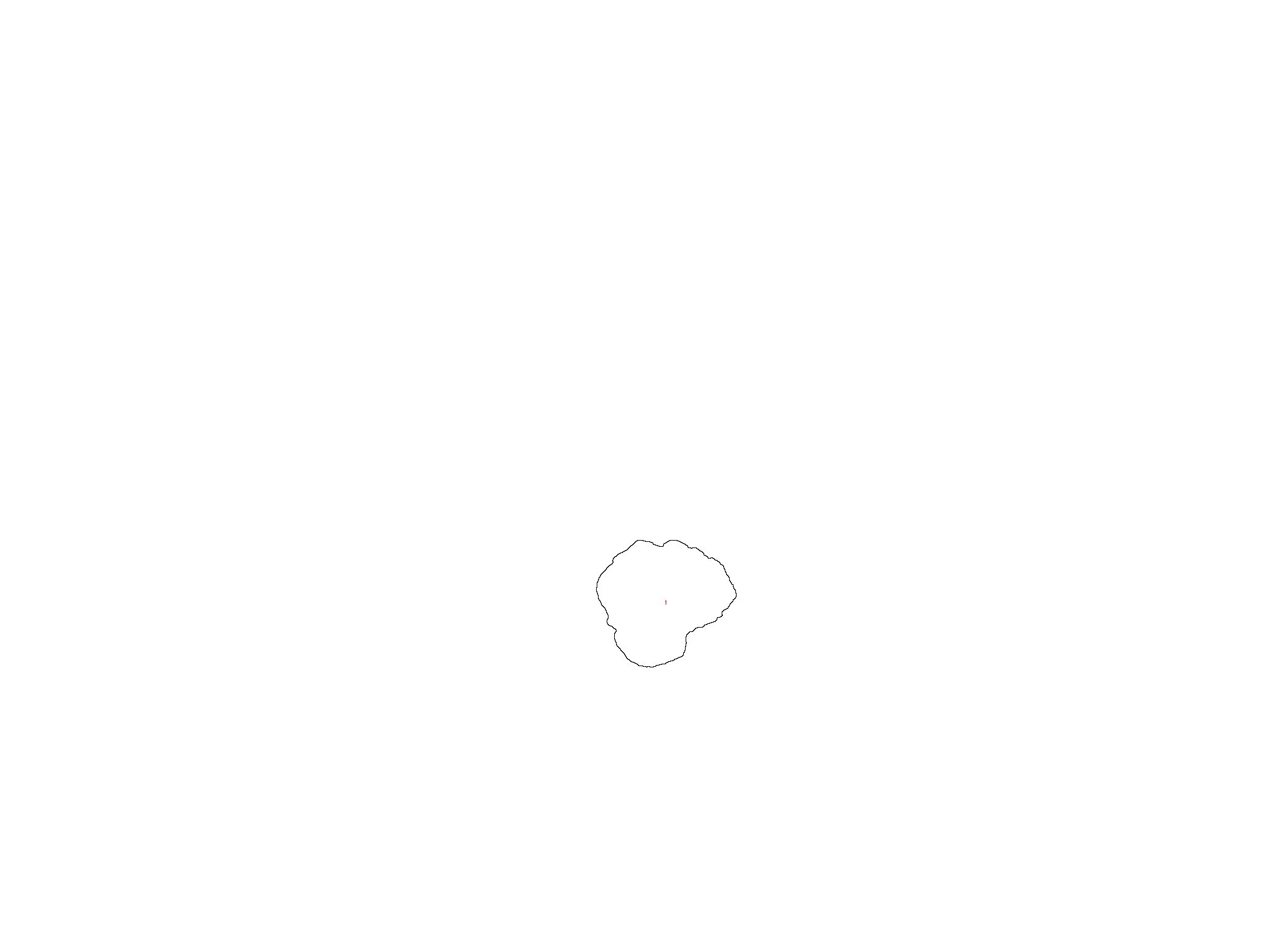

Supplement: S2 Dataset — (ZIP) [file pone.0304198.s005.zip › S2_Dataset_Raw_results_ImageJ/J2_100S_6070_3.jpg]

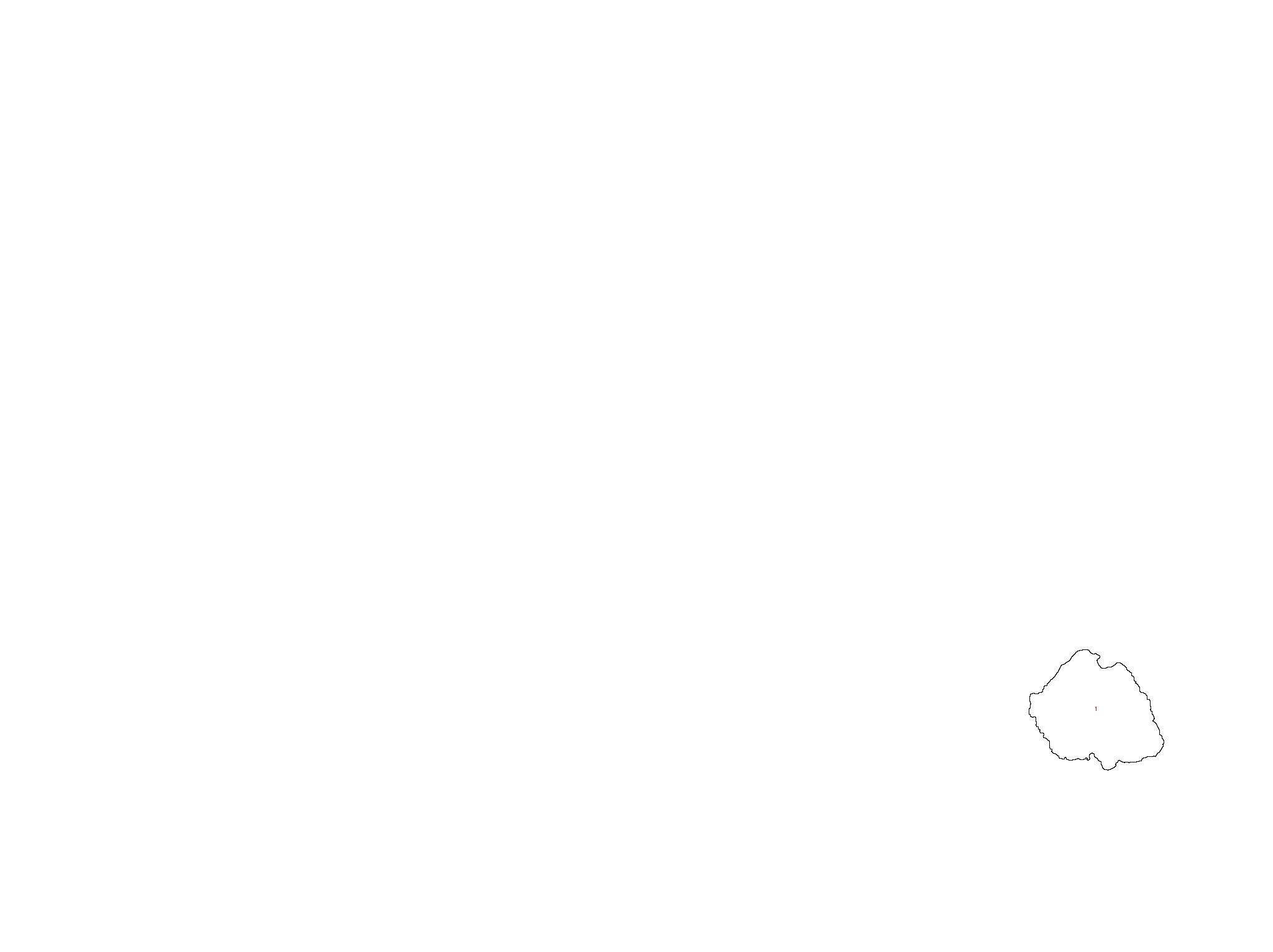

Supplement: S2 Dataset — (ZIP) [file pone.0304198.s005.zip › S2_Dataset_Raw_results_ImageJ/J2_100S_6070_4.jpg]

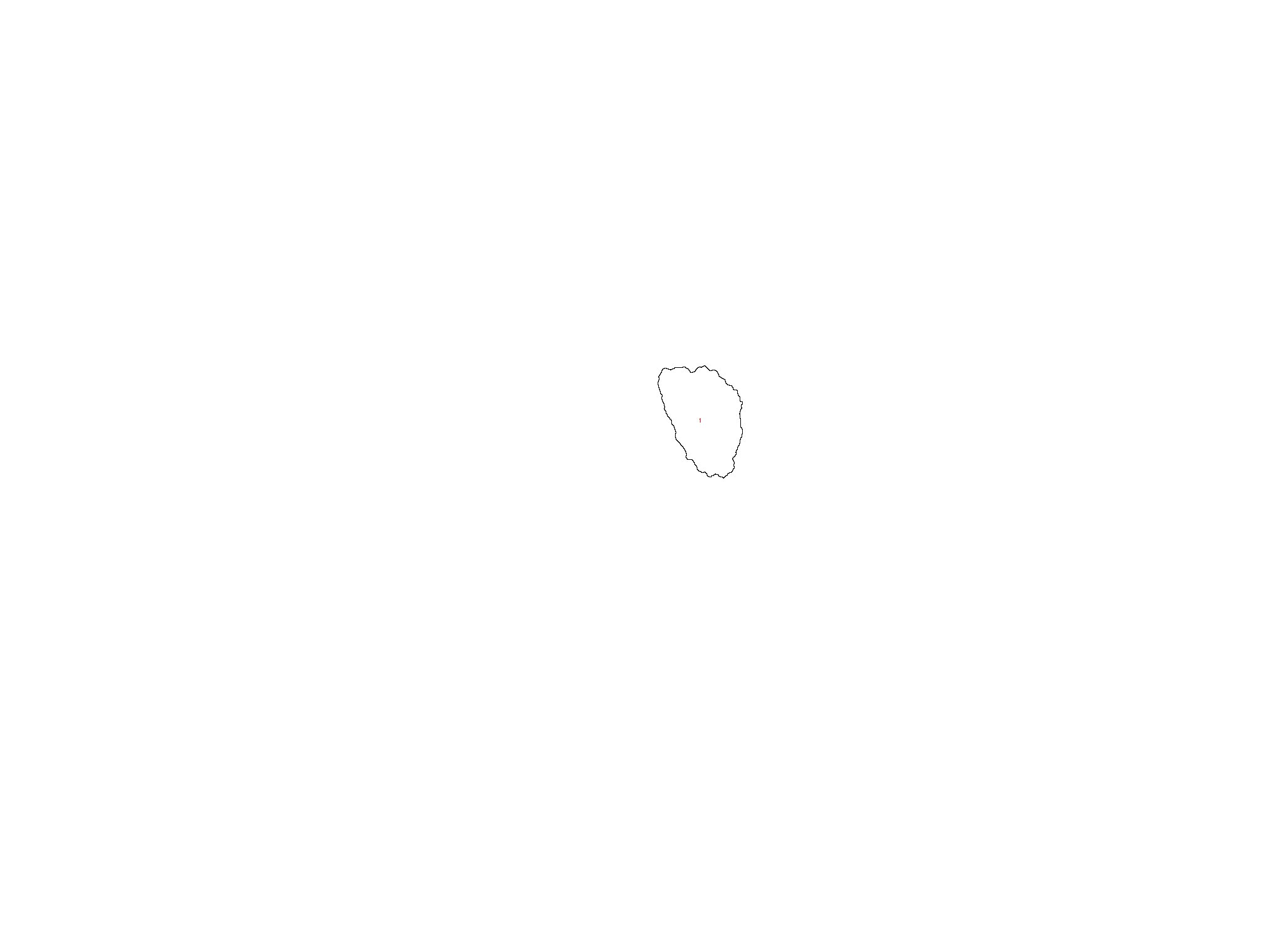

Supplement: S2 Dataset — (ZIP) [file pone.0304198.s005.zip › S2_Dataset_Raw_results_ImageJ/J2_100S_8090_1.jpg]

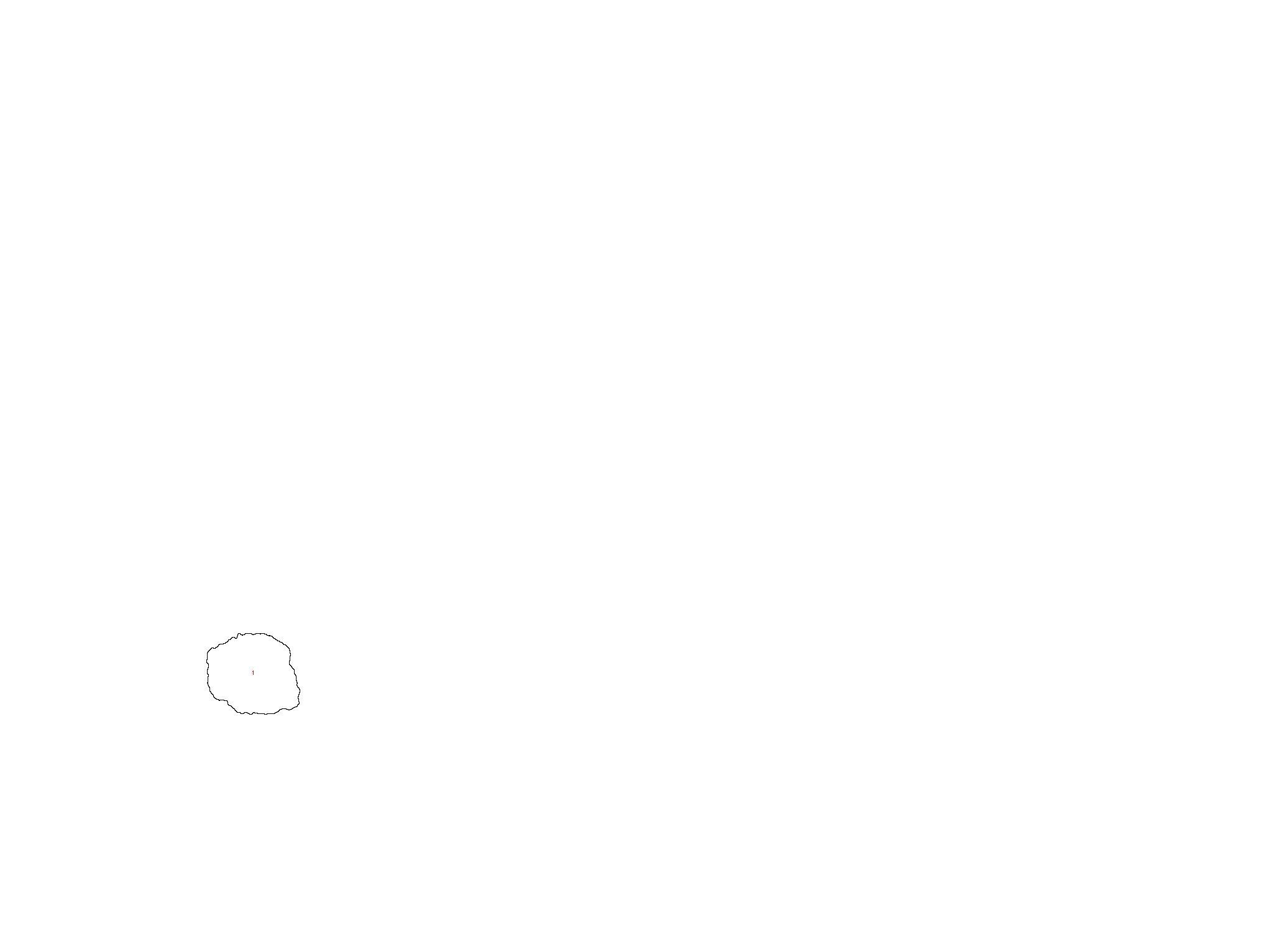

Supplement: S2 Dataset — (ZIP) [file pone.0304198.s005.zip › S2_Dataset_Raw_results_ImageJ/J2_100S_8090_2.jpg]

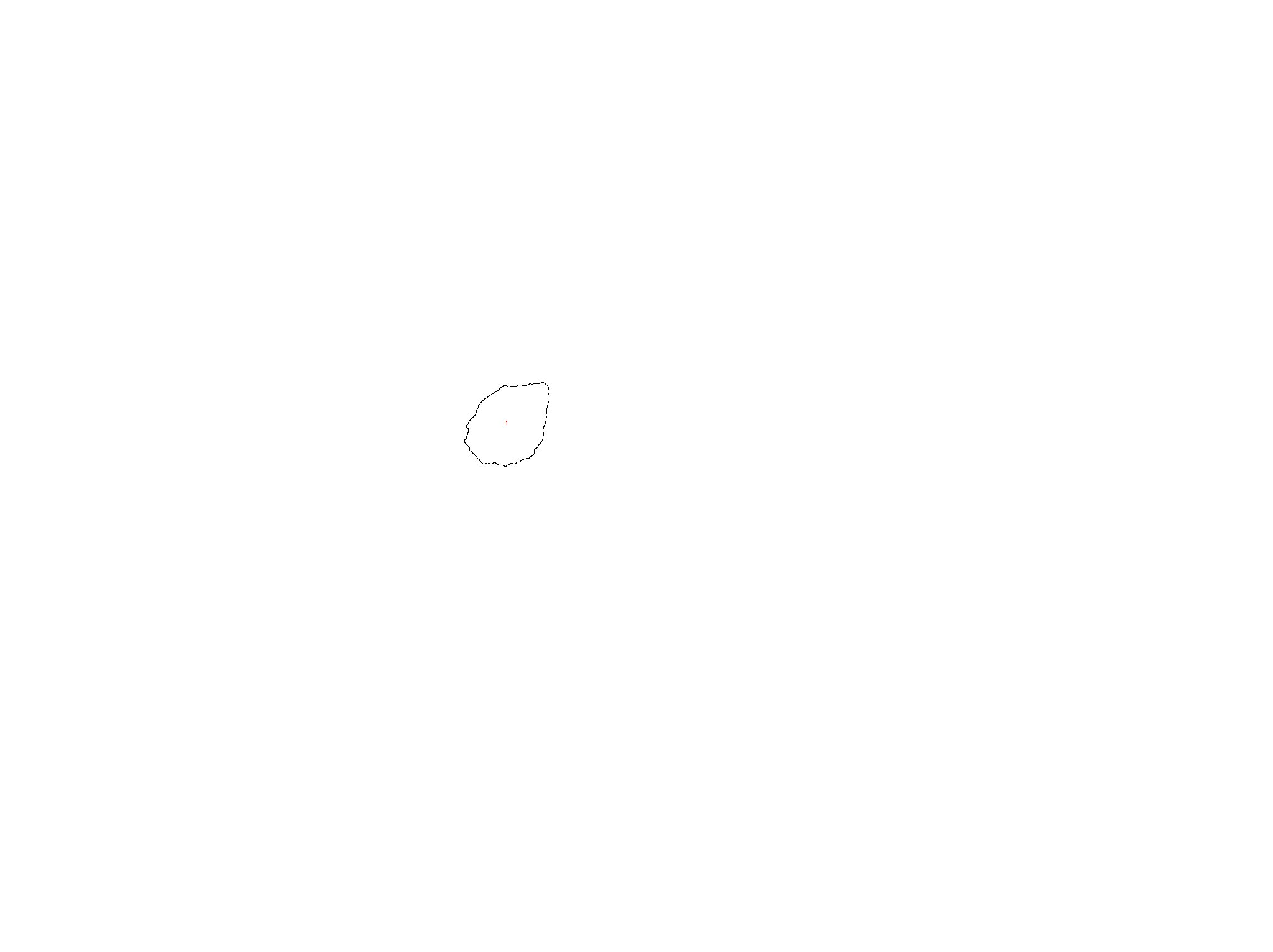

Supplement: S2 Dataset — (ZIP) [file pone.0304198.s005.zip › S2_Dataset_Raw_results_ImageJ/J2_100S_8090_3.jpg]

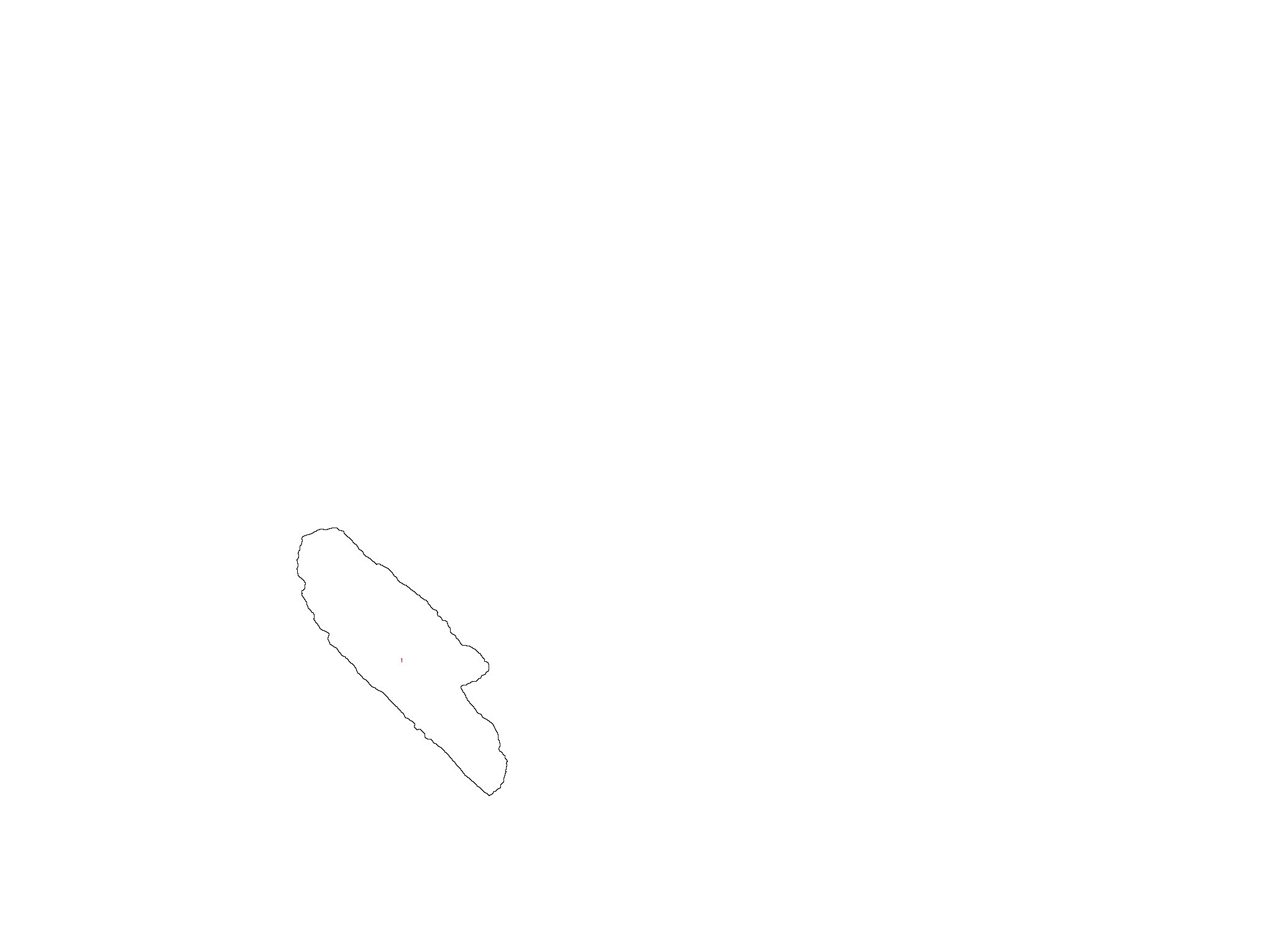

Supplement: S2 Dataset — (ZIP) [file pone.0304198.s005.zip › S2_Dataset_Raw_results_ImageJ/J2_100S_8090_4.jpg]

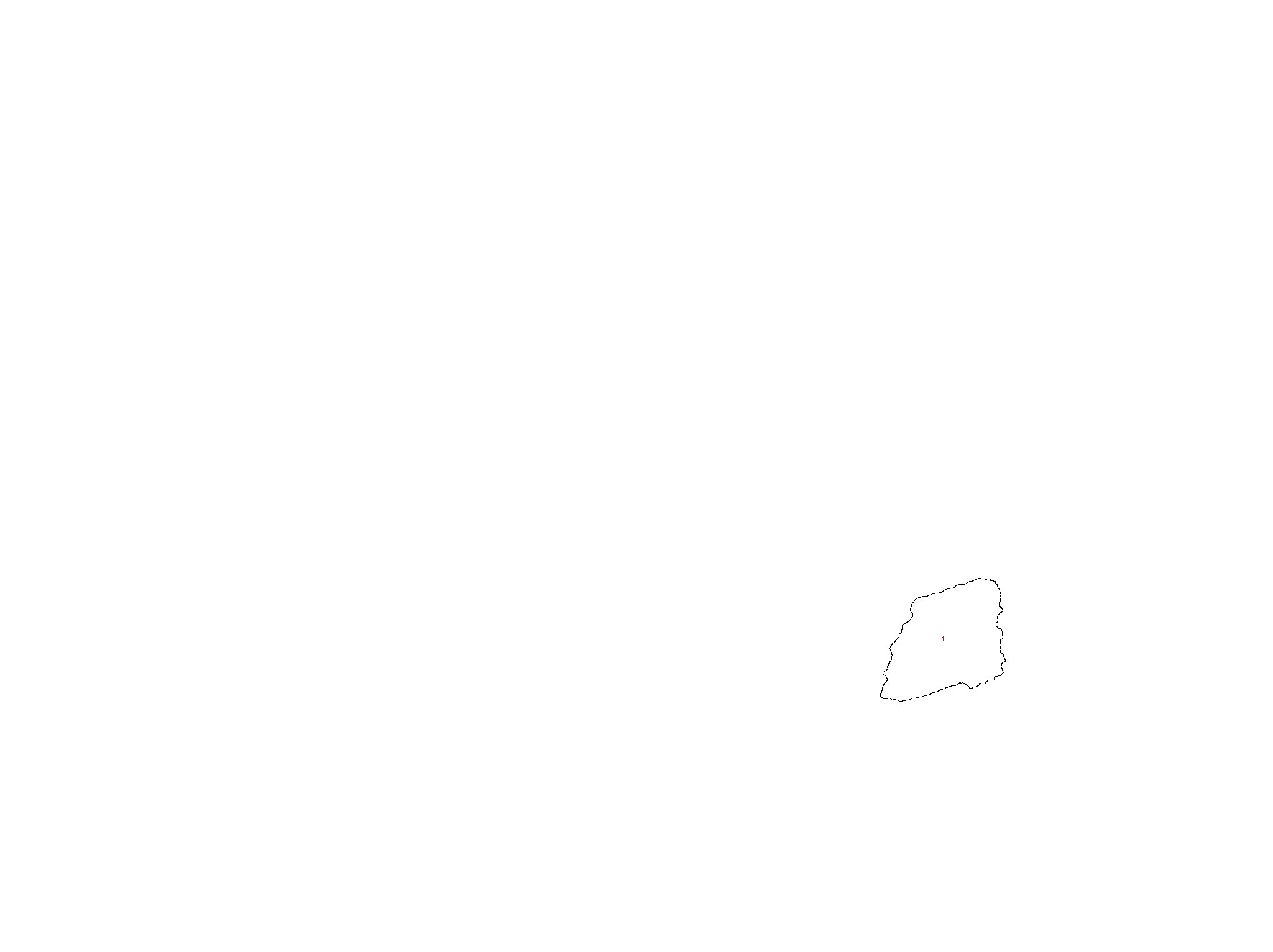

Supplement: S2 Dataset — (ZIP) [file pone.0304198.s005.zip › S2_Dataset_Raw_results_ImageJ/J2_100S_8090_5.jpg]

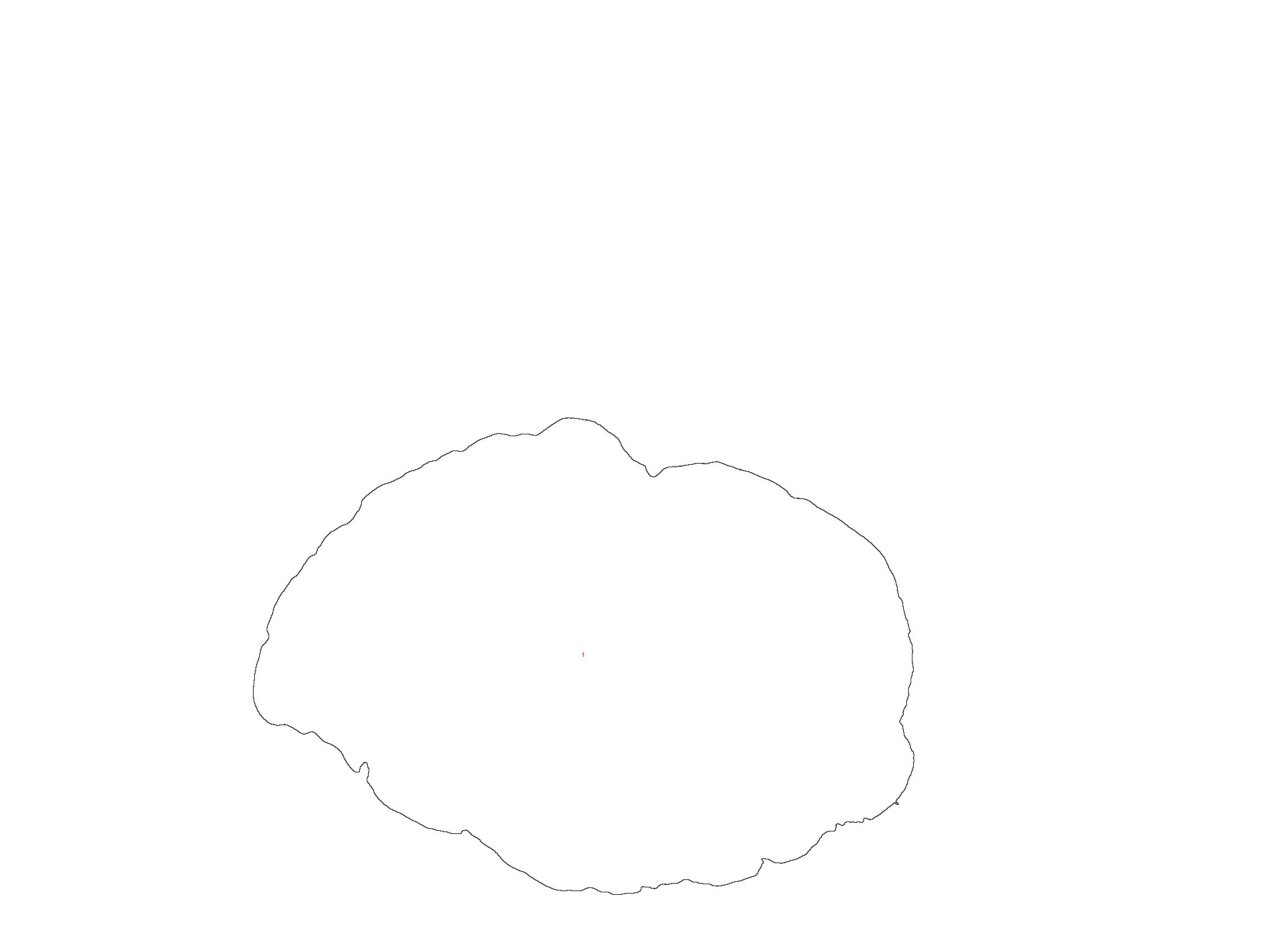

Supplement: S2 Dataset — (ZIP) [file pone.0304198.s005.zip › S2_Dataset_Raw_results_ImageJ/J2_200S_140150_1.jpg]

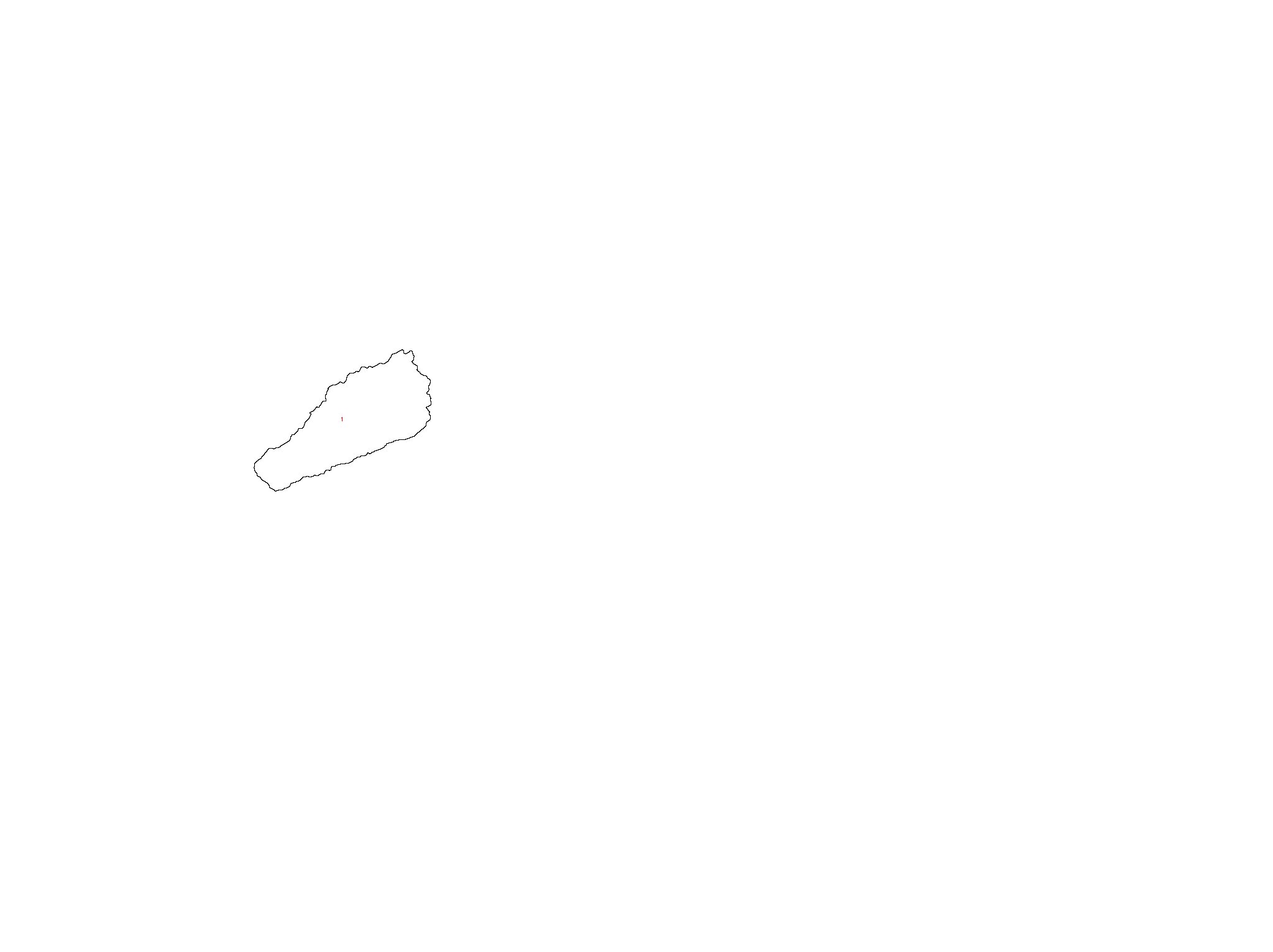

Supplement: S2 Dataset — (ZIP) [file pone.0304198.s005.zip › S2_Dataset_Raw_results_ImageJ/J2_200S_140150_2.jpg]

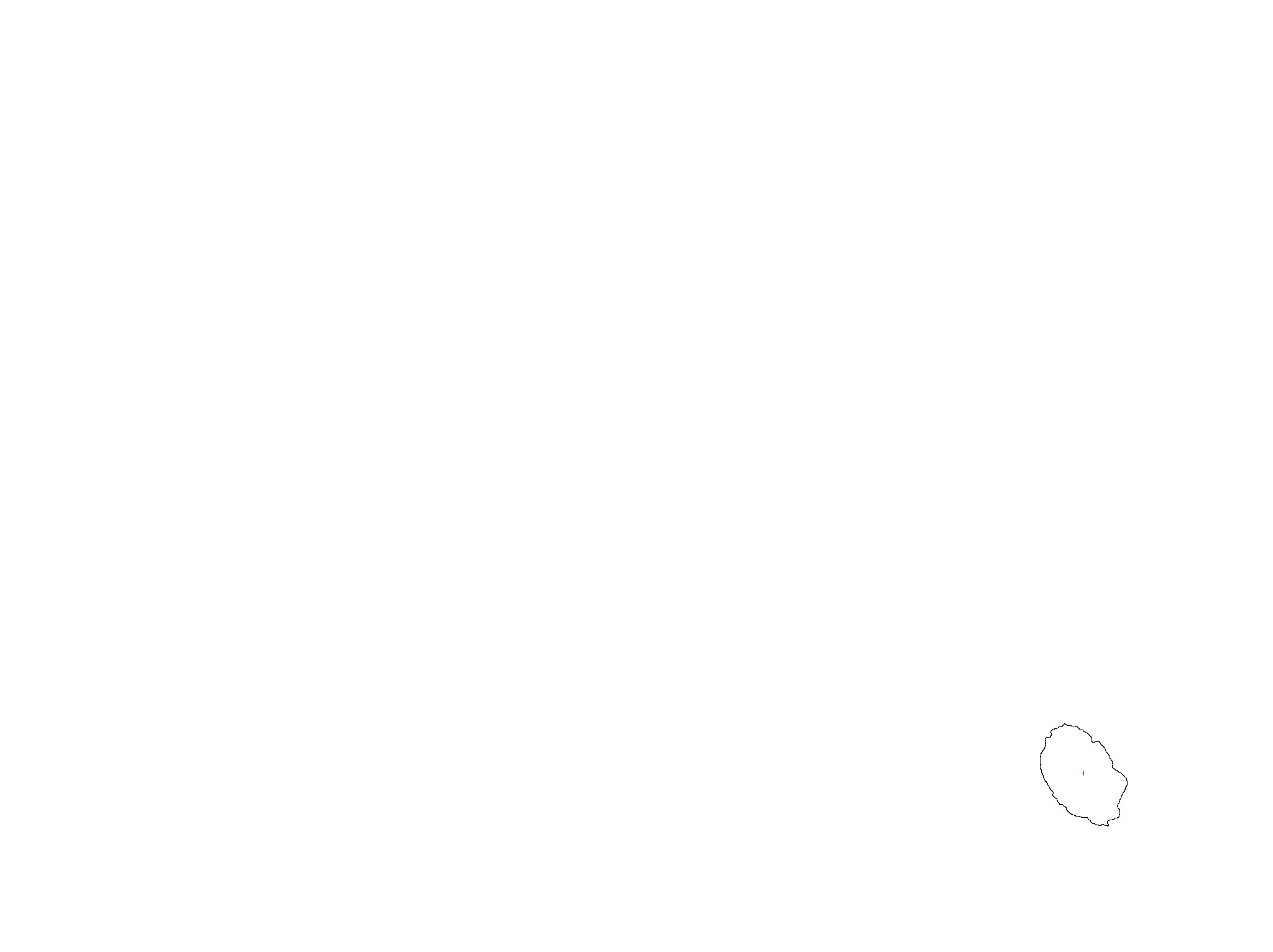

Supplement: S2 Dataset — (ZIP) [file pone.0304198.s005.zip › S2_Dataset_Raw_results_ImageJ/J2_200S_150160_1.jpg]

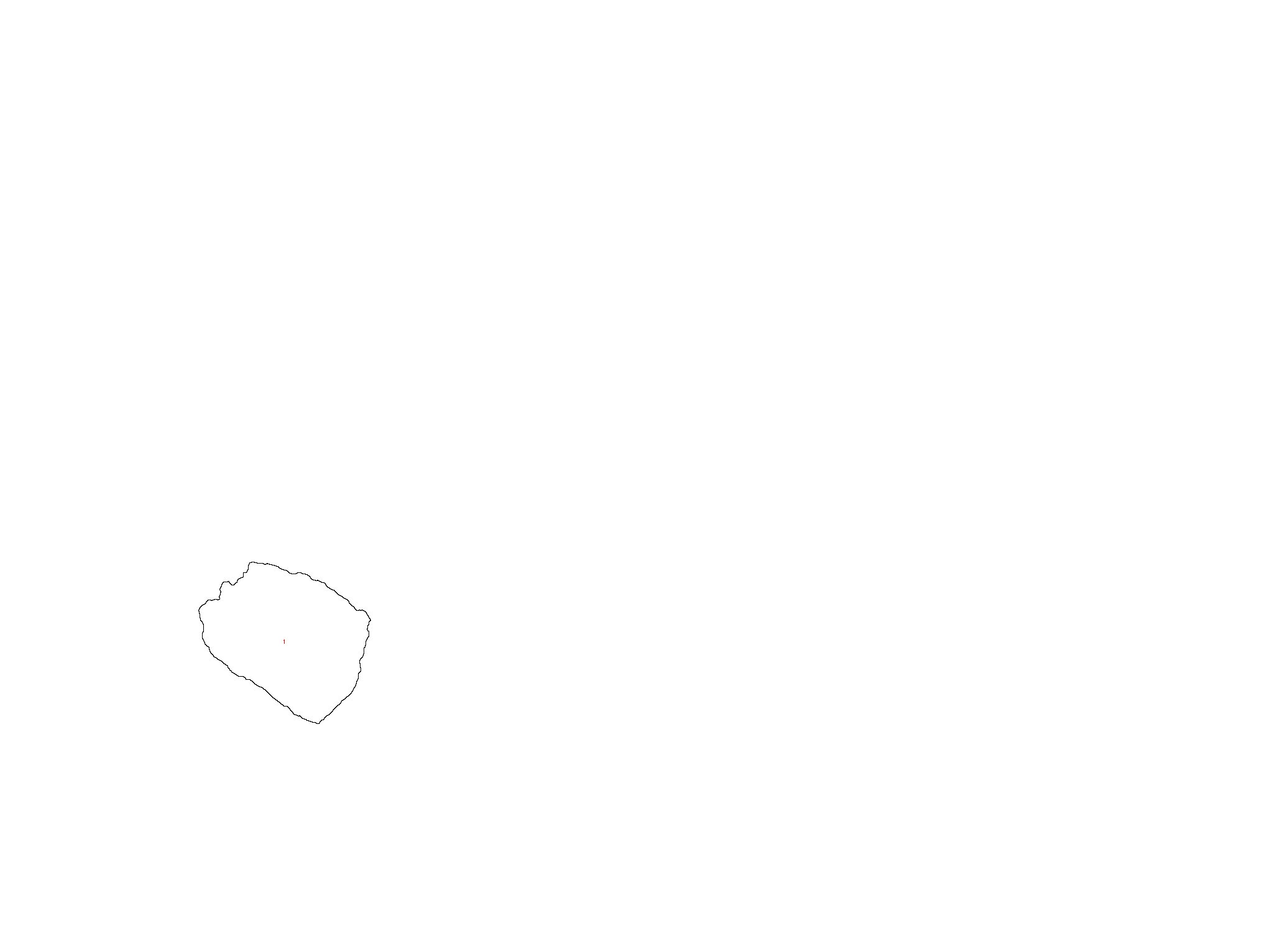

Supplement: S2 Dataset — (ZIP) [file pone.0304198.s005.zip › S2_Dataset_Raw_results_ImageJ/J2_200S_150160_2.jpg]

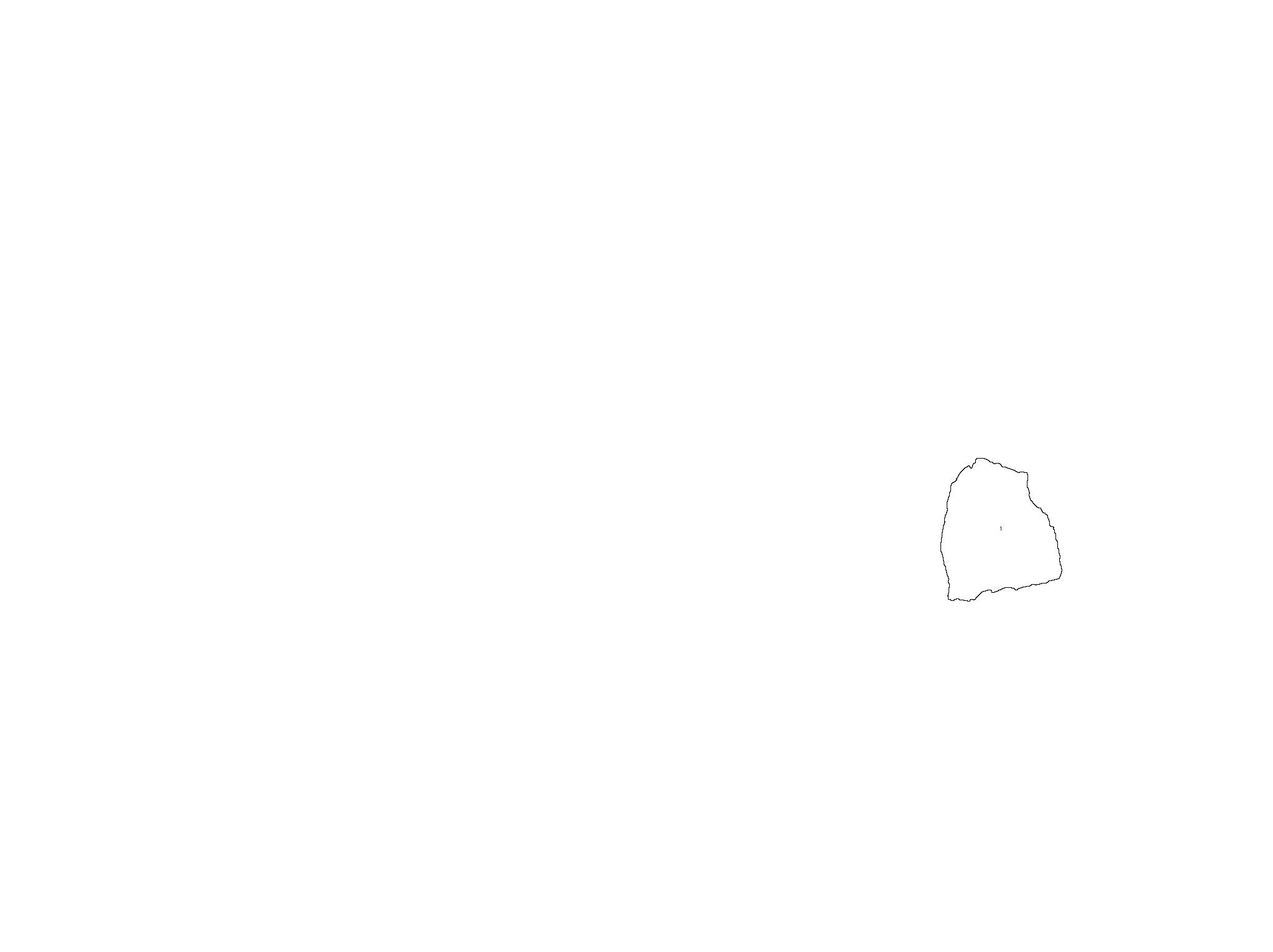

Supplement: S2 Dataset — (ZIP) [file pone.0304198.s005.zip › S2_Dataset_Raw_results_ImageJ/J2_200S_150160_3.jpg]

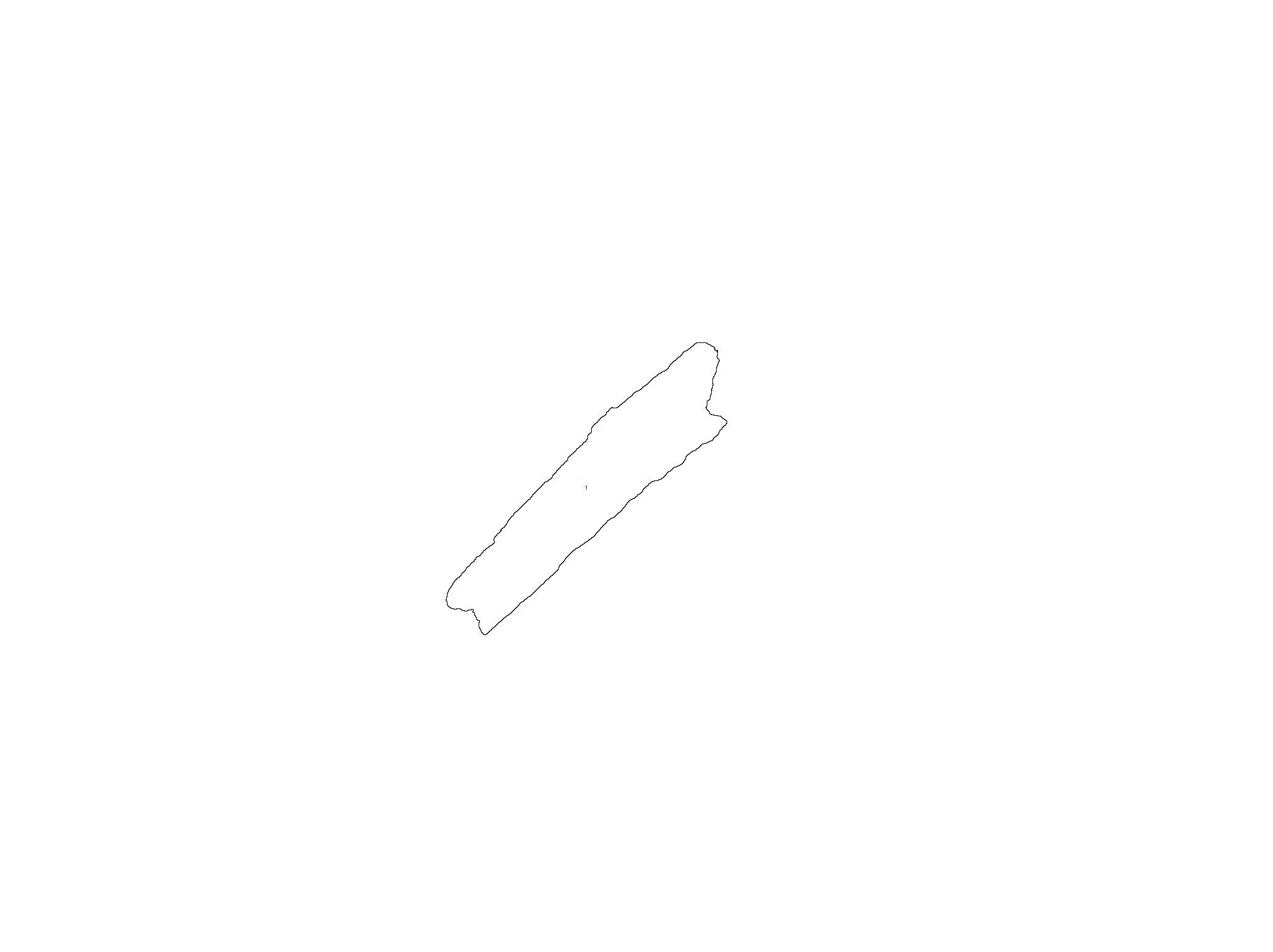

Supplement: S2 Dataset — (ZIP) [file pone.0304198.s005.zip › S2_Dataset_Raw_results_ImageJ/J2_200S_3040_1.jpg]

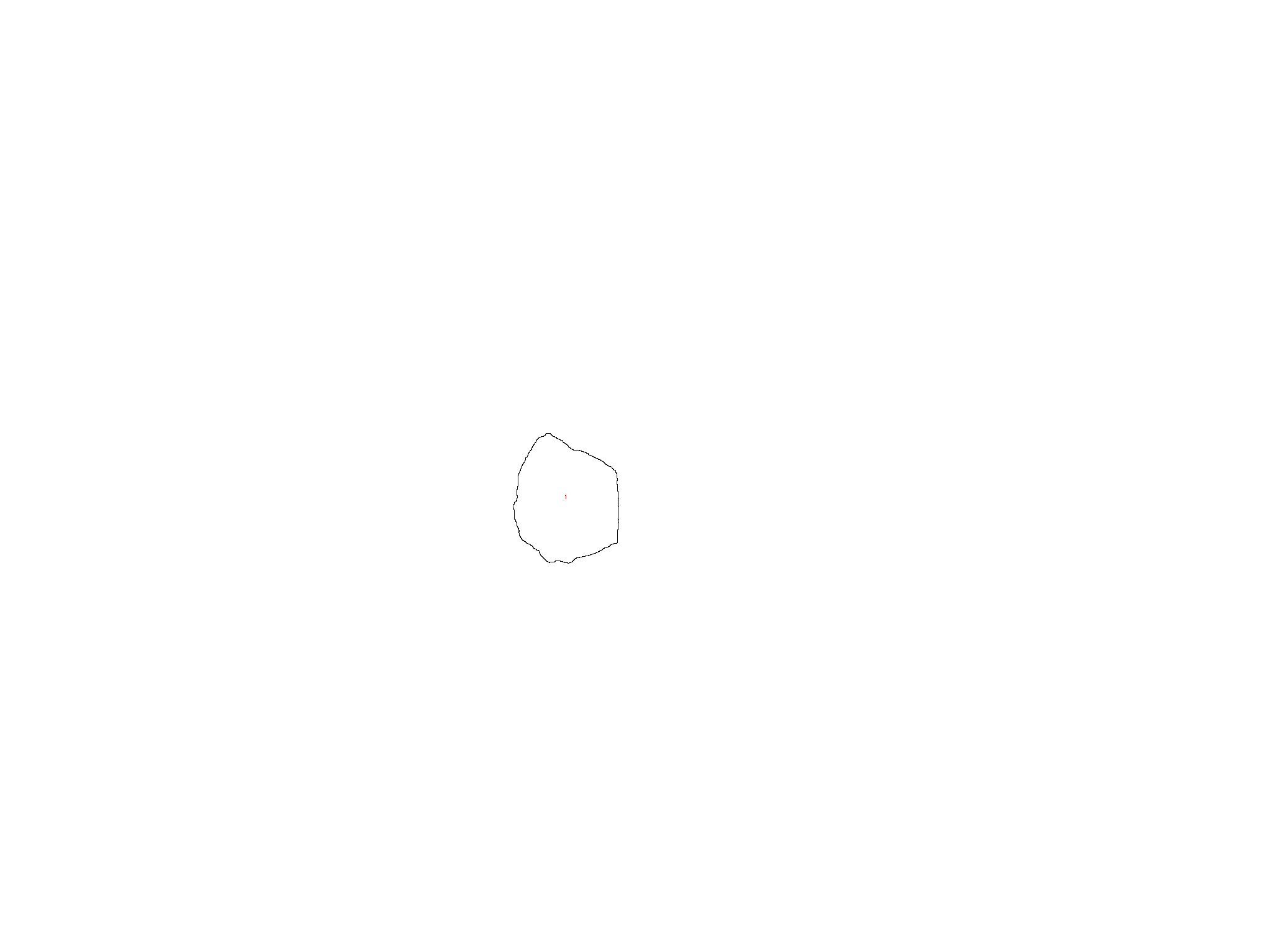

Supplement: S2 Dataset — (ZIP) [file pone.0304198.s005.zip › S2_Dataset_Raw_results_ImageJ/J2_200S_3040_10.jpg]

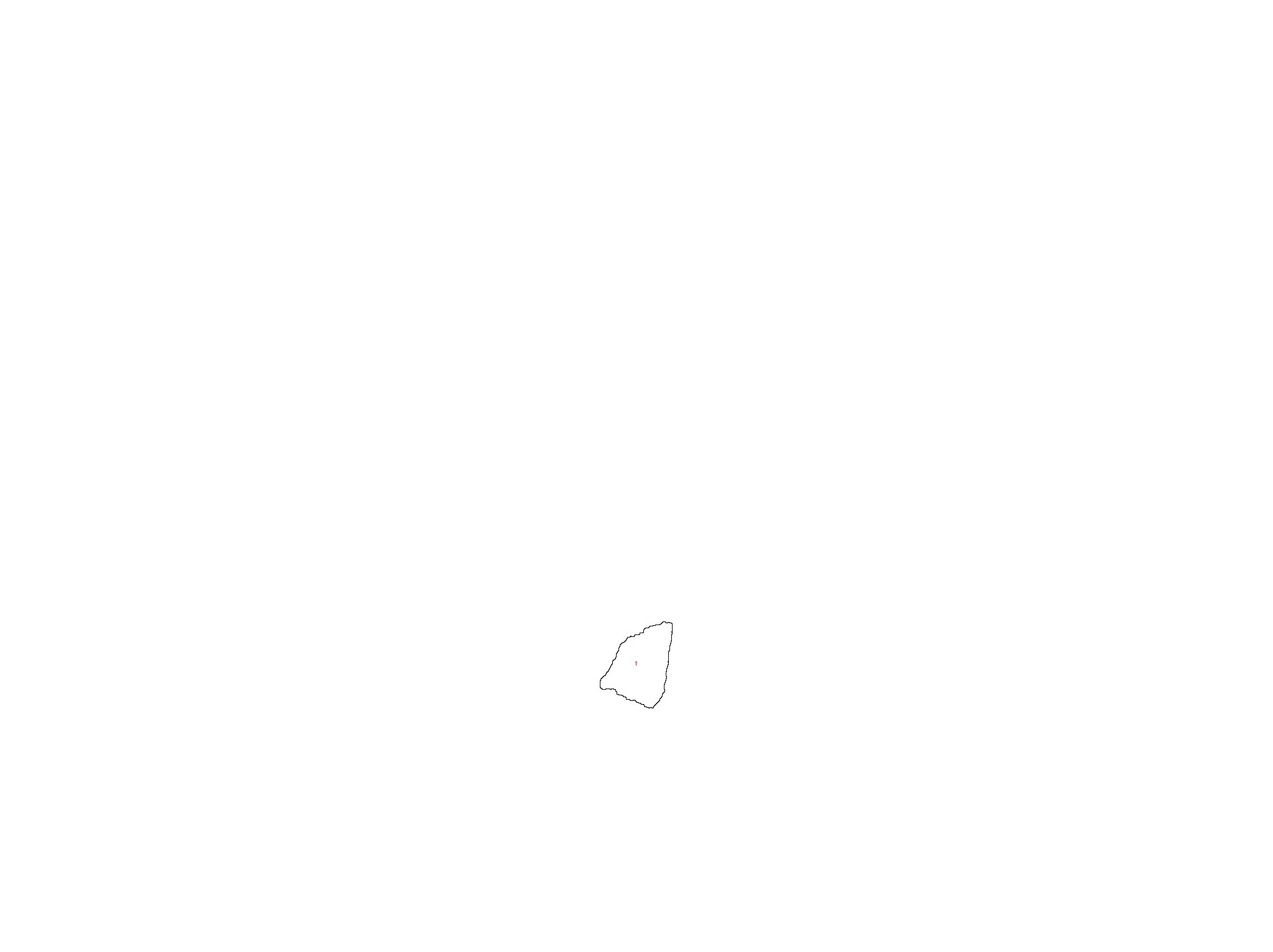

Supplement: S2 Dataset — (ZIP) [file pone.0304198.s005.zip › S2_Dataset_Raw_results_ImageJ/J2_200S_3040_2.jpg]

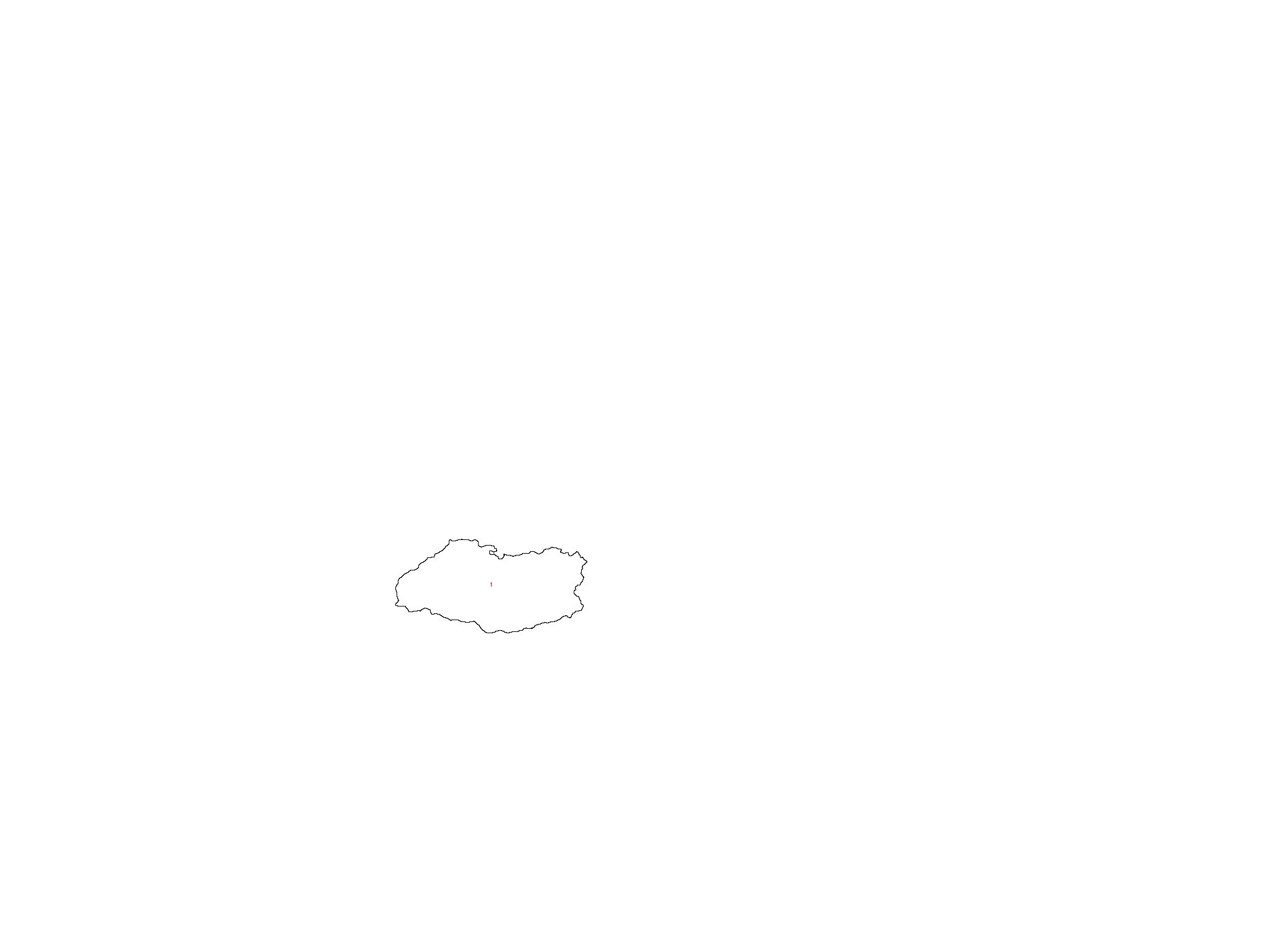

Supplement: S2 Dataset — (ZIP) [file pone.0304198.s005.zip › S2_Dataset_Raw_results_ImageJ/J2_200S_3040_3.jpg]

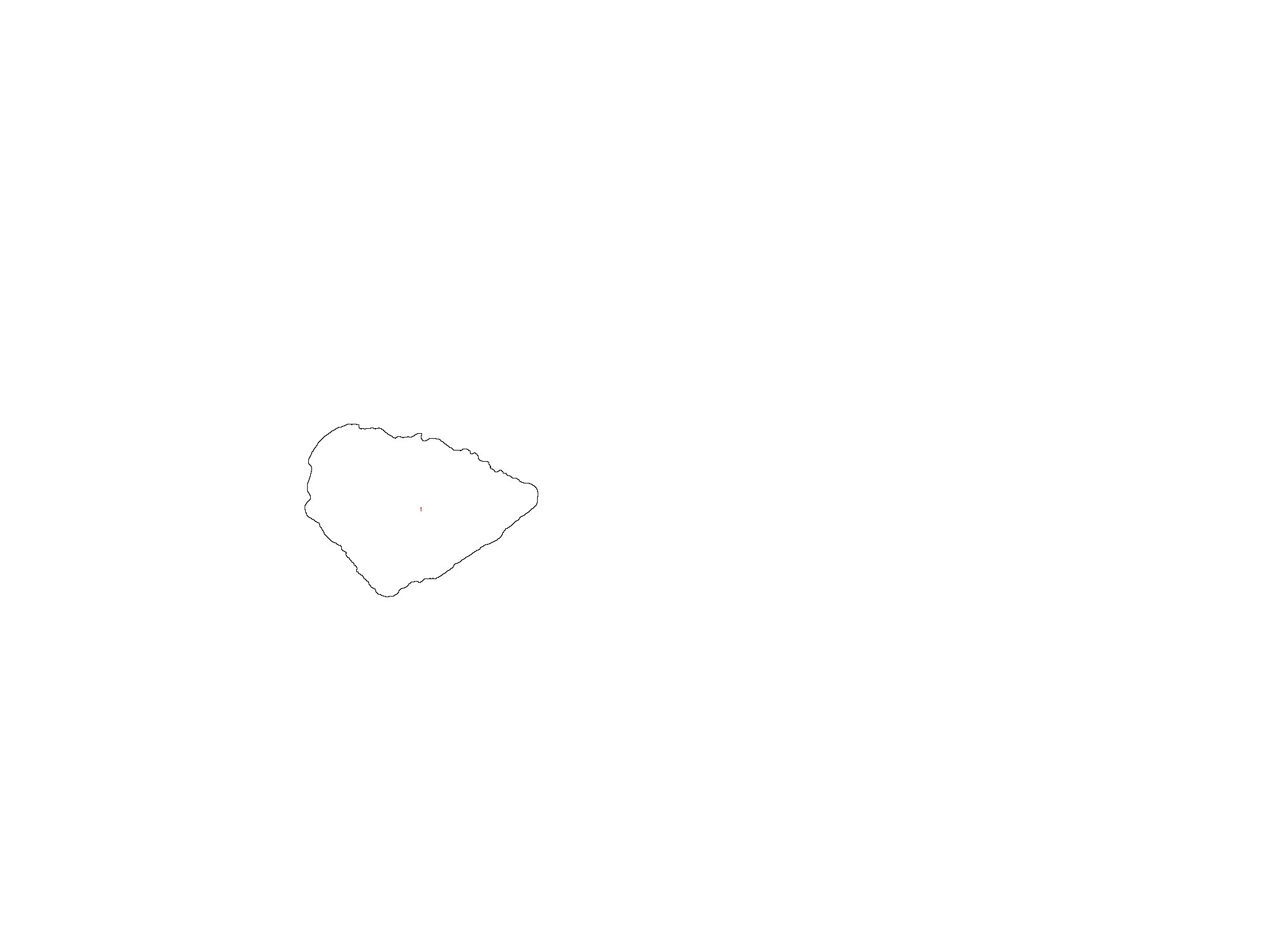

Supplement: S2 Dataset — (ZIP) [file pone.0304198.s005.zip › S2_Dataset_Raw_results_ImageJ/J2_200S_3040_4.jpg]

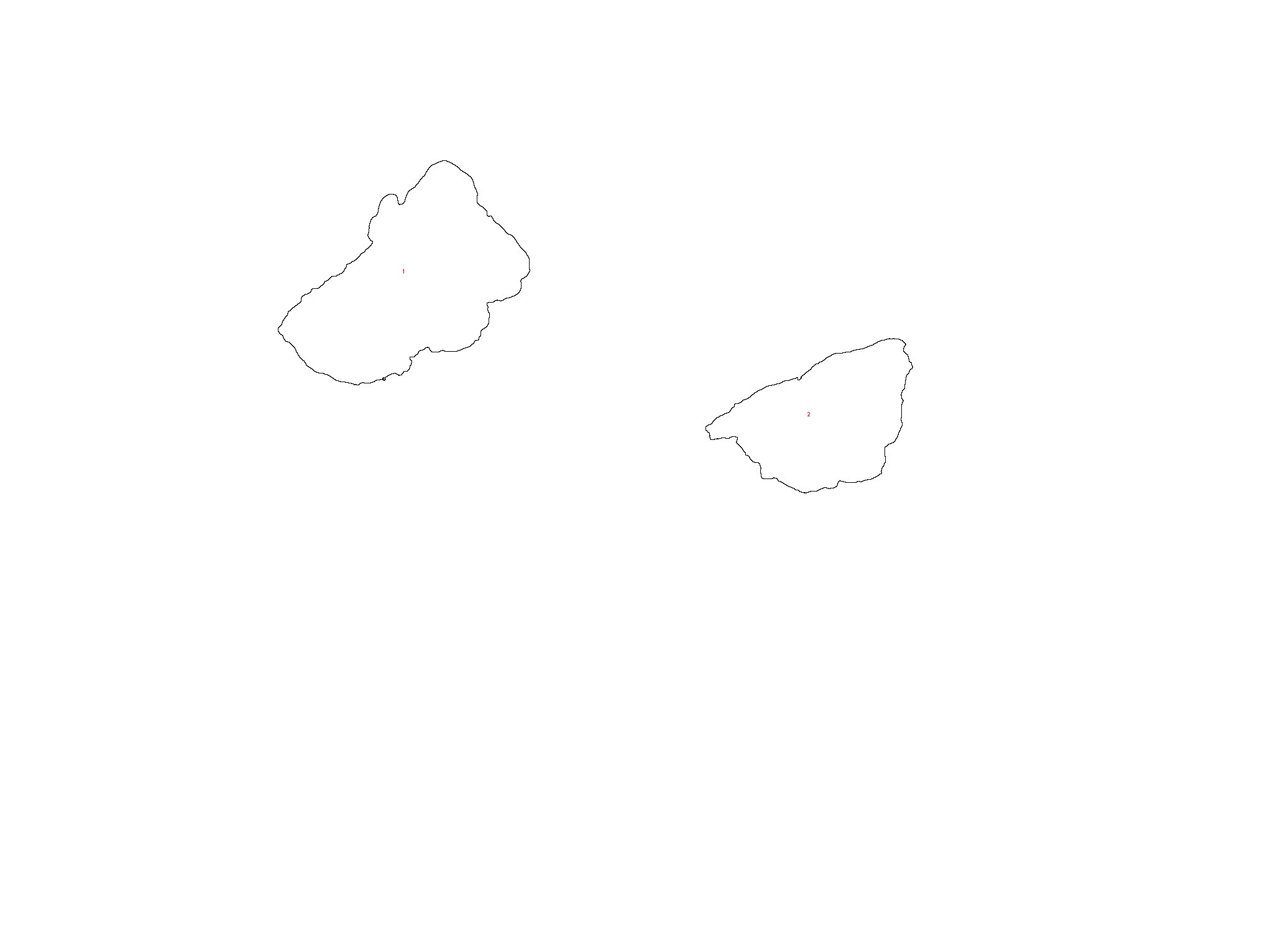

Supplement: S2 Dataset — (ZIP) [file pone.0304198.s005.zip › S2_Dataset_Raw_results_ImageJ/J2_200S_3040_5.jpg]

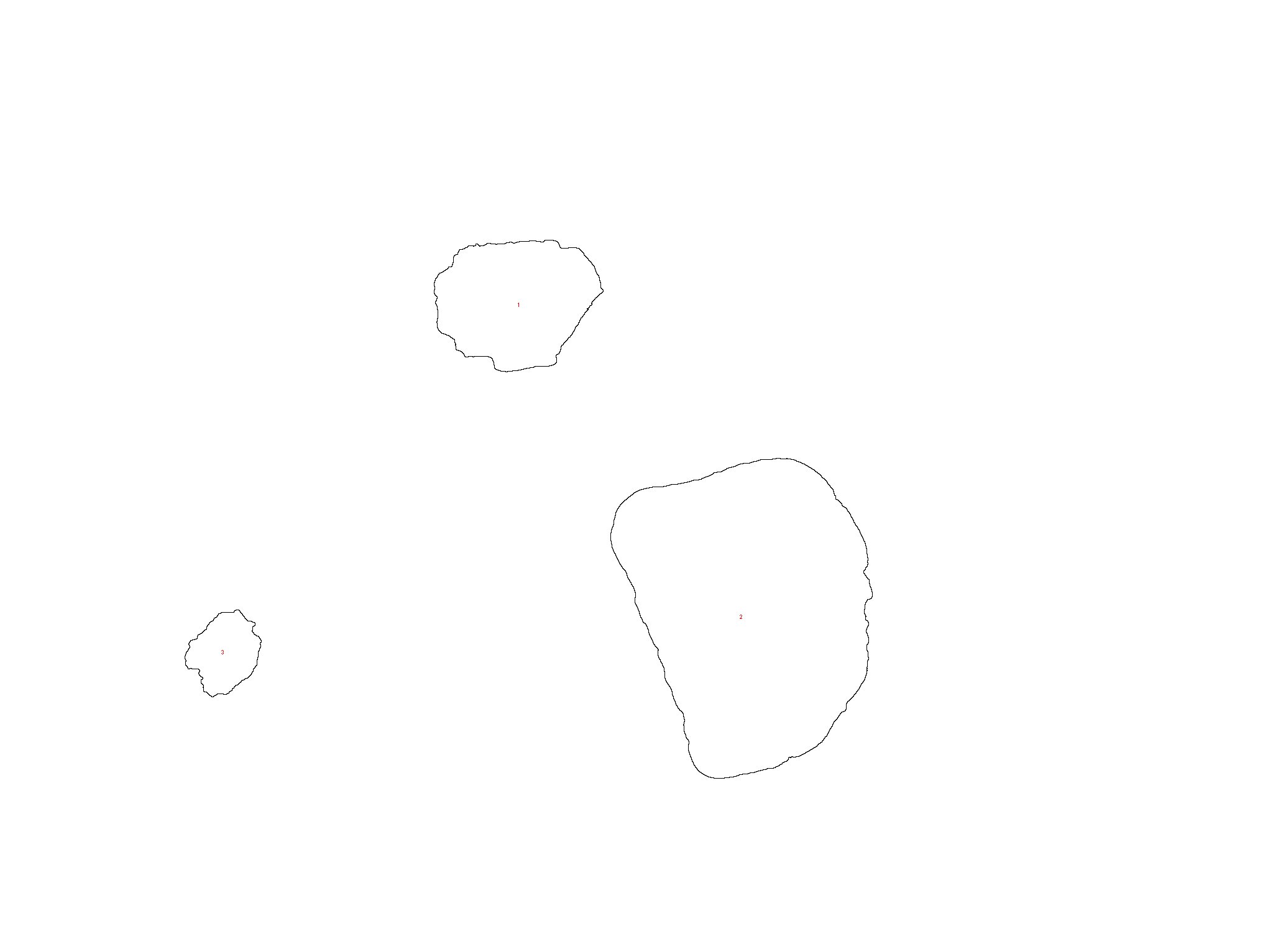

Supplement: S2 Dataset — (ZIP) [file pone.0304198.s005.zip › S2_Dataset_Raw_results_ImageJ/J2_200S_3040_6.jpg]
